# Allyl-isothiocyanate treatment induces a complex transcriptional reprogramming including heat stress, oxidative stress and plant defence responses in *Arabidopsis thaliana*

**DOI:** 10.1186/s12864-016-3039-x

**Published:** 2016-09-17

**Authors:** Ralph Kissen, Anders Øverby, Per Winge, Atle M. Bones

**Affiliations:** 1 Department of Biology, Norwegian University of Science and Technology (NTNU), NO-7491 Trondheim, Norway; 2 Present address: Center for Clinical Pharmacy and Clinical Sciences, School of Pharmaceutical Sciences, Kitasato University, Minato-ku, Tokyo, Japan

**Keywords:** Glucosinolates, Isothiocyanates, Transcriptomics, Microarray, Heat stress, Oxidative stress, Plant defence, Cell death

## Abstract

**Background:**

Isothiocyanates (ITCs) are degradation products of the plant secondary metabolites glucosinolates (GSLs) and are known to affect human health as well as plant herbivores and pathogens. To investigate the processes engaged in plants upon exposure to isothiocyanate we performed a genome scale transcriptional profiling of *Arabidopsis thaliana* at different time points in response to an exogenous treatment with allyl-isothiocyanate.

**Results:**

The treatment triggered a substantial response with the expression of 431 genes affected (*P* < 0.05 and log_2_ ≥ 1 or ≤ -1) already after 30 min and that of 3915 genes affected after 9 h of exposure, most of the affected genes being upregulated. These are involved in a considerable number of different biological processes, some of which are described in detail: glucosinolate metabolism, sulphate uptake and assimilation, heat stress response, oxidative stress response, elicitor perception, plant defence and cell death mechanisms.

**Conclusion:**

Exposure of *Arabidopsis thaliana* to vapours of allyl-isothiocyanate triggered a rapid and substantial transcriptional response affecting numerous biological processes. These include multiple stress stimuli such as heat stress response and oxidative stress response, cell death and sulphur secondary defence metabolism. Hence, effects of isothiocyanates on plants previously reported in the literature were found to be regulated at the gene expression level. This opens some avenues for further investigations to decipher the molecular mechanisms underlying the effects of isothiocyanates on plants.

**Electronic supplementary material:**

The online version of this article (doi:10.1186/s12864-016-3039-x) contains supplementary material, which is available to authorized users.

## Background

Isothiocyanates (ITCs) are a group of chemicals that can be generated by certain plants when secondary metabolites called glucosinolates (GSLs) are degraded by the enzymatic activity of myrosinase. Under certain reaction conditions other products such as nitriles and epithionitriles can be produced instead of ITCs [1, 2].

The effects of ITCs on human and animal cells are well documented such as modulating phase I and phase II enzymes, the antioxidant capacity, cell cycle and programmed cell death [3]. The role of ITCs in plant defence against insect pests and plant pathogens has also been extensively studied. ITCs have been shown to lead to reduced insect growth and development, as well as reduced offspring [4]. They also attract parasitoids of insect pests [5]. ITCs can lead to reduced bacterial proliferation and fungal growth [6, 7], although the molecular mechanisms leading to these effects are largely unknown.

As ITCs are generated upon plant tissue damage such as that occurring during herbivory, ITCs might in addition trigger plant defence responses. The effects of ITCs on the plants themselves have only been scarcely studied, but have gained more attention lately [8]. Incorporation into the soil of GSL-producing plant material or pure ITCs has been shown to have herbicidal activity [9, 10]. Application of high doses of ITCs directly onto plants has been shown to be phytotoxic while lower doses seem to render the plants more resistant to subsequent heat stress [11, 12]. Other studies have shown that an ITC treatment can lead to the closure of stomata in vitro [13, 14]. We have recently started to look at the molecular and cellular effects of ITCs in plants [15–18] in order to find the molecular mechanisms underlying some of these macroscopic observations.

To lay a basis for further mechanistic investigations we performed a genome scale transcriptional profiling of the *Arabidopsis thaliana* response to an exogenous treatment with allyl-isothiocyanate (allyl-ITC) at three time points: 30 min, 1 h and 9 h. Allyl-isothiocyanate is derived from the glucosinolate sinigrin, which is abundant in black mustard [19] as well as some accessions of the model plant *A. thaliana* [20]. The data illustrates that ITC in addition to its known toxic effect at higher doses elicits a complex and dynamic gene response that bears signatures of other abiotic and biotic stress responses. The aim of the present manuscript is to give a general overview of this transcriptional response, discuss in more detail some aspects of the response that we consider particularly interesting and point at some possible directions for further investigations of the effect of isothiocyanates on plant metabolic processes.

## Results and Discussion

### Extent and dynamics of the transcriptional response to allyl-ITC

**Fig. 1:**
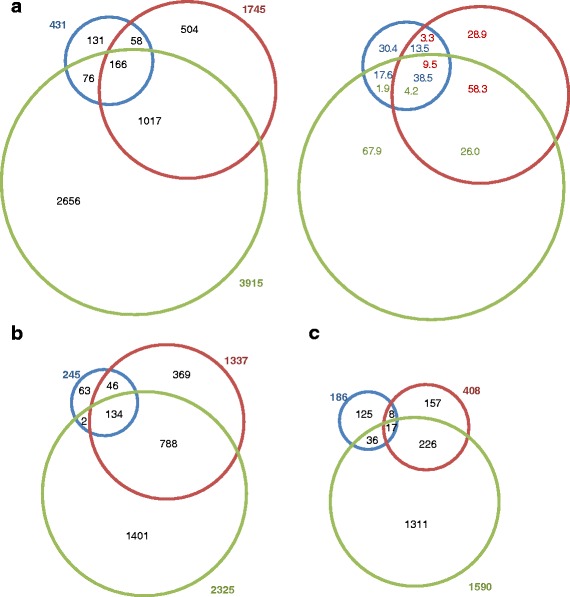
Overlap of genes whose expression is affected at the three time points. Venn diagrams showing the overlap of (**a**) affected (*P* < 0.05 and log_2_ ≥ 1 or ≤ -1) genes between 30 min (blue), 1 h (red) and 9 h (green) in absolute values (left) and percentages (right), (**b**) induced genes and (**c**) repressed genes

To analyse the early transcriptional response of *Arabidopsis thaliana* to an exogenous exposure with allyl-isothiocyanate (allyl-ITC), we performed genome scale transcriptional profiling by microarray at 30 min and 1 h. In addition, to assess the later *A. thaliana* response we chose a 9 h time point, after having performed pilot studies at different time points. Analysis at these three time points shows that the extent of the transcriptional response to the allyl-ITC treatment increased with the duration of exposure. Indeed, the number of genes whose expression was affected (*P* < 0.05 and log_2_ ≥ 1 or ≤ -1) increased from 431 after 30 min, to 1745 after 1 h and 3915 after 9 h of exposure to allyl-ITC (Fig. 1; Table 1; Additional file 1). At all three time points the majority of the affected genes were upregulated: 245 at 30 min, 1337 at 1 h and 2325 at 9 h. While at the 30 min and 9 h time points around 40 % of the genes were downregulated, the proportion of downregulated genes at 1 h decreased to 23 %.

**Table 1:** Number of genes whose expression levels are significantly affected by exposure to allyl-ITC at the three time points

	30 min	1 h	9 h
Upregulated	245	1337	2325
Downregulated	186	408	1590
Total	431	1745	3915

Also the overall intensity of the response varied between time points: the log_2_ value of the most upregulated gene increased from 4.7 to 8.3 to 11.6 from early to late time points, the log_2_ value of the most downregulated gene went from -3.5 to -3.8 to -4.8 (Table 2). The maximum absolute values for the mean and median were situated at the 1 h time point for upregulated genes but at the 9 h and 30 min time points respectively for the down regulated genes (Table 2).

**Table 2:** Intensity of the response to allyl-ITC

		30 min	1 h	9 h
Upregulated	Maximum	4.71	8.34	11.58
	Mean	1.61	2.26	2.12
	Median	1.37	1.87	1.72
Downregulated	Maximum	-3.54	-3.77	-4.79
	Mean	-1.25	-1.45	-1.50
	Median	-1.40	-1.29	-1.34

The values indicate the maximum, mean and median values of the log_2_ ratios of genes affected by allyl-ITC compared to the mock treatment

The numbers of genes that were only affected at one of the three time points were 131, 504 and 2656 at 30 min, 1 h and 9 h respectively (Fig. 1). This represented less than one third of the total number of affected genes at the two early time points and two thirds at the 9 h time point.

**Fig. 2:**
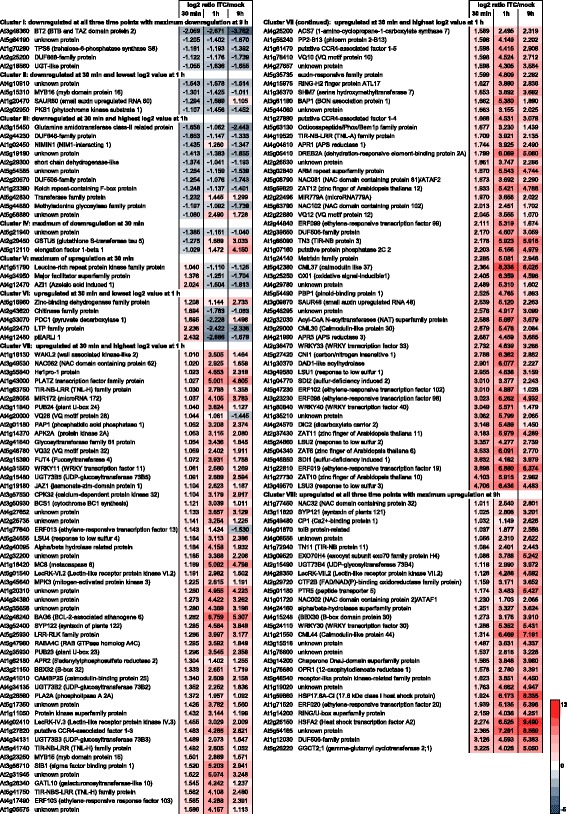
Dynamics of the allyl-ITC response. Genes significantly affected by the allyl-ITC treatment at all three time points are clustered based on the changes in log_2_ ratios ITC/mock between time points. The colour scale goes from blue (downregulated by allyl-ITC) to red (upregulated by allyl-ITC) and the extremes are set to cover the range of the log_2_ ratios for the most affected genes in the whole dataset

Early and later responses to allyl-ITC exposure differed thus in many of their characteristics. However as can be seen in Fig. 1, 38 % of the genes (i.e. 166) affected at the earliest time point were also affected at the two later time points (Fig. 1). When assessing in more detail the response dynamics of the genes affected at all three time points, 5 and 29 genes showed a continuous increase in down- and up-regulation from early to late time points (i.e. maximum of regulation at the 9 h time point) (Fig. 2; clusters I and VIII). Only 3 genes each showed their maximum of respectively down- and up-regulation at the earliest time point (clusters IV and V). Cluster VII was the largest cluster and contained 106 genes that were affected at all three time points with the highest induction at the 1 h time point (Fig. 2).

**Fig. 3:**
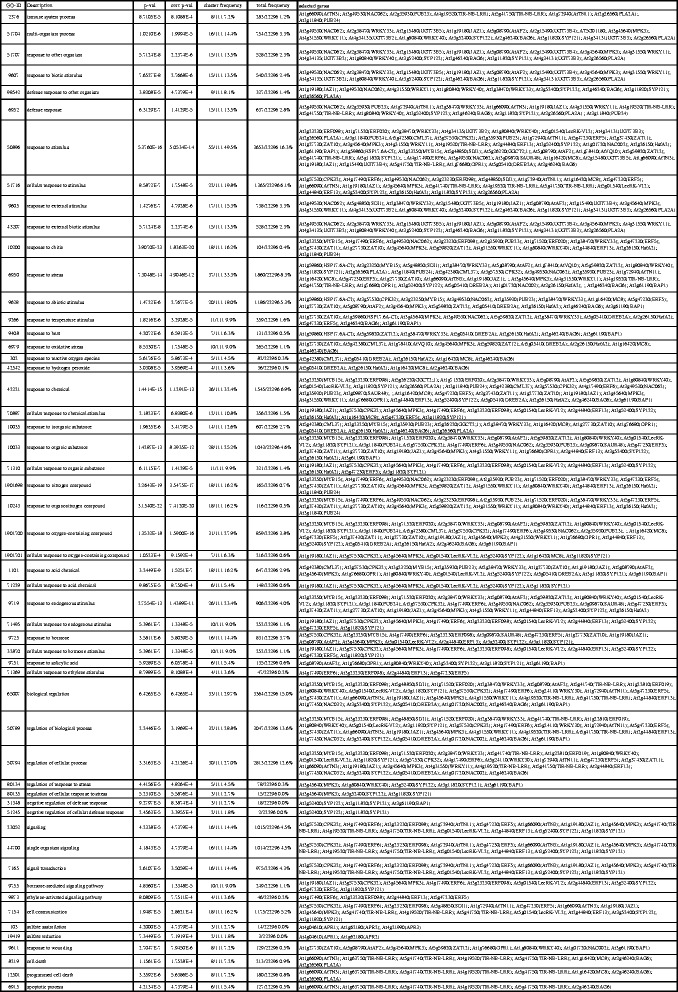
GO categories overrepresented among genes induced at the three time points. List of Gene Ontology (GO) categories overrepresented, as identified using BiNGO (hypergeometric test; Benjamini & Hochberg False Discovery Rate (FDR) correction; significance level 0.001), among the genes that were induced by allyl-ITC at all three time points

**Fig. 4:**
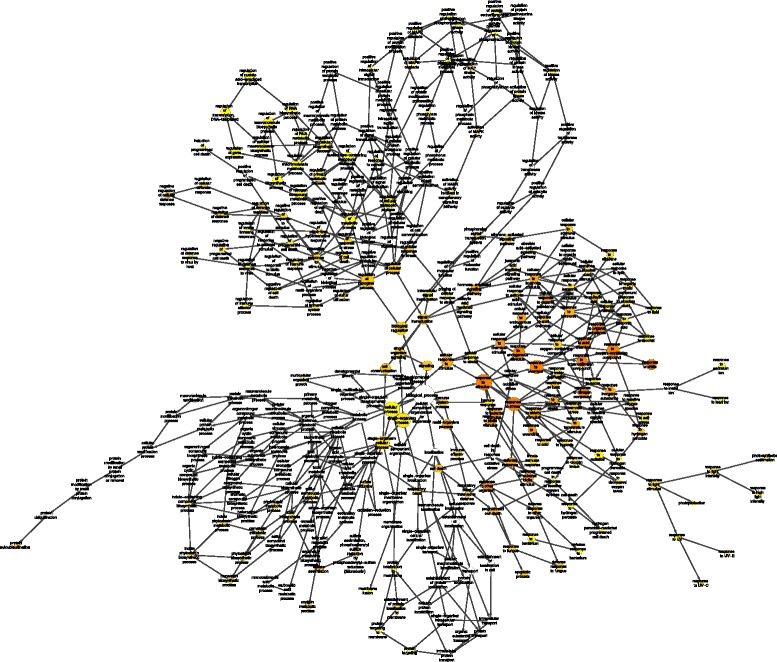
Network representation of overrepresented GO categories. Gene ontology (GO) categories that are overrepresented among the genes induced at all three time points of allyl-ITC treatment were identified and represented using the Cytoscape plugin BiNGO (hypergeometric test; Benjamini & Hochberg False Discovery Rate (FDR) correction; significance level 0.05)

Regarding the large number of genes that were affected by the allyl-ITC treatment, it is clear that describing all the transcriptional changes is beyond the scope of any article. The analysis of gene ontology (GO) categories overrepresented among the affected genes indicated that a considerable number of different biological processes were affected. Interesting patterns were revealed during these analyses, such as for example multiple responses to stress and stimuli, signalling, death and innate immunity for genes upregulated at all three time points (Figs. 3 and 4). We chose, therefore, a triple approach for this article: 1) relate transcriptional changes to the biosynthetic steps connected to the generation of ITCs, 2) show transcriptional changes that might explain effects of ITCs on plants that were reported in the literature and 3) present what we consider to be some of the most interesting aspects of this study that incite further investigation.

### Allyl-ITC shows a differential effect on the expression of genes involved in GSL biosynthesis and degradation

**Fig. 5:**
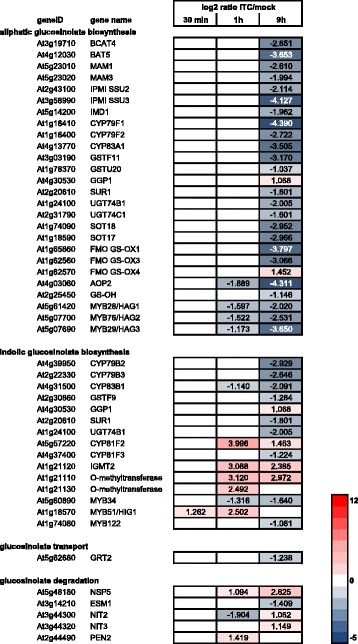
Glucosinolate-related genes affected by allyl-ITC. Changes in expression levels (log_2_ ratios) of genes involved in the biosynthesis, transport and degradation of glucosinolates after 30 min, 1 h and 9 h of allyl-isothiocyanate treatment, compared to the mock treatment. Only values for genes significantly affected at one time point at least are indicated. The colour scale goes from blue (downregulated by allyl-ITC) to red (upregulated by allyl-ITC) and the extremes are set to cover the range of the log_2_ ratios for the most affected genes in the whole dataset

Considering the fact that isothiocyanates such as allyl-ITC are degradation products of glucosinolates (GSLs), the response of genes involved in their biosynthesis and catabolism is of particular interest. Only twelve GSL-biosynthetic genes were significantly affected at the two early time points of 30 min and 1 h (Fig. 5). Among them were genes (putatively) involved in the biosynthesis of indolic GSLs such as *CYP81F2*, *IGMT2* and related *O-methyltransferase* [21], which were all upregulated. Most of these were also induced at the last time point of 9 h. Interestingly, the transcription factor *MYB51/HIG1* [22] was the only gene of this pathway that was affected more than two-fold after 30 min. In addition it was the only member of the *MYB* genes regulating GSL biosynthetic genes that was induced by allyl-ITC. Indeed the great majority of genes involved in the biosynthesis of aliphatic GSLs (*CYP79F1*, *CYP79F2*, *CYP83A1*, *FMO*_*GS-OX1*_, *MYB28*, *MYB29*, *MYB76*) and indolic GSLs (*CYP79B2*, *CYP79B3*, *CYP83B1*, *MYB34*, *MYB122*) [23] were downregulated, especially at the latest time point (Fig. 5). This indicates that the treatment with exogenous ITC might have a negative feedback on GSL biosynthesis, although the effect on GSL levels would not be expected to be seen after such a short exposure time. Whether this negative feedback is mediated directly by the GSL degradation product ITC, due to depletion of glutathione (GSH) [16] which is the sulphur donor in the biosynthesis of GSL [24] or through a sulphur starvation response (discussed later) would require further testing. In contrast to the downregulation of most GSL-biosynthetic genes, some of the genes involved in the degradation of GSLs were induced after 9 h of allyl-ITC treatment. Although the myrosinases *TGG1* and *TGG2* were not affected at the gene expression level, the atypical myrosinase *PEN2* was induced [25]. *NSP5*, one of the nitrile-specifier proteins known to divert GSL hydrolysis from the generation of ITCs to that of nitriles [26] was also induced. Concomitantly, the expression of the gene encoding epithiospecifier modifier 1 (ESM1) that favours the formation of ITCs [27] was reduced. The genes for nitrilases NIT2 and NIT3, which have been shown to act in vitro on several GSL-derived nitriles to generate a carboxylic acid and ammonia [28] and hypothesized to be involved in the endogenous catabolism of GSLs [29], were also induced. These gene expression changes indicate that the plant might sense the presence of exogenously applied ITC, reduce the biosynthesis of GSLs and prevent the generation of endogenous ITC from GSL breakdown by favouring the generation of nitriles and their further catabolism by nitrilases. It would therefore be interesting in future experiments to follow the levels of GSLs and their degradation products in plants exposed to ITC for a longer period of time.

### Allyl-ITC affects genes involved in glutathione homeostasis

ITCs have been shown to conjugate to glutathione (GSH) in humans, rats and insects by non-enzymatic or enzymatic processes, the enzymatic conjugation being mediated by glutathione transferases (GSTs) [30–33]. Studies in various mammalian systems have shown that ITCs can lead to an induction of GST gene expression levels and higher GST activities (for review [34]). In our present study, the expression of several GSTs was affected by the allyl-ITC treatment, the vast majority being induced after 9 h of allyl-ITC exposure. A detailed description of the effects of allyl-ITC on the expression of different GSTs will be published elsewhere (Øverby et al., in preparation).

As a conjugation of ITCs to GSH has, to our knowledge, not yet been documented in vivo for plants it would be interesting to attempt the detection of GSH-ITC and related conjugates in plant tissue. The reversible nature of this conjugation may however complicate the analysis and as this reaction can occur non-enzymatically, the presence of these conjugates would not definitely prove the implication of GSTs in this process either.

The expression of *DHAR2* (At1g75270) was also induced by ITC. Although DHARs belong to the GST superfamily, they do not carry out classic GST-type conjugations but reduce dehydroascorbate to ascorbate, concurrently oxidising GSH to glutathione disulphide (GSSG) [35].

If exposure to allyl-ITC leads to a conjugation with GSH and/or an oxidation of GSH to GSSG as the gene expression patterns might indicate, then levels of GSH would be expected to decrease unless the plant increases GSH biosynthesis and/or reduces GSSG to GSH. In a previous study with allyl-ITC, we showed that GSH levels decreased to about half after 1 h treatment and were maintained for the next two hours of the experiment [16]. The increased expression (log_2_ = 1.8) of the glutathione reductase *GR1* (At3g24170), responsible for the reduction of GSSG to GSH in the cytosol, after 9 h of ITC treatment in the current experiment might indeed indicate that at least part of the GSH pool is maintained. There was no indication at the transcriptional level of an increased synthesis of GSH as neither *GSH1* (At4g23100) nor *GSH2* (At5g27380), encoding the enzymes that catalyse the two steps leading from cysteine to GSH, were induced. However, the fact that total GSH levels can increase without the induction of *GSH1* and *GSH2* transcripts under oxidative stress has been reported [36].

Also genes encoding enzymes believed to be involved in the processing of GSSG and GSH conjugates were affected by allyl-ITC treatment. Hence the gene encoding the apoplastic γ-glutamyl transpeptidase GGT1 (At4g39640; [37]) and the cytosolic phytochelatin synthase PCS1 (At5g44070; [38]) and γ-glutamyl cyclotransferase GGCT2; 1 (At5g26220; [39]) were induced more than four-fold at 1 h and 9 h.

It was also recently shown that exposure to 4-methylsulphinylbutyl-ITC (sulforaphane) led to a depletion of total glutathione after 30 min [40]. It would therefore be interesting to complement our previous measurements of GSH during allyl-ITC exposure, by following the levels of GSSG and total glutathione levels, as well as those of precursors (e.g. cysteine, *O*-acetylserine, γ-glutamylcysteine; see below).

### Allyl-ITC effect on genes of sulphate uptake and assimilation

Sulphate uptake and assimilation is regulated by many factors [41, 42], such as environmental factors, hormones, the availability of sulphur and the levels of reduced sulphur such as cysteine and glutathione. High levels of GSH are known to repress the expression of genes involved in sulphate uptake and assimilation [43], while sulphur starvation conditions induce their expression [44–46].

Glucosinolate metabolism is intimately linked to sulphur. The core structure of GSLs contains sulphur that is incorporated at two steps during the biosynthesis, via GSH and PAPS [47, 48]. Many of the aliphatic GSLs also contain a sulphur group in their side chain as this is derived from the sulphur-containing amino acid methionine [23]. During the hydrolysis of GSLs to generate ITCs, sulphate is released [2]. The availability of sulphur has been shown to have an effect on the expression of GSL biosynthetic genes and on the content of GSLs, especially those derived from methionine [49].

**Fig. 6:**
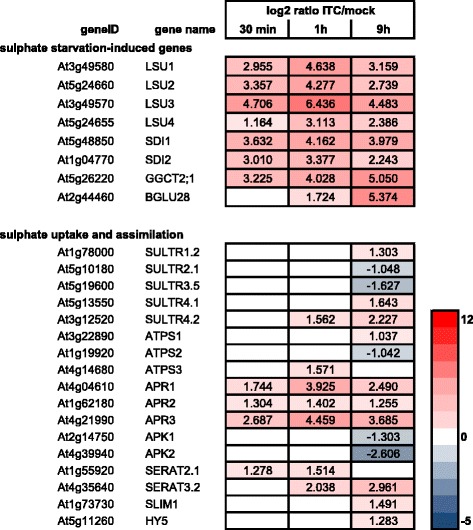
Effect of allyl-ITC on sulphur response. Changes in expression levels (log_2_ ratios) of selected sulphur starvation-induced genes and of genes involved in sulphate uptake and assimilation after 30 min, 1 h and 9 h of allyl-ITC treatment, compared to mock treatment. Only values for genes significantly affected at one time point at least are indicated. The colour scale goes from blue (downregulated by allyl-ITC) to red (upregulated by allyl-ITC) and the extremes are set to cover the range of the log_2_ ratios for the most affected genes in the whole dataset

Known sulphate starvation-induced genes such as sulphur-deficiency induced *SDI1* (At5g48850) and *SDI2* (At1g04770), response to low sulphur *LSU1* (At3g49580), *LSU2* (At5g24660) and *LSU3* (At3g49570) and the γ-glutamyl cyclotransferase *GGCT2;1* (At5g26220) [39, 50–52] were among the most highly induced genes after 30 min of allyl-ITC treatment (Fig. 6; Additional file 1). Therefore, we had a closer look at the effect of allyl-ITC on genes involved in sulphate uptake and assimilation.

**Fig. 7:**
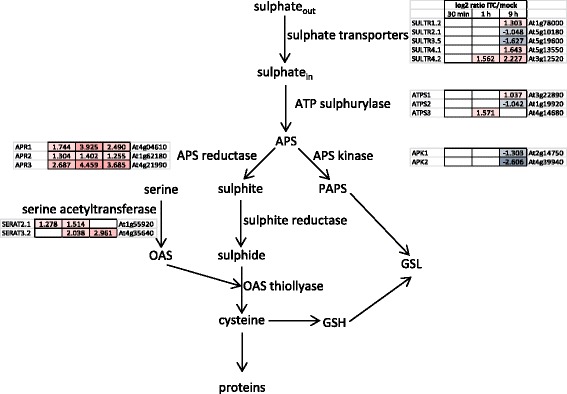
Diagram of the allyl-ITC effect on sulphate uptake and assimilation. Diagram showing gene expression changes (log_2_ ratios) in the early steps of sulphate uptake and assimilation after 30 min, 1 h and 9 h exposure to allyl-ITC. ATPS: ATP sulphurylase, APS: adenosine 5′-phosphosulphate, PAPS: 3′-phosphoadenosine 5′-phosphosulphate, APK: APS kinase, APR: APS reductase, SERAT: serine acetyltransferase, OAS: *O*-acetylserine, GSH: glutathione, GSL: glucosinolates

Several key genes of the sulphate uptake and assimilation pathways were affected by our allyl-ITC treatment (Figs. 6 and 7). The high affinity sulphate transporter *SULTR1;2* (At1g78000) [53] was induced after 9 h of allyl-ITC treatment. Also the genes encoding the sulphate transporters SULTR4;1 (At5g13550) and SULTR4;2 (At3g12520) that remobilize vacuolar sulphate by mediating its efflux from the vacuole under low sulphur conditions [54] were induced by allyl-ITC. Interestingly, the coexpressed SULTR2;1 and SULTR5;3 believed to interact in retrieving apoplastic sulphate to xylem parenchyma cells and contribute to its root-to-shoot transport [55] were both repressed at the gene level. Another major control point of the pathway, besides sulphate transport, is APS reductase which reduces adenosine 5′-phosphosulphate (APS) to sulphite [43, 56]. The genes encoding the three APR isoforms, in particular *APR1* (At4g04610) and *APR3* (At4g21990), were induced at all three time points, with a maximum after 1 h of allyl-ITC treatment. Also the genes encoding the ATP sulphurylases ATPS1 (At3g22890) and ATPS3 (At4g14680) were induced after 9 h and 1 h respectively. Genes encoding enzymes catalysing the further steps leading from sulphite to cysteine, sulphite reductase and OAS thiollyase were not affected by our allyl-ITC treatment.

One of the central metabolites in sulphate assimilation is *O*-acetylserine (OAS), which has been proposed to have a signalling function in sulphate starvation [57]. OAS accumulates during sulphur starvation and the effect of sulphur starvation can be mimicked by OAS treatment [44]. *A. thaliana* possesses five genes that encode a plastidic, a mitochondrial and three cytosolic isoforms of serine acetyltransferase (SERAT or SAT), the enzyme that catalyses the synthesis of OAS from serine [58]. Of these five genes, the ones encoding the plastidic SERAT2.1 (At1g55920) and the cytosolic SERAT3.2 (At4g35640) isoforms were induced by ITC, the former being upregulated at an earlier time point and the latter to a higher extent.

Consistent with the downregulation of GSL biosynthetic genes discussed above (Fig. 5), the genes encoding the two APS kinases APK1 (At2g14750) and APK2 (At4g39940) that generate PAPS (3′-phosphoadenosine 5′-phosphosulphate) important for the synthesis of sulphated metabolites [59], such as GSLs, were down regulated after 9 h.

How genes involved in sulphate uptake and assimilation are transcriptionally regulated is less well known. The SLIM1 (sulphur limitation 1; At1g73730) transcription factor has been identified as being one of the central regulators of sulphur uptake and assimilation by regulating the expression of several sulphate transporters and other sulphate responsive genes. Although it was reported not to be modulated by changes in sulphur conditions [50], *SLIM1* was upregulated (log_2_ = 1.491) after 9 h of allyl-ITC treatment. Similarly, the HY5 (elongated hypocotyl 5; At5g11260) transcription factor known to regulate the expression of APR1 and APR2 [60] was induced (log_2_ = 1.283) after 9 h. Also MYB transcription factors involved in the regulation of GSLs have been reported to regulate the expression of some of the APR, ATPS and APK-encoding genes [61, 62]. As described previously in the text, all the GSL-related MYBs were downregulated by allyl-ITC, except *MYB51* (Fig. 5).

Based on the expression profile of sulphate uptake and assimilation genes, one could predict an increased flux through this pathway after ITC exposure, leading to increased production of OAS and sulphite at the expense of PAPS and GSL. Sulphite is however cytotoxic and very reactive and accumulation of reduced sulphur would exert a different effect on the regulation of sulphate uptake and assimilation than OAS. In addition regulation of the pathway at the posttranscriptional and posttranslational levels [42] could change the predicted metabolic outcome. It would therefore be interesting to monitor the cellular levels of some of the compounds (e.g. sulphate, cysteine, OAS, GSH and GSSG; Fig. 7) during a prolonged allyl-ITC exposure.

Whether the effect we see on expression levels of genes regulated by sulphate starvation is mediated directly by sensing the presence of allyl-ITC or is an indirect effect due to the demand for reduced sulphur (e.g. as GSH is depleted through conjugation and oxidation) or another process affecting sulphate assimilation is difficult to assess at this point. One could hypothesize that an excess of GSL hydrolysis products fools the plant to believe that the sulphur-containing GSLs are broken down and that it consequently would increase the rate of sulphate assimilation in order to maintain the level of GSLs. The observed downregulation of APK-encoding genes, of many GSL biosynthetic genes and related MYB transcription factors would however argue against this. In addition, a myrosinase-catalysed hydrolysis of GSLs would lead to the release of sulphate, in which case many of the genes that were induced would be expected to be repressed [41, 42].

### Allyl-ITC treatment leads to a heat stress response

**Fig. 8:**
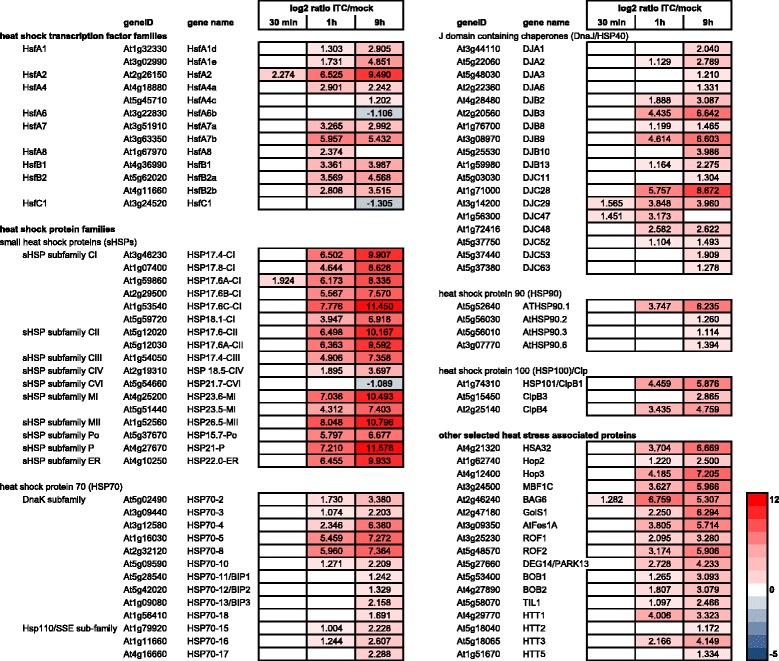
Heat stress response after allyl-ITC treatment. Changes in expression levels (log_2_ ratios) of genes involved in the heat stress response after 30 min, 1 h and 9 h of allyl-isothiocyanate treatment, compared to mock treatment. Only values for genes significantly affected at one time point at least are indicated. The colour scale goes from blue (downregulated by allyl-ITC) to red (upregulated by allyl-ITC) and the extremes are set to cover the range of the log_2_ ratios for the most affected genes in the whole dataset

Heat stress triggers a complex and multifaceted response in plants [63]. One of the aspects of this response involves the transcription of genes encoding heat shock proteins (HSPs) under control of heat shock transcription factors (Hsfs) by their binding to heat stress promoter elements [64]. Numerous genes encoding Hsfs and HSPs were among the most highly induced genes after 1 h and 9 h of allyl-ITC treatment (Fig. 8). Nine of the ten most highly induced genes at 9 h were Hsfs or HSPs (Additional file 1).

The expression of thirteen of the twenty-one known *A. thaliana* Hsfs [64] was affected by the allyl-ITC treatment at one time point at least. *HsfA2* (At2g26150) was the earliest and most highly induced Hsf throughout the experiment, interestingly showing an induction above log_2_ = 2 already after 30 min and close to log_2_ = 9.5 at the 9 h time point. HsfA2 plays a central role in the heat stress response and thermotolerance but is also a key regulator of the plant response to several other abiotic stresses [65, 66]. HsfA2 regulates the expression of several target genes, including various *sHSPs* [65–67] affected in our experiment. It is itself regulated by HsfA1d (At1g32330) and/or HsfA1e (At3g02990), which belong to the A1 class Hsfs considered as being the master regulators of the heat stress response [68–70]. HsfA1d/HsfA1e control the expression of other Hsfs such as HsfA7a (At3g51910), HsfA7b (At3g63350), HsfB1 (At4g36990) and HsfB2a (At5g62020) [69]. Upon allyl-ITC treatment, the transcriptional activation of *HsfA2* occurs however earlier than that of *HsfA1d* and *HsfA1e*, and also *HsfA7a*, *HsfA7b*, *HsfB1* and *HsfB2a* showed a higher induction by allyl-ITC at the 1 h time point than *HsfA1d/HsfA1e*. Allyl-ITC also induced *HsfB2b* (At4g11660), another member of the class B Hsfs, at the 1 h and 9 h time points. *HsfB1* and *HsfB2b* are rapidly induced by heat stress and have been shown to negatively regulate the expression of heat stress induced *Hsfs* and *HSPs* and of defence related genes [71, 72]. HsfA7a is important for heat acclimation [73], and *HsfA7a* is induced prior to *HsfA2* upon heat stress [74]. The opposite was observed under allyl-ITC treatment. Both members of the HsfA4 class, *HsfA4a* (At4g18880) and *HsfA4c* (At5g45710), were induced by allyl-ITC but to a lesser extent than most other Hsfs. Also, *HsfA4a* (At4g18880) was induced earlier and stronger than *HsfA4c* (At5g45710). It has been shown that both act as activators of heat stress gene expression [75] and in the case of HsfA4 also of oxidative stress responsive genes [76]. *HsfA8* (At1g67970) was only induced at the 1 h time point. Little is known about this Hsf but its expression is induced by HsfA4 overexpression [76] and it has recently been characterized as a redox-sensitive transcription factor that translocates to the nucleus upon oxidative stress [77].

Two points should be pointed out regarding allyl-ITC treatment and the heat stress response. First, HsfA3 (At5g03720) whose expression is heat stress induced, is dependent on DREB2A and DREB2C and triggers the induction of other heat stress related genes [78, 79], was not affected by allyl-ITC treatment at any of the three time points whereas *DREB2A* (At5g05410) and *DREB2C* (At2g40340) were induced (Additional file 1).

Second, only the two Hsfs *HsfA6* (At3g22830) and *HsfC1* (At3g24520) were downregulated by an exposure to allyl-ITC. Interestingly, these two genes are under positive regulation of the transcription factor NAC019 (At1g52890), which is induced by heat stress [80] but repressed by allyl-ITC (Additional file 1).

Although small heat shock proteins (sHSPs) are also induced by other abiotic stresses, it was proposed that sHSPs protect thermo-sensitive substrates from irreversible heat stress-induced denaturation and aggregation [81]. Of the 19 known *A. thaliana* genes encoding sHSPs, 16 were induced after 1 h and 9 h of allyl-ITC treatment, with invariably a higher induction at 9 h (Fig. 8). This mimics the unequivocal transcriptional response of these HSPs to heat stress conditions [81, 82].

It has been recently shown that the expression of *HSP70-4* (At3g12580), *HSP70-5* (At1g16030) and *HSP70-8* (At2g32120) was transiently increased, with a maximum at 1 h after treatment, when 21 day old plants were sprayed with a 2 mM phenethyl-ITC solution and monitored for 48 h [11]. We confirm that of the 18 *A. thaliana* HSP70-encoding genes [83, 84] these three genes were the most highly induced by our allyl-ITC treatment, but an additional nine *HSP70s* were revealed to be upregulated (Fig. 8). Also, all affected *HSP70s* were more highly induced after 9 h than after 1 h in our study. HSP70s are ATP-dependent chaperones involved in processes such as folding of *de novo* synthesized proteins and refolding of misfolded proteins and aggregated proteins [85], although the role of many of them in *A. thaliana* is still unclear. J-domain containing proteins (DnaJ/HSP40) are cochaperones of HSP70s [86]. More than 100 genes encoding DnaJ proteins have been identified in *A. thaliana* [82] and eighteen of these were induced to various degrees by allyl-ITC (Fig. 8). The reason for this and its biological significance are not known. For those DnaJ genes that were affected at two time points, the induction was higher after 9 h of allyl-ITC treatment than after 1 h, similar to the situation observed for *sHSPs* and *HSP70s* (Fig. 8).

Another family of HSPs are the HSP90s that exert their chaperone activity on a select number of client proteins involved in the heat stress response but also hormone signalling and developmental processes [87–89]. They have also been implicated in plant defence by regulating the activity of several so-called R (for resistance) proteins through complex formation [90–92]. Four *HSP90s* were induced by allyl-ITC, with the gene At5g52640 encoding the cytosolic HSP90.1 being most highly induced (Fig. 8). HSP90.1 expression is induced by heat stress and it physically interacts with HsfA2 [93, 94]. But HSP90.1 expression is also induced after pathogen challenge, interacts with disease resistance signalling components and is required for resistance mediated by RPS2 and cell death during the hypersensitive response (HR) [90]. The HSP90 and HSP70 chaperone machineries are connected via the tetratricopeptide repeat (TPR)-containing Sti1/Hop protein, which binds to both HSPs and allows the transfer of the client protein from HSP70 to HSP90 [95]. Although we are not aware of this having been described in plants, the heat induced genes *Hop2* (At1g62740) and *Hop3* (At4g12400) encoding TPR-containing proteins [96] were induced by allyl-ITC (Fig. 8).

Three members of the heat shock protein 100 (HSP100)/casein lytic proteinase (Clp) subclass B [97, 98] were induced by allyl-ITC: *HSP101/ClpB1* (At1g74310), *ClpB3* (At5g15450) and *ClpB4* (At2g25140) (Fig. 8). All three are induced by heat stress and the important role of HSP101 in thermotolerance is well documented [98–102]. HSP101 is hypothesized to act in a positive feedback loop with HSA32 (heat stress associated 32-kD protein, At4g21320) in the memory of heat acclimation [103, 104], a gene that was also induced by allyl-ITC after 1 h and 9 h.

The massive Hsf-controlled induction of HSPs, which act as molecular chaperones to protect proteins against denaturation and to facilitate refolding, is one aspect of the heat stress response in plants. But also other factors are involved in the protection against heat-induced damage and different signalling pathways such as abscisic acid (ABA), salicylic acid (SA), ethylene and oxidative burst seem to be involved in thermotolerance [99, 105].

*MBF1C* (At3g24500), one of the three genes encoding the highly conserved transcriptional co-activator MBF1 (multiprotein bridging factor 1) in *A. thaliana*, is induced by several stresses such as pathogen infection, salinity, drought, hydrogen peroxide (H_2_O_2_), and application of the plant hormones ABA or SA. It has also been identified as key regulator of thermotolerance that functions upstream of (trehalose, SA and) ethylene during heat stress [106]. *MBF1C* was highly upregulated by 1 h and 9 h (and log_2_ = 0.926 at 30 min) of allyl-ITC treatment (Fig. 8), whereas its interaction partner *TPS5* (trehalose phosphate synthase 5), also a heat-induced gene, was not induced by allyl-ITC. Of the ten other genes encoding TPS-like or active TPS (i.e. *TPS1*, *TPS2* and *TPS4*; [107]), nine were either downregulated or not affected by allyl-ITC and only *TPS2* (At1g16980) was induced after 9 h. Interestingly, overall only half (i.e. 49 out of 87) of the genes that showed an elevated expression in plants constitutively expressing *MBF1C* and grown under control conditions [108] were also induced at 1 h of allyl-ITC treatment. However, all eight of the ethylene-associated transcripts, comprising seven ethylene response factors (*ERF*s) and ACC synthase 6 (*ACS6*) were induced by allyl-ITC (Additional file 2A). This might indicate that the allyl-ITC-triggered heat stress response via MBF1C involves ethylene but not trehalose. Among the genes whose induction under heat stress was dependent on MBF1C [109] it is interesting to note that the two heat shock transcription factors *HsfB2a* (At5g62020) and *HsfB2b* (At4g11660), the transcriptional regulator *DREB2A/ERF045* (At5g05410) and four zinc finger protein genes were also induced by ITC after 1 h (Additional file 2B).

The expression of several other genes reported to be involved in the heat stress response was also affected by allyl-ITC treatment, some of which will be discussed shortly below. The gene encoding the Bcl-2–associated athanogene (BAG) protein BAG6 (At2g46240), belonging to a family of chaperone regulators that interact with HSP70 and HSC70 (heat shock cognate 70) proteins, was rapidly and strongly induced by allyl-ITC treatment, with the highest induction after 1 h, similar to its responsiveness to heat [110, 111]. *BAG6* was also identified as a target gene of HsfA2 [112]. Galactinol synthase 1 (GolS1; At2g47180) which is implicated in raffinose synthesis under heat stress and whose expression is controlled by HsfA1b and HsfA2 [65, 113], was induced by allyl-ITC treatment at 1 h and 9 h. At3g09350 coding for AtFes1A, a heat induced protein that associates with HSP70 and prevents its degradation [114], was induced at 1 h and 9 h by allyl-ITC. The genes encoding the prolyl cis-trans isomerase cochaperones ROF1 (At3g25230) and ROF2 (At5g48570) were also induced at 1 h and 9 h of allyl-ITC treatment. ROF1 is heat stress induced, binds HSP90.1 and affects the accumulation of HsfA2-regulated sHSPs [94, 115]. DEG14/PARK13 (At5g27660) was the only member of the DEG/HtrA (high-temperature requirement A) protease family involved in protein quality control [116] that was upregulated by allyl-ITC. DEG14/PARK13 is heat stress-induced, and confers thermotolerance by degrading misfolded protein targets [117]. The heat-induced gene At5g53400 encoding the noncanonical small heat shock protein Bobber 1 (BOB1) required for thermotolerance and the duplicated gene *BOB2* (At4g27890) were induced by allyl-ITC at 1 h and 9 h [118]. *TIL1* (temperature-induced lipocalin; At5g58070), another gene required for thermotolerance [119], was induced at 1 h and 9 h. Four of the five heat-inducible *TAS1*-derived siRNA mediated target genes [120] were induced by allyl-ITC: *HTT1* (heat-induced TAS1-target 1; At4g29770), *HTT2* (At5g18040), *HTT3* (At5g18065) and *HTT4* (At1g51670). *HTT1* and *HTT2* are probably direct targets of HsfA1a and HsfA1b [120]. As mentioned above, *HsfA1a* and *HsfA1b* were not induced by allyl-ITC, but these Hsfs can on their turn positively regulate the expression of several other Hsfs (i.e. *HsfA1d*, *1e*, *2*, *3*, *4c*, *7a* and *HsfB2b*) [70], most of which were induced by allyl-ITC.

Based on our transcriptional profiling results, exposure to allyl-ITC seems to trigger a heat stress response in *A. thaliana*. ITCs do not only seem to lead to a heat stress response in plants as it has been shown that 4-methylsulphinylbutyl-ITC (sulforaphane) activates a heat shock response in animal cells [121]. Treatment with phenethyl-ITC led to an increased thermotolerance in *A. thaliana* [11], although the pathways through which this was mediated are not yet known. Based on a recently proposed model integrating H_2_O_2_, nitric oxide (NO) and calmodulin in the heat stress response [122], ITC-triggered H_2_O_2_ might signal NO formation that leads to calmodulin 3 (CaM3) activation, stimulating the DNA-binding activity of Hsfs and the accumulation of HSPs. It has indeed been shown that ITC treatment triggers the production of reactive oxygen species (ROS) and NO in plants [13, 14]. Our transcriptional data however does not give clear support for the ITC-induced heat stress response acting through that model. The key component *CaM3* was only slightly (below our selection criteria) induced by allyl-ITC at the two latest time points, although *CaM2* and other calmodulin like (*CML*) genes were induced (data not shown). The gene At1g37130 encoding the major nitrate reductase isoform NIA2 leading to NO production was not affected and *NIA1* (At1g77760) was slightly (below our selection criteria) induced by allyl-ITC at 30 min but downregulated at the later time points. Also *NOA1* (At3g47450; NO associated 1), which affects NO accumulation, was not affected by allyl-ITC. Nevertheless it would be interesting to test this and other possible signalling pathways. Hence we are currently investigating 1) the effect of allyl-ITC on *A. thaliana* mutants known to have reduced thermotolerance in order to identify the underlying mechanisms and 2) the thermotolerance of loss of function mutants in genes induced by allyl-ITC to identify potentially new actors regulating the plant response to heat stress.

### The extent of overlap in the transcriptional response to allyl-ITC and oxidative stress

Treatment with ITCs has been reported to lead to the generation of ROS and NO [13, 14, 16]. Heat stress also triggers the generation of ROS and, as mentioned above, ROS are implicated in the heat stress response [105, 122, 123]. Hsfs have been proposed as H_2_O_2_ sensors in plants and recently HsfA4a and Hsfa8 have been described as redox-sensitive transcription factors [76, 77]. Part of the heat stress response detected upon allyl-ITC treatment as described in the previous paragraph may therefore be due to the generation of ROS. We analysed our microarray data after allyl-ITC treatment in the search of a ROS response/oxidative burst response by comparing it to some previously reported transcriptional profiling studies where ROS-generating treatments were used.

**Fig. 9:**
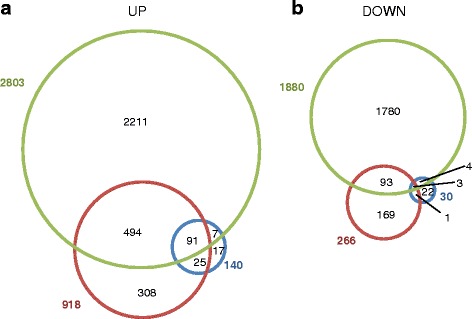
Overlap between responses to allyl-ITC and singlet oxygen or H_2_O_2_/superoxide. Venn diagrams showing the overlap between singlet oxygen (red) or H_2_O_2_/superoxide-affected (blue) genes [124] and allyl-ITC-affected (green). Genes in (**a**) were induced by log_2_ ≥ 1 while genes in (**b**) were repressed by log_2_ ≤ -1 at one time point at least

Of the 918 genes induced by at least log_2_ = 1 during the first two hours under singlet oxygen-producing conditions reported by op den Camp et al. [124], 63 % (585 genes) were also upregulated by allyl-ITC at one of the three time points (Fig. 9; Additional file 3A). Of the 266 singlet oxygen-downregulated genes, 96 genes (36 %) were also downregulated by allyl-ITC (Additional file 3B). When doing a similar comparison with the 140 genes induced by superoxide/H_2_O_2_ detected in the same study [124], 70 % (98 genes) were also induced by allyl-ITC at one of the three time points. Only 30 genes were downregulated by superoxide/H_2_O_2_ and only 7 of these (23 %) were also repressed by allyl-ITC. Ninety-one and three genes were respectively induced or repressed by all three treatments. Genes that were either induced or repressed by allyl-ITC only, were 2211 and 1780 (Fig. 9; Additional file 3).

Gadjev et al. [125] identified five transcripts considered to be hallmarks for the general oxidative stress response regardless of the type of ROS. At2g43510 (*ATTI1*) encoding a defensin-like protein was slightly downregulated at 1 h and 9 h of allyl-ITC treatment (Additional file 3C). At2g21640 and At1g05340, encoding proteins of unknown function, were induced after 9 h of allyl-ITC treatment. The two remaining hallmark genes, At1g57630 encoding a TIR-NB-LRR protein and the uncharacterized At1g19020, responded earlier and stronger to allyl-ITC (Additional file 3C).

Another 27 genes responding to a general oxidative stress were identified using a lower stringency [125]. Of these, 19 were induced by allyl-ITC and include heat stress associated genes (At3g08970/*DJB9*; At3g09350/*FesA1*), *GST*s and other stress associated genes (Additional file 3C).

Genes serving as hallmarks for specific oxidative stress conditions previously identified [125] were also used to compare to the allyl-ITC response. Of the 325 transcripts that are specifically singlet oxygen responsive (296 up- and 29 down-regulated), only 129 showed the same response to the allyl-ITC treatment (120 up- and 8 down-regulated) (Additional file 3D). The overlap between specifically superoxide-responsive genes and those affected by allyl-ITC was even smaller: of 106 and 32 genes respectively up- and down-regulated specifically by superoxide, only 8 and 2 genes showed the same response to allyl-ITC. The overlap in genes differentially regulated by the two conditions was actually bigger (data not shown). Of the 326 transcripts specific to the hydrogen peroxide response (189 up- and 137 down-regulated), 67 were equally affected by the allyl-ITC treatment (44 up- and 23 down-regulated) (Additional file 3D). The numbers of genes differentially regulated by the two conditions were almost as high: 35 genes upregulated by H_2_O_2_ but downregulated by allyl-ITC, 18 genes downregulated by H_2_O_2_ but upregulated by allyl-ITC (data not shown).

From these analyses, it seems clear to us that the transcriptional response to allyl-ITC cannot just be explained by ITC causing an oxidative burst. We identified a certain overlap between the allyl-ITC and oxidative stress responses, but this cannot be attributed to one particular ROS or oxidative stress condition. ROS production can occur at multiple locations in plant cells, such as chloroplasts, peroxisomes, mitochondria and on the outer surface of the plasma membrane. Such an extracellular burst of superoxide resulting from NADPH oxidase activity and the subsequent production of H_2_O_2_ are key features of the plant defence response [126, 127]. As GSL degradation products have well established roles in plant resistance, we analysed in more detail the allyl-ITC response in this respect in the next paragraph.

### Allyl-ITC and the perception of elicitors and effectors

Pattern recognition receptors (PRRs) at the plant cell surface can perceive the presence of pathogens by sensing pathogen/microbe-associated molecular patterns (PAMPs/MAMPs) and signals originating from the damaged plant (damage-associated molecular patterns; DAMPs). Recognition of PAMPs or DAMPs triggers a cascade of events, called PAMP-triggered immunity (PTI), which includes ion fluxes across the plasma membrane, generation of ROS, activation of mitogen-activated protein kinases (MAPKs) and transcriptional activation of genes. Successful pathogens can suppress PTI by excreting so-called effectors into the plant cell. When these effectors are perceived, typically by intracellular nucleotide-binding leucine-rich repeat (NB-LRR) proteins, the effector-triggered immunity (ETI) response, which is often accompanied by the so-called hypersensitive response characterized by rapid cell death, is triggered and leads to plant resistance [128].

PRRs can be divided into receptor-like kinases (RLKs), with an extracellular ligand-binding domain and an intracellular kinase domain, and receptor-like proteins (RLPs) that lack an intracellular kinase domain. The ectodomains can be of various types (e.g. leucine rich repeats (LRR), lysine motifs (LysM), lectin domain) and the *A. thaliana* genome contains several hundreds of genes encoding RLKs and RLPs that could putatively be involved in plant defence as PRRs, but only a few have been characterized so far [129].

**Fig. 10:**
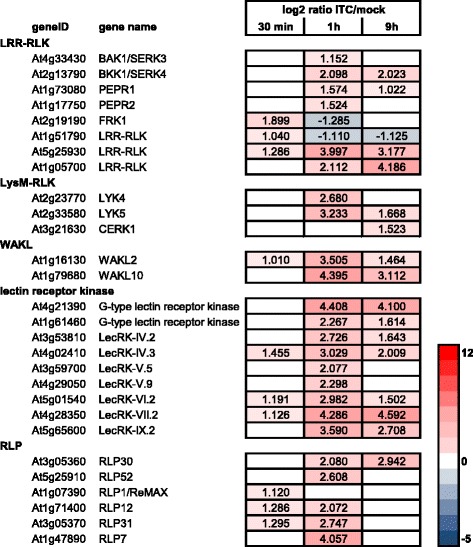
Effect of allyl-ITC on the expression of RLKs and RLPs. Changes in expression levels (log_2_ ratios) of selected RLKs and RLPs after 30 min, 1 h and 9 h of allyl-isothiocyanate treatment, compared to mock treatment. Only values for genes significantly affected at one time point at least are indicated. The colour scale goes from blue (downregulated by allyl-ITC) to red (upregulated by allyl-ITC) and the extremes are set to cover the range of the log_2_ ratios for the most affected genes in the whole dataset

RLKs and RLPs were not among the most highly responsive genes upon allyl-ITC exposure. However several interesting responses were observed (Fig. 10) that might point towards the role of allyl-ITC acting as DAMP or triggering the generation of DAMPs. The LRR-RLKs *BAK1/SERK3* (At4g33430) and *BKK1/SERK4* (At2g13790) that cooperate in PAMP and DAMP signalling [130] were induced by allyl-ITC (Fig. 10). Also *PEPR1* (At1g73080) and *PEPR2* (At1g17750) encoding LRR-RLKs that are the receptors of the elicitor active small AtPep peptides [131] were induced by allyl-ITC, in addition to the AtPep1 precursor gene *PROPEP1* (At5g64900) and two of its paralogs (*PROPEP3*/At5g64905 and *PROPEP5*/At5g09990) [132]. Several other LRR-RLK-encoding genes were induced by allyl-ITC treatment at the different time points, but the roles of these have not been described yet. Of particular interest for further studies might be *FRK1* (Flg22-induced receptor like kinase 1/At2g19190) and At1g51790 that were transiently induced at 30 min before being repressed at the later time points, or At5g25930 and At1g05700 which were among the most highly induced LRR-RLKs. The three genes encoding the LysM-RLKs LYK4 (At2g23770), LYK5 (At2g33580) and CERK1 (At3g21630), all involved in the recognition of the fungal cell wall PAMP chitin during plant innate immunity [133–135], were induced by allyl-ITC. WAKs (wall associated kinases) and WAKLs (WAK-like) are RLKs whose ectodomain contains epidermal growth factor-like repeats. WAK1 was identified as a receptor of oligogalacturonides (OGs), a DAMP generated from the plant cell wall polysaccharide homogalacturonan [136]. Nine of the 26 *WAK/WAKL* members were affected by allyl-ITC, in particular *WAKL2* (At1g16130) and *WAKL10* (At1g79680) (Fig. 10).

Lectin receptor kinases are another group of RLKs implicated in plant innate immunity [137]. Genes encoding the L-type lectin receptor kinases LecRK-IV.3 (At4g02410) and LecRK-VI.2 (At5g01540) were the *LecRK* genes most highly induced by allyl-ITC at 30 min and both LecRK are involved in pathogen resistance [138, 139]. The G-type At4g21390 and L-type At4g28350 (*LecRK-VII.2*) were the most highly induced *LecRKs* at 1 h and 9 h. Although the roles of most of the allyl-ITC induced lectin receptor kinases have not yet been revealed, many *LecRKs* have been shown to be particularly responsive to pathogens and PAMPs [140].

Receptor-like proteins (RLPs) have also been found to play a role in disease resistance [141]. Several RLP-encoding genes responded to allyl-ITC, the most highly induced being presented in Fig. 10. The LRR-RLPs members RLP30 (At3g05360) and RLP52 (At5g25910) are involved in resistance to fungal pathogens [142, 143]. ReMAX/RLP1 (At1g07390), with specificity for the bacterial proteinaceous MAMP eMax [144], was rapidly induced by allyl-ITC. Other, so far uncharacterized, *RLP*s showing an early response (i.e. At1g71400/*RLP12*; At3g05370/*RLP31*) and a strong response (i.e. At1g47890/*RLP7*) to allyl-ITC constitute interesting candidates for further studies.

NB-LRRs, acting in ETI that limits the proliferation of pathogens, constitute the major class of so-called R (resistance) proteins [145]. They are highly polymorphic and are classified based on the domains they contain [146, 147].

**Fig. 11:**
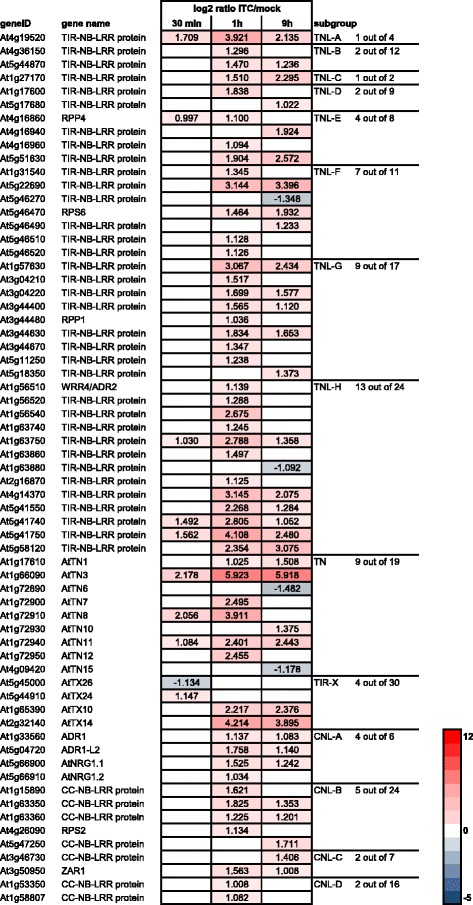
Effect of allyl-ITC on the expression of NB-LRRs. Changes in expression levels (log_2_ ratios) of NB-LRR-encoding genes after 30 min, 1 h and 9 h of allyl-ITC treatment, compared to mock treatment. The given NB-LRR subgroups are according to Meyers et al. [146]. Only values for genes significantly affected at one time point at least are indicated. The colour scale goes from blue (downregulated by allyl-ITC) to red (upregulated by allyl-ITC) and the extremes are set to cover the range of the log_2_ ratios for the most affected genes in the whole dataset

TIR-NB-LRR proteins contain an N-terminal Toll/Interleukin-1 Receptor homology region. Thirty-nine of the 94 TIR-NB-LRR-encoding genes identified by Meyers et al. [146] were affected by allyl-ITC treatment at one time point at least (Fig. 11; subgroups TNL-A to TNL-H). Two of these were downregulated at the 9 h time point: At1g63880 which was implicated in resistance to blackleg disease [148] and the uncharacterized At5g46270. Of the thirty-seven upregulated genes a large majority of 28 was induced highest at the 1 h time point while the remaining nine genes were most induced at the 9 h time point. Among these allyl-ITC responsive genes were some characterized resistance genes such as *RPP1* (At3g44480), *RPP4* (At4g16860), *RPS6* (At5g46470), *WRR4/ADR2* (At1g56510) [149–152], although they were not the most highly induced TIR-NB-LRR. The functions of most of the TIR-NB-LRR have however not been revealed yet. Hence, the most interesting TIR-NB-LRR-encoding genes in our dataset may be the still uncharacterized genes that were rapidly induced by allyl-ITC (i.e. At1g63750, At4g19520, At5g41740 and At5g41750) and those that were highly induced on at least two of the time points (i.e. At1g57630, At4g14370, At5g22690 and At5g58120) (Fig. 11).

Also eight genes classified as coding for TNs, TIR-NB proteins lacking a LRR domain [146], were affected by our allyl-ITC treatment. The genes At1g66090 (*AtTN3*) and At1g72940 (*AtTN11*) were induced at all three time points. *AtTN3* was the NB-LRR gene that showed the highest induction at each time point (i.e. log_2_ = 2.178 at 30 min; log_2_ = 5.923 at 1 h; log_2_ = 5.918 at 9 h) and was overall amongst the top-induced genes (Additional file 1). The specific functions of these two TNs have not been characterized yet but a role in plant defence has been postulated. They are induced by abiotic and biotic stresses, their transient expression leads to an EDS1 (enhanced disease susceptibility 1)-dependent HR cell death and they are able to interact with elicitors [147, 153]. They constitute therefore interesting candidates to investigate further for their role in the allyl-ITC response.

Of the 30 *TX* genes encoding proteins with a TIR domain but no LRR or NB domains [154], four were affected by the allyl-ITC treatment at any time point: At5g45000 (*AtTX26*) was downregulated at 30 min; At5g44910 (*AtTX24*) is upregulated at 30 min; while the two closely related At1g65390 (*AtTX10*) and At2g32140 (*AtTX14*) were upregulated at 1 h and 9 h. Although the specific functions of TX proteins are not yet known, their role in basal resistance has been recently investigated [153]. In particular, overexpression of At2g32140 (*AtTX14*) leads to activated expression of defence-related genes and an EDS1-dependent dwarf phenotype [155].

Most NB-LRR proteins that do not contain a TIR domain contain an N-terminal CC (coiled coil) domain. Of the 55 genes encoding CC-NB-LRR proteins [146], 13 genes were moderately upregulated by the allyl-ITC treatment at one time point at least (Fig. 11). These include the characterized resistance genes *ADR1* (At1g33560), *ADR1-L2* (At5g04720), *RPS2* (At4g26090) and *ZAR1* (At3g50950) [156–159].

Interestingly, some known *R* genes and plant defence-related genes were not affected by the allyl ITC treatment. For example the two *R* genes *RPM1* (At3g07040), encoding a CC-NBs-LRR, and *RPS4* (At5g5250), encoding a TIR-NB-LRR, signalling respectively through NDR1 (non-race specific disease resistance 1) and EDS1 were not significantly affected [162, 163]. Of these two major components in *R* gene-dependent defence activation *EDS1* (At3g48090) was induced at 1 h while *NDR1* (At3g20600) was not affected by the allyl-ITC treatment (Additional file 1). Some, but not all, NB-LRR–mediated ETI responses require accumulation of SA, which in turn controls transcriptional reprogramming through NPR1 (nonexpresser of PR genes 1; At1g64280). However *NPR1* was not induced in our dataset. Also the pathogen stress and SA signalling pathway markers *PR1* (pathogenesis-related protein 1; At2g14610) and *PR2* (At3g57260), and the plant defensins *PDF1.2a* (At5g44420) and *PDF1.2b* (At2g26020) were not affected by the allyl-ITC treatment.

GO-category analysis (Fig. 3) and the transcriptional changes of many defence-related genes (Additional file 1) such as the RLKs and RLPs described here, indicate that allyl-ITC triggers an immune response. The mechanisms remain unknown and the possibility that GSL-degradation products - allyl-ITC in the present case - might trigger this response by being perceived as DAMPs by a receptor, such as one of those described above, either directly or indirectly constitutes an interesting aspect worthy of further investigations. It could indeed be conceived that ITCs induce a receptor triggered response by changing the conformation of the receptor or that of a protein interacting with the receptor protein(s) in question (guard model; [160]). In animal systems ITCs have been shown to target proteins, triggering conformational changes and/or activation [164, 165]. Alternatively, receptor protein(s) could be activated by ITC-induced physiological changes by analogy to the mammalian NLRP3 receptor that is activated by various danger signals (e.g. PAMPs, DAMPs and environmental irritants) [161].

### Allyl-ITC and the triggering of cell death mechanisms

As mentioned above, programmed cell death (PCD) is a characteristic of the hypersensitive response (HR) during ETI. A recent study reported that the 4-methylsulphinylbutyl-ITC (sulforaphane) could induce PCD during HR [40]. The PCD during HR is characterized as “non-autolytic” and is often shown to be preceded by influx of calcium into the cytoplasm, activation of a MAPK signalling cascades, production of reactive oxygen intermediates and nitric oxide, and biosynthesis of SA [166]. PCD can also be mediated by the so-called ER stress which is due to the accumulation of un/misfolded proteins in the ER [167]. Our dataset revealed an overrepresentation of genes associated with cell death amongst those induced by allyl-ITC (Fig. 3), although the way gene expression was changed was not always in the sense of cell death promotion. Interestingly, it has recently been shown that the indole-GSL breakdown products indole-3-acetonitrile (IAN) and indole-3-carbinol (I3C), although not structurally related to ITCs, are able to attenuate PCD induced by the mycotoxin fumonisin B1 [168].

**Fig. 12:**
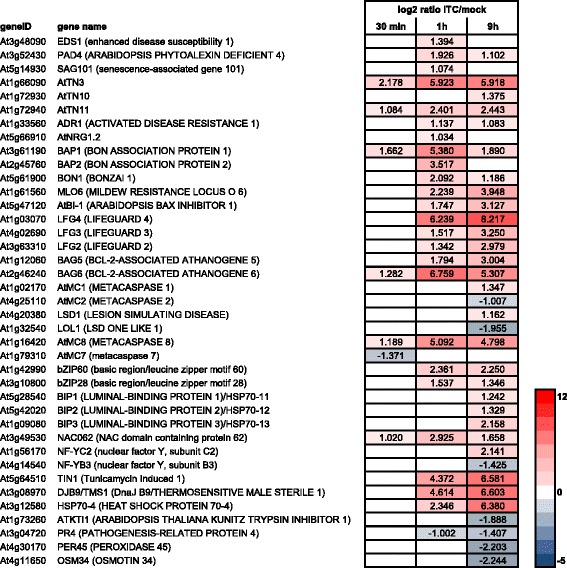
Cell death-related genes affected by allyl-ITC. Changes in expression levels (log_2_ ratios) of genes involved in cell death mechanisms after 30 min, 1 h and 9 h of allyl-ITC treatment, compared to mock treatment. Only values for genes significantly affected at one time point at least are indicated. The colour scale goes from blue (downregulated by allyl-ITC) to red (upregulated by allyl-ITC) and the extremes are set to cover the range of the log_2_ ratios for the most affected genes in the whole dataset. Genes are listed in the order they are described in the text

As described above, several TIR-NB-LRR-encoding genes were induced by the allyl-ITC treatment. Also three genes that form a signalling module integrating redox signals in a chain of events leading to PCD mediated by TIR-NB-LRR receptors [169], namely *EDS1* (At3g48090), *PAD4* (At3g52430) and *SAG101* (At5g14930), were induced at 1 h (Fig. 12). It has also been shown that EDS1-dependent cell death can be induced when some, but not all, TN proteins, TX proteins or the TIR domain of TIR-NB-LRR proteins are transiently expressed [153, 170]. Of the five TN/TXs showing this effect [153], three had increased gene expression after allyl-ITC treatment: At1g66090 (*AtTN3*), At1g72930 (*AtTN10*) and At1g72940 (*AtTN11*) (Figs. 11 and 12).

Among CC-NB-LRR-encoding genes that were induced by allyl-ITC (Fig. 11), *ADR1* (At1g33560) and *AtNRG1.2* (At5g66910), have been shown to induce HR as part of a defence response when their atypical CC-domains (called CC_R_) were transiently expressed [171]. It was speculated that they might sense indirect cellular insults or act downstream of canonical NB-LRR proteins in disease resistance [171]. Such a role as “helper NB-LRRs” was verified for three ADR1 family members, and the triple mutant exhibited compromised HR [156].

The three general repressors of cell death *BAP1* (At3g61190), *BAP2* (At2g45760) and their interaction partner *BON1* (At5g61900) are induced by the allyl-ITC treatment (Fig. 12). It has previously been shown that *BAP* transcripts are induced by a number of biotic and abiotic stimuli and that overexpression of *BAP1* and *BON1* delays HR induced by two avirulent strains of the bacterium *Pseudomonas syringae* and cell death induced by the ROS-generating herbicide paraquat [172].

Also *MLO6* (At1g61560), which belongs to the *MILDEW RESISTANCE LOCUS O* family of negative regulators of cell death [173], was induced at 1 h and 9 h.

Overexpression of plant cell death suppressor *BI-1* (bax inhibitor 1) proteins has shown to suppress cell death induced by a variety of factors: mammalian Bax (BCL2-associated X protein), pathogen attack, abiotic stresses, chemically-induced oxidative stresses [174]. *AtBI-1* (At5g47120; bax inhibitor 1) [175–177] and the three closely related genes encoding LFG4 (At1g03070), LFG3 (At4g02690) and LFG2 (At3g63310), belonging to the bax inhibitor-1 family and possibly inhibiting cell death [178], were all highly induced at 1 h and 9 h. Homologues of the mammalian pro- and anti-apoptotic proteins Bax and Bcl-2, respectively, have not yet been identified in plants. However, seven homologues of mammalian Bcl-2–associated athanogene (BAG) proteins, cytoprotective proteins acting as chaperone regulators that interact with HSP70 and HSC70 proteins, have been described for *A. thaliana* [179]. Allyl-ITC treatment for 1 h and 9 h induces the expression of genes encoding BAG5 (At1g12060) and BAG6 (At2g46240). *BAG 6* was among the top 10 allyl-ITC induced genes at the 1 h time point (Additional file 1). Both proteins contain in addition to the BAG domain (BD) a calmodulin-binding motif, a special feature of some plant BAG proteins [110]. BAG6 was characterized as a stress (SA, H_2_O_2_, heat) responsive protein that is able to bind calmodulins (in a Ca^2+^-independent manner) but not AtHSC70-1 in vitro and that, unexpectedly, induces PCD [111]. Another report however postulated that BAG6 has a cytoprotective role, promotes basal resistance to the necrotrophic fungus *Botrytis cinerea* and retards plant senescence [110]. Except for the finding that BAG5 (At1g12060) was able to bind AtHSC70-1 in vitro [111], little is known about the role of this BAG family member in plants.

Other actors with potential cell death regulatory function are metacaspases (MCs), and *A. thaliana* possesses nine MC-encoding genes [180]. The expression of *AtMC1* (At1g02170) and *AtMC2* (At4g25110), coding for two metacaspases that have antagonistic functions in regulating HR-associated cell death [181], responded differently to the allyl-ITC treatment. While the positive regulator of cell death AtMC1 was induced, the negative regulator of cell death AtMC2 was repressed at the last time point tested. In addition, the gene encoding LSD1 (lesion simulating disease 1; At4g20380), which is a negative cell death regulator and interacts with AtMC1 [181, 182], was upregulated at the 9 h time point. On the other hand, LOL1 (LSD one like 1; At1g32540), with homology to LSD1 and considered a positive regulator of cell death [183], was downregulated. Another metacaspase, the positive regulator of oxidative stress-induced cell death AtMC8 (At1g16420; [184]) was induced at all three time points, particularly at 1 h and 9 h. The yet uncharacterized *AtMC7* (At1g79310) was downregulated at the 30 min time point. Interestingly, AtMC4 (At1g79340), a positive regulator that contributes to cell death activation during oxidative stress and host–pathogen defence responses [185] was not significantly affected by allyl-ITC. It should be noted that post-translational control mechanisms and protein-protein interactions have been described to affect metacaspases [186] and our transcriptional profiling may hence not give the whole picture of how allyl-ITC affects metacaspases during its putative induction of cell death.

Several adverse biotic and abiotic environmental factors can lead to endoplasmic reticulum (ER) stress as the capacity of the protein folding and ER-associated degradation (ERAD) machinery is exceeded. This triggers a stress mitigation mechanism called unfolded protein response (UPR). ER stress sensors located on the ER membrane initiate the UPR signalling pathway that can ultimately lead to PCD. Two branches of the UPR signalling pathway have been identified in plants so far: the IRE and the bZIP28/bZIP17 (homologous to IRE and ATF6 in animals, respectively) [187].

Plant IRE1s (encoded by two genes in *A. thaliana*) are localized to the ER membrane, possess kinase and endoribonuclease domains, and in response to ER stress IRE1 splices the mRNA encoding bZIP60 [188]. Although neither *IRE1-1/IRE1b* (At5g24360) nor *IRE1-2/IRE1a* (At2g17520) were affected by allyl-ITC, *bZIP60* (At1g42990) was induced at 1 h and 9 h (Fig. 12). The spliced bZIP60 mRNA is translated, the protein translocates to the nucleus and activates directly UPR genes such as genes encoding chaperones of the HSP70 family called BIPs (luminal-binding proteins). *BIP1* (At5g28540), *BIP2* (At5g42020) and *BIP3* (At1g09080) were induced at 9 h allyl-ITC treatment (Fig. 12). bZIP60 also activates the transcription factors NAC062 (At3g49530), induced by allyl-ITC, and NAC103 (At5g64060) which then activate UPR genes such as *CNX1* (calnexin 1; At5g61790), *CRT1* (calreticulin 1; At5g56340) and *PDI5* (protein disulfide isomerase 5; At1g21750) [189, 190]. The other pathway is mediated by bZIP28 and bZIP17 that are also localized to the ER membrane under normal conditions [191]. While *bZIP28* (At3g10800) was induced after 1 h and 9 h of allyl-ITC exposure, *bZIP17* (At2g40950) was not affected. Upon ER stress, they are transported to the Golgi where they are proteolytically cleaved and then translocate to the nucleus, where they induce UPR genes. bZIP28 forms a transcriptional complex with a NF-YA4/NF-YB3/NF-YC2 (nuclear factor Y) trimer, leading to the induction of genes like *BIP3*, *SDF2* (stromal cell derived 2), *CNX1*, *PDI5* and *NF-YC2* [191]. *NF-YC2* (At1g56170) was upregulated at 9 h; *NF-YB3* (At4g14540) was downregulated at 9 h while the expression of *NF-YA4* (At2g34720) was not affected by the allyl-ITC treatment. Also other markers for ER stress and UPR were not induced such as *CNX1* (At5g61790), *AtCRT1* (At5g56340), *SDF2* (At2g25110), *NAC089* (At5g22290) transcription factor [192] or any of the six *PDI* genes induced by chemical ER stress inducers [193].

However genes reported to be induced by tunicamycin, a typical ER stress agent that elicits UPR, were also induced by allyl-ITC, such as *TIN1* (tunicamycin induced 1; At5g64510), *DJB9/TMS1* (DnaJ B9/thermosensitive male sterile 1; At3g08970), *HSP70-4* (At3g12580) and the already mentioned *AtBI-1* (At5g47120) [177, 188, 194–197] (Fig. 12). The allyl-ITC treatment also lead to the downregulation of genes (mostly after 9 h) that were reported to be downregulated by tunicamycin, such as the cell death antagonist *KTI1* (kunitz trypsin inhibitor 1; At1g73260) [198], *PR4* (At3g04720), *PER45* (peroxidase 45; At4g30170) and *OSM34* (osmotin 34; At4g11650) (Fig. 12).

Based on these expression profiles, it is possible that ITCs trigger an ER stress and an UPR response, leading ultimately to PCD. As different environmental conditions and chemical compounds can lead to ER stress, it is however difficult to pinpoint at how ITC might trigger ER stress. As discussed previously, ITCs might either directly affect the folding of proteins or might through the depletion of GSH affect the oxidizing status of the ER and hence the disulphide bond formation involved in protein folding. ITC might also lead to ER stress by triggering an oxidative stress. Hence, the action of allyl-ITC through this pathway is worth being further investigated, e.g. by using chemical chaperones in conjunction with allyl-ITC to reduce the load of misfolded proteins in the ER. Also, as it was shown that sulforaphane and benzyl-ITC, but not isopropyl-ITC, led to cell death [40], a larger range of ITCs should be tested. Cell death triggered by sulforaphane was observable 24 h after infiltration [40]. Most of the ER stress and UPR genes that responded to our allyl-ITC treatment were affected at the latest (9 h) time point that we assessed. It would therefore be interesting to see if other hallmarks of this pathway can be detected after a longer exposure to allyl-ITC.

Due to the spatial separation of GSLs and myrosinases in the intact plant cell [199] endogenous ITC generated upon cell rupture and exogenously applied ITC might be expected to act to a great degree in the apoplast and cytosol. Plants possess a mechanism to sense misfolded proteins in the cytosol, the so-called cytosolic protein response (CPR). Upon stress-triggered accumulation of misfolded proteins in the cytosol, HSP70/HSP90 chaperones are recruited. This disturbs the interaction between HSPs and Hsfs that is responsible for maintaining protein homeostasis. The released Hsfs trimerize to form active transcription factor complexes that get imported into the nucleus and activate the transcription of HSPs. Upon restoration of normal levels of free chaperones, Hsfs are inactivated by binding of the HSP70 machinery [64, 74]. Hence, the CPR is a subcomponent of the wider heat shock response already discussed earlier in this article. When comparing transcriptional features attributed to CPR [74] with the transcriptional response to allyl-ITC, a large overlap was detected within the induced response: of the 148 upregulated genes of the CPR, 111 genes were induced by allyl-ITC after 1 h and 132 genes were induced by allyl-ITC after 9 h (Additional file 4A and B). Many *HSPs* and *Hsfs*, including *HsfA2* that was identified as one of the regulatory components of CPR in *A. thaliana* [74], were induced in the CPR and by allyl-ITC. The list contains also many genes encoding other transcription factors and genes encoding proteins involved in protein degradation. The overlap in downregulated genes between the two conditions is much more restricted: of the 89 CPR-repressed genes, 19 and 30 were downregulated by allyl-ITC at 1 h and 9 h respectively (Additional file 4C and D). Interestingly however, eight of these 19 genes are known to be induced by auxin, and include genes such as *IAA1/AXR5* (At4g14560), *ACS4* (ACC synthase 4; At2g22810); the homeobox-leucine zipper *HAT2* (At5g47370) and three *SAUR* (small auxin-up RNAs) genes [200–204]. It might be that allyl-ITC exerts an auxin-antagonistic action, as has recently been shown for indole-3-carbinol [205], a degradation product of indole-3-methyl-GSL. Also other signalling molecules such as oligogalacturonides (OGs) have been reported to inhibit the induction of certain auxin responsive genes [206]. Another possible explanation might be that the allyl-ITC-triggered production of H_2_O_2_ suppresses the activation of auxin-inducible genes [207, 208]. In this context it is noteworthy that one of the few *SAUR* genes induced by allyl-ITC, *SAUR35* (At4g12410), was also induced during the CPR [74].

Characteristic features of the cytosolic protein response at the transcriptional level are hence part of the larger allyl-ITC response. The mechanism(s) through which allyl-ITC triggers this response and its outcome seem therefore to be interesting aspects deserving further investigations.

## Conclusion

Exposure of *Arabidopsis thaliana* to vapours of allyl-ITC triggered a rapid and substantial transcriptional response affecting numerous biological processes. For the purpose of this paper a few affected key processes were selected for a more detailed description of the genes involved: glucosinolate metabolism, sulphate uptake and assimilation, heat stress response, oxidative stress response, elicitor perception, plant defence and cell death mechanisms. These were chosen so as to relate transcriptional changes to the biosynthetic steps related to the generation of ITCs, to identify gene regulations that might be involved in the observed effects of ITC on plants reported in the literature and to present some avenues for further investigations in order to decipher the molecular mechanisms underlying the effects caused by ITCs in plants.

## Methods

### Plant material and growth conditions

Seeds of the *A. thaliana* accession Col-0 were surface sterilised and sown on Petri dishes (9 cm diameter) containing solid in vitro cultivation medium consisting of ½ x Murashige and Skoog basal salt mixture (Sigma-Aldrich, Saint Louis, USA), 2 % sucrose, 0.6 % phytoagar (w/v), pH 5.7. Seeds were stratified for 2 days at 4 °C before being transferred to a controlled growth chamber under a 16 h photoperiod (light intensity: 75 μmol.m^-2^.sec^-1^) at 21-23 °C.

### Exposure to allyl-isothiocyanate

Allyl-isothiocyanate (Sigma-Aldrich, Saint Louis, USA; Cat Nb 377430) was freshly diluted in commercial rape seed oil to a concentration of 0.05 M and 200 μl of this solution was applied to a piece of filter paper that was placed into a 14-cm diameter Petri dish. Exposure to allyl-isothiocyanate was obtained by putting a 9-cm dish (lid removed) containing ten-day old *A. thaliana* plants into this 14-cm diameter dish for 30 min, 1 h or 9 h. The plants were hence exposed to vapours of allyl-isothiocyanate in a closed atmosphere. The control consisted of filter paper onto which 200 μl of rape seed oil was applied.

### Microarray analysis

For microarray experiments, *A. thaliana* plants from the isothiocyanate and the control treatment were processed simultaneously at each time point through the following procedure. Shoots (including rosette leaves and hypocotyl) of the in vitro grown plantlets were harvested separately from two individual Petri dishes and immediately flash-frozen in liquid N_2_. The harvested tissue was stored at -80 °C until further processing. Frozen plant tissue was submitted to two disruption cycles with a TissueLyser II (Qiagen, Hilden, Germany) for 2 min at 25 Hz, using 2 ml tubes containing a 5 mm stainless steel bead. The TissueLyser adaptors used for the first disruption cycles, tubes and beads were prefrozen at -80 °C. Total RNA was extracted with the Spectrum Plant Total RNA kit (Sigma-Aldrich, Saint Louis, USA) as described by the supplier, but with lysis solution being added to the plant tissue between the two disruption cycles. An on-column DNase digestion was performed using the RNase-Free DNase Set (Qiagen, Hilden, Germany) to eliminate genomic DNA. RNasin (Promega, Madison, USA) was added to the RNA to a final concentration of 1 U/μl. Total RNA was quantified with a NanoDrop ND-1000 (Nanodrop, Delaware, USA) and RNA quality was verified by formaldehyde gel electrophoresis. Total RNA (200 ng) isolated as described above was reverse transcribed, amplified and labelled using the Low Input Quick Amp Labeling Kit, One-Color (Agilent Technologies, CatNb 5190–2305). 1650 ng cRNA from each sample was fragmented and hybridized on 4 × 44 K Arabidopsis (V4) Gene Expression Microarray (Agilent Technologies, CatNb G2519F-021169) in an Agilent G2545A Hybridization rotary oven (10 rpm, 65 °C, 17.5 h). Hybridization was performed with the Gene Expression Hybridization Kit (Agilent Technologies, CatNb 5188–5242). The slides were washed with buffer 1 & 2 from Gene Expression Wash Buffer kit (Agilent Technologies, CatNb 5188–5327) and scanned twice at 5 μm resolution on a laser scanner (Agilent Technologies G2505 B), using the “dynamic range expander” option in the scanner software. The resulting images were processed using Agilent Feature Extraction software v9.5.

### Statistical analysis of microarray data

The microarray data were preprocessed using the Limma package (version 3.2.3) as implemented in R [209]. Spots identified as feature outliers were excluded from analysis, and weak or non-detected spots were given reduced weight (0.5). The data were normalized using quantile normalization and no background subtraction was performed. The Benjamini and Hochberg's method was used to estimate the false discovery rate [210]. Values are an average of all probes mapping to the gene in question. Genes with an adjusted *p*-value below 0.05 were considered to be statistically significant differentially expressed but only genes whose expression is affected by log_2_ ≥ 1 or ≤ -1 are discussed in the text. The study is MIAME-compliant and raw microarray data files have been deposited in the Gene Expression Omnibus (GEO accession number: GSE81634).

### Representation of microarray data

Comparison between gene expression profiles within our dataset and between our dataset and publicly available datasets were done using the BioVenn web application [211]. Analysis of gene ontology categories overrepresented in the transcriptional response to allyl-ITC was performed using the Cytoscape plugin BiNGO using a hypergeometric test with a Benjamini & Hochberg False Discovery Rate (FDR) correction and a significance level of 0.05 [212].

## Additional files

Additional file 1: List of genes whose expression is significantly affected (*P* < 0.05 and log_2_ ≥ 1 or ≤ -1) by the allyl-ITC treatment at one of the three time points. (PDF 1744 kb) Additional file 2: Comparison of allyl-ITC-induced genes and MBF1C-dependent genes. (XLSX 19 kb) Additional file 3: Comparison of the allyl-ITC response with oxidative stress responses. (XLSX 230 kb) Additional file 4: Comparison of the transcriptional response to allyl-ITC with the CPR response. (XLSX 85 kb)
