# Supplementary material for: Allyl-isothiocyanate treatment induces a complex transcriptional reprogramming including heat stress, oxidative stress and plant defence responses in Arabidopsis thaliana
Source: BMC Genomics. 2016 Sep 17;17:740. doi: 10.1186/s12864-016-3039-x (PMC5027104; doi:10.1186/s12864-016-3039-x)
Supplement: Additional file 1: — List of genes whose expression is significantly affected (P < 0.05 and log2 ≥ 1 or ≤ -1) by the allyl-ITC treatment at one of the three time points. (PDF 1744 kb) [file 12864_2016_3039_MOESM1_ESM.pdf]

List of genes significantly affected (P<0.05 and log2≥1 or ≤-1) by allyl-ITC after 30min

Locus ID	gene description (TAIR)	log2 ITC/mock 30min	adj.P.Val
At1g01720	ANAC002 (Arabidopsis NAC domain containing protein 2); Belongs to a large family of putative trans	1.23	5.01E-05
At1g02450	NIMIN1 (NIM1-interacting 1); NIMIN1 modulates PR gene expression according the following model	-1.435	0.01559
At1g04000	unknown protein	1.057	0.00059
At1g04770	Tetratricopeptide repeat (TPR)-like superfamily protein	3.01	1.05E-08
At1g05135	expressed protein, isoform contains a non-consensus TG acceptor site at intron.; pseudogene of unknov	1.048	0.0003
At1g05575	unknown protein	1.586	1.94E-05
At1g07135	glycine-rich protein	2.958	0.00022
At1g07160	Protein phosphatase 2C family protein	2.203	3.92E-05
At1g07390	AtRLP1 (receptor like protein 1)	1.12	0.00024
At1g07520	GRAS family transcription factor	1.042	0.0006
At1g09350	AtGolS3 (galactinol synthase 3)	1.376	0.00038
At1g09500	NAD(P)-binding Rossmann-fold superfamily protein; similar to Eucalyptus gunnii alcohol dehydrogen	-1.17	0.0033
At1g11050	Protein kinase superfamily protein	1.432	3.44E-05
At1g11185	other RNA; Unknown gene	-1.352	0.01263
At1g12030	Protein of unknown function (DUF506)	3.126	0.0001
At1g12940	ATNRT2.5 (nitrate transporter2.5); member of High affinity nitrate transporter family	1.313	0.02873
At1g13080	CYP71B2 (cytochrome P450, family 71, subfamily B, polypeptide 2); cytochrome P450 monooxygena	-1.048	8.33E-05
At1g13210	ACA.l (autoinhibited Ca2+/ATPase II)	1.02	0.00098
At1g13650	BEST Arabidopsis thaliana protein match is: 18S pre-ribosomal assembly protein gar2-related (TAIR:/	-2.054	0.0004
At1g14200	RING/U-box superfamily protein	2.159	2.66E-06
At1g14370	APK2A (protein kinase 2A); Encodes protein kinase APK2a.	1.053	0.0004
At1g16130	WAKL2 (wall associated kinase-like 2); wall-associated kinase like	1.01	0.00299
At1g16420	ATMC8 (ARABIDOPSIS THALIANA METACASPASE 8); Encodes a metacaspase (cysteine-type en	1.189	0.04924
At1g18570	AtMYB51 (myb domain protein 51); Encodes a member of the R2R3-MYB transcription family. Invol	1.262	0.00056
At1g18710	AtMYB47 (myb domain protein 47); Member of the R2R3 factor gene family.	-1.499	0.00093
At1g18990	Protein of unknown function, DUF593	-1.096	0.00042
At1g19020	unknown protein	1.763	0.00026
At1g19180	JAZ1 (jasmonate-zim-domain protein 1); JAZ1 is a nuclear-localized protein involved in jasmonate sig	1.104	0.0061
At1g19320	Pathogenesis-related thaumatin superfamily protein	1.157	0.00123
At1g20310	unknown protein	1.26	0.0023
At1g20470	SAUR-like auxin-responsive protein family	-1.294	0.0003
At1g20823	RING/U-box superfamily protein	1.433	0.00232
At1g21240	WAK3 (wall associated kinase 3); encodes a wall-associated kinase	-1.321	0.04292
At1g21310	ATEXT3 (extensin 3); Encodes extensin 3.	1.441	0.0025
At1g21550	Calcium-binding EF-hand family protein	1.314	0.00086
At1g21890	nodulin MtN21 /EamA-like transporter family protein	-1.386	0.00055
At1g22770	GI (GIGANTEA); Together with CONSTANTS (CO) and FLOWERING LOCUS T (FT), GIGANTEA	1.014	7.56E-05
At1g22810	Integrase-type DNA-binding superfamily protein; encodes a member of the DREB subfamily A-5 of EI	3.898	1.05E-08
At1g23390	Kelch repeat-containing F-box family protein	-1.248	0.0004
At1g23840	unknown protein	-1.099	0.00012
At1g24140	Matrixin family protein	2.285	0.0007
At1g26390	FAD-binding Berberine family protein	-2.155	0.0061
At1g27020	unknown protein	1.376	0.0046
At1g27730	STZ (salt tolerance zinc finger); Related to Cys2/His2-type zinc-finger proteins found in higher plants.	4.103	5.86E-08
At1g27820	Polynucleotidyl transferase, ribonuclease H-like superfamily protein	1.483	4.75E-05
At1g27890	Polynucleotidyl transferase, ribonuclease H-like superfamily protein	1.666	1.52E-05
At1g30135	JAZ8 (jasmonate-zim-domain protein 8)	-1.535	0.00375
At1g30220	ATINT2 (ARABIDOPSIS THALIANA INOSITOL TRANSPORTER 2); Inositol transporter presentin	-1.143	0.00081
At1g30320	Remorin family protein	-1.004	9.32E-06
At1g30370	alpha/beta-Hydrolases superfamily protein	2.901	0.00161
At1g30650	ATWRKY14 (WRKY DNA-BINDING PROTEIN 14); member of WRKY Transcription Factor; Grou	1.116	0.00473
At1g30900	BP80-3.3 (binding protein of 80 kDa 3.3)	-1.157	0.00109
At1g31040	PLATZ transcription factor family protein	-1.096	0.00055
At1g32780	GroES-like zinc-binding dehydrogenase family protein	-1.021	0.00104
At1g33760	Integrase-type DNA-binding superfamily protein; encodes a member of the DREB subfamily A-4 of EI	2.552	0.00047
At1g33950	Avirulence induced gene (AIG1) family protein	-2.109	0.03777
At1g35210	unknown protein	3.062	1.10E-06
At1g36370	SHM7 (serine hydroxymethyltransferase 7); Encodes a putative serine hydroxymethyltransferase.	1.653	3.37E-05
At1g43000	PLATZ transcription factor family protein	1.027	0.00042
At1g45145	ATH5 (THIOREDOXIN H-TYPE 5); encodes a cytosolic thioredoxin that reduces disulfide bridges of	-1.141	0.02523
At1g49530	GGPS6 (geranylgeranyl pyrophosphate synthase 6); encodes a mitochondria-targeted geranylgeranyl py	-1.222	0.00018
At1g49640	alpha/beta-Hydrolases superfamily protein	1.271	0.01155
At1g49980	DNA/RNA polymerases superfamily protein	-1.02	0.00056
At1g50040	Protein of unknown function (DUF1005)	-1.175	0.00639
At1g51140	basic helix-loop-helix (bHLH) DNA-binding superfamily protein	-1.087	1.80E-05

At1g51790	Leucine-rich repeat protein kinase family protein	1.04	0.01234
At1g52250	NA	-1.136	0.00228
At1g52342	unknown protein	-1.542	0.00392
At1g53100	Core-2/I-branching beta-1,6-N-acetylglucosaminyltransferase family protein	-1.236	0.00874
At1g53885	Protein of unknown function (DUF581)	-2.452	0.00021
At1g55920	ATSERAT2.1 (serine acetyltransferase 2.1); Encodes a chloroplast/cytosol localized serine O-acetyltra	1.278	3.22E-06
At1g56240	AtPP2-B13 (phloem protein 2-B13)	1.596	0.0131
At1g56300	Chaperone DnaJ-domain superfamily protein	1.451	3.18E-05
At1g56650	ATMYB75 (MYB DOMAIN PROTEIN 75); Encodes a putative MYB domain containing transcriptio	-1.445	0.00638
At1g58225	unknown protein	-1.267	0.0017
At1g59860	HSP20-like chaperones superfamily protein	1.924	2.44E-05
At1g59870	ABCG36 (ATP-BINDING CASSETTE G36); ATP binding cassette transporter. Localized to the plasn	1.206	9.22E-05
At1g61120	GES (GERANYLLINALOOL SYNTHASE); Encodes a geranyllinalool synthase that produces a precu	-1.352	0.03132
At1g61290	SYP124 (syntaxin of plants 124); member of SYP12 Gene Family	1.174	0.00083
At1g61380	SD1-29 (S-domain-1 29)	1.144	4.81E-05
At1g61470	Polynucleotidyl transferase, ribonuclease H-like superfamily protein	1.598	1.38E-05
At1g61610	S-locus lectin protein kinase family protein	-1.415	0.00038
At1g62180	APR2 (5'adenylylphosphosulfate reductase 2); encodes a adenosine 5'-phosphosulfate reductase, involv	1.304	1.79E-05
At1g63750	Disease resistance protein (TIR-NBS-LRR class) family	1.03	0.0008
At1g63857	NA	1.035	0.00033
At1g64170	ATCHX16 (cation/H+ exchanger 16); member of Putative Na+/H+ antiporter family	1.436	0.00047
At1g65890	AAE12 (acyl activating enzyme 12)	-1.118	0.00232
At1g66090	Disease resistance protein (TIR-NBS class)	2.178	0.00443
At1g66230	AtMYB20 (myb domain protein 20); Encodes a putative transcription factor (MYB20).	-1.192	5.11E-05
At1g66370	AtMYB113 (myb domain protein 113); Encodes a member of the MYB family of transcription factors.	-2.235	0.00249
At1g66860	Class I glutamine amidotransferase-like superfamily protein	-2.61	4.34E-05
At1g66870	Carbohydrate-binding X8 domain superfamily protein	-1.332	0.01752
At1g68190	B-box zinc finger family protein	-1.246	4.02E-05
At1g68870	ATSOFL2 (SOB FIVE-LIKE 2); AtSOFL1 acts redundantly with AtSOFL2 as positive regulator of cyt	-1.294	3.22E-05
At1g70290	TPS8 (trehalose-6-phosphatase synthase S8); Encodes an enzyme putatively involved in trehalose bios	-1.181	0.00088
At1g70700	JAZ9 (JASMONATE-ZIM-DOMAIN PROTEIN 9); JAZ9 is a protein presumed to be involved in jasn	-1.269	1.14E-05
At1g71400	AtRLP12 (receptor like protein 12); Encodes a CLAVATA2 (CLV2)-related gene. Complements the cl	1.286	8.91E-05
At1g71520	Integrase-type DNA-binding superfamily protein; encodes a member of the DREB subfamily A-5 of EI	1.939	3.37E-05
At1g72910	Toll-Interleukin-Resistance (TIR) domain-containing protein	2.056	0.01274
At1g72920	Toll-Interleukin-Resistance (TIR) domain family protein	2.155	0.0005
At1g72940	Toll-Interleukin-Resistance (TIR) domain-containing protein	1.084	0.00143
At1g73500	MKK9 (MAP kinase kinase 9); member of MAP Kinase Kinase family. Autophosphorylates and also p	1.168	5.16E-05
At1g75770	unknown protein	-1.151	1.31E-05
At1g76110	HMG (high mobility group) box protein with ARID/BRIGHT DNA-binding domain	-1.537	9.25E-05
At1g76600	unknown protein	1.537	0.00011
At1g76640	Calcium-binding EF-hand family protein	-1.995	0.0069
At1g76680	ATOPR1 (ARABIDOPSIS 12-OXOPHYTODIENOATE REDUCTASE 1); Encodes a member of an a	1.578	0.0006
At1g76930	ATEXT1 (EXTENSIN 1); Encodes an Arabidopsis extensin gene that belongs to cell-wall hydroxyprol	1.374	0.0033
At1g77120	ADH (ALCOHOL DEHYDROGENASE); Catalyzes the reduction of acetaldehyde using NADH as rec	1.288	0.0276
At1g77450	anac032 (NAC domain containing protein 32)	1.011	0.00079
At1g77640	Integrase-type DNA-binding superfamily protein; encodes a member of the DREB subfamily A-5 of EI	1.143	0.00093
At1g78410	VQ motif-containing protein	1.598	0.0018
At1g79310	AtMC7 (metacaspase 7)	-1.371	0.00012
At1g79410	5-Oct (organic cation/carnitine transporter5)	1.322	6.48E-06
At1g80840	WRKY40 (WRKY DNA-binding protein 40); Pathogen-induced transcription factor. Binds W-box seq	3.049	0.0004
At2g01170	BAT1 (bidirectional amino acid transporter 1); Encodes a bidirectional amino acid transporter that can	1.007	3.26E-05
At2g01180	ATPAP1 (phosphatidic acid phosphatase 1); Encodes phosphatidate phosphatase. Up-regulated by gen	1.052	0.0014
At2g01890	ATPAP8 (PURPLE ACID PHOSPHATASE 8); Encodes a purple acid phosphatase (PAP) belonging to	-1.548	7.19E-05
At2g02810	ATUTR1 (UDP-GALACTOSE TRANSPORTER 1); Encodes a multitransmembrane hydrophobic pro	-1.175	6.48E-06
At2g02950	PKS1 (phytochrome kinase substrate 1); Encodes a basic soluble protein which can independently bind	-1.107	0.0002
At2g03230	GCK domain-containing protein	1.321	0.00152
At2g04450	ATNUDT6 (nudix hydrolase homolog 6); Encodes a protein with NADH pyrophosphatase activity. Alt	-1.544	0.00797
At2g13810	ALD1 (AGD2-like defense response protein 1)	-1.78	0.03855
At2g14610	ATPR1 (PATHOGENESIS-RELATED GENE 1); PR1 gene expression is induced in response to a var	-2.733	0.00908
At2g15390	FUT4 (fucosyltransferase 4); Predicted fucosyltransferase, based on similarity to FUT1, but not functic	1.072	0.00122
At2g15480	UGT73B5 (UDP-glucosyl transferase 73B5)	1.091	0.0003
At2g15490	UGT73B4 (UDP-glycosyltransferase 73B4)	1.116	0.0017
At2g17740	Cysteine/Histidine-rich C1 domain family protein	1.496	0.01656
At2g18210	unknown protein	1.141	0.0026
At2g18560	UDP-Glycosyltransferase superfamily protein	-1.055	0.00076
At2g18700	ATTPS11 (trehalose phosphatase/synthase 11); Encodes an enzyme putatively involved in trehalose bi	-1.375	0.0004
At2g19190	FRK1 (FLG22-induced receptor-like kinase 1); Receptor-like protein kinase. Involved in early defense	1.899	0.00541

At2g20142	Toll-Interleukin-Resistance (TIR) domain family protein	1.23	0.0309
At2g20670	Protein of unknown function (DUF506)	-1.254	0.00385
At2g21660	ATGRP7 (GLYCINE RICH PROTEIN 7); Encodes a small glycine-rich RNA binding protein that is p	1.248	0.0029
At2g22496	MIR779A (microRNA779A); Encodes a microRNA of unknown function. MicroRNAs are regulatory	1.97	2.56E-05
At2g22770	NAI1 (basic helix-loop-helix (bHLH) DNA-binding superfamily protein); regulates the development of	-2.103	2.70E-06
At2g22880	VQ motif-containing protein	2.045	0.0002
At2g25200	Plant protein of unknown function (DUF868)	-1.122	0.00059
At2g25735	unknown protein	1.141	0.00048
At2g26150	ATHSFA2 (heat shock transcription factor A2); member of Heat Stress Transcription Factor (Hsf) fam	2.274	2.44E-05
At2g26530	AR781 (Protein of unknown function (DUF1645)); unknown function	1.861	1.34E-05
At2g26560	PLA IIA (PHOSPHOLIPASE A 2A); Encodes a lipid acyl hydrolase with wide substrate specificity th	1.372	0.0285
At2g28056	MIR172 (MICRORNA 172); Encodes a microRNA that targets several genes containing AP2 domains	1.037	3.90E-05
At2g28305	LOG1 (LONELY GUY 1)	-1.262	0.0004
At2g29300	NAD(P)-binding Rossmann-fold superfamily protein	-1.374	0.00046
At2g29310	NAD(P)-binding Rossmann-fold superfamily protein	-1.004	8.26E-05
At2g29350	SAG13 (senescence-associated gene 13); senescence-associated gene SAG13 encoding a short-chain a	-1.775	0.0218
At2g29440	ATGSTU6 (glutathione S-transferase tau 6); Encodes glutathione transferase belonging to the tau class	-1.353	7.14E-06
At2g29450	ATGSTU1 (ARABIDOPSIS THALIANA GLUTATHIONE S-TRANSFERASE TAU 1); Encodes a m	-1.275	0.00011
At2g29720	CTF2B (FAD/NAD(P)-binding oxidoreductase family protein); Encodes CTF2B.	1.159	6.48E-06
At2g30550	alpha/beta-Hydrolases superfamily protein; Encodes a lipase that hydrolyzes phosphatidylcholine, glyc	-1.042	5.25E-05
At2g31180	ATMYB14 (ARABIDOPSIS THALIANA MYB DOMAIN PROTEIN 14); Member of the R2R3 facto	-1.255	0.00135
At2g31945	unknown protein	1.522	0.00047
At2g32030	Acyl-CoA N-acyltransferases (NAT) superfamily protein	2.588	8.33E-07
At2g32150	Haloacid dehalogenase-like hydrolase (HAD) superfamily protein	-1.419	0.0037
At2g32200	unknown protein	1.185	0.00945
At2g34600	JAZ7 (jasmonate-zim-domain protein 7)	-2.481	0.00069
At2g34940	BP80-3.2 (binding protein of 80 kDa 3.2)	-1.052	0.00581
At2g35658	unknown protein	1.28	0.0007
At2g35710	Nucleotide-diphospho-sugar transferases superfamily protein	1.158	0.0014
At2g35930	PUB23 (plant U-box 23); Encodes a cytoplasmically localized U-box domain containing E3 ubiquitin l	1.296	6.26E-06
At2g36650	unknown protein	-1.007	0.00604
At2g36690	2-oxoglutarate (2OG) and Fe(II)-dependent oxygenase superfamily protein	1.095	0.01719
At2g37430	C2H2 and C2HC zinc fingers superfamily protein	3.183	3.75E-07
At2g37870	Bifunctional inhibitor/lipid-transfer protein/seed storage 2S albumin superfamily protein	-1.219	0.0053
At2g37900	Major facilitator superfamily protein	-1.103	0.00287
At2g38470	ATWRKY33 (WRKY DNA-BINDING PROTEIN 33); Member of the plant WRKY transcription facto	2.732	2.90E-06
At2g38600	HAD superfamily, subfamily IIIB acid phosphatase	-1.623	0.0001
At2g38820	Protein of unknown function (DUF506)	-1.03	5.22E-05
At2g39200	ATMLO12 (MILDEW RESISTANCE LOCUS O 12); A member of a large family of seven-transmemb	1.05	0.0409
At2g39380	ATEXO70H2 (exocyst subunit exo70 family protein H2); A member of EXO70 gene family, putative e	1.472	0.00554
At2g39518	Uncharacterised protein family (UPF0497)	1.32	0.0073
At2g39650	Protein of unknown function (DUF506)	2.17	1.14E-05
At2g40095	Alpha/beta hydrolase related protein	1.184	0.00014
At2g40140	SZF2 ((SALT-INDUCIBLE ZINC FINGER 2)	1.912	4.51E-05
At2g41010	ATCAMBP25 (calmodulin (CAM)-binding protein of 25 kDa); Encodes a novel calmodulin binding pr	1.34	7.92E-05
At2g41230	unknown protein	1.59	0.00143
At2g41250	Haloacid dehalogenase-like hydrolase (HAD) superfamily protein	-1.001	0.0011
At2g41510	ATCKX1 (CYTOKININ OXIDASE/DEHYDROGENASE 1); It encodes a protein whose sequence is s	1.213	0.0019
At2g41640	Glycosyltransferase family 61 protein	1.054	0.00814
At2g42900	Plant basic secretory protein (BSP) family protein	-1.469	3.90E-05
At2g43570	CHI (chitinase, putative)	-1.69	0.02436
At2g43620	Chitinase family protein	1.694	0.00629
At2g44080	ARL (ARGOS-like); Encodes ARL, a gene similar to ARGOS involved in cell expansion-dependent or	1.37	2.14E-05
At2g44130	Galactose oxidase/kelch repeat superfamily protein	-1.04	0.00037
At2g44230	Plant protein of unknown function (DUF946)	-1.653	1.59E-05
At2g44240	Protein of Unknown Function (DUF239)	-3.535	0.00301
At2g44578	RING/U-box superfamily protein	-1.099	0.00032
At2g44840	ATERF13 (ETHYLENE-RESPONSIVE ELEMENT BINDING FACTOR 13); encodes a member of th	2.111	0.0024
At2g45350	CRR4 (CHLORORESPIRATORY REDUCTION 4); Encodes a member of a PCMP (plant combinator	-1.03	1.78E-05
At2g46240	ATBAG6 (ARABIDOPSIS THALIANA BCL-2-ASSOCIATED ATHANOGENE 6); A member of Ara	1.282	0.0005
At2g46510	AIB (ABA-inducible BHLH-type transcription factor); Encodes a nuclear localized BLH domain conta	-1.059	0.00013
At2g46970	PIL1 (phytochrome interacting factor 3-like 1); encodes a novel Myc-related bHLH transcription factor	1.121	0.0006
At3g02840	ARM repeat superfamily protein	1.87	0.00024
At3g04717	pseudogene, similar to pathogen- and wound-inducible antifungal protein CBP20 precursor, similar to	-1.514	0.00326
At3g05370	AtRLP31 (receptor like protein 31)	1.295	0.00032
At3g05936	unknown protein	-1.552	6.34E-05
At3g09520	ATEXO70H4 (exocyst subunit exo70 family protein H4); A member of EXO70 gene family, putative e	1.086	0.00047

At3g09870	SAUR-like auxin-responsive protein family	2.539	4.15E-05
At3g10930	unknown protein	1.554	4.83E-07
At3g11820	ATSYR1 (SYNTAXIN RELATED PROTEIN 1); Encodes a syntaxin localized at the plasma membrar	1.025	0.0005
At3g11840	PUB24 (plant U-box 24); Encodes a U-box-domain-containing E3 ubiquitin ligase that acts as a negati	1.04	0.0016
At3g12890	ASML2 (activator of spomin::LUC2); Encodes a protein belonging to a class of CCT (CONSTANS, C	1.07	0.0069
At3g14200	Chaperone DnaJ-domain superfamily protein	1.565	2.90E-06
At3g14760	unknown protein	-1.029	0.00292
At3g15270	SPL5 (squamosa promoter binding protein-like 5); Encodes a member of the SPL (squamosa-promoter	-1.101	0.00222
At3g15450	Aluminium induced protein with YGL and LRDR motifs	-1.658	8.60E-05
At3g15518	unknown protein	1.497	8.91E-05
At3g15536	Unknown gene; Unknown gene	-2.64	0.00665
At3g15770	unknown protein	-1.02	1.20E-05
At3g16120	Dynein light chain type 1 family protein	-1.233	0.00033
At3g16720	ATL2 (TOXICOS EN LEVADURA 2); RING-H2 protein induced after exposure to chitin or inactivate	1.987	2.42E-05
At3g19200	unknown protein	-1.183	0.00084
At3g21150	BBX32 (B-box 32); Encodes a protein with a B-box domain predicted to act as a transcription factor. I	1.333	0.01484
At3g21870	CYCP2.1 (cyclin p2.1)	-1.22	0.0028
At3g22120	CWLP (cell wall-plasma membrane linker protein); cell wall-plasma membrane linker protein homolog	1.107	0.008
At3g22121	other RNA; Potential natural antisense gene, locus overlaps with AT3G22120	1.309	0.00417
At3g22142	Bifunctional inhibitor/lipid-transfer protein/seed storage 2S albumin superfamily protein; Encodes a Pr	1.189	0.0011
At3g22275	unknown protein	-2.751	6.61E-06
At3g22886	MIR167A (microRNA167A); Encodes a microRNA that targets ARF family members ARF6 and ARF	1.428	3.22E-05
At3g23230	Integrase-type DNA-binding superfamily protein; encodes a member of the ERF (ethylene response fac	3.023	1.72E-05
At3g23240	ATERF1 (ETHYLENE RESPONSE FACTOR 1); encodes a member of the ERF (ethylene response fa	1.579	0.00043
At3g23250	ATMYB15 (MYB DOMAIN PROTEIN 15); Member of the R2R3 factor gene family.	1.501	0.0002
At3g23880	F-box and associated interaction domains-containing protein	-1.485	0.00024
At3g25010	AtRLP41 (receptor like protein 41)	-1.625	0.03261
At3g25180	CYP82G1 (cytochrome P450, family 82, subfamily G, polypeptide 1); member of CYP82G	-1.878	0.00843
At3g25250	OXI1 (oxidative signal-inducible1); Arabidopsis protein kinase	2.405	2.90E-06
At3g25600	Calcium-binding EF-hand family protein	1.115	0.00041
At3g25882	NIMIN-2 (NIM1-interacting 2); encodes a kinase that physically interacts with NPR1/NIM1	-1.427	0.00276
At3g28270	Protein of unknown function (DUF677)	-1.413	0.0014
At3g28340	GATL10 (galacturonosyltransferase-like 10); Encodes a protein with putative galacturonosyltransferas	1.545	0.0004
At3g28510	P-loop containing nucleoside triphosphate hydrolases superfamily protein	-2.64	0.009
At3g28540	P-loop containing nucleoside triphosphate hydrolases superfamily protein	-1.14	0.0477
At3g29000	Calcium-binding EF-hand family protein	2.679	3.07E-07
At3g29240	Protein of unknown function (DUF179)	-1.005	0.0008
At3g29370	unknown protein	-1.265	0.0021
At3g44350	anac061 (NAC domain containing protein 61)	-1.382	0.00474
At3g45640	ATMPK3 (mitogen-activated protein kinase 3); Encodes a mitogen-activated kinase whose mRNA lev	1.225	0.00116
At3g46300	unknown protein	-1.484	0.00142
At3g46490	2-oxoglutarate (2OG) and Fe(II)-dependent oxygenase superfamily protein	-1.005	0.00446
At3g46620	zinc finger (C3HC4-type RING finger) family protein	1.548	1.09E-05
At3g48080	alpha/beta-Hydrolases superfamily protein	-1.288	0.03102
At3g48360	BT2 (BTB and TAZ domain protein 2); encodes a protein (BT2) that is an essential component of the	-2.059	9.05E-05
At3g48520	CYP94B3 (cytochrome P450, family 94, subfamily B, polypeptide 3); member of CYP94B	-1.031	0.01702
At3g49530	ANAC062 (NAC domain containing protein 62); Transcription factor that serves as a molecular link b	1.02	4.82E-05
At3g49570	LSU3 (RESPONSE TO LOW SULFUR 3)	4.706	3.97E-08
At3g49580	LSU1 (RESPONSE TO LOW SULFUR 1)	2.955	1.36E-05
At3g49790	Carbohydrate-binding protein	-1.234	5.38E-05
At3g50060	MYB77 (myb domain protein 77); Encodes a member of the R2R3 transcription factor gene family. Ex	1.654	0.00049
At3g50280	HXXXD-type acyl-transferase family protein	-1.902	0.00017
At3g50560	NAD(P)-binding Rossmann-fold superfamily protein	-1.186	0.00131
At3g50930	BCS1 (cytochrome BC1 synthesis)	1.121	0.0081
At3g51180	Zinc finger C-x8-C-x5-C-x3-H type family protein	1.143	0.00033
At3g52400	SYP122 (syntaxin of plants 122); syntaxin protein, involved in the negative regulation of defense pathy	1.285	0.0005
At3g53232	DVL20 (DEVIL 20)	-1.141	0.00109
At3g54880	unknown protein	-1.156	0.00032
At3g55150	ATEXO70H1 (exocyst subunit exo70 family protein H1); A member of EXO70 gene family, putative e	1.047	0.00308
At3g55630	ATDFD (DHFS-FPGS homolog D)	-1.1	4.94E-05
At3g55840	Hs1pro-1 protein	1.023	0.0034
At3g56275	expressed protein; pseudogene of unknown protein	-1.343	0.0074
At3g56710	SIB1 (sigma factor binding protein 1); Sig1 binding protein; interacts with Sig1R4. As well as Sig1, Si	1.52	0.00021
At3g56880	VQ motif-containing protein	1.176	0.0002
At3g57530	CPK32 (calcium-dependent protein kinase 32); Calcium-dependent Protein Kinase. ABA signaling cor	1.104	0.00013
At3g59900	ARGOS (AUXIN-REGULATED GENE INVOLVED IN ORGAN SIZE); Encodes ARGOS (Auxin-Re	1.404	7.79E-06
At3g61060	AtPP2-A13 (phloem protein 2-A13)	-1.025	0.0031

At3g61190	BAP1 (BON association protein 1); Encodes a protein with a C2 domain that binds to BON1 in yeast t	1.662	0.0059
At3g61198	other RNA; Unknown gene	-1.08	1.91E-05
At3g62090	PIF6 (PHYTOCHROME-INTERACTING FACTOR 6); encodes a novel Myc-related bHLH transcript	1.089	4.94E-05
At3g62950	Thioredoxin superfamily protein	-1.01	0.03274
At4g00700	C2 calcium/lipid-binding plant phosphoribosyltransferase family protein	-1.122	0.03516
At4g01026	PYL7 (PYR1-like 7); Encodes a member of the PYR (pyrabactin resistance)/PYL(PYR1-like)/RCAR	-1.112	5.02E-05
At4g01460	basic helix-loop-helix (bHLH) DNA-binding superfamily protein	-1.155	8.45E-05
At4g01680	AtMYB55 (myb domain protein 55); Encodes a putative transcription factor (MYB55).	-1.085	0.00138
At4g01870	tolB protein-related	1.037	0.0047
At4g02410	Concanavalin A-like lectin protein kinase family protein	1.455	4.12E-05
At4g03400	DFL2 (DWARF IN LIGHT 2); Encodes a GH3-related gene involved in red light-specific hypocotyl el	1.013	3.26E-05
At4g04490	CRK36 (cysteine-rich RLK (RECEPTOR-like protein kinase) 36); Encodes a cysteine-rich receptor-lik	-1.232	0.03545
At4g04500	CRK37 (cysteine-rich RLK (RECEPTOR-like protein kinase) 37); Encodes a cysteine-rich receptor-lik	-2.1	0.00633
At4g04610	APR1 (APS reductase 1); Encodes a protein disulfide isomerase-like (PDIL) protein, a member of a mu	1.744	1.29E-05
At4g08555	unknown protein	1.056	0.01739
At4g08910	unknown protein	-1.185	0.0022
At4g10500	2-oxoglutarate (2OG) and Fe(II)-dependent oxygenase superfamily protein	-2.496	0.02758
At4g10910	unknown protein	-1.543	0.00047
At4g11655	Uncharacterised protein family (UPF0497)	-1.492	0.01683
At4g12470	AZI1 (azelaic acid induced 1); Encodes AZI1 (AZELAIC ACID INDUCED 1). Involved in the primin	2.024	0.00857
At4g12480	pEARLI 1 (Bifunctional inhibitor/lipid-transfer protein/seed storage 2S albumin superfamily protein);	2.432	0.01651
At4g12500	Bifunctional inhibitor/lipid-transfer protein/seed storage 2S albumin superfamily protein	2.425	0.02181
At4g12970	STOMAGEN (STOMAGEN); Encodes a cysteine-rich peptide, a secretory factor that is produced in th	-1.14	0.00047
At4g14390	Ankyrin repeat family protein	-1.5	0.00099
At4g14450	unknown protein	2.274	4.42E-06
At4g15248	B-box type zinc finger family protein	1.273	0.02128
At4g15550	IAGLU (indole-3-acetate beta-D-glucosyltransferase); UDP-glucose:indole-3-acetate beta-D-glucosyltr	1.51	5.65E-06
At4g15630	Uncharacterised protein family (UPF0497)	-1.184	0.00017
At4g15975	RING/U-box superfamily protein	1.627	5.28E-05
At4g17030	ATEXLB1 (expansin-like B1); Encodes EXLB1 (expansin-like B1), a member of the expansin family.	-1.054	0.002
At4g17490	ATERF6 (ethylene responsive element binding factor 6); Encodes a member of the ERF (ethylene resp	1.585	0.0003
At4g18200	NA	1.146	0.0008
At4g19520	disease resistance protein (TIR-NBS-LRR class) family	1.709	0.0004
At4g20000	VQ motif-containing protein	1.044	0.02058
At4g21990	APR3 (APS reductase 3); Encodes a protein disulfide isomerase-like (PDIL) protein, a member of a mu	2.687	1.66E-07
At4g22470	protease inhibitor/seed storage/lipid transfer protein (LTP) family protein	2.236	0.00308
At4g22485	Bifunctional inhibitor/lipid-transfer protein/seed storage 2S albumin superfamily protein; Encodes a Pr	1.09	0.01953
At4g22505	Bifunctional inhibitor/lipid-transfer protein/seed storage 2S albumin superfamily protein	1.066	0.01274
At4g23600	CORI3 (CORONATINE INDUCED 1); Encodes cystine lyase which is expected to be involved in ami	1.042	0.0363
At4g23810	WRKY53 (WRKY family transcription factor); member of WRKY Transcription Factor; Group III	1.531	0.0001
At4g24160	alpha/beta-Hydrolases superfamily protein; Encodes a soluble lysophosphatidic acid acyltransferase wi	1.251	0.0005
At4g24380	INVOLVED IN: 10-formyltetrahydrofolate biosynthetic process, folic acid and derivative biosynthetic	1.273	0.00069
At4g24570	DIC2 (dicarboxylate carrier 2); Encodes one of the mitochondrial dicarboxylate carriers (DIC): DIC1 (	3.146	1.31E-05
At4g25000	AMY1 (alpha-amylase-like); Predicted to be secreted protein based on signalP prediction. Involved in	-1.826	0.00176
At4g25480	CBF3 (C-REPEAT BINDING FACTOR 3); encodes a member of the DREB subfamily A-1 of ERF/A	1.007	0.0002
At4g25750	ABC-2 type transporter family protein	1.14	0.00441
At4g25780	CAP (Cysteine-rich secretory proteins, Antigen 5, and Pathogenesis-related 1 protein) superfamily pro	-1.005	0.00107
At4g26200	ACS7 (1-amino-cyclopropane-1-carboxylate synthase 7); Member of a family of proteins in Arabidops	1.589	0.00974
At4g27280	Calcium-binding EF-hand family protein	2.118	0.00048
At4g27410	ANAC072 (Arabidopsis NAC domain containing protein 72); Encodes a NAC transcription factor indu	-1.064	0.00566
At4g27652	unknown protein	1.133	6.54E-05
At4g27657	unknown protein	1.598	0.00034
At4g28350	Concanavalin A-like lectin protein kinase family protein	1.126	0.0002
At4g29520	LOCATED IN: endoplasmic reticulum, plasma membrane	-1.035	0.00012
At4g29610	Cytidine/deoxycytidylate deaminase family protein	1.57	0.00013
At4g29780	unknown protein	2.489	0.00012
At4g30180	sequence-specific DNA binding transcription factors	-1.498	0.00112
At4g31550	WRKY11 (WRKY DNA-binding protein 11); member of WRKY Transcription Factor; Group II-d; ne	1.081	0.0002
At4g33070	Thiamine pyrophosphate dependent pyruvate decarboxylase family protein	1.695	0.04413
At4g33550	Bifunctional inhibitor/lipid-transfer protein/seed storage 2S albumin superfamily protein	-1.24	0.00345
At4g33666	unknown protein	-1.032	0.03245
At4g34131	UGT73B3 (UDP-glucosyl transferase 73B3)	1.489	0.00021
At4g34135	UGT73B2 (UDP-glucosyltransferase 73B2); The At4g34135 gene encodes a flavonol 7-O-glucosyltran	1.352	0.00127
At4g34950	Major facilitator superfamily protein	1.376	0.00013
At4g36850	PQ-loop repeat family protein / transmembrane family protein	-1.182	0.00349
At4g36950	MAPKKK21 (mitogen-activated protein kinase kinase kinase 21); member of MEKK subfamily	-1.276	0.01922
At4g37150	ATMES9 (ARABIDOPSIS THALIANA METHYL ESTERASE 9); Encodes a protein shown to have c	-1.494	4.39E-05

At4g37290	unknown protein	1.847	0.00051
At5g01180	ATPTR5 (ARABIDOPSIS THALIANA PEPTIDE TRANSPORTER 5); Encodes a dipeptide transport	1.174	0.00011
At5g01540	LECRKA4.1 (lectin receptor kinase a4.1); Encodes LecRKA4.1, a member of the lectin receptor kinas	1.191	0.00148
At5g02190	ATASP38 (ARABIDOPSIS THALIANA ASPARTIC PROTEASE 38); encodes an aspartic protease, I	1.391	1.31E-05
At5g04340	CZF2 (COLD INDUCED ZINC FINGER PROTEIN 2); putative c2h2 zinc finger transcription factor r	3.533	3.97E-08
At5g04370	NAMT1 (S-adenosyl-L-methionine-dependent methyltransferases superfamily protein); A member of t	-1.336	6.23E-05
At5g05250	unknown protein	1.093	0.0054
At5g05290	ATEXPA2 (expansin A2); Encodes an expansin. Naming convention from the Expansin Working Grou	1.253	0.00406
At5g05410	DREB2 (DEHYDRATION-RESPONSIVE ELEMENT BINDING PROTEIN 2); Encodes a transcriptic	1.799	8.73E-05
At5g06865	other RNA; Potential natural antisense gene, locus overlaps with AT5G06860	-1.074	0.00172
At5g08790	anac081 (Arabidopsis NAC domain containing protein 81); induced by wounding, belongs to a large fa	1.873	5.71E-06
At5g09290	Inositol monophosphatase family protein	-1.407	0.0147
At5g10150	Domain of unknown function (DUF966)	-1.144	0.0008
At5g11670	ATNADP-ME2 (Arabidopsis thaliana NADP-malic enzyme 2); The malic enzyme (EC 1.1.1.40) encoc	1.056	0.00021
At5g12110	Glutathione S-transferase, C-terminal-like	-1.029	0.00021
At5g13220	JAS1 (JASMONATE-ASSOCIATED 1); Plants overexpressing At5g13220.3, but not At5g13220.1 sh	-1.948	0.0006
At5g14730	unknown protein	1.294	1.14E-05
At5g15310	ATMYB16 (myb domain protein 16); Member of the R2R3 factor gene family.	-1.301	4.43E-05
At5g15950	Adenosylmethionine decarboxylase family protein	1.441	3.84E-05
At5g16960	Zinc-binding dehydrogenase family protein	1.208	4.41E-05
At5g17350	unknown protein	1.426	0.0004
At5g17490	RGL3 (RGA-like protein 3); DELLA subfamily member involved in GA signal transduction	-1.096	0.00013
At5g19190	unknown protein	-1.413	2.34E-05
At5g20670	Protein of unknown function (DUF1677)	-1.083	0.0005
At5g21940	unknown protein	-1.385	2.60E-05
At5g22545	unknown protein	-1.215	0.02022
At5g22570	ATWRKY38 (ARABIDOPSIS THALIANA WRKY DNA-BINDING PROTEIN 38); member of WRK	-2.357	0.0034
At5g22920	CHY-type/CTCHY-type/RING-type Zinc finger protein	-1.478	2.72E-05
At5g23425	NA	-1.225	0.01489
At5g24110	ATWRKY30 (ARABIDOPSIS THALIANA WRKY DNA-BINDING PROTEIN 30); member of WRK	1.288	0.0009
At5g24150	SQE5 (SQUALENE MONOOXYGENASE 5); squalene monooxygenase gene homolog	-1.173	0.00301
At5g24155	FAD/NAD(P)-binding oxidoreductase family protein	-1.236	0.00068
At5g24470	APRR5 (pseudo-response regulator 5); Encodes a pseudo-response regulator whose mutation affects va	1.031	0.00603
At5g24655	LSU4 (RESPONSE TO LOW SULFUR 4)	1.164	0.00273
At5g24660	LSU2 (RESPONSE TO LOW SULFUR 2)	3.357	3.97E-08
At5g25130	CYP71B12 (cytochrome P450, family 71, subfamily B, polypeptide 12); putative cytochrome P450	-1.265	0.00183
At5g25930	Protein kinase family protein with leucine-rich repeat domain	1.286	0.00011
At5g26220	ChaC-like family protein	3.225	1.19E-07
At5g26690	Heavy metal transport/detoxification superfamily protein	-2.186	0.01655
At5g27420	CNI1 (carbon/nitrogen insensitive 1); Encodes CNI1 (Carbon/Nitrogen Insensitive1) (also named as A	2.786	2.90E-06
At5g28237	Pyridoxal-5'-phosphate-dependent enzyme family protein	-1.74	0.0002
At5g28490	LSH1 (LIGHT-DEPENDENT SHORT HYPOCOTYLS 1); Encodes a nuclear protein that mediates lig	1.091	0.00239
At5g35735	Auxin-responsive family protein	1.599	0.00039
At5g39760	AtHB23 (homeobox protein 23)	1.02	0.0002
At5g41740	Disease resistance protein (TIR-NBS-LRR class) family	1.492	0.0005
At5g41750	Disease resistance protein (TIR-NBS-LRR class) family	1.562	0.0041
At5g42380	CML37 (calmodulin like 37)	2.364	0.0046
At5g42830	HXXXD-type acyl-transferase family protein	-1.232	0.00102
At5g44060	unknown protein	1.663	4.18E-05
At5g44380	FAD-binding Berberine family protein	1.13	0.005
At5g44680	DNA glycosylase superfamily protein	-1.197	0.00054
At5g44910	Toll-Interleukin-Resistance (TIR) domain family protein	1.147	0.00779
At5g45000	Disease resistance protein (TIR-NBS-LRR class) family	-1.134	0.01495
At5g45340	CYP707A3 (cytochrome P450, family 707, subfamily A, polypeptide 3); Encodes a protein with ABA	2.344	2.67E-05
At5g46295	unknown protein	2.576	2.90E-06
At5g46780	VQ motif-containing protein	1.059	0.0004
At5g47230	ATERF-5 (ETHYLENE RESPONSIVE ELEMENT BINDING FACTOR- 5); encodes a member of the	3.01	1.29E-05
At5g47960	ATRABA4C (RAB GTPase homolog A4C); Encodes a small molecular weight g-protein.	1.295	0.00074
At5g48430	Eukaryotic aspartyl protease family protein	1.177	0.01667
At5g48490	Bifunctional inhibitor/lipid-transfer protein/seed storage 2S albumin superfamily protein	-1.276	0.00041
At5g48540	receptor-like protein kinase-related family protein	1.623	0.00021
At5g48850	ATSDI1 (SULPHUR DEFICIENCY-INDUCED 1); homologous to the wheat sulphate deficiency-indu	3.632	1.31E-05
At5g49360	ATBXL1 (BETA-XYLOSIDASE 1); Encodes a bifunctional {beta}-D-xylosidase/{alpha}-L-arabinofu	-1.539	0.0049
At5g49480	ATCP1 (Ca2+-binding protein 1); AtCP1 encodes a novel Ca2+-binding protein, which shares sequen	1.032	0.00084
At5g50915	basic helix-loop-helix (bHLH) DNA-binding superfamily protein	-1.351	0.0008
At5g51190	Integrase-type DNA-binding superfamily protein; encodes a member of the ERF (ethylene response fac	2.603	0.0004
At5g52120	AtPP2-A14 (phloem protein 2-A14)	-1.045	0.00012

At5g52390	PAR1 protein	-1.379	0.02084
At5g52415	pseudogene, hypothetical protein	1.024	0.00492
At5g52860	ABC-2 type transporter family protein	1.256	0.00188
At5g54165	unknown protein	2.365	1.72E-05
At5g54470	B-box type zinc finger family protein	1.048	0.00372
At5g54490	PBP1 (pinoid-binding protein 1); Encodes a PINOID (PID)-binding protein containing putative EF-har	2.525	2.90E-06
At5g54585	unknown protein	-1.264	0.00075
At5g54610	ANK (ankyrin); Induced in response to Salicylic acid. Belongs to the ankyrin repeat protein family.	-2.172	0.00927
At5g54720	Ankyrin repeat family protein	2.182	5.38E-05
At5g56100	glycine-rich protein / oleosin	-1.507	0.00027
At5g56550	ATOXS3 (OXIDATIVE STRESS 3); Encodes OXIDATIVE STRESS 3 (OXS3)， involved in	-1.512	6.48E-06
At5g56880	unknown protein	-1.08	0.0017
At5g57785	unknown protein	-1.213	0.00024
At5g58770	Undecaprenyl pyrophosphate synthetase family protein	-1.487	0.00031
At5g59220	HAI1 (highly ABA-induced PP2C gene 1)	-1.235	9.25E-05
At5g59580	UGT76E1 (UDP-glucosyl transferase 76E1)	-1.298	0.00267
At5g59820	RHL41 (RESPONSIVE TO HIGH LIGHT 41); Encodes a zinc finger protein involved in high light and	1.933	0.00122
At5g60900	RLK1 (receptor-like protein kinase 1); Encodes a receptor-like protein kinase.	-1.166	0.03562
At5g61160	AACT1 (anthocyanin 5-aromatic acyltransferase 1)	1.641	0.00126
At5g61440	ACHT5 (atypical CYS HIS rich thioredoxin 5); Encodes a member of the thioredoxin family protein.	-1.249	0.0127
At5g61600	ERF104 (ethylene response factor 104); encodes a member of the ERF (ethylene response factor) subfa	2.608	2.90E-06
At5g63130	Octicosapeptide/Phox/Bem1p family protein	1.677	3.34E-05
At5g63135	unknown protein	-2.589	0.0465
At5g63450	CYP94B1 (cytochrome P450, family 94, subfamily B, polypeptide 1); member of CYP94B	-1.687	0.00109
At5g63790	ANAC102 (NAC domain containing protein 102); Encodes a member of the NAC family of transcripti	2.013	5.06E-08
At5g64120	Peroxidase superfamily protein; encodes a cell wall bound peroxidase that is induced by hypo-osmolar	1.465	0.04121
At5g64190	unknown protein	-1.205	0.0039
At5g65207	unknown protein	-1.214	0.00037
At5g66650	Protein of unknown function (DUF607)	1.07	0.0178
At5g67060	HEC1 (HECATE 1)	1.079	0.00385
At5g67190	DEAR2 (DREB and EAR motif protein 2); encodes a member of the DREB subfamily A-5 of ERF/AP.	-1.2	3.90E-05

List of genes significantly affected (P<0.05 and log2≥1 or ≤-1) by allyl-ITC after 1h

Locus ID	gene description (TAIR)	log2 ITC/mock 1h	adj.P.Val
At1g01340	ATCNGC10 (cyclic nucleotide gated channel 10); member of Cyclic nucleotide gated channel family	1.287	6.67E-05
At1g01420	UGT72B3 (UDP-glucosyl transferase 72B3)	-1.328	7.00E-07
At1g01480	ACS2 (1-amino-cyclopropane-1-carboxylate synthase 2); a member of the 1-aminocyclopropane-1-carbo	1.061	0.02915
At1g01560	ATMPK11 (MAP kinase 11); member of MAP Kinase	3.246	1.04E-05
At1g01720	ANAC002 (Arabidopsis NAC domain containing protein 2); Belongs to a large family of putative transc	1.703	2.43E-07
At1g01940	Cyclophilin-like peptidyl-prolyl cis-trans isomerase family protein	1.065	0.00078
At1g02090	ATCSN7 (ARABIDOPSIS THALIANA COP9 SIGNALOSOME SUBUNIT 7); encodes a phosphoprot	1.487	1.01E-05
At1g02230	ANAC004 (NAC domain containing protein 4)	1.643	0.00083
At1g02360	Chitinase family protein	1.226	1.12E-05
At1g02400	ATGA2OX4 (Arabidopsis thaliana gibberellin 2-oxidase 4); Encodes a gibberellin 2-oxidase that acts o	3.562	5.54E-10
At1g02450	NIMIN1 (NIM1-interacting 1); NIMIN1 modulates PR gene expression according the following model:	1.26	0.02078
At1g02700	unknown protein	2.182	1.96E-09
At1g03070	Bax inhibitor-1 family protein	6.239	7.97E-12
At1g03220	Eukaryotic aspartyl protease family protein	2.386	8.17E-09
At1g03230	Eukaryotic aspartyl protease family protein	2.049	3.15E-09
At1g03300	ATDUF1 (DOMAIN OF UNKNOWN FUNCTION 724 1); Member of the plant-specific DUF724 prote	-1.166	4.91E-06
At1g03730	unknown protein	1.074	0.0078
At1g03740	Protein kinase superfamily protein	1.94	3.19E-09
At1g03905	P-loop containing nucleoside triphosphate hydrolases superfamily protein	1.226	1.28E-06
At1g04000	unknown protein	1.633	1.97E-06
At1g04490	Protein of unknown function (DUF3527)	1.179	0.00017
At1g04570	Major facilitator superfamily protein	4.242	2.29E-10
At1g04770	Tetratricopeptide repeat (TPR)-like superfamily protein	3.377	4.91E-11
At1g05100	MAPKKK18 (mitogen-activated protein kinase kinase kinase 18); member of MEKK subfamily	1.442	5.15E-07
At1g05170	Galactosyltransferase family protein	1.231	9.60E-07
At1g05250	Peroxidase superfamily protein	1.63	0.0059
At1g05420	ATOFP12 (ARABIDOPSIS THALIANA OVATE FAMILY PROTEIN 12)	-1.121	3.38E-05
At1g05575	unknown protein	4.157	8.71E-11
At1g05670	Pentatricopeptide repeat (PPR-like) superfamily protein	1.552	2.08E-07
At1g05680	UGT74E2 (Uridine diphosphate glycosyltransferase 74E2); Encodes a UDP-glucosyltransferase, UGT7	2.139	0.00021
At1g05700	Leucine-rich repeat transmembrane protein kinase protein	2.112	3.31E-06
At1g06540	unknown protein	-1.046	2.98E-06
At1g06870	Peptidase S24/S26A/S26B/S26C family protein	1.274	1.49E-07
At1g06980	unknown protein	-1.222	2.19E-05
At1g07000	ATEXO70B2 (exocyst subunit exo70 family protein B2); A member of EXO70 gene family, putative ex	2.161	1.16E-06
At1g07135	glycine-rich protein	5.307	9.29E-08
At1g07160	Protein phosphatase 2C family protein	5.156	5.61E-10
At1g07190	BEST Arabidopsis thaliana protein match is: lon protease 1 (TAIR:AT5G26860.1)	1.026	1.83E-07
At1g07330	unknown protein	3.5	3.94E-10
At1g07350	RNA-binding (RRM/RBD/RNP motifs) family protein	2.646	4.21E-10
At1g07400	HSP20-like chaperones superfamily protein	4.644	8.76E-09
At1g07410	ATRAB-A2B (ARABIDOPSIS RAB GTPASE HOMOLOG A2B)	1.063	5.68E-06
At1g07500	unknown protein	4.334	1.00E-10
At1g07520	GRAS family transcription factor	2.456	2.30E-08
At1g07720	KCS3 (3-ketoacyl-CoA synthase 3); Encodes KCS3, a member of the 3-ketoacyl-CoA synthase family i	-1.068	1.18E-05
At1g07900	LBD1 (LOB domain-containing protein 1)	2.846	1.17E-06
At1g08105	hAT-like transposase family (hobo/Ac/Tam3), has a 1.0e-298 P-value blast match to GB:AAD24567 tra	1.713	9.07E-08
At1g08860	BON3 (BONZAI 3); Encodes a copine-like protein, which is a member of a newly identified class of ca	1.637	0.00017
At1g08930	ERD6 (EARLY RESPONSE TO DEHYDRATION 6); encodes a putative sucrose transporter whose ger	2.172	2.34E-09
At1g08940	Phosphoglycerate mutase family protein	2.123	5.34E-09
At1g09070	(AT)SRC2 (SOYBEAN GENE REGULATED BY COLD-2); SRC2 specifically binds the peptide PIEP	1.622	9.17E-07
At1g09140	ATSRP30 (SERINE-ARGININE PROTEIN 30); Encodes a serine-arginine rich RNA binding protein ir	1.189	6.07E-06
At1g09240	ATNAS3 (ARABIDOPSIS THALIANA NICOTIANAMINE SYNTHASE 3); Encodes a nicotianamine	-1.349	0.01974
At1g09932	Phosphoglycerate mutase family protein	2.235	0.0002
At1g09940	HEMA2 (Glutamyl-tRNA reductase family protein); Encodes glutamyl-tRNA reductase. Involved in hei	2.276	2.38E-09
At1g09970	RLK7 (receptor-like kinase 7); RLK7 belongs to a leucine-rich repeat class of receptor-likekinase (LRR	1.303	4.94E-07
At1g10040	alpha/beta-Hydrolases superfamily protein	3.485	5.55E-11
At1g10050	glycosyl hydrolase family 10 protein / carbohydrate-binding domain-containing protein; Encodes a puta	2.758	4.63E-10
At1g10990	unknown protein	1.074	0.00013
At1g11050	Protein kinase superfamily protein	3.144	8.51E-10
At1g11055	Encodes a defensin-like (DEFL) family protein.; Encodes a defensin-like (DEFL) family protein.	1.04	4.88E-07
At1g11100	SNF2 domain-containing protein / helicase domain-containing protein / zinc finger protein-related	2.361	2.20E-09
At1g11540	Sulfite exporter TauE/SafE family protein	-1.905	7.86E-10
At1g11660	heat shock protein 70 (Hsp 70) family protein	1.244	3.25E-07
At1g11803	pseudogene, auxin responsive protein, blastp match of 74% identity and 1.2e-15 P-value to GP|304353(	-2.764	1.66E-08

At1g12030	Protein of unknown function (DUF506)	4.593	3.87E-07
At1g12060	ATBAG5 (BCL-2-associated athanogene 5); A member of Arabidopsis BAG (Bcl-2-associated athanog	1.794	3.95E-08
At1g12064	unknown protein	1.086	0.0018
At1g12294	MIR472A (microRNA472A); Encodes a microRNA that targets several CC-NBS-LRR family members	1.265	8.30E-08
At1g12740	CYP87A2 (cytochrome P450, family 87, subfamily A, polypeptide 2); encodes a protein with cytochron	1.716	5.10E-08
At1g13210	ACA.l (autoinhibited Ca2+/ATPase II)	2.903	7.96E-09
At1g13245	DVL4 (DEVIL 4)	-1.358	3.34E-07
At1g13300	HRS1 (HYPERSENSITIVITY TO LOW PI-ELICITED PRIMARY ROOT SHORTENING 1); Overexp	-1.244	0.02539
At1g13340	Regulator of Vps4 activity in the MVB pathway protein	1.496	0.0257
At1g13600	AtbZIP58 (basic leucine-zipper 58)	-1.889	0.0082
At1g13620	RGF2 (root meristem growth factor 2); Encodes a root meristem growth factor (RGF). Belongs to a fam	1.556	1.54E-07
At1g14040	EXS (ERD1/XPR1/SYG1) family protein	1.222	4.91E-06
At1g14070	FUT7 (fucosyltransferase 7); member of Xyloglucan fucosyltransferase family	1.523	3.56E-06
At1g14200	RING/U-box superfamily protein	4.038	1.54E-10
At1g14205	Ribosomal L18p/L5e family protein	2.072	6.63E-06
At1g14260	RING/FYVE/PHD zinc finger superfamily protein	1.076	1.86E-06
At1g14370	APK2A (protein kinase 2A); Encodes protein kinase APK2a.	3.115	1.36E-09
At1g14420	AT59 (Pectate lyase family protein)	1.205	2.23E-07
At1g14480	Ankyrin repeat family protein	2.723	2.52E-09
At1g14540	Peroxidase superfamily protein	4.865	3.30E-09
At1g14600	Homeodomain-like superfamily protein	-1.112	2.31E-06
At1g14640	SWAP (Suppressor-of-White-APricot)/surp domain-containing protein	1.423	5.91E-07
At1g14870	PCR2 (PLANT CADMIUM RESISTANCE 2)	1.489	0.0065
At1g15190	Fasciclin-like arabinogalactan family protein	2	1.64E-07
At1g15240	Phox-associated domain	1.058	1.68E-06
At1g15430	Protein of unknown function (DUF1644)	2.461	1.40E-09
At1g15520	ABCG40 (ATP-BINDING CASSETTE FAMILY G40); ABC transporter family involved in ABA trans	3.189	0.00933
At1g15530	Concanavalin A-like lectin protein kinase family protein	1.505	3.84E-08
At1g15540	2-oxoglutarate (2OG) and Fe(II)-dependent oxygenase superfamily protein	1.637	5.17E-05
At1g15550	ATGA3OX1 (ARABIDOPSIS THALIANA GIBBERELLIN 3 BETA-HYDROXYLASE 1); Involved ir	-1.618	6.78E-07
At1g15890	Disease resistance protein (CC-NBS-LRR class) family	1.621	2.71E-08
At1g16000	unknown protein	-1.027	0.00832
At1g16030	Hsp70b (heat shock protein 70B)	5.459	1.56E-11
At1g16040	FUNCTIONS IN: molecular_function unknown; INVOLVED IN: GPI anchor biosynthetic process; LO(	1.183	1.75E-06
At1g16090	WAKL7 (wall associated kinase-like 7); WAK-like kinase	1.155	0.00054
At1g16110	WAKL6 (wall associated kinase-like 6); WAK-like kinase	1.867	4.88E-07
At1g16130	WAKL2 (wall associated kinase-like 2); wall-associated kinase like	3.505	6.88E-09
At1g16150	WAKL4 (wall associated kinase-like 4); Encodes a cell-wall associated kinase like protein of the recepto	1.152	0.00021
At1g16420	ATMC8 (ARABIDOPSIS THALIANA METACASPASE 8); Encodes a metacaspase (cysteine-type enc	5.092	2.41E-07
At1g16640	AP2/B3-like transcriptional factor family protein	1.179	2.39E-06
At1g16670	Protein kinase superfamily protein	1.269	6.29E-07
At1g16930	F-box/RNI-like/FBD-like domains-containing protein	1.17	1.03E-05
At1g17147	VQ motif-containing protein	2.241	4.12E-06
At1g17380	JAZ5 (jasmonate-zim-domain protein 5)	1.076	0.0383
At1g17420	LOX3 (lipoxygenase 3); Lipoxygenase	1.691	6.38E-05
At1g17600	Disease resistance protein (TIR-NBS-LRR class) family	1.838	2.44E-09
At1g17610	Disease resistance protein (TIR-NBS class)	1.025	6.05E-05
At1g17750	AtPEPR2 (PEP1 RECEPTOR 2); Encodes PEPR2, a plasma membrane leucine-rich repeat receptor kin	1.524	2.83E-08
At1g17860	Kunitz family trypsin and protease inhibitor protein	1.209	0.0002
At1g17870	ATEGY3 (ETHYLENE-DEPENDENT GRAVITROPISM-DEFICIENT AND YELLOW-GREEN-LIKE	3.497	1.21E-09
At1g18075	MIR159 (MICRORNA 159); Encodes a microRNA that targets several MYB family members. MicroRI	1.055	1.98E-06
At1g18300	atnudt4 (nudix hydrolase homolog 4)	2.506	7.86E-10
At1g18330	EPR1 (EARLY-PHYTOCHROME-RESPONSIVE1); EARLY-PHYTOCHROME-RESPONSIVE1	1.8	1.07E-08
At1g18380	NA	3.344	5.55E-11
At1g18382	other RNA; Potential natural antisense gene, locus overlaps with AT1G18380	1.285	9.68E-09
At1g18390	Protein kinase superfamily protein	2.903	1.53E-10
At1g18570	AtMYB51 (myb domain protein 51); Encodes a member of the R2R3-MYB transcription family. Involv	2.502	1.42E-07
At1g18740	Protein of unknown function (DUF793)	1.513	8.08E-07
At1g18810	phytochrome kinase substrate-related	-1.264	4.01E-05
At1g18890	ATCDPK1 (calcium-dependent protein kinase 1); encodes a calcium-dependent protein kinase whose g	1.472	1.95E-07
At1g19020	unknown protein	4.662	2.31E-09
At1g19180	JAZ1 (jasmonate-zim-domain protein 1); JAZ1 is a nuclear-localized protein involved in jasmonate sigr	2.623	1.28E-06
At1g19210	Integrase-type DNA-binding superfamily protein; encodes a member of the DREB subfamily A-5 of ER	2.857	9.68E-09
At1g19320	Pathogenesis-related thaumatin superfamily protein	1.947	2.63E-06
At1g19380	Protein of unknown function (DUF1195)	1.207	0.00147
At1g19620	unknown protein	-1.633	1.32E-05
At1g19770	ATPUP14 (purine permease 14); Member of a family of proteins related to PUP1, a purine transporter.	2.038	1.97E-07

At1g20070	unknown protein	-2.164	7.71E-08
At1g20310	unknown protein	4.955	8.27E-10
At1g20350	ATTIM17-1 (translocase inner membrane subunit 17-1); mitochondrial inner membrane translocase	1.486	8.75E-06
At1g20470	SAUR-like auxin-responsive protein family	-1.689	3.72E-06
At1g20510	OPCL1 (OPC-8:0 CoA ligase1)	1.722	1.27E-06
At1g20520	Arabidopsis protein of unknown function (DUF241)	1.434	3.08E-07
At1g20640	Plant regulator RWP-RK family protein	2.487	1.06E-09
At1g20823	RING/U-box superfamily protein	3.033	7.26E-07
At1g21010	unknown protein	2.869	1.51E-09
At1g21110	O-methyltransferase family protein	3.12	4.23E-07
At1g21120	O-methyltransferase family protein	3.088	7.12E-09
At1g21130	O-methyltransferase family protein	2.492	1.41E-08
At1g21326	VQ motif-containing protein	2.352	5.89E-08
At1g21460	Nodulin MtN3 family protein	-1.002	2.04E-06
At1g21550	Calcium-binding EF-hand family protein	6.469	4.01E-11
At1g22280	PAPP2C (phytochrome-associated protein phosphatase type 2C); Encodes a phytochrome-associated pr	1.425	2.39E-07
At1g22340	AtUGT85A7 (UDP-glucosyl transferase 85A7)	-1.009	3.99E-05
At1g22470	unknown protein	2.736	1.59E-06
At1g22500	RING/U-box superfamily protein	-1.393	0.00086
At1g22640	ATMYB3 (ARABIDOPSIS THALIANA MYB DOMAIN PROTEIN 3); MYB-type transcription factor	-1.25	4.68E-07
At1g22810	Integrase-type DNA-binding superfamily protein; encodes a member of the DREB subfamily A-5 of ER	6.88	2.24E-12
At1g23180	ARM repeat superfamily protein	1.239	1.21E-07
At1g23340	Protein of Unknown Function (DUF239)	-1.423	0.00041
At1g23390	Kelch repeat-containing F-box family protein	-1.137	0.0001
At1g23550	similar to RCD one 2 (SRO2); FUNCTIONS IN: NAD+ ADP-ribosyltransferase activity; INVOLVED I	2.01	5.78E-08
At1g23710	Protein of unknown function (DUF1645)	2.908	2.65E-08
At1g23830	unknown protein	2.065	3.05E-09
At1g24130	Transducin/WD40 repeat-like superfamily protein	1.753	2.05E-07
At1g24140	Matrixin family protein	5.081	5.84E-08
At1g24145	unknown protein	2.455	1.86E-05
At1g24150	ATFH4 (FORMIN HOMOLOGUE 4); Encodes a group I formin. Localized to cell junctions. Polymeriz	2.762	2.83E-08
At1g24260	AGL9 (AGAMOUS-like 9); Member of the MADs box transcription factor family. SEP3 is redundant v	-1.313	0.0098
At1g24577	unknown protein	-1.737	1.75E-06
At1g24580	RING/U-box superfamily protein	-3.354	0.02009
At1g24909	Glutamine amidotransferase type 1 family protein	1.303	2.01E-05
At1g25390	Protein kinase superfamily protein	1.407	4.55E-08
At1g25400	unknown protein	2.172	1.21E-06
At1g25440	B-box type zinc finger protein with CCT domain	-1.822	3.40E-06
At1g26420	FAD-binding Berberine family protein	1.161	0.00071
At1g26600	CLE9 (CLAVATA3/ESR-RELATED 9); Member of a large family of putative ligands homologous to tl	-1.572	2.82E-08
At1g26762	unknown protein	-1.043	2.90E-05
At1g26770	AT-EXP10 (ARABIDOPSIS THALIANA EXPANSIN 10); Encodes an expansin. Naming convention f	-1.025	4.61E-05
At1g26800	RING/U-box superfamily protein	3.885	2.05E-10
At1g26920	unknown protein	-1.418	5.73E-06
At1g27100	Actin cross-linking protein	1.844	7.65E-08
At1g27170	transmembrane receptors	1.51	3.13E-08
At1g27720	TAF4 (TBP-associated factor 4)	1.89	1.18E-08
At1g27730	STZ (salt tolerance zinc finger); Related to Cys2/His2-type zinc-finger proteins found in higher plants. (	5.915	5.49E-11
At1g27770	ACA1 (autoinhibited Ca2+-ATPase 1); Encodes a chloroplast envelope Ca2+-ATPase with an N-termin	2.289	1.74E-08
At1g27820	Polynucleotidyl transferase, ribonuclease H-like superfamily protein	4.265	1.09E-10
At1g27890	Polynucleotidyl transferase, ribonuclease H-like superfamily protein	4.531	4.89E-11
At1g28160	Integrase-type DNA-binding superfamily protein; encodes a member of the ERF (ethylene response fact	-1.252	0.0034
At1g28190	unknown protein	3.104	1.14E-10
At1g28280	VQ motif-containing protein	1.114	2.39E-05
At1g28310	Dof-type zinc finger DNA-binding family protein	-1.148	2.67E-07
At1g28370	ATERF11 (ERF DOMAIN PROTEIN 11); encodes a member of the ERF (ethylene response factor) sub	3.184	2.95E-09
At1g28380	NSL1 (necrotic spotted lesions 1); This gene is predicted to encode a protein involved in negatively reg	1.946	8.53E-08
At1g28480	GRX480 (Thioredoxin superfamily protein); Encodes GRX480, a member of the glutaredoxin family th	2.576	0.0058
At1g29290	unknown protein	1.173	1.95E-05
At1g29340	ATPUB17 (ARABIDOPSIS THALIANA PLANT U-BOX 17); Encodes a protein containing a UND, a	1.808	1.04E-07
At1g29420	SAUR-like auxin-responsive protein family	-1.9	1.25E-08
At1g29430	SAUR-like auxin-responsive protein family	-2.864	3.08E-09
At1g29440	SAUR-like auxin-responsive protein family	-3.517	8.96E-09
At1g29450	SAUR-like auxin-responsive protein family	-3.261	3.61E-08
At1g29460	SAUR-like auxin-responsive protein family	-3.772	1.45E-10
At1g29500	SAUR-like auxin-responsive protein family	-3.296	2.07E-09
At1g29510	SAUR68 (SMALL AUXIN UPREGULATED 68)	-2.957	3.77E-09

At1g29680	Protein of unknown function (DUF1264)	1.117	9.03E-06
At1g29690	CAD1 (constitutively activated cell death 1); Encodes a protein containing a domain with significant ho	3.235	3.14E-10
At1g30040	ATGA2OX2 (gibberellin 2-oxidase); Encodes a gibberellin 2-oxidase that acts on C-19 gibberellins. At	1.225	0.0131
At1g30070	SGS domain-containing protein	3.048	2.27E-09
At1g30190	unknown protein	2.712	1.92E-10
At1g30370	alpha/beta-Hydrolases superfamily protein	6.077	4.28E-07
At1g30700	FAD-binding Berberine family protein	2.686	6.73E-05
At1g30730	FAD-binding Berberine family protein	1.958	6.57E-08
At1g30755	Protein of unknown function (DUF668)	2.7	1.49E-10
At1g30810	Transcription factor jumonji (jmj) family protein / zinc finger (C5HC2 type) family protein	1.254	5.09E-06
At1g30870	Peroxidase superfamily protein	1.725	0.01081
At1g31130	unknown protein	1.792	5.33E-07
At1g31280	AGO2 (argonaute 2); An Argonaute gene	2.056	1.45E-08
At1g31290	AGO3 (ARGONAUTE 3)	2.28	2.74E-07
At1g31370	Ubiquitin-specific protease family C19-related protein	1.434	1.36E-07
At1g31490	HXXXD-type acyl-transferase family protein	-1.019	0.00119
At1g31540	Disease resistance protein (TIR-NBS-LRR class) family	1.345	1.40E-05
At1g32330	ATHSFA1D (heat shock transcription factor A1D); Member of Heat Stress Transcription Factor (Hsf) fa	1.303	7.29E-07
At1g32640	JAI1 (JASMONATE INSENSITIVE 1); Encodes a MYC-related transcriptional activator with a typical	1.703	4.53E-05
At1g32740	SBP (S-ribonuclease binding protein) family protein	-1.059	1.78E-06
At1g32920	unknown protein	3.061	9.83E-09
At1g32928	unknown protein	2.909	3.77E-09
At1g32960	SBT3.3 (Subtilase family protein)	2.866	4.90E-07
At1g32970	Subtilisin-like serine endopeptidase family protein	2.103	1.28E-06
At1g33030	O-methyltransferase family protein	1.049	0.00351
At1g33055	unknown protein	-1.446	0.02273
At1g33430	Galactosyltransferase family protein	1.019	3.80E-05
At1g33500	unknown protein	1.019	1.71E-05
At1g33560	ADR1 (ACTIVATED DISEASE RESISTANCE 1); Encodes a NBS-LRR disease resistance protein tha	1.137	0.00017
At1g33610	Leucine-rich repeat (LRR) family protein	1.633	5.56E-06
At1g33670	Leucine-rich repeat (LRR) family protein	1.133	1.60E-05
At1g33760	Integrase-type DNA-binding superfamily protein; encodes a member of the DREB subfamily A-4 of ER	3.003	2.09E-05
At1g33770	Protein kinase superfamily protein	1.4	2.90E-05
At1g33855	MuDr-element domain (Mutator-like transposase family, has a 1.7e-18 P-value blast match to Q9SKL7	2.307	1.64E-06
At1g34042	unknown protein	1.352	4.50E-06
At1g34420	leucine-rich repeat transmembrane protein kinase family protein	1.238	0.00023
At1g35140	EXL7 (EXORDIUM LIKE 7); At1g35140 (At1g35140/T32G9_32) mRNA, complete cds	1.286	0.0445
At1g35210	unknown protein	5.799	6.97E-11
At1g35230	AGP5 (arabinogalactan protein 5); Encodes arabinogalactan-protein (AGP5).	2.032	0.01229
At1g35350	EXS (ERD1/XPR1/SYG1) family protein	1.36	2.69E-06
At1g35660	unknown protein	1.73	7.93E-08
At1g36370	SHM7 (serine hydroxymethyltransferase 7); Encodes a putative serine hydroxymethyltransferase.	3.892	4.77E-10
At1g42990	ATBZIP60 (basic region/leucine zipper motif 60); AtbZIP60 consists of a bZIP DNA binding domain fo	2.361	2.37E-10
At1g43000	PLATZ transcription factor family protein	5.001	1.97E-11
At1g43610	Haloacid dehalogenase-like hydrolase (HAD) superfamily protein	1.048	0.00013
At1g43675	expressed protein (transposable element gene); similarity to non-LTR retroelement protein	-1.349	0.00014
At1g44224	ECA1 gametogenesis related family protein; Encodes a ECA1 gametogenesis related family protein	-1.026	3.84E-06
At1g44414	unknown protein	5.531	4.01E-11
At1g45616	AtRLP6 (receptor like protein 6)	-1.182	0.01334
At1g47510	5PTASE11 (inositol polyphosphate 5-phosphatase 11); Encodes a phosphatidylinositol polyphosphate 5	2.351	4.33E-07
At1g47890	AtRLP7 (receptor like protein 7)	4.057	4.40E-06
At1g48780	unknown protein	-1.025	3.71E-05
At1g48930	AtGH9C1 (glycosyl hydrolase 9C1)	1.041	0.01097
At1g49050	Eukaryotic aspartyl protease family protein	1.217	1.33E-05
At1g49200	RING/U-box superfamily protein	-2.077	1.15E-07
At1g49210	RING/U-box superfamily protein	-2.078	3.44E-07
At1g49230	RING/U-box superfamily protein	-1.727	1.23E-08
At1g49370	pseudogene, similar to OSJNBa0072F16.8, blastp match of 42% identity and 6.6e-73 P-value to GP|217	-1.162	5.85E-06
At1g49470	Family of unknown function (DUF716)	-1.024	5.22E-05
At1g49780	PUB26 (plant U-box 26)	1.791	1.54E-06
At1g49790	F-box associated ubiquitination effector family protein	1.407	2.67E-07
At1g50040	Protein of unknown function (DUF1005)	-1.473	0.00059
At1g50420	SCL-3 (SCARECROW-LIKE 3); Encodes a scarecrow-like protein (SCL3) Putative transcription factor	-1.201	3.39E-06
At1g50590	RmlC-like cupins superfamily protein	1.396	6.64E-06
At1g50740	Transmembrane proteins 14C	3.551	1.71E-09
At1g51170	Protein kinase superfamily protein	-1.098	6.00E-06
At1g51270	structural molecules	2.729	1.22E-08

At1g51330	Serine protease inhibitor (SERPIN) family protein	1.3	0.00049
At1g51470	BGLU35 (beta glucosidase 35); Encodes a myrosinase.	1.888	0.00195
At1g51620	Protein kinase superfamily protein	1.788	4.49E-06
At1g51660	ATMEK4 (ARABIDOPSIS THALIANA MITOGEN-ACTIVATED PROTEIN KINASE KINASE 4); E	2.592	3.30E-08
At1g51700	ADOF1 (DOF zinc finger protein 1); Encodes dof zinc finger protein (adof1).	2.062	8.48E-07
At1g51790	Leucine-rich repeat protein kinase family protein	-1.11	0.00487
At1g51820	Leucine-rich repeat protein kinase family protein	1.173	0.00159
At1g51850	Leucine-rich repeat protein kinase family protein	-1.564	0.02395
At1g52560	HSP20-like chaperones superfamily protein	8.048	8.07E-12
At1g52790	2-oxoglutarate (2OG) and Fe(II)-dependent oxygenase superfamily protein; encodes a putative oxidored	2.441	0.00033
At1g52820	2-oxoglutarate (2OG) and Fe(II)-dependent oxygenase superfamily protein	1.445	2.50E-05
At1g52870	Peroxisomal membrane 22 kDa (Mpv17/PMP22) family protein	1.009	0.00051
At1g53060	Legume lectin family protein	1.171	8.71E-06
At1g53080	Legume lectin family protein	2.844	2.31E-09
At1g53350	Disease resistance protein (CC-NBS-LRR class) family	1.008	7.27E-07
At1g53430	Leucine-rich repeat transmembrane protein kinase	2.311	1.69E-09
At1g53540	HSP20-like chaperones superfamily protein	7.776	3.14E-10
At1g53625	unknown protein	1.802	0.00047
At1g53870	Protein of unknown function (DUF567)	-1.292	1.88E-05
At1g54050	HSP20-like chaperones superfamily protein	4.906	5.39E-11
At1g54120	unknown protein	-1.12	4.60E-06
At1g54200	unknown protein	-1.266	2.11E-07
At1g55230	Family of unknown function (DUF716)	1.393	7.16E-05
At1g55300	TAF7 (TBP-associated factor 7)	1.411	6.13E-09
At1g55450	S-adenosyl-L-methionine-dependent methyltransferases superfamily protein	2.537	6.93E-07
At1g55460	DNA/RNA-binding protein Kin17, conserved region	1.267	1.03E-06
At1g55530	RING/U-box superfamily protein	2.643	3.89E-10
At1g55920	ATSERAT2.1 (serine acetyltransferase 2.1); Encodes a chloroplast/cytosol localized serine O-acetyltran	1.514	1.81E-08
At1g56010	anac021 (Arabidopsis NAC domain containing protein 21); Encodes a transcription factor involved in s	-1.749	1.66E-05
At1g56060	unknown protein	4.599	0.00014
At1g56140	Leucine-rich repeat transmembrane protein kinase	1.843	8.21E-10
At1g56240	AtPP2-B13 (phloem protein 2-B13)	4.149	1.95E-06
At1g56250	AtPP2-B14 (phloem protein 2-B14)	3.869	2.19E-07
At1g56300	Chaperone DnaJ-domain superfamily protein	3.173	1.11E-09
At1g56440	Tetratricopeptide repeat (TPR)-like superfamily protein	1.038	6.15E-06
At1g56510	ADR2 (ACTIVATED DISEASE RESISTANCE 2); TIR-NB-LRR protein that confers resistance to fou	1.139	0.0005
At1g56520	Disease resistance protein (TIR-NBS-LRR class) family	1.288	1.85E-05
At1g56540	Disease resistance protein (TIR-NBS-LRR class) family	2.675	4.58E-10
At1g56600	AtGolS2 (galactinol synthase 2)	1.062	0.00053
At1g56710	Pectin lyase-like superfamily protein	-1.196	8.83E-05
At1g57630	Toll-Interleukin-Resistance (TIR) domain family protein	3.067	0.00882
At1g57980	Nucleotide-sugar transporter family protein	1.284	1.93E-05
At1g57990	ATPUP18 (purine permease 18); Member of a family of proteins related to PUP1, a purine transporter.	3.228	1.18E-08
At1g58170	Disease resistance-responsive (dirigent-like protein) family protein	2.564	1.74E-10
At1g58420	Uncharacterised conserved protein UCP031279	2.904	2.16E-07
At1g58807	Disease resistance protein (CC-NBS-LRR class) family	1.082	7.29E-07
At1g59590	ZCF37; ZCF37 mRNA, complete cds	3.275	2.66E-09
At1g59730	ATH7 (thioredoxin H-type 7)	-1.19	0.01368
At1g59860	HSP20-like chaperones superfamily protein	6.173	2.26E-11
At1g59865	unknown protein	1.283	0.001
At1g59870	ABCG36 (ATP-BINDING CASSETTE G36); ATP binding cassette transporter. Localized to the plasm	1.168	1.49E-05
At1g59910	Actin-binding FH2 (formin homology 2) family protein	2.445	2.85E-09
At1g59980	ARL2 (ARG1-like 2)	1.164	9.43E-07
At1g61140	EDA16 (embryo sac development arrest 16)	1.588	7.74E-06
At1g61230	Mannose-binding lectin superfamily protein	1.098	1.47E-06
At1g61255	BEST Arabidopsis thaliana protein match is: glycine-rich protein (TAIR:AT4G21620.2)	-1.001	0.00056
At1g61260	Protein of unknown function (DUF761)	1.091	2.14E-06
At1g61280	Phosphatidylinositol N-acetylglucosaminyltransferase, GPI19/PIG-P subunit	1.329	7.75E-05
At1g61290	SYP124 (syntaxin of plants 124); member of SYP12 Gene Family	3.092	1.36E-08
At1g61360	S-locus lectin protein kinase family protein	1.08	0.0003
At1g61460	S-locus protein kinase, putative	2.267	2.43E-09
At1g61470	Polynucleotidyl transferase, ribonuclease H-like superfamily protein	4.415	3.68E-11
At1g61475	ATP binding	1.674	1.35E-08
At1g61560	ATMLO6 (MILDEW RESISTANCE LOCUS O 6); A member of a large family of seven-transmembran	2.239	1.61E-09
At1g61665	pseudogene, similar to serine/threonine kinase, blastp match of 55% identity and 3.4e-14 P-value to GP	1.237	1.69E-06
At1g61740	Sulfite exporter TauE/SafE family protein	-1.861	2.05E-10
At1g62030	Cysteine/Histidine-rich C1 domain family protein	-1.406	1.53E-07

At1g62180	APR2 (5'adenylylphosphosulfate reductase 2); encodes a adenosine 5'-phosphosulfate reductase, involve	1.402	5.26E-07
At1g62300	WRKY6 (WRKY family transcription factor); Encodes a transcription factor WRKY6. Regulates Phos	1.587	2.24E-06
At1g62420	Protein of unknown function (DUF506)	1.562	6.82E-08
At1g62580	Flavin-binding monooxygenase family protein	-1.597	0.00066
At1g62740	stress-inducible protein, putative	1.22	1.47E-06
At1g63245	CLE14 (CLAVATA3/ESR-RELATED 14); Member of a large family of putative ligands homologous to	1.079	0.00081
At1g63350	Disease resistance protein (CC-NBS-LRR class) family	1.825	1.83E-08
At1g63360	Disease resistance protein (CC-NBS-LRR class) family	1.225	8.03E-06
At1g63720	BEST Arabidopsis thaliana protein match is: hydroxyproline-rich glycoprotein family protein (TAIR:A7	3.088	2.00E-09
At1g63740	Disease resistance protein (TIR-NBS-LRR class) family	1.245	1.69E-07
At1g63750	Disease resistance protein (TIR-NBS-LRR class) family	2.788	1.69E-08
At1g63820	CCT motif family protein	3.132	4.22E-09
At1g63830	PLAC8 family protein	1.761	2.14E-09
At1g63840	RING/U-box superfamily protein	1.595	1.63E-08
At1g63857	NA	1.715	3.85E-07
At1g63860	Disease resistance protein (TIR-NBS-LRR class) family	1.497	8.29E-06
At1g64065	Late embryogenesis abundant (LEA) hydroxyproline-rich glycoprotein family	2.724	8.22E-07
At1g64405	unknown protein	-1.072	6.09E-05
At1g64610	Transducin/WD40 repeat-like superfamily protein	1.721	8.85E-07
At1g64625	Serine/threonine-protein kinase WNK (With No Lysine)-related	-1.034	8.18E-05
At1g65240	Eukaryotic aspartyl protease family protein	1.358	0.002
At1g65310	ATXTH17 (XYLOGLUCAN ENDOTRANSGLUCOSYLASE/HYDROLASE 17); Encodes a xylogluca	1.154	0.00242
At1g65390	ATPP2-A5 (phloem protein 2 A5)	2.217	4.91E-06
At1g65481	unknown protein	-1.368	0.0169
At1g66080	unknown protein	4.443	5.39E-11
At1g66090	Disease resistance protein (TIR-NBS class)	5.923	1.74E-07
At1g66160	CMPG1 (CYS, MET, PRO, and GLY protein 1)	3.274	1.96E-09
At1g66280	BGLU22 (Glycosyl hydrolase superfamily protein)	1.896	0.01622
At1g66400	CML23 (calmodulin like 23); Encodes a calmodulin-like protein. Regulates nitric oxide levels and tran	1.884	1.65E-06
At1g66480	PMI2 (plastid movement impaired 2); Involved in chloroplast avoidance movement under intermediate	1.159	2.62E-05
At1g66500	Pre-mRNA cleavage complex II	3.129	1.72E-08
At1g66510	AAR2 protein family	2.121	8.10E-09
At1g66920	Protein kinase superfamily protein	1.218	0.00017
At1g67060	unknown protein	1.44	4.26E-07
At1g67100	LBD40 (LOB domain-containing protein 40)	-1.336	0.0043
At1g67265	DVL3 (DEVIL 3)	-1.526	1.04E-05
At1g67310	Calmodulin-binding transcription activator protein with CG-1 and Ankyrin domains	1.048	2.58E-06
At1g67360	Rubber elongation factor protein (REF)	2.549	1.37E-08
At1g67365	other RNA; Potential natural antisense gene, locus overlaps with AT1G67370	3.323	8.05E-11
At1g67460	Minichromosome maintenance (MCM2/3/5) family protein	1.784	1.13E-06
At1g67470	Protein kinase superfamily protein	2.202	6.73E-09
At1g67530	ARM repeat superfamily protein	1.121	1.39E-06
At1g67810	SUFE2 (sulfur E2); Encodes a protein capable of stimulating the cysteine desulfurase activity of CpNifS	2.073	3.75E-06
At1g67856	RING/U-box superfamily protein	2.252	1.34E-08
At1g67920	unknown protein	3.983	6.31E-10
At1g67970	HSFA8 (heat shock transcription factor A8); member of Heat Stress Transcription Factor (Hsf) family	2.374	2.32E-08
At1g68300	Adenine nucleotide alpha hydrolases-like superfamily protein	1.013	1.33E-05
At1g68320	AtMYB62 (myb domain protein 62); putative transcription factor: R2R3-MYB transcription family. In	1.652	3.21E-07
At1g68330	unknown protein	1.882	8.95E-08
At1g68390	Core-2/I-branching beta-1,6-N-acetylglucosaminyltransferase family protein	1.093	2.83E-05
At1g68450	VQ motif-containing protein	2.244	1.59E-08
At1g68500	unknown protein	-1.09	7.64E-05
At1g68520	B-box type zinc finger protein with CCT domain	-1.402	0.0003
At1g68620	alpha/beta-Hydrolases superfamily protein	2.924	4.49E-05
At1g68690	Protein kinase superfamily protein	1.975	1.15E-07
At1g68930	pentatricopeptide (PPR) repeat-containing protein	1.805	2.48E-08
At1g69040	ACR4 (ACT domain repeat 4); ACT-domain containing protein involved in feedback regulation of amin	-1.135	1.86E-05
At1g69160	unknown protein	-1.377	3.92E-07
At1g69270	RPK1 (receptor-like protein kinase 1); RPK1 is a leucine-rich receptor-like kinase located in the plasma	1.026	2.32E-06
At1g69430	unknown protein	1.295	4.50E-05
At1g69490	ANAC029 (Arabidopsis NAC domain containing protein 29); Encodes a member of the NAC transcript	1.055	0.0175
At1g69530	ATEXPA1 (expansin A1); Member of Alpha-Expansin Gene Family. Naming convention from the Expa	-1.182	4.42E-06
At1g69570	Dof-type zinc finger DNA-binding family protein	-1.608	7.59E-08
At1g69760	unknown protein	-1.115	1.45E-05
At1g69820	GGT3 (gamma-glutamyl transpeptidase 3); Note that conflicting nomenclature exists in the literature: A	1.794	4.70E-07
At1g69840	SPFH/Band 7/PHB domain-containing membrane-associated protein family	1.577	0.0159
At1g69880	ATH8 (thioredoxin H-type 8)	-1.725	0.0039

At1g69890	Protein of unknown function (DUF569)	3.295	2.49E-08
At1g69900	Actin cross-linking protein	2.462	3.91E-08
At1g69920	ATGSTU12 (ARABIDOPSIS THALIANA GLUTATHIONE S-TRANSFERASE TAU 12); Encodes glu	1.939	0.00016
At1g70140	ATFH8 (formin 8); Encodes a group I formin. Binds to F-actin barbed ends. Has severing actin filament	1.255	5.97E-05
At1g70170	MMP (matrix metalloproteinase); mutant has Late flowering; Early senescence; Matrix Metalloproteina	1.171	0.00028
At1g70290	TPS8 (trehalose-6-phosphatase synthase S8); Encodes an enzyme putatively involved in trehalose biosy	-1.193	0.00022
At1g70300	KUP6 (K+ uptake permease 6); potassium transporter	1.722	1.32E-07
At1g70420	Protein of unknown function (DUF1645)	1.72	4.94E-07
At1g70530	CRK3 (cysteine-rich RLK (RECEPTOR-like protein kinase) 3); Encodes a cysteine-rich receptor-like pr	1.978	2.86E-09
At1g70581	other RNA	1.321	0.00098
At1g70740	Protein kinase superfamily protein	1.733	3.64E-07
At1g70800	Calcium-dependent lipid-binding (CaLB domain) family protein	-1.005	0.0158
At1g70990	proline-rich family protein	2.274	1.54E-08
At1g71000	Chaperone DnaJ-domain superfamily protein	5.757	8.88E-07
At1g71030	ATMYBL2 (ARABIDOPSIS MYB-LIKE 2); Encodes a putative myb family transcription factor. In co	-1.011	0.00225
At1g71230	CSN5B (COP9-signalosome 5B); Encodes a subunit of the COP9 complex, similar to JAB1, a specific	1.521	1.08E-07
At1g71400	AtRLP12 (receptor like protein 12); Encodes a CLAVATA2 (CLV2)-related gene. Complements the clv	2.072	7.63E-08
At1g71520	Integrase-type DNA-binding superfamily protein; encodes a member of the DREB subfamily A-5 of ER	5.135	1.41E-10
At1g71530	Protein kinase superfamily protein	1.254	7.44E-05
At1g71970	unknown protein	-1.004	4.53E-06
At1g72100	late embryogenesis abundant domain-containing protein / LEA domain-containing protein	1.767	6.57E-09
At1g72240	unknown protein	1.631	1.62E-06
At1g72280	AERO1 (endoplasmic reticulum oxidoreductins 1); endoplasmic reticulum oxidoreductin	1.832	1.67E-07
At1g72416	Chaperone DnaJ-domain superfamily protein	2.582	2.30E-09
At1g72660	P-loop containing nucleoside triphosphate hydrolases superfamily protein	6.443	3.22E-13
At1g72830	NF-YA3 (nuclear factor Y, subunit A3); Encodes a subunit of CCAAT-binding complex, binds to CCA	-1.019	3.22E-05
At1g72900	Toll-Interleukin-Resistance (TIR) domain-containing protein	2.495	0.0001
At1g72910	Toll-Interleukin-Resistance (TIR) domain-containing protein	3.911	4.58E-05
At1g72920	Toll-Interleukin-Resistance (TIR) domain family protein	3.515	1.17E-06
At1g72940	Toll-Interleukin-Resistance (TIR) domain-containing protein	2.401	1.97E-07
At1g72950	Disease resistance protein (TIR-NBS class)	2.455	1.72E-08
At1g73080	ATPEPR1 (PEP1 RECEPTOR 1); Encodes a leucine-rich repeat receptor kinase. Functions as a recepto	1.574	1.23E-08
At1g73480	alpha/beta-Hydrolases superfamily protein	1.703	5.24E-06
At1g73500	MKK9 (MAP kinase kinase 9); member of MAP Kinase Kinase family. Autophosphorylates and also ph	1.886	3.24E-08
At1g73540	atnudt21 (nudix hydrolase homolog 21)	1.693	5.10E-08
At1g73740	UDP-Glycosyltransferase superfamily protein	2.81	9.63E-10
At1g73805	Calmodulin binding protein-like	1.962	0.0001
At1g73810	Core-2/I-branching beta-1,6-N-acetylglucosaminyltransferase family protein	4.186	3.87E-05
At1g74310	ATHSP101 (heat shock protein 101); Encodes ClpB1, which belongs to the Casein lytic proteinase/heat	4.459	7.60E-10
At1g74320	Protein kinase superfamily protein; encodes a choline kinase, whose expression is induced by high salt a	1.502	2.22E-07
At1g74330	Protein kinase superfamily protein	1.705	2.55E-08
At1g74360	Leucine-rich repeat protein kinase family protein	2.434	8.56E-06
At1g74440	Protein of unknown function (DUF962)	1.981	2.69E-05
At1g74450	Protein of unknown function (DUF793)	1.974	3.76E-08
At1g74590	ATGSTU10 (GLUTATHIONE S-TRANSFERASE TAU 10); Encodes glutathione transferase belonging	1.06	0.03516
At1g74870	RING/U-box superfamily protein	1.147	0.00015
At1g74940	Protein of unknown function (DUF581)	-1.86	4.67E-08
At1g75000	GNS1/SUR4 membrane protein family	2.109	5.88E-06
At1g75020	LPAT4 (lysophosphatidyl acyltransferase 4)	2	2.97E-07
At1g75270	DHAR2 (dehydroascorbate reductase 2)	1.838	1.35E-06
At1g75580	SAUR-like auxin-responsive protein family	-1.139	0.00282
At1g76040	CPK29 (calcium-dependent protein kinase 29); member of Calcium Dependent Protein Kinase	1.163	0.0002
At1g76070	unknown protein	3.303	1.45E-10
At1g76360	Protein kinase superfamily protein	1.048	6.48E-05
At1g76410	ATL8 (RING/U-box superfamily protein)	-1.108	1.87E-05
At1g76440	HSP20-like chaperones superfamily protein	1.328	6.70E-07
At1g76600	unknown protein	2.816	2.84E-08
At1g76650	CML38 (calmodulin-like 38)	1.22	0.01864
At1g76680	ATOPR1 (ARABIDOPSIS 12-OXOPHYTODIENOATE REDUCTASE 1); Encodes a member of an alp	2.78	3.83E-07
At1g76700	DNAJ heat shock N-terminal domain-containing protein	1.199	1.54E-08
At1g76878	other RNA; Potential natural antisense gene, locus overlaps with AT1G76880	1.5	1.10E-06
At1g76890	GT2 (Duplicated homeodomain-like superfamily protein); encodes a plant trihelix DNA-binding protein	-1.04	4.15E-06
At1g76965	FUNCTIONS IN: molecular_function unknown; INVOLVED IN: biological_process unknown; LOCAT	1.421	1.74E-06
At1g76970	Target of Myb protein 1	1.397	1.08E-07
At1g76980	BEST Arabidopsis thaliana protein match is: embryo defective 2170 (TAIR:AT1G21390.1)	2.941	7.73E-09
At1g77120	ADH (ALCOHOL DEHYDROGENASE); Catalyzes the reduction of acetaldehyde using NADH as redu	-1.456	0.0092
At1g77410	BGAL16 (beta-galactosidase 16)	1.116	4.17E-06

At1g77450	anac032 (NAC domain containing protein 32)	2.54	2.00E-08
At1g77460	Armadillo/beta-catenin-like repeat	1.009	6.55E-05
At1g77530	O-methyltransferase family protein	1.393	7.54E-06
At1g77570	Winged helix-turn-helix transcription repressor DNA-binding	1.461	1.78E-05
At1g77640	Integrase-type DNA-binding superfamily protein; encodes a member of the DREB subfamily A-5 of ER	1.424	3.44E-05
At1g77890	DNA-directed RNA polymerase II protein	1.323	1.15E-06
At1g78170	unknown protein	-1.695	1.57E-08
At1g78270	AtUGT85A4 (UDP-glucosyl transferase 85A4)	-1.212	3.27E-06
At1g78310	VQ motif-containing protein	2.228	4.92E-09
At1g78390	ATNCED9 (NINE-CIS-EPOXYCAROTENOID DIOXYGENASE 9); Encodes 9-cis-epoxycarotenoid d	1.688	3.85E-08
At1g78410	VQ motif-containing protein	4.524	6.56E-09
At1g78450	SOUL heme-binding family protein	-1.02	0.001
At1g78600	DBB3 (DOUBLE B-BOX 3)	1.087	4.64E-06
At1g79110	zinc ion binding	-1.512	1.56E-06
At1g79160	unknown protein	-1.695	1.73E-08
At1g79400	ATCHX2 (cation/H+ exchanger 2); member of Putative Na+/H+ antiporter family	-1.067	0.02908
At1g79410	5-Oct (organic cation/carnitine transporter5)	1.023	3.70E-06
At1g79670	RFO1 (RESISTANCE TO FUSARIUM OXYSPORUM 1); Encodes a receptor-like kinase that does not	1.163	5.25E-08
At1g79680	WAKL10 (WALL ASSOCIATED KINASE (WAK)-LIKE 10)	4.395	3.97E-08
At1g79770	Protein of unknown function (DUF1677)	-1.062	0.0002
At1g79920	Heat shock protein 70 (Hsp 70) family protein	1.004	7.97E-06
At1g80080	AtRLP17 (Receptor Like Protein 17); Encodes a transmembrane leucine-repeat containing receptor-like	-1.214	8.85E-05
At1g80130	Tetratricopeptide repeat (TPR)-like superfamily protein	-1.329	0.0079
At1g80440	Galactose oxidase/kelch repeat superfamily protein	-1.291	6.08E-05
At1g80820	CCR2 (cinnamoyl coa reductase); Encodes an cinnamoyl CoA reductase isoform. Involved in lignin bio	2.464	3.34E-06
At1g80840	WRKY40 (WRKY DNA-binding protein 40); Pathogen-induced transcription factor. Binds W-box sequ	5.571	1.60E-07
At2g01020	5SrRNA (rRNA)	-1.347	0.02794
At2g01180	ATPAP1 (phosphatidic acid phosphatase 1); Encodes phosphatidate phosphatase. Up-regulated by genc	3.208	1.01E-08
At2g01505	CLE16 (CLAVATA3/ESR-RELATED 16); Member of a large family of putative ligands homologous to	-1.15	5.61E-06
At2g01530	MLP329 (MLP-like protein 329)	1.538	0.01625
At2g02220	ATPSKR1 (PHYTOSULFOKIN RECEPTOR 1); Encodes a protein interacting with phytosulfokine, a f	1.658	6.66E-08
At2g02580	CYP71B9 (cytochrome P450, family 71, subfamily B, polypeptide 9); member of CYP71B	-1.107	1.72E-05
At2g02750	Pentatricopeptide repeat (PPR) superfamily protein	1.182	0.0213
At2g02850	ARPN (plantacyanin); Encodes plantacyanin one of blue copper proteins. Involved in anther developme	-1.013	0.00051
At2g02860	ATSUC3 (ARABIDOPSIS THALIANA SUCROSE TRANSPORTER 3); encodes a sucrose transporter	1.551	1.68E-06
At2g02950	PKS1 (phytochrome kinase substrate 1); Encodes a basic soluble protein which can independently bind	-1.456	3.16E-06
At2g03890	ATPI4K GAMMA 7 (phosphoinositide 4-kinase gamma 7); Phosphoinositide kinase which undergo au	-1.134	7.29E-07
At2g04240	XERICO (RING/U-box superfamily protein); Encodes a small protein with an N-terminal trans-membra	-1.126	5.87E-08
At2g04600	similar to unknown protein [Arabidopsis thaliana] (TAIR:AT2G13865.1)	1.716	0.00133
At2g05050	Protein phosphatase 2C family protein	2.483	1.14E-10
At2g05940	Protein kinase superfamily protein	1.519	4.57E-05
At2g06530	VPS2.1 (SNF7 family protein)	1.255	2.77E-06
At2g09994	pseudogene of U-box domain-containing protein / armadillo/beta-catenin repeat family protein; pseudog	1.259	3.70E-05
At2g13790	ATSERK4 (SOMATIC EMBRYOGENESIS RECEPTOR-LIKE KINASE 4)	2.098	1.46E-06
At2g13800	ATSERK5 (SOMATIC EMBRYOGENESIS RECEPTOR LIKE KINASE 5)	1.047	0.0009
At2g14247	Expressed protein	-1.166	0.00564
At2g15040	AtRLP18 (receptor like protein 18)	2.346	5.80E-07
At2g15390	FUT4 (fucosyltransferase 4); Predicted fucosyltransferase, based on similarity to FUT1, but not functior	3.931	9.28E-10
At2g15480	UGT73B5 (UDP-glucosyl transferase 73B5)	2.689	5.60E-09
At2g15490	UGT73B4 (UDP-glycosyltransferase 73B4)	2.99	5.69E-08
At2g15760	Protein of unknown function (DUF1645)	1.412	5.45E-07
At2g16060	AHB1 (hemoglobin 1); Encodes a class 1 nonsymbiotic hemoglobin induced by low oxygen levels with	-1.505	0.0032
At2g16720	ATMYB7 (ARABIDOPSIS THALIANA MYB DOMAIN PROTEIN 7); Encodes a member of MYB3R	1.077	6.45E-06
At2g16870	Disease resistance protein (TIR-NBS-LRR class) family	1.125	1.24E-05
At2g16900	Arabidopsis phospholipase-like protein (PEARLI 4) family	1.114	4.57E-07
At2g17040	anac036 (NAC domain containing protein 36); Member of the NAC transcription factor family and mor	2.084	0.00014
At2g17220	Protein kinase superfamily protein	1.108	3.81E-07
At2g17705	unknown protein	1.8	1.55E-08
At2g17850	Rhodanese/Cell cycle control phosphatase superfamily protein	-1.676	0.0117
At2g17900	SDG37 (SET domain group 37); Homology Subgroup S-ET - Protein containing an interrupted SET dor	1.319	9.82E-07
At2g18010	SAUR-like auxin-responsive protein family	-1.495	1.10E-05
At2g18210	unknown protein	3.733	1.36E-08
At2g18300	basic helix-loop-helix (bHLH) DNA-binding superfamily protein	-1.46	5.18E-07
At2g18560	UDP-Glycosyltransferase superfamily protein	-1.636	2.84E-06
At2g18680	unknown protein	1.752	9.77E-06
At2g19190	FRK1 (FLG22-induced receptor-like kinase 1); Receptor-like protein kinase. Involved in early defense	-1.285	0.0286
At2g19310	HSP20-like chaperones superfamily protein	1.895	6.21E-09

At2g19630	F-box and associated interaction domains-containing protein	1.696	5.31E-10
At2g19710	Regulator of Vps4 activity in the MVB pathway protein	1.993	1.56E-08
At2g19980	CAP (Cysteine-rich secretory proteins, Antigen 5, and Pathogenesis-related 1 protein) superfamily prote	-1.61	1.83E-06
At2g19990	PR-1-LIKE (pathogenesis-related protein-1-like); Encodes a PR-1-like protein homolog that is differenti	-1.011	7.05E-05
At2g20110	Tesmin/TSO1-like CXC domain-containing protein	1.21	5.50E-05
At2g20142	Toll-Interleukin-Resistance (TIR) domain family protein	3.181	1.05E-06
At2g20560	DNAJ heat shock family protein	4.435	1.18E-11
At2g20562	unknown protein	2.582	7.97E-08
At2g20670	Protein of unknown function (DUF506)	-1.076	0.00504
At2g20720	Pentatricopeptide repeat (PPR) superfamily protein	1.976	1.41E-09
At2g20880	Integrase-type DNA-binding superfamily protein	-1.15	0.01253
At2g20960	pEARLI4 (Arabidopsis phospholipase-like protein (PEARLI 4) family)	1.353	5.75E-07
At2g21080	unknown protein	-1.053	3.39E-06
At2g21120	Protein of unknown function (DUF803)	1.098	1.19E-07
At2g21185	unknown protein	-1.13	4.13E-05
At2g21200	SAUR-like auxin-responsive protein family	-1.488	1.74E-07
At2g21210	SAUR-like auxin-responsive protein family ; Putative auxin-regulated protein whose expression is dow	-2.853	1.86E-10
At2g21560	unknown protein	-1.058	1.43E-06
At2g21940	SK1 (shikimate kinase 1)	1.476	8.72E-07
At2g22200	Integrase-type DNA-binding superfamily protein; encodes a member of the DREB subfamily A-6 of ER	-2.164	4.70E-07
At2g22300	CAMTA3 (CALMODULIN-BINDING TRANSCRIPTION ACTIVATOR 3); Encodes a putative CAM	1.727	9.23E-09
At2g22496	MIR779A (microRNA779A); Encodes a microRNA of unknown function. MicroRNAs are regulatory R	3.656	3.11E-09
At2g22500	ATPUMP5 (PLANT UNCOUPLING MITOCHONDRIAL PROTEIN 5); Encodes one of the mitochond	2.536	1.43E-07
At2g22770	NAI1 (basic helix-loop-helix (bHLH) DNA-binding superfamily protein); regulates the development of	-1.157	3.01E-05
At2g22800	HAT9 (Homeobox-leucine zipper protein family); Encodes homeobox protein HAT9.	-1.418	1.11E-07
At2g22810	ACC4 (1-AMINOCYCLOPROPANE-1-CARBOXYLIC ACID SYNTHASE POLYPEPTIDE); key reg	-1.444	2.42E-05
At2g22880	VQ motif-containing protein	3.555	1.09E-07
At2g23110	Late embryogenesis abundant protein, group 6	1.336	0.00024
At2g23270	unknown protein	2.328	4.50E-08
At2g23320	WRKY15 (WRKY DNA-binding protein 15); Encodes WRKY DNA-binding protein 15 (WRKY15).	1.829	2.43E-08
At2g23430	KRP1 (KIP-RELATED PROTEIN 1); Encodes a cyclin-dependent kinase inhibitor protein that function	-1.407	4.25E-06
At2g23680	Cold acclimation protein WCOR413 family	1.911	2.43E-07
At2g23755	unknown protein	-1.412	2.21E-08
At2g23770	protein kinase family protein / peptidoglycan-binding LysM domain-containing protein	2.68	8.34E-07
At2g23810	TET8 (tetraspanin8); Member of TETRASPANIN family	3.145	1.14E-07
At2g24100	unknown protein	1.352	6.82E-08
At2g24165	pseudogene, similar to HcrVf3 protein, blastp match of 37% identity and 5.1e-32 P-value to GP|143307	1.929	2.16E-07
At2g24600	Ankyrin repeat family protein	3.259	2.41E-08
At2g24610	ATCNGC14 (cyclic nucleotide-gated channel 14); member of Cyclic nucleotide gated channel family	1.691	6.58E-08
At2g25140	CLPB-M (CASEIN LYTIC PROTEINASE B-M); Encodes ClpB4, which belongs to the Casein lytic pro	3.435	7.49E-10
At2g25200	Plant protein of unknown function (DUF868)	-1.176	9.02E-05
At2g25460	CONTAINS InterPro DOMAIN/s: C2 calcium-dependent membrane targeting (InterPro:IPR000008); B	2.22	1.43E-06
At2g25680	MOT1 (molybdate transporter 1); Encodes a high-affinity molybdate transporter. Mutant has reduced co	-1.014	2.98E-05
At2g25735	unknown protein	3.254	2.59E-09
At2g25780	Protein of unknown function (DUF1677)	-1.242	0.00018
At2g26130	RING/U-box protein with C6HC-type zinc finger	-1.126	0.00555
At2g26140	ftsh4 (FTSH protease 4); encodes an FtsH protease that is localized to the mitochondrion	1.032	3.47E-06
At2g26150	ATHSFA2 (heat shock transcription factor A2); member of Heat Stress Transcription Factor (Hsf) famil	6.525	5.40E-11
At2g26190	calmodulin-binding family protein	2.949	6.49E-09
At2g26520	unknown protein	1.005	9.89E-05
At2g26530	AR781 (Protein of unknown function (DUF1645)); unknown function	3.747	6.19E-10
At2g26560	PLA IIA (PHOSPHOLIPASE A 2A); Encodes a lipid acyl hydrolase with wide substrate specificity tha	1.957	0.0021
At2g26710	BAS1 (PHYB ACTIVATION TAGGED SUPPRESSOR 1); Encodes a member of the cytochrome p450	-1.054	1.76E-05
At2g26975	Ctr copper transporter family	1.093	9.06E-07
At2g27080	Late embryogenesis abundant (LEA) hydroxyproline-rich glycoprotein family	1.599	7.72E-05
At2g27260	Late embryogenesis abundant (LEA) hydroxyproline-rich glycoprotein family	1.309	1.43E-05
At2g27310	F-box family protein	2.021	1.42E-07
At2g27570	P-loop containing nucleoside triphosphate hydrolases superfamily protein	1.099	5.15E-05
At2g27580	A20/AN1-like zinc finger family protein	2.331	2.35E-09
At2g27830	unknown protein	1.226	0.0032
At2g28040	Eukaryotic aspartyl protease family protein	1.561	1.22E-06
At2g28056	MIR172 (MICRORNA 172); Encodes a microRNA that targets several genes containing AP2 domains i	4.105	8.07E-12
At2g28180	CHX8 (CATION/H+ EXCHANGER 8); member of Putative Na+/H+ antiporter family	1.328	0.00057
At2g28210	ACA2 (alpha carbonic anhydrase 2)	1.065	0.0308
At2g28400	Protein of unknown function, DUF584	1.838	7.37E-06
At2g28410	unknown protein	-1.13	2.81E-05
At2g28500	LBD11 (LOB domain-containing protein 11)	1.326	1.84E-06

At2g28550	RAP2.7 (related to AP2.7)	-1.381	3.03E-08
At2g28650	ATEXO70H8 (exocyst subunit exo70 family protein H8); A member of EXO70 gene family, putative ex	1.761	4.65E-05
At2g28830	PUB12 (PLANT U-BOX 12)	1.621	3.09E-09
At2g28870	unknown protein	-1.61	1.08E-07
At2g29060	GRAS family transcription factor	1.565	1.46E-07
At2g29300	NAD(P)-binding Rossmann-fold superfamily protein	-1.041	0.00098
At2g29370	NAD(P)-binding Rossmann-fold superfamily protein	-1.098	0.00147
At2g29420	ATGSTU7 (glutathione S-transferase tau 7); Encodes glutathione transferase belonging to the tau class	1.373	1.75E-07
At2g29450	ATGSTU1 (ARABIDOPSIS THALIANA GLUTATHIONE S-TRANSFERASE TAU 1); Encodes a me	1.669	9.34E-07
At2g29480	ATGSTU2 (glutathione S-transferase tau 2); Encodes glutathione transferase belonging to the tau class	1.726	8.10E-06
At2g29490	ATGSTU1 (glutathione S-transferase TAU 1); Encodes glutathione transferase belonging to the tau clas	1.34	6.35E-05
At2g29500	HSP20-like chaperones superfamily protein	5.567	6.47E-10
At2g29720	CTF2B (FAD/NAD(P)-binding oxidoreductase family protein); Encodes CTF2B.	3.171	1.95E-11
At2g29740	UGT71C2 (UDP-glucosyl transferase 71C2)	1.765	0.0002
At2g29870	Aquaporin-like superfamily protein	-1.72	0.03085
At2g30000	PHF5-like protein	2.196	3.87E-10
At2g30020	Protein phosphatase 2C family protein; Encodes AP2C1. Belongs to the clade B of the PP2C-superfami	2.565	3.72E-11
At2g30424	TCL2 (TRICHOMELESS 2); In a tandem repeat with AT2g30432 (TCL1) and AT2g30420 (ETC2)	-1.166	1.38E-06
At2g30480	unknown protein	1.186	3.24E-06
At2g30600	BTB/POZ domain-containing protein	-1.128	0.00034
At2g31230	ATERF15 (ethylene-responsive element binding factor 15); encodes a member of the ERF (ethylene res	1.727	6.49E-07
At2g31680	AtRABA5d (RAB GTPase homolog A5D)	1.053	8.26E-07
At2g31865	PARG2 (poly(ADP-ribose) glycohydrolase 2)	1.563	0.00053
At2g31880	EVR (EVERSHED); Encodes a putative leucine rich repeat transmembrane protein that is expressed in	2.016	3.07E-06
At2g31945	unknown protein	5.074	5.53E-10
At2g31990	Exostosin family protein	2.389	4.09E-09
At2g32010	CVL1 (CVP2 like 1); Encodes an inositol polyphosphate 5'-phosphatase (5PTase). Mediating phospho	-1.015	5.05E-06
At2g32020	Acyl-CoA N-acyltransferases (NAT) superfamily protein	2.951	1.06E-09
At2g32030	Acyl-CoA N-acyltransferases (NAT) superfamily protein	5.697	1.91E-11
At2g32100	ATOFP16 (RABIDOPSIS THALIANA OVATE FAMILY PROTEIN 16)	-2.937	1.68E-07
At2g32120	HSP70T-2 (heat-shock protein 70T-2)	5.96	1.14E-11
At2g32130	Plant protein of unknown function (DUF641)	4.277	1.41E-08
At2g32140	transmembrane receptors	4.214	1.76E-09
At2g32190	unknown protein	2.062	0.00011
At2g32200	unknown protein	3.368	4.57E-07
At2g32210	unknown protein	3.473	1.27E-06
At2g33170	Leucine-rich repeat receptor-like protein kinase family protein	-1.005	8.01E-05
At2g33580	Protein kinase superfamily protein	3.233	4.18E-08
At2g33585	unknown protein	1.719	1.04E-08
At2g33590	NAD(P)-binding Rossmann-fold superfamily protein	2.456	4.46E-08
At2g33700	Protein phosphatase 2C family protein	1.284	1.25E-08
At2g34010	unknown protein	-1.794	2.08E-06
At2g34030	Calcium-binding EF-hand family protein	1.17	0.0004
At2g34040	Apoptosis inhibitory protein 5 (API5)	1.138	5.00E-06
At2g34390	NIP2.1 (NOD26-like intrinsic protein 2.1); aquaporin NIP2.1	-1.494	0.02531
At2g34650	ABR (ABRUPTUS); Encodes a protein serine/threonine kinase that may act as a positive regulator of ce	1.215	5.53E-06
At2g35000	RING/U-box superfamily protein; E3 ligase-like protein induced by chitin oligomers.	1.06	2.29E-06
At2g35035	URED (urease accessory protein D); Encodes a urease accessory protein which is essential for the activa	1.648	1.33E-07
At2g35658	unknown protein	4.369	8.50E-10
At2g35710	Nucleotide-diphospho-sugar transferases superfamily protein	3.222	2.98E-08
At2g35930	PUB23 (plant U-box 23); Encodes a cytoplasmically localized U-box domain containing E3 ubiquitin li	3.545	1.91E-11
At2g35980	ATNHL10 (ARABIDOPSIS NDR1/HIN1-LIKE 10); Encodes a protein whose sequence is similar to tol	2.78	0.03037
At2g36050	ATOFP15 (ARABIDOPSIS THALIANA OVATE FAMILY PROTEIN 15)	-1.03	9.21E-05
At2g36220	unknown protein	4.049	5.20E-10
At2g36630	Sulfite exporter TauE/SafE family protein	-1.459	3.19E-06
At2g36770	UDP-Glycosyltransferase superfamily protein	2.674	9.74E-10
At2g36950	Heavy metal transport/detoxification superfamily protein	1.375	1.93E-06
At2g37430	C2H2 and C2HC zinc fingers superfamily protein	5.979	3.03E-11
At2g37710	RLK (receptor lectin kinase); Induced in response to Salicylic acid.	1.671	7.13E-07
At2g37740	ATZFP10 (ZINC-FINGER PROTEIN 10)	-1.354	0.00496
At2g37880	Protein of unknown function, DUF617	1.02	3.92E-05
At2g37940	AtIPCS2 (Arabidopsis Inositol phosphorylceramide synthase 2); I	2.268	5.50E-08
At2g37980	O-fucosyltransferase family protein	1.34	2.05E-06
At2g38250	Homeodomain-like superfamily protein	1.855	1.40E-06
At2g38340	Integrase-type DNA-binding superfamily protein; encodes a member of the DREB subfamily A-2 of ER	4.404	1.01E-08
At2g38470	ATWRKY33 (WRKY DNA-BINDING PROTEIN 33); Member of the plant WRKY transcription factor	4.639	4.21E-10
At2g38790	unknown protein	2.146	2.03E-07

At2g38820	Protein of unknown function (DUF506)	-1.128	9.53E-07
At2g39030	Acyl-CoA N-acyltransferases (NAT) superfamily protein	-1.754	0.02572
At2g39650	Protein of unknown function (DUF506)	4.607	2.89E-10
At2g39660	BIK1 (botrytis-induced kinase1); Encodes a plasma membrane-localized ser/thr protein kinase that is a	1.69	2.71E-08
At2g39681	TAS2 (trans-acting siRNA2); Trans-acting siRNA primary transcript. RDR6-dependent trans-acting siR	-1.049	2.03E-05
At2g40000	ATHSPRO2 (ARABIDOPSIS ORTHOLOG OF SUGAR BEET HS1 PRO-1 2)	3.215	7.43E-08
At2g40090	ATATH9 (ABC2 homolog 9); member of ATH subfamily	1.223	4.50E-06
At2g40095	Alpha/beta hydrolase related protein	4.158	7.39E-11
At2g40113	Pollen Ole e 1 allergen and extensin family protein	1.115	0.00045
At2g40140	SZF2 ((SALT-INDUCIBLE ZINC FINGER 2)	3.686	3.33E-09
At2g40180	ATHPP2C5 (phosphatase 2C5); Encodes PP2C5, a member of the PP2C family phosphatases. PP2C5 a	1.078	0.00115
At2g40330	PYL6 (PYR1-like 6); Encodes a member of the PYR (pyrabactin resistance)/PYL(PYR1-like)/RCAR (	-1.796	9.17E-07
At2g40340	DREB2C (Integrase-type DNA-binding superfamily protein); Encodes a member of the DREB subfamil	3.258	1.96E-09
At2g40435	BEST Arabidopsis thaliana protein match is: transcription regulators (TAIR:AT3G56220.1)	-1.043	0.00014
At2g40475	unknown protein	-1.277	1.79E-06
At2g40610	ATEXPA8 (expansin A8); member of Alpha-Expansin Gene Family. Naming convention from the Expa	-1.232	0.0011
At2g40670	ARR16 (response regulator 16); response regulator 16	-1.101	3.07E-05
At2g40740	ATWRKY55 (WRKY DNA-BINDING PROTEIN 55); member of WRKY Transcription Factor; Group	1.218	0.00249
At2g41010	ATCAMBP25 (calmodulin (CAM)-binding protein of 25 kDa); Encodes a novel calmodulin binding pro	2.609	8.95E-09
At2g41100	ATCAL4 (ARABIDOPSIS THALIANA CALMODULIN LIKE 4); encodes a calmodulin-like protein, v	2.194	1.25E-07
At2g41110	CAM2 (calmodulin 2); Encodes a touch-inducible calmodulin that has higher affinity to kinesin-like cal	1.183	1.70E-07
At2g41160	Ubiquitin-associated (UBA) protein	1.827	1.95E-09
At2g41230	unknown protein	-1.396	0.00127
At2g41280	M10; Encodes a hydrophilic protein similar to Late Embryogenesis Activated (LEA) proteins expressed	1.176	2.97E-06
At2g41342	unknown protein	1.693	9.78E-07
At2g41640	Glycosyltransferase family 61 protein	3.436	8.19E-08
At2g41800	Protein of unknown function, DUF642	1.843	0.00121
At2g41835	zinc finger (C2H2 type, AN1-like) family protein	3.761	2.04E-11
At2g41940	ZFP8 (zinc finger protein 8); Encodes a zinc finger protein containing only a single zinc finger.	-1.104	2.15E-06
At2g42280	basic helix-loop-helix (bHLH) DNA-binding superfamily protein	-1.286	8.63E-08
At2g42360	RING/U-box superfamily protein	1.053	0.0137
At2g42510	FUNCTIONS IN: molecular_function unknown; INVOLVED IN: spliceosome assembly, nuclear mRN/	1.005	0.0001
At2g42870	HLH1 (HELIX-LOOP-HELIX 1); Encodes PHYTOCHROME RAPIDLY REGULATED1 (PAR1), an a	-1.546	2.22E-05
At2g42950	Magnesium transporter CorA-like family protein	1.595	6.82E-08
At2g42980	Eukaryotic aspartyl protease family protein	2.65	9.33E-07
At2g43000	anac042 (NAC domain containing protein 42)	1.714	0.00106
At2g43120	RmlC-like cupins superfamily protein	1.697	1.67E-05
At2g43320	S-adenosyl-L-methionine-dependent methyltransferases superfamily protein	1.642	1.03E-06
At2g43375	other RNA	1.05	1.02E-07
At2g43445	F-box and associated interaction domains-containing protein	-1.139	3.93E-06
At2g43500	Plant regulator RWP-RK family protein	1.348	9.10E-06
At2g43620	Chitinase family protein	-1.763	0.00243
At2g44080	ARL (ARGOS-like); Encodes ARL, a gene similar to ARGOS involved in cell expansion-dependent org	1.032	2.35E-05
At2g44170	ATNMT2 (ARABIDOPSIS N-MYRISTOYLTRANSFERASE 2)	1.057	5.20E-05
At2g44230	Plant protein of unknown function (DUF946)	-1.147	3.58E-05
At2g44460	BGLU28 (beta glucosidase 28)	1.724	0.00023
At2g44490	BGLU26 (BETA GLUCOSIDASE 26); Encodes a glycosyl hydrolase that localizes to peroxisomes and	1.419	9.03E-07
At2g44500	O-fucosyltransferase family protein	2.125	5.42E-07
At2g44578	RING/U-box superfamily protein	2.412	1.92E-08
At2g44581	RING/U-box superfamily protein	1.375	3.20E-07
At2g44600	unknown protein	1.082	3.36E-05
At2g44840	ATERF13 (ETHYLENE-RESPONSIVE ELEMENT BINDING FACTOR 13); encodes a member of the	5.319	1.25E-07
At2g44940	Integrase-type DNA-binding superfamily protein; encodes a member of the DREB subfamily A-4 of ER	-1.476	3.70E-07
At2g45315	other RNA; Potential natural antisense gene, locus overlaps with AT2G45310	-1.027	7.86E-06
At2g45760	BAL (BON ASSOCIATION PROTEIN 1-LIKE); encodes a protein that is similar to BONZAI1-binding	3.517	0.00253
At2g45900	Phosphatidylinositol N-acetyglucosaminlytransferase subunit P-related	-1.154	0.00025
At2g46240	ATBAG6 (ARABIDOPSIS THALIANA BCL-2-ASSOCIATED ATHANOGENE 6); A member of Ara	6.759	1.41E-11
At2g46330	AGP16 (arabinogalactan protein 16); Encodes arabinogalactan protein (AGP16).	1.499	1.66E-06
At2g46400	ATWRKY46 (WRKY DNA-BINDING PROTEIN 46); member of WRKY Transcription Factor; Group	4.166	6.67E-07
At2g46410	CPC (CAPRICE); Nuclear-localized R3-type MYB transcription factor. Positive regulator of hair-cell di	1.106	0.00026
At2g46430	ATCNGC3 (cyclic nucleotide gated channel 3); cyclic nucleotide gated channel (CNGC4), downstream	1.17	0.00054
At2g46600	Calcium-binding EF-hand family protein	1.211	5.67E-05
At2g46620	P-loop containing nucleoside triphosphate hydrolases superfamily protein	1.3	3.01E-06
At2g46660	CYP78A6 (cytochrome P450, family 78, subfamily A, polypeptide 6); member of CYP78A	-1.29	1.70E-06
At2g46690	SAUR-like auxin-responsive protein family	-1.109	7.09E-06
At2g46735	unknown protein	2.187	1.74E-05
At2g46940	unknown protein	1.348	1.43E-05

At2g46970	PIL1 (phytochrome interacting factor 3-like 1); encodes a novel Myc-related bHLH transcription factor,	-1.008	0.0003
At2g47060	Protein kinase superfamily protein	1.933	2.32E-07
At2g47130	NAD(P)-binding Rossmann-fold superfamily protein	1.574	0.0002
At2g47140	NAD(P)-binding Rossmann-fold superfamily protein	3.063	1.71E-09
At2g47150	NAD(P)-binding Rossmann-fold superfamily protein	1.033	0.00042
At2g47180	AtGolS1 (galactinol synthase 1)	2.25	7.47E-08
At3g01070	ENODL16 (early nodulin-like protein 16)	3.072	2.28E-10
At3g01175	Protein of unknown function (DUF1666)	1.614	5.12E-05
At3g01190	Peroxidase superfamily protein	1.576	0.00332
At3g01290	SPFH/Band 7/PHB domain-containing membrane-associated protein family	1.515	2.87E-06
At3g01350	Major facilitator superfamily protein	-1.123	1.14E-07
At3g01420	ALPHA-DOX1 (alpha-dioxygenase 1); Encodes an alpha-dioxygenase involved in protection against ox	1.214	0.03576
At3g02020	AK3 (aspartate kinase 3); encodes a monofunctional aspartate kinase	-1.207	3.29E-05
At3g02170	LNG2 (LONGIFOLIA2); Encodes LONGIFOLIA2 (LNG2). Regulates leaf morphology by promoting c	-1.045	2.61E-05
At3g02240	RGF7 (root meristem growth factor 7); Encodes a root meristem growth factor (RGF). Belongs to a fam	-1.125	0.02274
At3g02800	Tyrosine phosphatase family protein	2.466	1.61E-09
At3g02840	ARM repeat superfamily protein	5.543	6.69E-10
At3g02990	ATHSFA1E (heat shock transcription factor A1E); member of Heat Stress Transcription Factor (Hsf) fa	1.731	1.05E-05
At3g03820	SAUR-like auxin-responsive protein family	-2.748	5.59E-08
At3g03840	SAUR-like auxin-responsive protein family	-1.27	1.13E-05
At3g03850	SAUR-like auxin-responsive protein family	-2.462	3.65E-07
At3g03855	expressed protein, similar to GB:AAC72977 (Arabidopsis thaliana); pseudogene of disease resistance p	1.517	2.96E-07
At3g04110	GLR1 (GLUTAMATE RECEPTOR 1); putative glutamate receptor (GLR1.1). Contains a functional ca	1.513	1.40E-06
At3g04200	RmlC-like cupins superfamily protein	1.449	0.00031
At3g04210	Disease resistance protein (TIR-NBS class)	1.517	0.00014
At3g04220	Disease resistance protein (TIR-NBS-LRR class) family	1.699	9.30E-07
At3g04300	RmlC-like cupins superfamily protein	3.051	2.50E-09
At3g04640	glycine-rich protein	2.572	5.11E-06
At3g04710	ankyrin repeat family protein	1.949	4.16E-08
At3g04720	HEL (HEVEIN-LIKE); Encodes a protein similar to the antifungal chitin-binding protein hevein from ru	-1.002	0.00224
At3g05030	NHX2 (sodium hydrogen exchanger 2); member of Sodium proton exchanger family	1.795	9.04E-09
At3g05120	ATGID1A (GA INSENSITIVE DWARF1A); Encodes a gibberellin (GA) receptor ortholog of the rice G	-1.18	7.56E-08
At3g05200	ATL6 (RING/U-box superfamily protein); Encodes a putative RING-H2 zinc finger protein ATL6 (ATL	2.491	1.87E-08
At3g05320	O-fucosyltransferase family protein	2.204	3.47E-07
At3g05340	Tetratricopeptide repeat (TPR)-like superfamily protein	1.299	5.07E-06
At3g05360	AtRLP30 (receptor like protein 30)	2.08	1.67E-05
At3g05370	AtRLP31 (receptor like protein 31)	2.747	2.79E-08
At3g05400	Major facilitator superfamily protein	1.851	3.00E-07
At3g05580	Calcineurin-like metallo-phosphoesterase superfamily protein	1.051	1.08E-06
At3g05820	At-A/N-InvH (Arabidopsis alkaline/neutral invertase H); Encodes a putative plastid-targeted alkaline/ne	1.334	1.08E-06
At3g05905	other RNA; Potential natural antisense gene, locus overlaps with AT3G05900	1.044	3.01E-05
At3g06070	unknown protein	-1.497	2.44E-05
At3g06660	PAPA-1-like family protein / zinc finger (HIT type) family protein	1.078	7.95E-06
At3g06890	unknown protein	1.696	2.52E-06
At3g07090	PPPDE putative thiol peptidase family protein	3.035	1.75E-10
At3g07150	unknown protein	4.614	2.24E-12
At3g07195	RPM1-interacting protein 4 (RIN4) family protein	1.639	1.99E-05
At3g07215	other RNA; Unknown gene	1.143	1.22E-06
At3g08690	UBC11 (ubiquitin-conjugating enzyme 11)	1.303	1.64E-06
At3g08710	ATH9 (thioredoxin H-type 9); Associated to plasma membrane. Moves cell to cell, suggesting a role in	1.886	1.30E-08
At3g08720	ATPK19 (Arabidopsis thaliana protein kinase 19); Encodes a ribosomal-protein S6 kinase. Gene expres	3.433	1.06E-09
At3g08760	ATSIK (Protein kinase superfamily protein); Encodes an osmotic stress-inducible kinase that functions	1.489	1.28E-06
At3g08870	Concanavalin A-like lectin protein kinase family protein	1.826	1.97E-06
At3g08970	TMS1 (THERMOSENSITIVE MALE STERILE 1); J domain protein localized in ER lumen. Can comp	4.614	4.80E-10
At3g09010	Protein kinase superfamily protein	1.465	8.11E-07
At3g09020	alpha 1,4-glycosyltransferase family protein	2.238	1.53E-07
At3g09032	unknown protein	2.131	2.76E-09
At3g09280	unknown protein	1.57	3.13E-05
At3g09350	Fes1A (Fes1A); Encodes one of the Arabidopsis orthologs of the human Hsp70-binding protein 1 (HspB	3.805	6.64E-11
At3g09440	Heat shock protein 70 (Hsp 70) family protein	1.074	9.01E-05
At3g09450	CONTAINS InterPro DOMAIN/s: Fusaric acid resistance protein, conserved region (InterPro:IPR00672	-1.098	0.0004
At3g09520	ATEXO70H4 (exocyst subunit exo70 family protein H4); A member of EXO70 gene family, putative ex	3.788	3.62E-10
At3g09640	APX1B (ASCORBATE PEROXIDASE 1B); Encodes a cytosolic ascorbate peroxidase APX2. Ascorba	6.799	2.77E-10
At3g09830	Protein kinase superfamily protein	2.238	8.78E-08
At3g09870	SAUR-like auxin-responsive protein family	5.12	2.56E-09
At3g10020	unknown protein	2.535	3.62E-08
At3g10040	sequence-specific DNA binding transcription factors	-1.287	0.03457

At3g10114	pseudogene of glycosyl hydrolase family 81 protein; pseudogene of glycosyl hydrolase family 81 protein	2.128	6.46E-09
At3g10500	anac053 (NAC domain containing protein 53)	1.383	4.53E-07
At3g10525	LGO (LOSS OF GIANT CELLS FROM ORGANS); Encodes LGO (loss of giant cells from organs) req	-1.041	0.0002
At3g10640	VPS60.1 (SNF7 family protein)	1.908	3.90E-10
At3g10800	BZIP28 (Basic-leucine zipper (bZIP) transcription factor family protein); Encodes bZIP28, a putative m	1.537	9.30E-08
At3g10815	RING/U-box superfamily protein	3.171	8.86E-09
At3g10820	Transcription elongation factor (TFIIS) family protein	1.658	1.03E-07
At3g10930	unknown protein	3.43	1.14E-11
At3g10985	ATWI-12 (ARABIDOPSIS THALIANA WOUND-INDUCED PROTEIN 12); A senescence-associated	1.609	3.25E-06
At3g11020	DREB2 (DEHYDRATION-RESPONSIVE ELEMENT BINDING PROTEIN 2); encodes a member of t	4.869	2.49E-11
At3g11650	NHL2 (NDR1/HIN1-like 2); Encodes a protein whose sequence is similar to tobacco hairpin-induced ge	1.045	2.16E-07
At3g11820	ATSYR1 (SYNTAXIN RELATED PROTEIN 1); Encodes a syntaxin localized at the plasma membrane	2.806	3.58E-09
At3g11840	PUB24 (plant U-box 24); Encodes a U-box-domain-containing E3 ubiquitin ligase that acts as a negativ	3.824	9.42E-10
At3g12040	DNA-3-methyladenine glycosylase (MAG)	1.368	6.20E-08
At3g12050	Aha1 domain-containing protein	1.749	9.60E-09
At3g12360	ITN1 (INCREASED TOLERANCE TO NACL); Encodes a protein with an ankyrin motif and transmem	1.028	3.06E-08
At3g12520	SULTR4.2 (sulfate transporter 4.2); Encodes a sulfate transporter that in induced under sulfate limitatio	1.562	2.47E-08
At3g12580	ATHSP70 (ARABIDOPSIS HEAT SHOCK PROTEIN 70)	2.346	1.19E-07
At3g12740	ALIS1 (ALA-interacting subunit 1); Physically interacts with ALA3, and is required for the phospholip	1.989	8.62E-09
At3g12910	NAC (No Apical Meristem) domain transcriptional regulator superfamily protein	5.604	5.89E-09
At3g12920	SBP (S-ribonuclease binding protein) family protein	-1.715	1.55E-08
At3g12960	unknown protein	1.393	2.27E-08
At3g13229	Plant protein of unknown function (DUF868)	1.029	4.33E-05
At3g13320	CAX2 (cation exchanger 2); low affinity calcium antiporter CAX2	1.09	2.80E-07
At3g13435	unknown protein	1.063	0.00035
At3g13437	unknown protein	1.252	8.22E-06
At3g13600	calmodulin-binding family protein	1.427	2.29E-06
At3g13850	LBD22 (LOB domain-containing protein 22)	1.214	6.73E-05
At3g13980	unknown protein	-1.803	3.82E-08
At3g14050	AT-RSH2 (RELA-SPOT HOMOLOG 2)	2.406	2.49E-07
At3g14200	Chaperone DnaJ-domain superfamily protein	3.848	2.04E-11
At3g14225	GLIP4 (GDSL-motif lipase 4); Contains lipase signature motif and GDSL domain.	1.42	6.09E-08
At3g14440	NCED3 (nine-cis-epoxycarotenoid dioxygenase 3); Encodes 9-<i>cis</i>-epoxycarotenoid dioxygenas	1.49	0.0003
At3g14560	unknown protein	-1.183	7.29E-07
At3g15210	ATERF-4 (ETHYLENE RESPONSIVE ELEMENT BINDING FACTOR 4); Encodes a member of the I	1.086	3.62E-06
At3g15340	PPI2 (proton pump interactor 2); Hypothetical protein similar to PPI1, a protein that interacts with the p	4.126	1.51E-10
At3g15356	Legume lectin family protein	1.243	0.00027
At3g15450	Aluminium induced protein with YGL and LRDR motifs	-1.062	0.0006
At3g15500	ANAC055 (NAC domain containing protein 55); Encodes an ATAF-like NAC-domain transcription fac	-1.011	0.01051
At3g15518	unknown protein	3.631	1.25E-09
At3g15570	Phototropic-responsive NPH3 family protein	-1.066	4.61E-06
At3g15630	unknown protein	-1.229	0.0052
At3g15760	unknown protein	2.596	1.46E-07
At3g16030	CES101 (CALLUS EXPRESSION OF RBCS 101)	1.834	2.82E-08
At3g16050	ATPDX1.2 (ARABIDOPSIS THALIANA PYRIDOXINE BIOSYNTHESIS 1.2); Encodes a protein wit	3.522	9.14E-11
At3g16510	Calcium-dependent lipid-binding (CaLB domain) family protein	2.32	9.12E-08
At3g16720	ATL2 (TOXICOS EN LEVADURA 2); RING-H2 protein induced after exposure to chitin or inactivated	3.319	7.08E-09
At3g17110	pseudogene, glycine-rich protein	2.079	1.90E-06
At3g17260	hAT-like transposase family (hobo/Ac/Tam3), has a 2.6e-27 P-value blast match to GB:CAA29005 OR	1.719	0.01254
At3g17330	ECT6 (evolutionarily conserved C-terminal region 6)	1.728	2.41E-08
At3g17400	F-box family protein	2.887	3.30E-09
At3g17611	ATRBL14 (RHOMBOID-like protein 14)	2.916	3.62E-10
At3g17700	ATCNGC20 (CYCLIC NUCLEOTIDE-GATED CHANNEL 20); cyclic nucleotide-binding transporter	1.209	1.36E-06
At3g17920	Outer arm dynein light chain 1 protein	1.091	9.19E-08
At3g18295	Protein of unknown function (DUF1639)	1.169	4.91E-06
At3g18320	F-box and associated interaction domains-containing protein	-1.381	1.41E-06
At3g18690	MKS1 (MAP kinase substrate 1); Encodes a nuclear-localized member of a plant specific gene family in	1.595	1.04E-07
At3g18710	ATPUB29 (ARABIDOPSIS THALIANA PLANT U-BOX 29); Encodes a protein containing a U-box an	2.443	3.03E-08
At3g18773	RING/U-box superfamily protein	-1.057	6.58E-05
At3g18950	Transducin/WD40 repeat-like superfamily protein	2.495	7.32E-08
At3g19010	2-oxoglutarate (2OG) and Fe(II)-dependent oxygenase superfamily protein	1.661	3.92E-07
At3g19380	PUB25 (plant U-box 25)	1.353	2.57E-05
At3g19580	AZF2 (zinc-finger protein 2); Encodes zinc finger protein. mRNA levels are upregulated in response to	1.387	0.0009
At3g19850	Phototropic-responsive NPH3 family protein	-1.212	1.81E-06
At3g19970	alpha/beta-Hydrolases superfamily protein	1.233	1.28E-05
At3g20180	Copper transport protein family	3.097	1.75E-08
At3g20395	RING/U-box superfamily protein	-1.13	0.0091

At3g21070	ATNADK-1 (NAD KINASE 1); Encodes a protein with NAD(H) kinase activity.	2.138	8.90E-09
At3g21150	BBX32 (B-box 32); Encodes a protein with a B-box domain predicted to act as a transcription factor. E:	2.651	4.00E-05
At3g21220	ATMEK5 (ARABIDOPSIS THALIANA MITOGEN-ACTIVATED PROTEIN KINASE KINASE 5); E	1.425	5.52E-08
At3g21700	SGP2 (Ras-related small GTP-binding family protein); Monomeric G protein. Expressed in root epiderr	1.451	9.37E-07
At3g21780	UGT71B6 (UDP-glucosyl transferase 71B6); Encodes a protein with UDP-glucosyl transferase activity	2.55	2.54E-08
At3g21781	other RNA; Potential natural antisense gene, locus overlaps with AT3G21780	3.075	1.17E-09
At3g21900	Receptor-like protein kinase-related family protein	1.395	0.00165
At3g21920	Domain of unknown function (DUF26)	1.351	6.72E-05
At3g21930	Domain of unknown function (DUF26)	1.375	0.00012
At3g22060	Receptor-like protein kinase-related family protein; contains Pfam profile: PF01657 Domain of unknow	1.319	0.00044
At3g22090	unknown protein	1.257	1.42E-06
At3g22104	Phototropic-responsive NPH3 family protein	-1.015	0.0001
At3g22160	VQ motif-containing protein	1.964	2.76E-06
At3g22370	AOX1A (alternative oxidase 1A); Encodes AOX1a, an isoform of alternative oxidase that is expressed i	1.183	0.0004
At3g22886	MIR167A (microRNA167A); Encodes a microRNA that targets ARF family members ARF6 and ARF8	1.791	2.22E-07
At3g22910	ATPase E1-E2 type family protein / haloacid dehalogenase-like hydrolase family protein	3.098	0.00132
At3g22930	CML11 (calmodulin-like 11); Encodes a calmodulin-like protein.	2.023	3.80E-07
At3g23030	IAA2 (indole-3-acetic acid inducible 2); auxin inducible gene expressed in the nucleus	-1.227	3.54E-06
At3g23220	Integrase-type DNA-binding superfamily protein; encodes a member of the ERF (ethylene response fact	2.132	3.46E-08
At3g23230	Integrase-type DNA-binding superfamily protein; encodes a member of the ERF (ethylene response fact	6.262	6.47E-10
At3g23240	ATERF1 (ETHYLENE RESPONSE FACTOR 1); encodes a member of the ERF (ethylene response fac	4.71	1.41E-09
At3g23250	ATMYB15 (MYB DOMAIN PROTEIN 15); Member of the R2R3 factor gene family.	2.889	3.56E-08
At3g24100	Uncharacterised protein family SERF	1.365	0.0008
At3g24140	FMA (FAMA); Encodes a basic helix-loop-helix transcription factor whose activity is required to prom	-1.058	2.01E-05
At3g24500	MBF1C (multiprotein bridging factor 1C); One of three genes in A. thaliana encoding multiprotein brid	3.627	1.25E-09
At3g25013	Synaptobrevin family protein	1.08	3.84E-06
At3g25230	FKBP62 (FK506 BINDING PROTEIN 62); Encodes a a high molecular weight member of the FK506 b	2.095	5.54E-09
At3g25240	Protein of unknown function (DUF506)	1.384	0.0286
At3g25250	OXI1 (oxidative signal-inducible1); Arabidopsis protein kinase	6.359	1.14E-11
At3g25590	unknown protein	-1.267	2.83E-07
At3g25600	Calcium-binding EF-hand family protein	2.839	6.33E-09
At3g25610	ATPase E1-E2 type family protein / haloacid dehalogenase-like hydrolase family protein	3.349	2.45E-09
At3g25710	BHLH32 (basic helix-loop-helix 32); Encodes a basic helix–loop–helix transcription factor that is expr	-1.481	9.53E-07
At3g25717	DVL6 (DEVIL 6)	-2.387	6.91E-10
At3g25780	AOC3 (allene oxide cyclase 3); Encodes allene oxide cyclase, one of the enzymes involved in jasmonic	1.536	0.0002
At3g25870	unknown protein	-1.308	2.37E-08
At3g26020	Protein phosphatase 2A regulatory B subunit family protein; Encodes protein phosphatase 2A (PP2A) E	1.124	1.06E-06
At3g26290	CYP71B26 (cytochrome P450, family 71, subfamily B, polypeptide 26); putative cytochrome P450	-1.384	8.36E-06
At3g26470	Powdery mildew resistance protein, RPW8 domain	1.477	0.00606
At3g26500	PIRL2 (plant intracellular ras group-related LRR 2); Encodes PIRL2, a member of the Plant Intracellula	2.347	1.47E-09
At3g26680	ATSNM1 (SENSITIVE TO NITROGEN MUSTARD 1); involved in a SNM-dependent recombinationa	1.351	1.56E-07
At3g26812	MIR169I (microRNA169I); Encodes a microRNA that targets several HAP2 family members. MicroRN	1.792	2.70E-07
At3g26816	MIR169L (microRNA169L); Encodes a microRNA that targets several HAP2 family members. MicroR	1.11	0.00038
At3g26819	MIR169N (microRNA169N); Encodes a microRNA that targets several HAP2 family members. MicroF	1.098	1.81E-05
At3g26910	hydroxyproline-rich glycoprotein family protein	2.805	2.82E-08
At3g26980	MUB4 (membrane-anchored ubiquitin-fold protein 4 precursor)	1.458	6.95E-09
At3g27027	Protein of unknown function (DUF 3339)	-1.194	1.08E-07
At3g27030	unknown protein	-1.173	3.79E-07
At3g27997	expressed protein; pseudogene of unknown protein	1.235	0.00017
At3g28210	SAP12 (STRESS-ASSOCIATED PROTEIN 12); Encodes a putative zinc finger protein (PMZ).	4.526	6.82E-08
At3g28340	GATL10 (galacturonosyltransferase-like 10); Encodes a protein with putative galacturonosyltransferase	4.242	3.94E-09
At3g28420	Putative membrane lipoprotein	-1.68	8.41E-07
At3g28450	Leucine-rich repeat protein kinase family protein	1.168	1.14E-07
At3g28580	P-loop containing nucleoside triphosphate hydrolases superfamily protein	2.347	0.00157
At3g28740	CYP81D1 (Cytochrome P450 superfamily protein); Encodes a member of the cytochrome p450 family.	3.008	1.91E-08
At3g28850	Glutaredoxin family protein	1.851	1.78E-09
At3g28960	Transmembrane amino acid transporter family protein	-1.013	1.86E-05
At3g29000	Calcium-binding EF-hand family protein	5.476	1.36E-11
At3g29590	AT5MAT (HXXXD-type acyl-transferase family protein); At3g29590 (At5MAT) encodes a malonyl-Co	-1.198	0.01193
At3g29970	B12D protein	-2.143	0.00265
At3g30180	BR6OX2 (brassinosteroid-6-oxidase 2); Encodes a cytochrome p450 enzyme that catalyzes the last reac	-1.157	5.82E-06
At3g42800	unknown protein	-1.49	3.99E-05
At3g43250	Family of unknown function (DUF572)	1.869	2.85E-05
At3g44120	F-box and associated interaction domains-containing protein	-1.179	2.78E-06
At3g44190	FAD/NAD(P)-binding oxidoreductase family protein	1.407	1.22E-06
At3g44260	Polynucleotidyl transferase, ribonuclease H-like superfamily protein	2.529	1.24E-08
At3g44300	AtNIT2 (nitrilase 2); Encodes an enzyme that catalyzes the hydrolysis of indole-3-acetonitrile (IAN) to	-1.904	0.00141

At3g44350	anac061 (NAC domain containing protein 61)	1.563	0.00088
At3g44400	Disease resistance protein (TIR-NBS-LRR class) family	1.565	3.85E-06
At3g44480	RPP1 (recognition of peronospora parasitica 1); Encodes a TIR-NB-LRR R-protein RPP1 that confers re	1.036	0.0005
At3g44630	Disease resistance protein (TIR-NBS-LRR class) family	1.834	6.77E-07
At3g44670	Disease resistance protein (TIR-NBS-LRR class) family	1.347	4.79E-06
At3g44720	ADT4 (arogenate dehydratase 4); Encodes a plastid-localized arogenate dehydratase involved in phenyla	1.753	1.18E-06
At3g45638	other RNA; Unknown gene	1.372	3.82E-08
At3g45640	ATMPK3 (mitogen-activated protein kinase 3); Encodes a mitogen-activated kinase whose mRNA leve	2.815	9.34E-08
At3g45940	Glycosyl hydrolases family 31 protein	-1.173	0.00024
At3g46070	C2H2-type zinc finger family protein	1.69	2.44E-05
At3g46080	C2H2-type zinc finger family protein	4.817	2.03E-05
At3g46090	ZAT7 (C2H2 and C2HC zinc fingers superfamily protein)	5.446	1.29E-06
At3g46110	LOCATED IN: plasma membrane (Domain of unknown function (DUF966))	2.367	5.02E-08
At3g46130	ATMYB48 (myb domain protein 48); Encodes a putative transcription factor (MYB48) that functions to	-1.629	6.84E-07
At3g46200	aTNUDT9 (nudix hydrolase homolog 9)	1.019	3.42E-07
At3g46230	ATHSP17.4 (ARABIDOPSIS THALIANA HEAT SHOCK PROTEIN 17.4); member of the class I sma	6.502	5.98E-10
At3g46280	protein kinase-related	-1.809	0.00567
At3g46600	GRAS family transcription factor	1.468	1.70E-07
At3g46620	zinc finger (C3HC4-type RING finger) family protein	2.978	6.47E-10
At3g46930	Protein kinase superfamily protein	1.899	3.45E-08
At3g47090	Leucine-rich repeat protein kinase family protein	1.414	5.22E-05
At3g47210	Plant protein of unknown function (DUF247)	1.526	0.00037
At3g47500	CDF3 (cycling DOF factor 3); Dof-type zinc finger domain-containing protein, identical to H-protein pr	-1.209	1.90E-07
At3g47550	RING/FYVE/PHD zinc finger superfamily protein	2.082	1.76E-09
At3g47600	ATMYB94 (myb domain protein 94); Encodes a putative transcription factor (MYB94).	-1.09	6.35E-06
At3g47720	similar to RCD one 4 (SRO4); FUNCTIONS IN: NAD+ ADP-ribosyltransferase activity; INVOLVED I	-2.32	0.002
At3g47780	ATATH6 (A. THALIANA ABC2 HOMOLOG 6); member of ATH subfamily	1.687	1.35E-05
At3g47820	PUB39 (PLANT U-BOX 39)	2.053	1.44E-08
At3g48070	RING/U-box superfamily protein	1.586	7.08E-08
At3g48090	EDS1 (enhanced disease susceptibility 1); Component of R gene-mediated disease resistance in Arabido	1.394	0.0013
At3g48240	Octicosapeptide/Phox/Bem1p family protein	2.264	2.85E-09
At3g48360	BT2 (BTB and TAZ domain protein 2); encodes a protein (BT2) that is an essential component of the T	-2.671	7.61E-07
At3g48640	unknown protein	3.775	0.0003
At3g48650	pseudogene, At14a-related protein, similar to At14a (GI:11994571 and GI:11994573) (Arabidopsis thal	3.55	6.48E-06
At3g49530	ANAC062 (NAC domain containing protein 62); Transcription factor that serves as a molecular link be	2.925	1.14E-10
At3g49540	unknown protein	2.501	1.59E-07
At3g49570	LSU3 (RESPONSE TO LOW SULFUR 3)	6.436	5.62E-11
At3g49580	LSU1 (RESPONSE TO LOW SULFUR 1)	4.638	7.74E-09
At3g49810	ARM repeat superfamily protein	1.008	0.0084
At3g50010	Cysteine/Histidine-rich C1 domain family protein	-1.081	0.00525
At3g50060	MYB77 (myb domain protein 77); Encodes a member of the R2R3 transcription factor gene family. Exp	2.628	1.10E-06
At3g50260	CEJ1 (cooperatively regulated by ethylene and jasmonate 1); Encodes a member of the DREB subfamily	2.707	7.00E-08
At3g50310	MAPKKK20 (mitogen-activated protein kinase kinase kinase 20); member of MEKK subfamily	-1.522	4.11E-07
At3g50570	hydroxyproline-rich glycoprotein family protein	-1.307	1.66E-06
At3g50800	unknown protein	2.842	3.30E-08
At3g50930	BCS1 (cytochrome BC1 synthesis)	3.039	1.35E-07
At3g50940	P-loop containing nucleoside triphosphate hydrolases superfamily protein	1.053	0.00011
At3g50950	ZAR1 (HOPZ-ACTIVATED RESISTANCE 1)	1.563	9.33E-06
At3g51180	Zinc finger C-x8-C-x5-C-x3-H type family protein	1.524	3.62E-06
At3g51890	Clathrin light chain protein	1.518	3.79E-07
At3g51910	AT-HSFA7A (ARABIDOPSIS THALIANA HEAT SHOCK TRANSCRIPTION FACTOR A7A); mem	3.265	5.55E-11
At3g51920	CAM9 (calmodulin 9); encodes a divergent member of calmodulin, which is an EF-hand family of Ca2+	1.197	4.75E-06
At3g52400	SYP122 (syntaxin of plants 122); syntaxin protein, involved in the negative regulation of defense pathw	4.564	3.42E-10
At3g52430	ATPAD4 (ARABIDOPSIS PHYTOALEXIN DEFICIENT 4); Encodes a lipase-like gene that is importa	1.926	6.75E-05
At3g52450	PUB22 (plant U-box 22); Encodes a cytoplasmically localized U-box domain E3 ubiquitin ligase protein	1.668	7.47E-06
At3g52520	unknown protein	1.343	5.70E-05
At3g52800	A20/AN1-like zinc finger family protein	1.752	1.28E-06
At3g53810	Concanavalin A-like lectin protein kinase family protein	2.726	7.81E-10
At3g53830	Regulator of chromosome condensation (RCC1) family protein	1.116	1.02E-05
At3g54100	O-fucosyltransferase family protein	1.182	1.65E-07
At3g54150	S-adenosyl-L-methionine-dependent methyltransferases superfamily protein	4.445	1.07E-06
At3g54160	RNI-like superfamily protein	1.493	3.19E-07
At3g54200	Late embryogenesis abundant (LEA) hydroxyproline-rich glycoprotein family	1.291	1.96E-06
At3g54810	BME3 (BLUE MICROPYLAR END 3); Encodes a protein containing a GATA type zinc finger domain	1.308	5.07E-06
At3g55240	Plant protein 1589 of unknown function; Overexpression leads to PEL (Pseudo-Etiolation in Light) phen	-1.806	3.73E-05
At3g55580	Regulator of chromosome condensation (RCC1) family protein	1.351	1.98E-06
At3g55840	Hs1pro-1 protein	4.653	6.14E-10

At3g55880	SUE4 (sulphate utilization efficiency 4); A gain-of-function mutant of SUE4 exhibited improved low su	1.046	0.0003
At3g55980	SZF1 (salt-inducible zinc finger 1)	2.041	0.0171
At3g56200	Transmembrane amino acid transporter family protein; Encodes a putative amino acid transporter.	2.744	1.57E-09
At3g56250	unknown protein	1.408	3.88E-07
At3g56400	ATWRKY70 (ARABIDOPSIS THALIANA WRKY DNA-BINDING PROTEIN 70); member of WRKY	1.102	0.01082
At3g56410	Protein of unknown function (DUF3133)	1.531	0.0002
At3g56710	SIB1 (sigma factor binding protein 1); Sig1 binding protein; interacts with Sig1R4. As well as Sig1, Sib	5.203	1.47E-10
At3g56880	VQ motif-containing protein	1.959	1.28E-07
At3g57210	Protein of unknown function (DUF626)	1.223	0.0006
At3g57450	unknown protein	2.465	9.79E-09
At3g57530	CPK32 (calcium-dependent protein kinase 32); Calcium-dependent Protein Kinase. ABA signaling com	3.179	3.90E-10
At3g57630	exostosin family protein	1.049	7.06E-07
At3g57640	Protein kinase superfamily protein	2.127	1.84E-08
At3g57730	Protein kinase superfamily protein	2.544	4.35E-09
At3g57740	Protein kinase superfamily protein	3.437	7.39E-11
At3g57750	Protein kinase superfamily protein	1.482	1.98E-07
At3g57880	Calcium-dependent lipid-binding (CaLB domain) plant phosphoribosyltransferase family protein	1.408	6.42E-07
At3g58490	Phosphatidic acid phosphatase (PAP2) family protein	1.245	1.86E-06
At3g59080	Eukaryotic aspartyl protease family protein	2.75	3.01E-07
At3g59150	F-box/RNI-like superfamily protein	1.044	1.22E-05
At3g59310	Eukaryotic protein of unknown function (DUF914)	1.106	0.0001
At3g59520	ATRBL13 (RHOMBOID-like protein 13)	2.151	9.39E-09
At3g59530	LAP3 (LESS ADHERENT POLLEN 3)	1.982	1.53E-08
At3g59700	ATHLECRK (lectin-receptor kinase); member of Receptor kinase-like protein family	2.077	6.84E-09
At3g59880	unknown protein	2.018	6.31E-07
At3g59940	Galactose oxidase/kelch repeat superfamily protein	-1.448	1.19E-06
At3g60110	DNA-binding bromodomain-containing protein	1.036	0.0004
At3g60120	BGLU27 (beta glucosidase 27)	1.906	0.01812
At3g60290	2-oxoglutarate (2OG) and Fe(II)-dependent oxygenase superfamily protein	-1.022	0.00104
At3g61190	BAP1 (BON association protein 1); Encodes a protein with a C2 domain that binds to BON1 in yeast tw	5.38	4.76E-08
At3g61250	AtMYB17 (myb domain protein 17); Member of the R2R3 factor gene family.	-1.261	0.00287
At3g61900	SAUR-like auxin-responsive protein family	-1.021	6.14E-05
At3g61950	basic helix-loop-helix (bHLH) DNA-binding superfamily protein	-1.116	3.94E-05
At3g62150	PGP21 (P-glycoprotein 21)	1.411	1.41E-07
At3g62260	Protein phosphatase 2C family protein	3.283	4.08E-10
At3g62630	Protein of unknown function (DUF1645)	-1.079	4.51E-06
At3g62720	XT1 (xylosyltransferase 1); Encodes a protein with xylosyltransferase activity, which is specific for UD	1.362	2.89E-06
At3g62780	Calcium-dependent lipid-binding (CaLB domain) family protein	1.127	9.76E-05
At3g62930	Thioredoxin superfamily protein	-1.704	1.15E-06
At3g63110	ATIPT3 (isopentenyltransferase 3); Encodes cytokinin synthase involved in cytokinin biosynthesis. IPT	-1.353	2.17E-05
At3g63210	MARD1 (MEDIATOR OF ABA-REGULATED DORMANCY 1); encodes a novel zinc-finger protein w	-1.719	4.34E-08
At3g63310	BIL4 (BRZ-INSENSITIVE-LONG HYPOCOTYLS 4); Mediates cell elongation in brassinosteroid sign	1.342	2.36E-07
At3g63350	HSFA7B (HEAT SHOCK TRANSCRIPTION FACTOR A7B); member of Heat Stress Transcription F	5.957	1.36E-10
At4g00342	unknown protein	2.727	0.00012
At4g00870	basic helix-loop-helix (bHLH) DNA-binding superfamily protein	-1.002	0.00013
At4g00940	Dof-type zinc finger DNA-binding family protein	2.003	3.26E-08
At4g00970	CRK41 (cysteine-rich RLK (RECEPTOR-like protein kinase) 41); Encodes a cysteine-rich receptor-like	2.683	1.08E-07
At4g01010	ATCNGC13 (CYCLIC NUCLEOTIDE-GATED CHANNEL 13); member of Cyclic nucleotide gated ch	3.029	8.20E-09
At4g01250	WRKY22 (WRKY family transcription factor); member of WRKY Transcription Factor; Group II-e	1.316	9.87E-05
At4g01540	ANAC068 (Arabidopsis NAC domain containing protein 68); Encodes a membrane-bound NAC (for N	1.359	4.68E-05
At4g01550	anac069 (NAC domain containing protein 69)	1.017	4.54E-05
At4g01870	tolB protein-related	1.877	9.48E-06
At4g01950	GPAT3 (glycerol-3-phosphate acyltransferase 3); Encodes a member of a family of proteins with glycer	1.336	1.70E-05
At4g02200	Drought-responsive family protein	2.611	2.42E-09
At4g02330	ATPMEPCRB (Plant invertase/pectin methylesterase inhibitor superfamily)	1.506	0.0003
At4g02380	AtLEA5 (Arabidopsis thaliana late embryogenensis abundant like 5); Encodes AtLEA5 (late embryogen	1.157	0.0002
At4g02410	Concanavalin A-like lectin protein kinase family protein	3.029	1.91E-09
At4g02425	unknown protein	2.031	4.39E-09
At4g02550	unknown protein	3.256	1.16E-11
At4g02690	Bax inhibitor-1 family protein	1.517	0.002
At4g02950	Ubiquitin family protein	1.377	4.63E-07
At4g03038	other RNA; Unknown gene	-1.41	2.98E-05
At4g03060	similar to AOP3 (2-oxoglutarate?dependent dioxygenase 3), oxidoreductase, acting on paired donors, w	-1.889	4.49E-07
At4g03292	Polynucleotidyl transferase, ribonuclease H-like superfamily protein	1.105	0.0011
At4g03450	Ankyrin repeat family protein	3.125	0.00068
At4g04020	FIB (fibrillin); Fibrillin precursor protein. The fibrillin preprotein, but not the mature protein interacts w	1.051	0.0004
At4g04293	pseudogene, similar to P0703B11.15, blastp match of 38% identity and 2.2e-25 P-value to GP|1884482	-1.88	2.52E-06

At4g04540	CRK39 (cysteine-rich RLK (RECEPTOR-like protein kinase) 39); Encodes a cysteine-rich receptor-like	1.096	6.72E-06
At4g04610	APR1 (APS reductase 1); Encodes a protein disulfide isomerase-like (PDIL) protein, a member of a mu	3.925	1.88E-10
At4g05010	F-box family protein	1.352	6.77E-08
At4g08230	glycine-rich protein	1.029	2.27E-05
At4g08320	Tetratricopeptide repeat (TPR)-like superfamily protein	1.242	1.60E-08
At4g08500	MEKK1 (MAPK/ERK kinase kinase 1); Encodes a member of the A1 subgroup of the MEKK (MAPK/	1.304	1.28E-07
At4g08555	unknown protein	2.31	2.19E-05
At4g08770	Peroxidase superfamily protein	-1.247	0.02678
At4g08910	unknown protein	-1.201	0.00073
At4g09150	T-complex protein 11	2.76	1.02E-09
At4g09570	CPK4 (calcium-dependent protein kinase 4); Encodes a member of Calcium Dependent Protein Kinase	1.907	1.94E-09
At4g09820	TT8 (TRANSPARENT TESTA 8); TT8 is a regulation factor that acts in a concerted action with TT1, F	-1.098	0.00417
At4g10250	ATHSP22.0 (HSP20-like chaperones superfamily protein); Columbia endomembrane-localized small he	6.455	7.08E-08
At4g10265	Wound-responsive family protein	-1.039	0.0255
At4g10270	Wound-responsive family protein	-1.364	0.03177
At4g10910	unknown protein	-1.578	7.74E-05
At4g11000	Ankyrin repeat family protein	2.364	2.12E-08
At4g11280	ACS6 (1-aminocyclopropane-1-carboxylic acid (acc) synthase 6); encodes a a member of the 1-aminocy	3.487	3.97E-08
At4g11330	ATMPK5 (MAP kinase 5); MAP kinase	1.653	2.40E-08
At4g11350	Protein of unknown function (DUF604)	1.203	5.92E-05
At4g11360	RHA1B (RING-H2 finger A1B); Encodes a putative RING-H2 finger protein RHA1b.	1.163	4.24E-06
At4g11370	RHA1A (RING-H2 finger A1A); Encodes a putative RING-H2 finger protein RHA1a.	2.411	1.46E-08
At4g11460	CRK30 (cysteine-rich RLK (RECEPTOR-like protein kinase) 30); Encodes a cysteine-rich receptor-like	-1.309	5.17E-05
At4g11470	CRK31 (cysteine-rich RLK (RECEPTOR-like protein kinase) 31); Encodes a cysteine-rich receptor-like	1.886	1.94E-07
At4g11500	pseudogene, similar to putative receptor-like serine-threonine protein kinase, blastp match of 59% ident	1.114	0.00114
At4g11660	HSFB2B (HEAT SHOCK TRANSCRIPTION FACTOR B2B); member of Heat Stress Transcription Fa	2.808	5.55E-11
At4g11740	SAY1 (Ubiquitin-like superfamily protein); Isolated as a suppressor of a dominant mutant in the Ara4 g	1.042	6.34E-07
At4g11850	MEE54 (maternal effect embryo arrest 54); phospholipase D (gamma)	1.559	5.53E-08
At4g12120	SEC1B (Sec1/munc18-like (SM) proteins superfamily); member of KEULE Gene Family	2.175	3.77E-09
At4g12334	Cytochrome P450 superfamily protein	1.462	3.47E-06
At4g12400	stress-inducible protein, putative	4.185	5.52E-10
At4g12410	SAUR-like auxin-responsive protein family	3.382	5.31E-10
At4g12470	AZI1 (azelaic acid induced 1); Encodes AZI1 (AZELAIC ACID INDUCED 1). Involved in the priming	-1.504	0.0271
At4g12480	pEARLI 1 (Bifunctional inhibitor/lipid-transfer protein/seed storage 2S albumin superfamily protein); a	-2.886	0.0035
At4g12490	Bifunctional inhibitor/lipid-transfer protein/seed storage 2S albumin superfamily protein	-3.419	0.02493
At4g12500	Bifunctional inhibitor/lipid-transfer protein/seed storage 2S albumin superfamily protein	-2.922	0.00466
At4g12710	ARM repeat superfamily protein	1.032	0.002
At4g12720	AtNUDT7 (Arabidopsis thaliana Nudix hydrolase homolog 7); Encodes a protein with ADP-ribose hydr	2.793	7.08E-08
At4g13180	NAD(P)-binding Rossmann-fold superfamily protein	1.914	5.39E-08
At4g13260	YUC2 (YUCCA2)	-1.399	1.84E-08
At4g13395	DVL10 (DEVIL 10)	2.167	6.27E-05
At4g13495	other RNA; Unknown gene	-1.026	1.50E-05
At4g13820	Leucine-rich repeat (LRR) family protein	1.336	7.27E-07
At4g14090	UDP-Glycosyltransferase superfamily protein; The At4g14090 encodes a anthocyanidin 5-O-glucosyltra	-1.127	0.02095
At4g14220	RHF1A (RING-H2 group F1A); encodes a RING-type E3 ubiquitin ligase implicated in gametogenesis.	1.083	2.62E-05
At4g14365	XBAT34 (XB3 ortholog 4 in Arabidopsis thaliana)	2.777	2.03E-06
At4g14370	Disease resistance protein (TIR-NBS-LRR class) family	3.145	0.0001
At4g14450	unknown protein	3.052	7.12E-09
At4g14480	Protein kinase superfamily protein	-1.834	5.52E-07
At4g14560	AXR5 (AUXIN RESISTANT 5); auxin (indole-3-acetic acid) induced gene (IAA1) encoding a short-liv	-1.048	7.39E-05
At4g14680	APS3 (Pseudouridine synthase/archaeosine transglycosylase-like family protein); ATP sulfurylase	1.571	8.32E-08
At4g14980	Cysteine/Histidine-rich C1 domain family protein	-3.567	0.0297
At4g15248	B-box type zinc finger family protein	3.176	8.93E-06
At4g15420	Ubiquitin fusion degradation UFD1 family protein	2.224	1.60E-05
At4g15500	UGT84A4 (UDP-Glycosyltransferase superfamily protein); Encodes a protein that might have sinapic a	-1.191	0.00013
At4g15660	Thioredoxin superfamily protein	-1.026	0.00147
At4g15670	Thioredoxin superfamily protein	-1.567	4.36E-06
At4g15680	Thioredoxin superfamily protein	-1.534	5.66E-05
At4g15700	Thioredoxin superfamily protein	-1.26	3.43E-05
At4g15975	RING/U-box superfamily protein	3.89	7.14E-10
At4g16447	unknown protein	-1.216	2.10E-07
At4g16460	unknown protein	1.363	2.89E-05
At4g16680	P-loop containing nucleoside triphosphate hydrolases superfamily protein	1.452	0.00031
At4g16820	PLA-I{beta]2 (phospholipase A I beta 2); Encodes a lipase that hydrolyzes phosphatidylcholine, glycoli	3.692	6.58E-10
At4g16860	RPP4 (recognition of peronospora parasitica 4); Confers resistance to Peronospora parasitica. RPP4 is c	1.1	1.40E-05
At4g16960	Disease resistance protein (TIR-NBS-LRR class) family	1.094	8.84E-06
At4g17230	SCL13 (SCARECROW-like 13); Encodes a scarecrow-like protein (SCL13). Member of GRAS gene fa	1.827	2.92E-07

At4g17250	unknown protein	3.698	8.66E-11
At4g17490	ATERF6 (ethylene responsive element binding factor 6); Encodes a member of the ERF (ethylene respo	4.288	1.85E-09
At4g17500	ATERF-1 (ethylene responsive element binding factor 1); Encodes a member of the ERF (ethylene resp	2.951	4.68E-07
At4g17615	ATCBL1 (ARABIDOPSIS THALIANA CALCINEURIN B-LIKE PROTEIN); Member of AtCBL (Calc	1.626	4.26E-09
At4g17900	PLATZ transcription factor family protein	2.641	2.13E-10
At4g18010	5PTASE2 (myo-inositol polyphosphate 5-phosphatase 2); Encodes an inositol polyphosphate 5-phospha	1.065	2.94E-06
At4g18200	NA	2.837	2.20E-08
At4g18250	receptor serine/threonine kinase, putative	2.608	1.86E-07
At4g18450	Integrase-type DNA-binding superfamily protein; encodes a member of the ERF (ethylene response fact	1.547	1.21E-07
At4g18880	AT-HSFA4A (ARABIDOPSIS THALIANA HEAT SHOCK TRANSCRIPTION FACTOR A4A); mem	2.901	1.94E-10
At4g18950	Integrin-linked protein kinase family	1.353	1.10E-05
At4g19170	CCD4 (carotenoid cleavage dioxygenase 4); chloroplast-targeted member of a family of enzymes simila	-1.22	0.00038
At4g19515	NA	3.417	8.03E-08
At4g19520	disease resistance protein (TIR-NBS-LRR class) family	3.921	1.81E-08
At4g19700	RING (SBP (S-ribonuclease binding protein) family protein)	-1.943	4.47E-10
At4g19720	Glycosyl hydrolase family protein with chitinase insertion domain	-1.592	0.03681
At4g20000	VQ motif-containing protein	1.061	0.01254
At4g20780	CML42 (calmodulin like 42); Calcium sensor involved in trichome branching.	3.438	4.05E-09
At4g20820	FAD-binding Berberine family protein	1.607	3.45E-07
At4g20860	FAD-binding Berberine family protein	2.073	3.18E-08
At4g21120	AAT1 (amino acid transporter 1); Encodes a member of the cationic amino acid transporter (CAT) subf	1.185	0.00149
At4g21320	HSA32 (HEAT-STRESS-ASSOCIATED 32); Encodes heat-stress-associated 32-kD protein. Up-regulat	3.704	1.53E-09
At4g21323	Subtilase family protein	4.655	3.21E-11
At4g21390	B120 (S-locus lectin protein kinase family protein)	4.408	2.64E-10
At4g21700	Protein of unknown function (DUF2921)	1.323	6.93E-09
At4g21920	unknown protein	3.144	4.91E-06
At4g21990	APR3 (APS reductase 3); Encodes a protein disulfide isomerase-like (PDIL) protein, a member of a mu	4.459	4.23E-11
At4g22470	protease inhibitor/seed storage/lipid transfer protein (LTP) family protein	-2.422	0.00067
At4g22530	S-adenosyl-L-methionine-dependent methyltransferases superfamily protein	1.511	1.54E-06
At4g22560	unknown protein	-1.169	7.71E-05
At4g22690	CYP706A1 (cytochrome P450, family 706, subfamily A, polypeptide 1); member of CYP706A	1.068	0.0001
At4g22710	CYP706A2 (cytochrome P450, family 706, subfamily A, polypeptide 2); member of CYP706A	1.544	2.40E-07
At4g22740	glycine-rich protein	1.164	5.20E-08
At4g22780	ACR7 (ACT domain repeat 7); Member of a family of ACT domain containing proteins . ACT domains	1.113	0.002
At4g23170	CRK9 (CYSTEINE-RICH RLK (RECEPTOR-LIKE PROTEIN KINASE) 9); Induced in response to Sa	1.93	3.94E-06
At4g23180	CRK10 (cysteine-rich RLK (RECEPTOR-like protein kinase) 10); Encodes a receptor-like protein kinas	2.219	3.14E-07
At4g23190	AT-RLK3 (RECEPTOR LIKE PROTEIN KINASE 3); Encodes putative receptor-like protein kinase tha	3.597	5.16E-10
At4g23200	CRK12 (cysteine-rich RLK (RECEPTOR-like protein kinase) 12); Encodes a cysteine-rich receptor-like	1.175	0.00464
At4g23215	pseudogene of cysteine-rich receptor-like protein kinase family protein; pseudogene of cysteine-rich rec	2.796	1.96E-07
At4g23220	CRK14 (cysteine-rich RLK (RECEPTOR-like protein kinase) 14); Encodes a cysteine-rich receptor-like	2.452	1.14E-07
At4g23270	CRK19 (cysteine-rich RLK (RECEPTOR-like protein kinase) 19); Encodes a cysteine-rich receptor-like	1.995	1.46E-07
At4g23280	CRK20 (cysteine-rich RLK (RECEPTOR-like protein kinase) 20); Encodes a cysteine-rich receptor-like	1.258	2.67E-05
At4g23300	CRK22 (cysteine-rich RLK (RECEPTOR-like protein kinase) 22); Encodes a cysteine-rich receptor-like	-1.2	2.21E-05
At4g23493	unknown protein	5.676	1.91E-11
At4g23550	WRKY29 (WRKY family transcription factor); Encodes WRKY DNA-binding protein 29 (WRKY29).	-1.004	7.52E-06
At4g23570	SGT1A (phosphatase-related); Closely related to SGT1B, may function in SCF(TIR1) mediated protein	1.698	4.00E-08
At4g23610	Late embryogenesis abundant (LEA) hydroxyproline-rich glycoprotein family	2.113	3.19E-05
At4g23700	ATCHX17 (cation/H+ exchanger 17); member of Putative Na+/H+ antiporter family	1.068	0.00478
At4g23810	WRKY53 (WRKY family transcription factor); member of WRKY Transcription Factor; Group III	2.708	1.30E-07
At4g23880	unknown protein	1.008	3.91E-07
At4g23885	unknown protein	1.464	4.59E-07
At4g23980	ARF9 (auxin response factor 9); Encodes auxin response factor 9 (ARF9).	-1.286	0.01979
At4g24120	ATYSL1 (YELLOW STRIPE LIKE 1); Member of a small family of oligopeptide transporters similar to	-1.094	0.0011
At4g24160	alpha/beta-Hydrolases superfamily protein; Encodes a soluble lysophosphatidic acid acyltransferase wit	3.327	1.01E-09
At4g24380	INVOLVED IN: 10-formyltetrahydrofolate biosynthetic process, folic acid and derivative biosynthetic p	4.422	6.66E-10
At4g24410	unknown protein	2.313	2.00E-06
At4g24415	MIR824A (microRNA824A); Encodes a microRNA that targets AGL16. MicroRNAs are regulatory RN	3.76	2.29E-10
At4g24570	DIC2 (dicarboxylate carrier 2); Encodes one of the mitochondrial dicarboxylate carriers (DIC): DIC1 (A	5.489	2.35E-09
At4g25030	unknown protein	1.159	1.58E-06
At4g25200	ATHSP23.6-MITO (mitochondrion-localized small heat shock protein 23.6); AtHSP23.6-mito mRNA, r	7.036	5.88E-09
At4g25350	SHB1 (SHORT HYPOCOTYL UNDER BLUE1); SHB1 encodes a nuclear and cytosolic protein that ha	3.168	3.02E-09
At4g25380	SAP10 (stress-associated protein 10)	5.81	1.79E-10
At4g25420	ATGA20OX1 (ARABIDOPSIS THALIANA GIBBERELLIN 20-OXIDASE 1); Encodes gibberellin 20	-2.167	2.71E-08
At4g25470	CBF2 (C-repeat/DRE binding factor 2); Encodes a member of the DREB subfamily A-1 of ERF/AP2 tra	1.019	9.18E-05
At4g25640	ATDTX35 (detoxifying efflux carrier 35); Encodes a multidrug and toxin efflux family transporter. Invo	1.062	0.0007
At4g25810	XTH23 (xyloglucan endotransglucosylase/hydrolase 23); xyloglucan endotransglycosylase-related prote	2.038	0.00137
At4g25820	XTH14 (xyloglucan endotransglucosylase/hydrolase 14); Encodes a xyloglucan endotransglycosylase w	2.382	0.00234

At4g26010	Peroxidase superfamily protein	1.332	0.04582
At4g26090	RPS2 (RESISTANT TO P. SYRINGAE 2); Encodes a plasma membrane protein with leucine-rich repe	1.134	0.0001
At4g26120	Ankyrin repeat family protein / BTB/POZ domain-containing protein	1.214	3.72E-06
At4g26140	BGAL12 (beta-galactosidase 12); putative beta-galactosidase	1.343	3.44E-07
At4g26200	ACS7 (1-amino-cyclopropane-1-carboxylate synthase 7); Member of a family of proteins in Arabidopsis	2.495	0.00017
At4g26270	PFK3 (phosphofructokinase 3)	2.019	1.54E-06
At4g26460	S-adenosyl-L-methionine-dependent methyltransferases superfamily protein	-1.483	0.00453
At4g26580	RING/U-box superfamily protein	1.738	2.20E-06
At4g26990	unknown protein	2.028	4.55E-07
At4g27280	Calcium-binding EF-hand family protein	3.679	4.09E-07
At4g27350	Protein of unknown function (DUF1223)	1.396	1.33E-08
At4g27360	Dynein light chain type 1 family protein	2.893	8.02E-07
At4g27652	unknown protein	3.857	4.00E-11
At4g27657	unknown protein	4.305	2.69E-09
At4g27670	HSP21 (heat shock protein 21); chloroplast located small heat shock protein.	7.21	6.02E-09
At4g27730	ATOPT6 (ARABIDOPSIS THALIANA OLIGOPEPTIDE TRANSPORTER 6); oligopeptide transporte	-1.03	0.00017
At4g27890	HSP20-like chaperones superfamily protein	1.807	3.05E-06
At4g27940	ATMTM1 (ARABIDOPSIS MANGANESE TRACKING FACTOR FOR MITOCHONDRIAL SOD2)	1.175	2.00E-07
At4g27970	SLAH2 (SLAC1 homologue 2); Encodes a protein with ten predicted transmembrane helices. The SLAl	1.972	7.34E-08
At4g27980	Domain of unknown function (DUF3444)	2.33	1.52E-08
At4g28085	unknown protein	1.431	0.0025
At4g28240	Wound-responsive family protein	-1.524	1.58E-06
At4g28350	Concanavalin A-like lectin protein kinase family protein	4.286	5.55E-11
At4g28390	AAC3 (ADP/ATP carrier 3); Encodes a mitochondrial ADP/ATP carrier protein. Shown in heterologous	1.575	1.08E-06
At4g28400	Protein phosphatase 2C family protein	2.089	1.09E-09
At4g28460	unknown protein	1.393	0.00069
At4g28480	DNAJ heat shock family protein	1.888	6.58E-09
At4g28490	HAE (HAESA); member of Receptor kinase-like protein family	1.165	0.00023
At4g28703	RmlC-like cupins superfamily protein	3.653	3.86E-08
At4g28720	YUC8 (YUCCA 8); Auxin biosynthetic gene regulated by RVE1. Overexpression leads to suppression	-1.534	1.38E-06
At4g29050	Concanavalin A-like lectin protein kinase family protein	2.298	1.30E-07
At4g29240	Leucine-rich repeat (LRR) family protein	-1.159	0.00145
At4g29330	DER1 (DERLIN-1)	1.373	1.09E-07
At4g29340	PRF4 (profilin 4); Profilin is a low-molecular weight, actin monomer-binding protein that regulates the	1.575	1.31E-07
At4g29740	CKX4 (cytokinin oxidase 4); It encodes a protein whose sequence is similar to cytokinin oxidase/dehyd	1.436	2.86E-07
At4g29770	unknown protein; Target of trans acting-siR480/255.	4.006	1.62E-10
At4g29780	unknown protein	5.31	6.38E-09
At4g30090	emb1353 (embryo defective 1353)	1.048	0.00044
At4g30100	P-loop containing nucleoside triphosphate hydrolases superfamily protein	1.024	7.06E-05
At4g30210	ATR2 (P450 reductase 2); Encodes NADPH-cytochrome P450 reductase that catalyzes the first oxidativ	1.663	5.54E-07
At4g30240	Syntaxin/t-SNARE family protein	1.024	4.28E-06
At4g30280	ATXTH18 (XYLOGLUCAN ENDOTRANSGLUCOSYLASE/HYDROLASE 18); Encodes a xylogluca	2.516	1.30E-06
At4g30300	ATNAP15 (ARABIDOPSIS THALIANA NON-INTRINSIC ABC PROTEIN 15); member of NAP subf	1.407	5.82E-07
At4g30390	unknown protein	1.394	5.13E-07
At4g30400	RING/U-box superfamily protein	-1.117	1.27E-06
At4g30430	TET9 (tetraspanin9); Member of TETRASPANIN family	2.878	1.01E-05
At4g30490	AFG1-like ATPase family protein	1.212	1.28E-06
At4g30510	ATATG18B (ARABIDOPSIS THALIANA HOMOLOG OF YEAST AUTOPHAGY 18 (ATG18) B)	1.399	3.48E-07
At4g30850	HHP2 (heptahelical transmembrane protein2); heptahelical transmembrane protein homologous to hum	1.969	1.26E-07
At4g31398	other RNA; Potential natural antisense gene, locus overlaps with AT4G31400	1.372	6.77E-06
At4g31500	ATR4 (ALTERED TRYPTOPHAN REGULATION 4); Encodes an oxime-metabolizing enzyme in the	-1.14	1.63E-05
At4g31550	WRKY11 (WRKY DNA-binding protein 11); member of WRKY Transcription Factor; Group II-d; neg	2.58	1.25E-08
At4g31800	ATWRKY18 (ARABIDOPSIS THALIANA WRKY DNA-BINDING PROTEIN 18); Pathogen-induced	1.451	2.37E-05
At4g31877	MIR156C (microRNA156C); Encodes a microRNA that targets several SPL family members, including	2.782	1.14E-08
At4g31950	CYP82C3 (cytochrome P450, family 82, subfamily C, polypeptide 3); member of CYP82C	2.873	4.56E-06
At4g31970	CYP82C2 (cytochrome P450, family 82, subfamily C, polypeptide 2); member of CYP82C	1.799	9.83E-06
At4g32208	heat shock protein 70 (Hsp 70) family protein	1.407	4.01E-06
At4g32280	IAA29 (indole-3-acetic acid inducible 29); Auxin inducible protein.	-1.313	0.00052
At4g32300	SD2-5 (S-domain-2 5)	1.064	1.74E-06
At4g32480	Protein of unknown function (DUF506)	1.025	0.0171
At4g32920	glycine-rich protein	-1.23	0.0456
At4g33040	Thioredoxin superfamily protein	1.56	2.93E-06
At4g33050	EDA39 (embryo sac development arrest 39)	2.376	1.06E-07
At4g33060	Cyclophilin-like peptidyl-prolyl cis-trans isomerase family protein	1.201	5.58E-07
At4g33070	Thiamine pyrophosphate dependent pyruvate decarboxylase family protein	-2.228	0.00737
At4g33430	ATSERK3 (SOMATIC EMBRYOGENESIS RECEPTOR-LIKE KINASE 3); Leu-rich receptor Serine/t	1.152	0.0002
At4g33440	Pectin lyase-like superfamily protein	2.29	6.17E-09

At4g33560	Wound-responsive family protein	-1.314	0.02731
At4g33660	unknown protein	1.656	8.11E-06
At4g33905	Peroxisomal membrane 22 kDa (Mpv17/PMP22) family protein	1.307	3.25E-07
At4g33920	Protein phosphatase 2C family protein	2.686	1.78E-09
At4g33940	RING/U-box superfamily protein	1.524	3.76E-07
At4g33985	Protein of unknown function (DUF1685)	2.758	9.19E-11
At4g34131	UGT73B3 (UDP-glucosyl transferase 73B3)	2.073	1.20E-06
At4g34135	UGT73B2 (UDP-glucosyltransferase 73B2); The At4g34135 gene encodes a flavonol 7-O-glucosyltrans	2.252	3.10E-06
At4g34138	UGT73B1 (UDP-glucosyl transferase 73B1)	1.431	1.43E-06
At4g34150	Calcium-dependent lipid-binding (CaLB domain) family protein	2.908	2.04E-08
At4g34180	Cyclase family protein	1.249	5.02E-07
At4g34300	Cupredoxin superfamily protein; Encodes protein with 14.7% glycine residues, similar to auxin respons	1.98	2.75E-06
At4g34380	Transducin/WD40 repeat-like superfamily protein	1.103	9.96E-06
At4g34390	XLG2 (extra-large GTP-binding protein 2)	2.438	2.19E-08
At4g34410	RRTF1 (redox responsive transcription factor 1); encodes a member of the ERF (ethylene response fact	1.335	0.00427
At4g34560	unknown protein	-1.091	1.45E-05
At4g34750	SAUR-like auxin-responsive protein family	-1.376	0.0002
At4g34760	SAUR-like auxin-responsive protein family	-2.294	1.36E-07
At4g34770	SAUR-like auxin-responsive protein family	-1.211	7.82E-05
At4g34790	SAUR-like auxin-responsive protein family	-1.358	1.43E-06
At4g34800	SAUR-like auxin-responsive protein family	-1.11	0.00066
At4g34950	Major facilitator superfamily protein	-1.251	4.36E-05
At4g35110	Arabidopsis phospholipase-like protein (PEARLI 4) family	2.149	3.21E-07
At4g35180	LHT7 (LYS/HIS transporter 7)	1.053	0.02598
At4g35270	Plant regulator RWP-RK family protein	-1.008	7.91E-06
At4g35480	RHA3B (RING-H2 finger A3B); Encodes a putative RING-H2 finger protein RHA3b.	1.556	7.27E-07
At4g35640	ATSERAT3.2 (serine acetyltransferase 3.2); Encodes a cytosolic serine O-acetyltransferase involved in	2.038	9.00E-07
At4g35940	unknown protein	1.704	3.65E-07
At4g36040	Chaperone DnaJ-domain superfamily protein	-1.604	1.59E-07
At4g36110	SAUR-like auxin-responsive protein family	-1.414	2.43E-05
At4g36150	Disease resistance protein (TIR-NBS-LRR class) family	1.296	4.34E-06
At4g36380	ROT3 (ROTUNDIFOLIA 3); Encodes a cytochrome P-450 gene that is involved in leaf blade expansior	-1.143	6.65E-07
At4g36500	unknown protein	2.421	1.36E-08
At4g36540	BEE2 (BR enhanced expression 2)	-1.419	2.78E-07
At4g36550	ARM repeat superfamily protein	2.181	6.62E-10
At4g36988	CPuORF49 (conserved peptide upstream open reading frame 49); Upstream open reading frames (uORI	2.837	2.82E-08
At4g36990	AT-HSFB1 (ARABIDOPSIS THALIANA CLASS B HEAT SHOCK FACTOR B1); encodes a protein	3.361	6.38E-09
At4g37260	MYB73 (myb domain protein 73); Member of the R2R3 factor gene family.	1.748	1.18E-07
At4g37290	unknown protein	5.396	2.34E-09
At4g37295	unknown protein	-1.453	9.64E-05
At4g37370	CYP81D8 (cytochrome P450, family 81, subfamily D, polypeptide 8); member of CYP81D	2.834	9.53E-08
At4g37470	alpha/beta-Hydrolases superfamily protein	-1.077	0.0023
At4g37540	LBD39 (LOB domain-containing protein 39)	-1.691	2.12E-08
At4g37730	AtbZIP7 (basic leucine-zipper 7)	1.234	5.73E-06
At4g37790	HAT22 (Homeobox-leucine zipper protein family); Encodes homeobox protein HAT22, member of the	-1.503	4.82E-07
At4g38560	Arabidopsis phospholipase-like protein (PEARLI 4) family	1.956	1.32E-06
At4g38620	ATMYB4 (myb domain protein 4); Encodes a R2R3 MYB protein which is involved in the response to	1.037	0.00012
At4g38825	SAUR-like auxin-responsive protein family	-1.912	4.04E-06
At4g38840	SAUR-like auxin-responsive protein family	-2.653	1.78E-09
At4g38850	ATSAUR15 (ARABIDOPSIS THALIANA SMALL AUXIN UPREGULATED 15); mRNA is rapidly i	-2.057	3.53E-07
At4g38860	SAUR-like auxin-responsive protein family	-2.747	2.43E-08
At4g38940	Galactose oxidase/kelch repeat superfamily protein	2.163	8.49E-09
At4g39030	EDS5 (ENHANCED DISEASE SUSCEPTIBILITY 5); Encodes an orphan multidrug and toxin extrusic	1.252	0.0031
At4g39270	Leucine-rich repeat protein kinase family protein	1.752	1.60E-07
At4g39580	Galactose oxidase/kelch repeat superfamily protein	2.379	1.07E-07
At4g39640	GGT1 (gamma-glutamyl transpeptidase 1); The gene encodes a gamma-glutamyltransferase (AKA gam	2.276	2.83E-09
At4g39670	Glycolipid transfer protein (GLTP) family protein	4.695	7.64E-07
At4g39675	unknown protein	-1.131	0.0012
At4g39780	Integrase-type DNA-binding superfamily protein; encodes a member of the DREB subfamily A-6 of ER	-1.699	1.69E-07
At4g39830	Cupredoxin superfamily protein	1.721	0.0002
At4g39890	AtRABH1c (RAB GTPase homolog H1C)	1.719	1.22E-07
At4g40020	Myosin heavy chain-related protein	1.253	0.00069
At4g40065	other RNA	-1.411	0.00019
At4g40070	RING/U-box superfamily protein	-1.399	4.51E-06
At4g40080	ENTH/ANTH/VHS superfamily protein	2.834	1.20E-08
At4g40090	AGP3 (arabinogalactan protein 3)	2.18	0.00532
At5g01060	Protein kinase protein with tetratricopeptide repeat domain	2.712	4.06E-08

At5g01100	O-fucosyltransferase family protein	3.758	1.35E-08
At5g01180	ATPTR5 (ARABIDOPSIS THALIANA PEPTIDE TRANSPORTER 5); Encodes a dipeptide transporte	3.483	2.52E-10
At5g01320	Thiamine pyrophosphate dependent pyruvate decarboxylase family protein	-1.257	0.00292
At5g01380	Homeodomain-like superfamily protein	1.424	0.00194
At5g01540	LECRKA4.1 (lectin receptor kinase a4.1); Encodes LecRKA4.1, a member of the lectin receptor kinase	2.982	5.78E-08
At5g01550	LECRKA4.2 (lectin receptor kinase a4.1); Encodes LecRKA4.2, a member of the lectin receptor kinase	1.713	9.29E-05
At5g01732	other RNA; Potential natural antisense gene, locus overlaps with AT5G01730	1.175	0.00322
At5g01740	Nuclear transport factor 2 (NTF2) family protein	-1.652	0.00015
At5g01830	ARM repeat superfamily protein	1.587	4.43E-08
At5g01880	RING/U-box superfamily protein	-1.123	0.0033
At5g02170	Transmembrane amino acid transporter family protein	1.04	0.00048
At5g02290	NAK (Protein kinase superfamily protein); Encodes a candidate protein kinase NAK that is similar to th	1.871	8.56E-07
At5g02480	HSP20-like chaperones superfamily protein	-1.149	3.45E-05
At5g02490	Heat shock protein 70 (Hsp 70) family protein	1.73	0.00014
At5g02680	LOCATED IN: endomembrane system	1.478	1.04E-06
At5g02760	Protein phosphatase 2C family protein	-1.52	4.94E-07
At5g03010	Galactose oxidase/kelch repeat superfamily protein	1.525	1.75E-08
At5g03210	unknown protein	5.653	3.83E-09
At5g03230	Protein of unknown function, DUF584	-1.2	2.29E-06
At5g03285	other RNA	1.412	1.75E-07
At5g03340	ATPase, AAA-type, CDC48 protein	2.139	7.93E-09
At5g03380	Heavy metal transport/detoxification superfamily protein	1.954	1.88E-07
At5g03390	Protein of unknown function (DUF295)	1.401	2.21E-06
At5g03435	Ca2+dependent plant phosphoribosyltransferase family protein (Ca2+dependent plant phosphoribosyltr	1.152	3.32E-06
At5g03700	D-mannose binding lectin protein with Apple-like carbohydrate-binding domain	1.179	1.51E-05
At5g03990	unknown protein	3.85	8.71E-11
At5g04020	calmodulin binding	1.442	2.83E-06
At5g04190	PKS4 (phytochrome kinase substrate 4); Encodes phytochrome kinase substrate 4, a phytochrome signa	-1.177	0.00011
At5g04340	CZF2 (COLD INDUCED ZINC FINGER PROTEIN 2); putative c2h2 zinc finger transcription factor m	6.091	8.07E-12
At5g04410	anac078 (Arabidopsis NAC domain containing protein 78); NAC family member, functions as a transcr	1.236	1.99E-06
At5g04610	S-adenosyl-L-methionine-dependent methyltransferases superfamily protein	2.61	1.53E-09
At5g04720	ADR1-L2 (ADR1-like 2)	1.758	3.39E-06
At5g04840	bZIP protein	1.716	5.54E-09
At5g04930	ALA1 (aminophospholipid ATPase 1); Encodes a putative aminophospholipid translocase (p-type ATPa	1.644	1.86E-06
At5g04960	Plant invertase/pectin methylesterase inhibitor superfamily	1.108	0.0192
At5g05220	unknown protein	4.984	2.18E-11
At5g05300	unknown protein	4.146	1.05E-07
At5g05320	FAD/NAD(P)-binding oxidoreductase family protein	1.419	6.21E-07
At5g05340	Peroxidase superfamily protein	-1.567	0.0051
At5g05410	DREB2 (DEHYDRATION-RESPONSIVE ELEMENT BINDING PROTEIN 2); Encodes a transcription	6.069	2.03E-11
At5g05420	FKBP-like peptidyl-prolyl cis-trans isomerase family protein	-1.095	0.00011
At5g05500	Pollen Ole e 1 allergen and extensin family protein	1.547	0.00276
At5g06320	NHL3 (NDR1/HIN1-like 3); encodes a protein whose sequence is similar to tobacco hairpin-induced ge	2.83	3.44E-08
At5g06510	NF-YA10 (nuclear factor Y, subunit A10)	-1.039	0.00597
At5g06750	Protein phosphatase 2C family protein	1.251	8.22E-08
At5g06820	SRF2 (STRUBBELIG-receptor family 2)	1.97	5.67E-08
At5g07330	unknown protein	2.813	1.85E-10
At5g07690	ATMYB29 (myb domain protein 29); Encodes a putative transcription factor (MYB29).	-1.173	0.00029
At5g07700	AtMYB76 (myb domain protein 76); Encodes a putative transcription factor (MYB76).	-1.522	1.39E-05
At5g08240	unknown protein	1.859	0.0001
At5g08790	anac081 (Arabidopsis NAC domain containing protein 81); induced by wounding, belongs to a large fa	3.692	2.53E-10
At5g09590	HSC70-5 (HEAT SHOCK COGNATE); heat shock protein 70 (Hsc70-5); nuclear	1.271	1.49E-06
At5g09800	ARM repeat superfamily protein	2.737	6.15E-09
At5g09930	ATGCN2 (ARABIDOPSIS THALIANA GENERAL CONTROL NON-REPRESSIBLE 2); member of (	2.391	2.56E-07
At5g10040	unknown protein	-2.629	0.002
At5g10336	unknown protein	1.06	5.36E-06
At5g10380	RING1 (RING/U-box superfamily protein); Encodes a RING finger domain protein with E3 ligase activ	1.093	0.01385
At5g10695	unknown protein	5.67	7.55E-11
At5g10830	S-adenosyl-L-methionine-dependent methyltransferases superfamily protein	2.163	3.98E-10
At5g10970	C2H2 and C2HC zinc fingers superfamily protein	-1.782	1.96E-09
At5g10990	SAUR-like auxin-responsive protein family	2.219	6.24E-06
At5g11100	SYT4 (synaptotagmin 4)	1.905	1.55E-07
At5g11140	Arabidopsis phospholipase-like protein (PEARLI 4) family	2.521	9.14E-10
At5g11250	Disease resistance protein (TIR-NBS-LRR class)	1.238	2.94E-06
At5g11650	alpha/beta-Hydrolases superfamily protein	1.69	1.04E-07
At5g11680	FUNCTIONS IN: molecular_function unknown; INVOLVED IN: biological_process unknown; LOCAT	1.652	3.12E-08
At5g11930	Thioredoxin superfamily protein	1.085	8.46E-05

At5g12020	HSP17.6II (17.6 kDa class II heat shock protein)	6.498	1.65E-09
At5g12030	AT-HSP17.6A (heat shock protein 17.6A); Encodes a cytosolic small heat shock protein with chaperone	6.363	2.42E-09
At5g12110	Glutathione S-transferase, C-terminal-like	1.472	9.17E-07
At5g12340	unknown protein	1.102	0.01623
At5g12930	unknown protein	1.047	4.16E-06
At5g13180	ANAC083 (NAC domain containing protein 83); Encodes a NAC domain transcription factor that intera	-1.145	1.18E-05
At5g13190	CONTAINS InterPro DOMAIN/s: LPS-induced tumor necrosis factor alpha factor (InterPro:IPR006629	2.567	1.40E-08
At5g13200	GRAM domain family protein	3.653	6.33E-09
At5g13210	Uncharacterised conserved protein UCP015417, vWA	1.719	0.0003
At5g14020	Endosomal targeting BRO1-like domain-containing protein	2.278	8.99E-09
At5g14110	Protein of unknown function (DUF 3339)	-1.335	7.24E-07
At5g14360	Ubiquitin-like superfamily protein	-1.086	4.91E-06
At5g14410	unknown protein	-1.007	0.0001
At5g14470	GHMP kinase family protein	1.043	0.00253
At5g14700	NAD(P)-binding Rossmann-fold superfamily protein	2.247	8.43E-07
At5g14730	unknown protein	1.14	2.04E-06
At5g14930	SAG101 (senescence-associated gene 101); encodes an acyl hydrolase involved in senescence .	1.074	0.0026
At5g14940	Major facilitator superfamily protein	-1.343	1.47E-05
At5g15120	Protein of unknown function (DUF1637)	-1.069	0.03417
At5g15250	FTSH6 (FTSH protease 6); Encodes an FtsH protease that is localized to the chloroplast. AtFtsH6 is inv	5.705	1.29E-13
At5g15310	ATMYB16 (myb domain protein 16); Member of the R2R3 factor gene family.	-1.425	1.54E-06
At5g15830	AtbZIP3 (basic leucine-zipper 3)	-1.349	5.14E-07
At5g15870	glycosyl hydrolase family 81 protein	1.057	1.17E-05
At5g16200	50S ribosomal protein-related	1.115	1.00E-07
At5g16640	Pentatricopeptide repeat (PPR) superfamily protein	-1.088	1.55E-06
At5g16960	Zinc-binding dehydrogenase family protein	1.144	6.75E-06
At5g16970	AER (alkenal reductase); encodes a 2-alkenal reductase (EC 1.3.1.74), plays a key role in the detoxifica	1.061	6.79E-05
At5g16980	Zinc-binding dehydrogenase family protein	1.429	2.39E-05
At5g17000	Zinc-binding dehydrogenase family protein	1.283	7.22E-06
At5g17350	unknown protein	3.782	4.34E-09
At5g17820	Peroxidase superfamily protein	2.913	0.00257
At5g17860	CAX7 (calcium exchanger 7)	-1.527	1.51E-06
At5g18010	SAUR-like auxin-responsive protein family	-2.249	4.29E-08
At5g18020	SAUR-like auxin-responsive protein family	-3.181	3.88E-09
At5g18030	SAUR-like auxin-responsive protein family	-3.021	4.26E-09
At5g18050	SAUR-like auxin-responsive protein family	-2.408	1.54E-08
At5g18060	SAUR-like auxin-responsive protein family	-2.168	2.92E-08
At5g18065	unknown protein	2.166	3.11E-08
At5g18080	SAUR-like auxin-responsive protein family	-2.793	5.39E-09
At5g18150	Methyltransferase-related protein	1.271	5.15E-07
At5g18290	SIP1B (SMALL AND BASIC INTRINSIC PROTEIN 1B); Belongs to a family of plant aquaporins.Sim	2.392	1.17E-08
At5g18340	ARM repeat superfamily protein	3.423	7.93E-11
At5g18400	Cytokine-induced anti-apoptosis inhibitor 1, Fe-S biogenesis	1.399	1.95E-05
At5g18470	Curculin-like (mannose-binding) lectin family protein	2.365	1.34E-06
At5g18490	Plant protein of unknown function (DUF946)	2.588	5.73E-11
At5g18930	BUD2 (BUSHY AND DWARF 2)	-1.432	3.10E-08
At5g19190	unknown protein	-1.383	1.89E-06
At5g19230	Glycoprotein membrane precursor GPI-anchored	2.512	2.64E-08
At5g19240	Glycoprotein membrane precursor GPI-anchored	1.482	9.26E-06
At5g19875	unknown protein	1.121	6.57E-06
At5g20225	other RNA; Potential natural antisense gene, locus overlaps with AT5G20220	4.892	2.63E-11
At5g20230	ATBCB (blue-copper-binding protein); Al-stress-induced gene	3.258	0.0004
At5g20670	Protein of unknown function (DUF1677)	-1.32	1.67E-05
At5g20790	unknown protein	-1.816	0.00098
At5g21900	RNI-like superfamily protein	1.039	1.51E-05
At5g21940	unknown protein	-1.151	1.22E-05
At5g22060	ATJ2 (ARABIDOPSIS THALIANA DNAJ HOMOLOGUE 2); Co-chaperonin similar to E. coli DnaJ	1.129	5.91E-06
At5g22250	Polynucleotidyl transferase, ribonuclease H-like superfamily protein	3.688	1.05E-10
At5g22410	RHS18 (root hair specific 18)	1.305	0.0002
At5g22520	unknown protein	2.9	2.15E-06
At5g22530	unknown protein	3.824	2.87E-07
At5g22540	Plant protein of unknown function (DUF247)	3.518	1.21E-06
At5g22630	ADT5 (arogenate dehydratase 5); Encodes a plastid-localized arogenate dehydratase involved in phenyla	1.27	5.61E-05
At5g22670	F-box/RNI-like/FBD-like domains-containing protein	1.874	2.34E-10
At5g22680	FUNCTIONS IN: molecular_function unknown; INVOLVED IN: biological_process unknown; LOCAT	2.563	6.27E-06
At5g22690	Disease resistance protein (TIR-NBS-LRR class) family	3.144	4.01E-11
At5g22930	Protein of unknown function (DUF1635)	-1.1	6.06E-05

At5g23480	SWIB/MDM2 domain	1.595	9.84E-07
At5g23510	unknown protein	1.514	1.14E-06
At5g24110	ATWRKY30 (ARABIDOPSIS THALIANA WRKY DNA-BINDING PROTEIN 30); member of WRK	5.352	1.26E-10
At5g24210	alpha/beta-Hydrolases superfamily protein	1.298	0.006
At5g24590	ANAC091 (Arabidopsis NAC domain containing protein 91); Member of NAc protein family. Interacts	1.603	4.20E-07
At5g24655	LSU4 (RESPONSE TO LOW SULFUR 4)	3.113	8.31E-08
At5g24660	LSU2 (RESPONSE TO LOW SULFUR 2)	4.277	1.07E-10
At5g25050	Major facilitator superfamily protein	1.897	2.45E-09
At5g25160	ZFP3 (zinc finger protein 3); Encodes a zinc finger protein containing only a single zinc finger.	-1.283	0.00013
At5g25170	PPPDE putative thiol peptidase family protein	1.558	6.95E-08
At5g25190	Integrase-type DNA-binding superfamily protein; encodes a member of the ERF (ethylene response fact	-1.512	7.96E-06
At5g25250	SPFH/Band 7/PHB domain-containing membrane-associated protein family	2.595	0.0008
At5g25350	EBF2 (EIN3-binding F box protein 2); Arabidopsis thaliana EIN3-binding F-box protein 2 (EBF2) mRN	-1.206	3.38E-06
At5g25440	Protein kinase superfamily protein	1.427	0.00018
At5g25450	Cytochrome bd ubiquinol oxidase, 14kDa subunit	3.065	0.00509
At5g25910	AtRLP52 (receptor like protein 52); putative disease resistance protein induced by chitin oligomers.	2.608	8.17E-09
At5g25920	BEST Arabidopsis thaliana protein match is: Eukaryotic aspartyl protease family protein (TAIR:AT3G2	2.797	3.59E-07
At5g25930	Protein kinase family protein with leucine-rich repeat domain	3.997	1.55E-10
At5g26030	FC1 (ferrochelatase 1); encodes ferrochelatase I located in plastids. Involved in heme biosynthesis in no	1.758	8.79E-07
At5g26200	Mitochondrial substrate carrier family protein	-1.761	8.43E-07
At5g26220	ChaC-like family protein	4.028	3.95E-10
At5g26920	CBP60G (Cam-binding protein 60-like G); Encodes a calmodulin-binding protein CBP60g (calmodulin	3.061	6.24E-07
At5g27420	CNI1 (carbon/nitrogen insensitive 1); Encodes CNI1 (Carbon/Nitrogen Insensitive1) (also named as AT	6.362	3.21E-11
At5g27660	Trypsin family protein with PDZ domain	2.728	4.63E-10
At5g27771	pseudogene of auxin-responsive family protein; pseudogene of auxin-responsive family protein	-1.35	3.16E-05
At5g27780	SAUR-like auxin-responsive protein family	-1.793	2.00E-08
At5g28176	pseudogene, hypothetical protein (transposable element gene)	2.466	6.58E-10
At5g28300	Duplicated homeodomain-like superfamily protein	-1.174	7.64E-06
At5g28610	BEST Arabidopsis thaliana protein match is: glycine-rich protein (TAIR:AT5G28630.1)	2.511	9.94E-06
At5g28630	glycine-rich protein	2.764	4.82E-07
At5g29037	gypsy-like retrotransposon family, has a 2.2e-209 P-value blast match to GB:AAD19359 polyprotein (g	1.696	0.00751
At5g32072	pseudogene of Glucose-6-phosphate isomerase; pseudogene of Glucose-6-phosphate isomerase	1.063	0.00072
At5g35320	unknown protein	3.863	3.97E-12
At5g35460	unknown protein	1.133	1.72E-06
At5g35480	unknown protein	-1.278	0.00176
At5g35735	Auxin-responsive family protein	4.809	1.09E-09
At5g36002	other RNA; Potential natural antisense gene, locus overlaps with AT5G36001 and AT5G36000	1.123	2.54E-05
At5g36075	MuDr-element domain (Mutator-like transposase family, has a 3.2e-61 P-value blast match to Q9S9L6 /	-1.141	0.04599
At5g36920	unknown protein	1.617	7.73E-08
At5g36925	unknown protein	1.629	1.54E-05
At5g36970	NHL25 (NDR1/HIN1-like 25); NDR1/HIN1-like protein, expression induced during incompatible respo	2.464	2.20E-05
At5g37340	ZPR1 zinc-finger domain protein	2.759	3.21E-11
At5g37400	Family of unknown function (DUF577)	1.266	2.70E-06
At5g37420	Family of unknown function (DUF577); Note that previous reports (Plant Cell 2003,15:1538; PNAS 20	1.88	2.29E-06
At5g37670	HSP20-like chaperones superfamily protein	5.797	3.21E-11
At5g37750	Chaperone DnaJ-domain superfamily protein	1.104	2.49E-05
At5g37770	CML24 (CALMODULIN-LIKE 24); Encodes a protein with 40% similarity to calmodulin. Binds Ca(2+	1.886	1.63E-05
At5g38240	Protein kinase family protein	1.329	7.03E-07
At5g38310	unknown protein	2.592	4.26E-09
At5g38895	RING/U-box superfamily protein	1.51	2.77E-09
At5g39000	Malectin/receptor-like protein kinase family protein	1.002	0.00095
At5g39020	Malectin/receptor-like protein kinase family protein	3.376	1.12E-10
At5g39030	Protein kinase superfamily protein	1.237	1.05E-05
At5g39080	HXXXD-type acyl-transferase family protein	-1.323	5.98E-07
At5g39610	ANAC092 (Arabidopsis NAC domain containing protein 92); Encodes a NAC-domain transcription fac	-1.549	1.94E-05
At5g39670	Calcium-binding EF-hand family protein	3.601	2.73E-05
At5g39785	Protein of unknown function (DUF1666)	1.87	2.35E-07
At5g39860	BHLH136 (BASIC HELIX-LOOP-HELIX PROTEIN 136); Encodes PRE1 (PACLOBUTRAZOL RESI	-1.147	7.21E-05
At5g39890	Protein of unknown function (DUF1637)	-1.526	0.0085
At5g40170	AtRLP54 (receptor like protein 54)	1.518	7.18E-07
At5g40460	unknown protein	2.015	1.52E-08
At5g40880	WD-40 repeat family protein / zfwd3 protein (ZFWD3)	1.211	2.45E-05
At5g41100	FUNCTIONS IN: molecular_function unknown; INVOLVED IN: biological_process unknown; LOCAT	2.038	8.46E-07
At5g41320	unknown protein	1.022	9.36E-05
At5g41550	Disease resistance protein (TIR-NBS-LRR class) family	2.268	2.93E-08
At5g41610	ATCHX18 (ARABIDOPSIS THALIANA CATION/H+ EXCHANGER 18); member of Putative Na+/H	1.405	0.00011
At5g41740	Disease resistance protein (TIR-NBS-LRR class) family	2.805	3.65E-07

At5g41750	Disease resistance protein (TIR-NBS-LRR class) family	4.108	2.05E-07
At5g41900	alpha/beta-Hydrolases superfamily protein	-1.143	5.37E-06
At5g42050	DCD (Development and Cell Death) domain protein	1.981	5.26E-07
At5g42150	Glutathione S-transferase family protein	1.119	2.60E-05
At5g42200	RING/U-box superfamily protein	-1.346	0.0002
At5g42380	CML37 (calmodulin like 37)	8.336	1.57E-08
At5g42440	Protein kinase superfamily protein	1.24	4.62E-06
At5g42510	Disease resistance-responsive (dirigent-like protein) family protein	1.019	0.01344
At5g42830	HXXXD-type acyl-transferase family protein	1.445	6.80E-05
At5g42965	Polynucleotidyl transferase, ribonuclease H-like superfamily protein	1.586	1.02E-05
At5g43170	AZF3 (zinc-finger protein 3); Encodes zinc finger protein. mRNA levels are elevated in response to hig	1.839	2.06E-07
At5g43290	ATWRKY49 (ARABIDOPSIS THALIANA WRKY DNA-BINDING PROTEIN 49); member of WRKY	-1.373	0.00012
At5g43420	RING/U-box superfamily protein	2.649	1.26E-07
At5g43620	Pre-mRNA cleavage complex II	2.148	9.48E-08
At5g43650	BHLH92 (basic helix-loop-helix (bHLH) DNA-binding superfamily protein)	1.189	0.00105
At5g43700	ATAUX2-11 (AUXIN INDUCIBLE 2-11); Auxin inducible protein similar to transcription factors.	-1.259	7.16E-05
At5g44060	unknown protein	3.155	6.11E-09
At5g44065	unknown protein	2.201	2.74E-09
At5g44070	ATPCS1 (ARABIDOPSIS THALIANA PHYTOCHELATIN SYNTHASE 1); Phytochelatin synthase ge	2.672	6.65E-09
At5g44350	ethylene-responsive nuclear protein -related	2.054	3.50E-07
At5g44360	FAD-binding Berberine family protein	1.701	8.37E-08
At5g44575	unknown protein	-1.873	0.0117
At5g44585	unknown protein	1.022	0.00414
At5g44620	CYP706A3 (cytochrome P450, family 706, subfamily A, polypeptide 3); member of CYP706A	1.388	1.39E-05
At5g44680	DNA glycosylase superfamily protein	-1.092	0.00027
At5g44870	Disease resistance protein (TIR-NBS-LRR class) family	1.47	1.97E-07
At5g44990	Glutathione S-transferase family protein	2.417	1.54E-05
At5g45110	NPR3 (NPR1-like protein 3); Encodes a paralog of NPR1. Involved in negative regulation of defense re	1.802	4.03E-08
At5g45340	CYP707A3 (cytochrome P450, family 707, subfamily A, polypeptide 3); Encodes a protein with ABA 8	3.024	1.16E-07
At5g45630	Protein of unknown function, DUF584	4.528	5.98E-10
At5g45820	CIPK20 (CBL-interacting protein kinase 20); Encodes a CBL-interacting serine/threonine protein kinas	-1.439	0.00013
At5g46080	Protein kinase superfamily protein	3.103	1.41E-10
At5g46170	F-box family protein	1.512	1.51E-08
At5g46295	unknown protein	4.917	1.48E-10
At5g46470	RPS6 (RESISTANT TO P. SYRINGAE 6); Encodes RPS6 (RESISTANT TO P. SYRINGAE 6), a mem	1.464	6.97E-05
At5g46500	BEST Arabidopsis thaliana protein match is: disease resistance protein (TIR-NBS-LRR class) family (T	1.007	1.34E-06
At5g46510	Disease resistance protein (TIR-NBS-LRR class) family	1.128	1.25E-07
At5g46520	Disease resistance protein (TIR-NBS-LRR class) family	1.126	1.78E-05
At5g46710	PLATZ transcription factor family protein	2.693	6.84E-10
At5g46780	VQ motif-containing protein	2.402	1.88E-08
At5g46910	Transcription factor jumonji (jmj) family protein / zinc finger (C5HC2 type) family protein	1.982	5.23E-09
At5g47070	Protein kinase superfamily protein	1.991	3.72E-08
At5g47120	ATBI-1 (ARABIDOPSIS BAX INHIBITOR 1); Encodes BI-1, a homolog of mammalian Bax inhibitor	1.747	1.76E-07
At5g47160	YDG/SRA domain-containing protein	1.353	2.50E-06
At5g47220	ATERF-2 (ETHYLENE RESPONSE FACTOR- 2); Encodes a member of the ERF (ethylene response fa	2.237	2.95E-09
At5g47230	ATERF-5 (ETHYLENE RESPONSIVE ELEMENT BINDING FACTOR- 5); encodes a member of the	4.897	4.40E-09
At5g47370	HAT2 (Homeobox-leucine zipper protein 4 (HB-4) / HD-ZIP protein); homeobox-leucine zipper genes i	-1.78	7.78E-08
At5g47830	unknown protein	2.088	1.72E-09
At5g47850	CCR4 (CRINKLY4 related 4)	3.277	1.74E-09
At5g47910	RBOHD (respiratory burst oxidase homologue D); NADPH/respiratory burst oxidase protein D (RbohD	2.358	1.74E-07
At5g47960	ATRABA4C (RAB GTPase homolog A4C); Encodes a small molecular weight g-protein.	3.592	6.76E-09
At5g48070	ATXTH20 (XYLOGLUCAN ENDOTRANSGLUCOSYLASE/HYDROLASE 20); putative xyloglucan	1.592	2.10E-06
At5g48180	NSP5 (nitrile specifier protein 5); Encodes a nitrile-specifier protein NSP5. NSP5 is one out of five (At3	1.094	3.61E-05
At5g48380	BIR1 (BAK1-interacting receptor-like kinase 1); Encodes a BAK1-interacting receptor-like kinase name	1.909	1.54E-08
At5g48530	unknown protein	3.045	1.05E-10
At5g48540	receptor-like protein kinase-related family protein	3.851	4.92E-09
At5g48570	FKBP-type peptidyl-prolyl cis-trans isomerase family protein	3.174	1.96E-09
At5g48610	unknown protein	1.006	1.44E-05
At5g48655	RING/U-box superfamily protein	1.31	2.86E-07
At5g48657	defense protein-related	1.474	0.0005
At5g48850	ATSDI1 (SULPHUR DEFICIENCY-INDUCED 1); homologous to the wheat sulphate deficiency-induc	4.162	1.70E-07
At5g49330	ATMYB111 (ARABIDOPSIS MYB DOMAIN PROTEIN 111); Member of the R2R3 factor gene famil	-1.295	2.08E-06
At5g49450	AtbZIP1 (basic leucine-zipper 1)	1.087	0.00043
At5g49480	ATCP1 (Ca2+-binding protein 1); AtCP1 encodes a novel Ca2+-binding protein, which shares sequenc	1.149	8.34E-05
At5g49520	ATWRKY48 (ARABIDOPSIS THALIANA WRKY DNA-BINDING PROTEIN 48); Encodes WRKY4	2.012	1.90E-07
At5g49730	ATFRO6 (ferric reduction oxidase 6); Encodes a plasma membrane-located ferric chelate reductase. Its	-1.626	0.0018
At5g49740	ATFRO7 (FERRIC REDUCTION OXIDASE 7); Encodes a chloroplast ferric chelate reductase. Shows	-1.011	0.0053

At5g50120	Transducin/WD40 repeat-like superfamily protein	-1.063	0.00532
At5g50610	unknown protein	-1.035	0.00115
At5g50800	Nodulin MtN3 family protein	-1.579	0.00585
At5g51190	Integrase-type DNA-binding superfamily protein; encodes a member of the ERF (ethylene response fact	4.184	7.76E-07
At5g51370	RNI-like superfamily protein	1.17	4.16E-08
At5g51440	HSP20-like chaperones superfamily protein	4.312	2.31E-09
At5g51630	Disease resistance protein (TIR-NBS-LRR class) family	1.904	2.09E-08
At5g51740	Peptidase family M48 family protein	1.95	1.62E-09
At5g52050	MATE efflux family protein	1.26	2.03E-05
At5g52250	Transducin/WD40 repeat-like superfamily protein; Encodes a transducin protein whose gene expressior	-1.074	3.44E-07
At5g52640	AtHsp90-1 (HEAT SHOCK PROTEIN 90-1); Encodes a cytosolic heat shock protein AtHSP90.1. AtH	3.747	5.48E-09
At5g52740	Copper transport protein family	2.187	1.44E-06
At5g52750	Heavy metal transport/detoxification superfamily protein	2.981	3.13E-08
At5g52760	Copper transport protein family	3.849	3.86E-05
At5g52790	CBS domain-containing protein with a domain of unknown function (DUF21)	2.227	3.17E-09
At5g52900	unknown protein	-2.443	2.99E-08
At5g53048	other RNA; Potential natural antisense gene, locus overlaps with AT5G53050	-1.114	0.0147
At5g53130	ATCNGC1 (CYCLIC NUCLEOTIDE-GATED CHANNEL 1); member of Cyclic nucleotide gated chan	1.089	8.53E-07
At5g53400	BOB1 (BOBBER1); Encodes BOBBER1 (BOB1), a non-canonical small heat shock protein required fo	1.265	2.98E-07
At5g53680	RNA-binding (RRM/RBD/RNP motifs) family protein	2.327	5.49E-11
At5g54060	UF3GT (UDP-glucose:flavonoid 3-o-glucosyltransferase)	-1.349	0.0229
At5g54130	Calcium-binding endonuclease/exonuclease/phosphatase family	-1.155	0.00012
At5g54145	unknown protein	-2.475	7.57E-10
At5g54165	unknown protein	7.291	2.21E-11
At5g54490	PBP1 (pinoid-binding protein 1); Encodes a PINOID (PID)-binding protein containing putative EF-hanc	4.765	1.68E-10
At5g54585	unknown protein	-1.159	0.0004
At5g54710	Ankyrin repeat family protein	2.135	9.30E-08
At5g54720	Ankyrin repeat family protein	3.594	3.04E-08
At5g54730	ATATG18F (ARABIDOPSIS THALIANA HOMOLOG OF YEAST AUTOPHAGY 18 (ATG18) F)	1.054	3.36E-05
At5g54860	Major facilitator superfamily protein	2.387	9.68E-10
At5g55620	unknown protein	-1.071	1.99E-05
At5g55970	RING/U-box superfamily protein	2.338	8.09E-07
At5g56040	Leucine-rich receptor-like protein kinase family protein	-1.064	6.70E-07
At5g56550	ATOXS3 (OXIDATIVE STRESS 3); Encodes OXIDATIVE STRESS 3 (OXS3)， involved in t	-1.654	9.88E-08
At5g56580	ANQ1 (ARABIDOPSIS NQK1); Encodes a member of the MAP Kinase Kinase family of proteins. It ca	1.065	5.07E-05
At5g56880	unknown protein	2.49	2.26E-07
At5g56960	basic helix-loop-helix (bHLH) DNA-binding family protein	5.692	5.73E-11
At5g56980	unknown protein	1.053	0.0002
At5g57010	calmodulin-binding family protein	2.673	1.76E-05
At5g57035	U-box domain-containing protein kinase family protein	1.295	1.02E-06
At5g57050	ABI2 (ABA INSENSITIVE 2); Encodes a protein phosphatase 2C and is involved in ABA signal transd	1.311	6.05E-06
At5g57123	unknown protein	1.227	0.00218
At5g57220	CYP81F2 (cytochrome P450, family 81, subfamily F, polypeptide 2); member of CYP81F, involved in g	3.996	4.51E-09
At5g57480	P-loop containing nucleoside triphosphate hydrolases superfamily protein	1.564	7.09E-09
At5g57500	Galactosyltransferase family protein	1.418	1.49E-08
At5g57510	unknown protein	2.574	0.00034
At5g57520	ATZFP2 (ZINC FINGER PROTEIN 2); Encodes a zinc finger protein containing only a single zinc fing	-1.03	9.11E-05
At5g57560	TCH4 (Touch 4); Encodes a cell wall-modifying enzyme, rapidly upregulated in response to environmen	2.246	0.00057
At5g57625	CAP (Cysteine-rich secretory proteins, Antigen 5, and Pathogenesis-related 1 protein) superfamily prote	1.381	0.0149
At5g57760	unknown protein	-1.541	1.99E-05
At5g57780	EXPRESSED IN: 18 plant structures	-1.634	1.53E-06
At5g57840	HXXXD-type acyl-transferase family protein; encodes a protein whose sequence is similar to anthranila	-1.341	0.03257
At5g57887	unknown protein	-1.003	4.43E-06
At5g57910	unknown protein	1.146	2.92E-06
At5g58070	ATTIL (TEMPERATURE-INDUCED LIPOCALIN); Encodes a temperature-induced lipocalin TIL1. In	1.097	1.82E-05
At5g58120	Disease resistance protein (TIR-NBS-LRR class) family	2.354	3.60E-07
At5g58360	ATOFP3 (ARABIDOPSIS THALIANA OVATE FAMILY PROTEIN 3)	-1.404	1.91E-07
At5g58430	ATEXO70B1 (exocyst subunit exo70 family protein B1); A member of EXO70 gene family, putative ex	2.469	5.62E-11
At5g58570	unknown protein	-1.058	0.00045
At5g58680	ARM repeat superfamily protein	3.776	7.24E-12
At5g58787	RING/U-box superfamily protein	2.25	6.29E-11
At5g58900	Homeodomain-like transcriptional regulator	-1.139	4.83E-06
At5g58940	CRCK1 (calmodulin-binding receptor-like cytoplasmic kinase 1); Arabidopsis thaliana calmodulin-binc	1.499	3.35E-07
At5g59070	UDP-Glycosyltransferase superfamily protein	2.734	1.36E-08
At5g59360	unknown protein	-1.046	7.97E-06
At5g59450	GRAS family transcription factor	1.466	0.0002
At5g59510	DVL18 (DEVIL 18)	1.796	3.72E-06

At5g59550	zinc finger (C3HC4-type RING finger) family protein	2.99	4.95E-08
At5g59720	HSP18.2 (heat shock protein 18.2); encodes a low molecular weight heat shock protein that contains the	3.947	3.08E-10
At5g59730	ATEXO70H7 (exocyst subunit exo70 family protein H7); A member of EXO70 gene family, putative ex	2.191	5.25E-07
At5g59780	ATMYB59 (MYB DOMAIN PROTEIN 59); Encodes a putative transcription factor (MYB59).	-2.611	3.07E-09
At5g59820	RHL41 (RESPONSIVE TO HIGH LIGHT 41); Encodes a zinc finger protein involved in high light and	5.421	1.31E-08
At5g60050	BTB/POZ domain-containing protein	-1.05	7.46E-07
At5g60130	AP2/B3-like transcriptional factor family protein	1.497	7.15E-08
At5g60140	AP2/B3-like transcriptional factor family protein	-1.795	0.0358
At5g60270	Concanavalin A-like lectin protein kinase family protein	1.701	3.47E-06
At5g60530	late embryogenesis abundant protein-related / LEA protein-related	1.118	0.00761
At5g60610	F-box/RNI-like superfamily protein	1.135	3.05E-06
At5g60800	Heavy metal transport/detoxification superfamily protein	1.25	0.00018
At5g60850	OBP4 (OBF binding protein 4); Encodes a zinc finger protein.	-1.288	5.74E-07
At5g60890	ATR1 (ALTERED TRYPTOPHAN REGULATION 1); Myb-like transcription factor that modulates ex	-1.316	3.26E-06
At5g61420	HAG1 (HIGH ALIPHATIC GLUCOSINOLATE 1); Encodes a nuclear localized member of the MYB t	-1.597	1.66E-07
At5g61560	U-box domain-containing protein kinase family protein	2.499	9.45E-10
At5g61570	Protein kinase superfamily protein	-1.162	3.18E-06
At5g61590	Integrase-type DNA-binding superfamily protein; encodes a member of the ERF (ethylene response fact	-1.981	6.20E-08
At5g61600	ERF104 (ethylene response factor 104); encodes a member of the ERF (ethylene response factor) subfar	3.575	3.53E-09
At5g61900	BON1 (BONZAI 1); Encodes a plasma-membrane localized, copine-like protein, which is a member of	2.092	1.23E-07
At5g62020	AT-HSFB2A (ARABIDOPSIS THALIANA HEAT SHOCK TRANSCRIPTION FACTOR B2A); mem	3.569	1.24E-10
At5g62280	Protein of unknown function (DUF1442)	-2.61	3.20E-07
At5g62340	Plant invertase/pectin methylesterase inhibitor superfamily protein	2.018	0.00721
At5g62430	CDF1 (cycling DOF factor 1); Dof-type zinc finger domain-containing protein, similar to H-protein pro	1.047	0.0001
At5g62470	ATMYB96 (MYB DOMAIN PROTEIN 96); Encodes a R2R3 type Myb transcription factor whose exp	1.011	1.13E-05
At5g62520	similar to RCD one 5 (SRO5); FUNCTIONS IN: NAD+ ADP-ribosyltransferase activity; INVOLVED I	-1.472	0.01667
At5g62570	Calmodulin binding protein-like	2.129	5.37E-08
At5g62620	Galactosyltransferase family protein	1.228	1.53E-07
At5g62730	Major facilitator superfamily protein	-1.532	0.00222
At5g62770	Protein of unknown function (DUF1645)	2.125	1.37E-07
At5g62900	unknown protein	-1.063	2.00E-06
At5g62920	ARR6 (response regulator 6); Encodes a Type-A response regulator that is responsive to cytokinin treat	-1.422	3.13E-06
At5g63130	Octicosapeptide/Phox/Bem1p family protein	3.23	2.94E-09
At5g63300	Ribosomal protein S21 family protein	1.633	8.83E-07
At5g63370	Protein kinase superfamily protein	1.362	6.91E-09
At5g63500	Protein of unknown function (DUF 3339)	1.087	5.03E-06
At5g63790	ANAC102 (NAC domain containing protein 102); Encodes a member of the NAC family of transcriptic	2.451	1.87E-10
At5g64190	unknown protein	-1.402	0.00053
At5g64310	AGP1 (arabinogalactan protein 1); Encodes arabinogalactan-protein (AGP1).	2.196	1.99E-08
At5g64510	unknown protein	4.372	5.63E-11
At5g64660	CMPG2 (CYS, MET, PRO, and GLY protein 2)	2.353	5.82E-10
At5g64810	ATWRKY51 (ARABIDOPSIS THALIANA WRKY DNA-BINDING PROTEIN 51); member of WRKY	1.154	0.02037
At5g64870	SPFH/Band 7/PHB domain-containing membrane-associated protein family	2.777	8.95E-06
At5g64900	ATPEP1 (ARABIDOPSIS THALIANA PEPTIDE 1); Encodes a putative 92-aa protein that is the precu	1.096	1.43E-05
At5g64905	PROPEP3 (elicitor peptide 3 precursor)	2.793	5.41E-08
At5g64950	Mitochondrial transcription termination factor family protein	1.469	1.96E-07
At5g65300	unknown protein	1.448	0.00357
At5g65600	Concanavalin A-like lectin protein kinase family protein	3.59	5.85E-08
At5g65660	hydroxyproline-rich glycoprotein family protein	-1.241	1.26E-06
At5g65920	ARM repeat superfamily protein	2.514	8.86E-09
At5g66070	RING/U-box superfamily protein	2.364	2.99E-06
At5g66210	CPK28 (calcium-dependent protein kinase 28); member of Calcium Dependent Protein Kinase	1.954	7.86E-06
At5g66480	unknown protein	1.783	2.01E-08
At5g66580	unknown protein	-2.75	3.00E-08
At5g66620	DAR6 (DA1-related protein 6)	2.384	2.49E-07
At5g66640	DAR3 (DA1-related protein 3)	2.161	4.57E-06
At5g66650	Protein of unknown function (DUF607)	2.672	6.39E-06
At5g66670	Protein of unknown function (DUF677)	1.398	9.86E-08
At5g66675	Protein of unknown function (DUF677)	2.906	4.43E-09
At5g66790	Protein kinase superfamily protein	2.002	1.08E-07
At5g66850	MAPKKK5 (mitogen-activated protein kinase kinase kinase 5); member of MEKK subfamily	1.138	5.93E-05
At5g66880	SNRK2-3 (SUCROSE NONFERMENTING 1 (SNF1)-RELATED PROTEIN KINASE 2-3); encodes a	2.204	3.96E-10
At5g66900	Disease resistance protein (CC-NBS-LRR class) family	1.525	4.53E-07
At5g66910	Disease resistance protein (CC-NBS-LRR class) family	1.034	5.80E-07
At5g66985	unknown protein	-1.644	0.0218
At5g67190	DEAR2 (DREB and EAR motif protein 2); encodes a member of the DREB subfamily A-5 of ERF/AP2	-1.419	5.45E-07
At5g67310	CYP81G1 (cytochrome P450, family 81, subfamily G, polypeptide 1); member of CYP81G	1.872	0.00204

At5g67340	ARM repeat superfamily protein	1.601	2.21E-06
At5g67350	unknown protein	1.019	0.0001
At5g67390	unknown protein	-1.802	4.01E-06
At5g67430	Acyl-CoA N-acyltransferases (NAT) superfamily protein	1.01	0.00043
At5g67450	AZF1 (zinc-finger protein 1); Encodes zinc-finger protein. mRNA levels are elevated in response to low	3.066	3.48E-08

List of genes significantly affected (P<0.05 and log2≥1 or ≤-1) by allyl-ITC after 9h

Locus ID	gene description (TAIR)	log2 ITC/mock 9h	adj.P.Val
At1g01060	LHY (LATE ELONGATED HYPOCOTYL); LHY encodes a myb-related putative transcription factor involve	3.558	1.40E-09
At1g01190	CYP78A8 (cytochrome P450, family 78, subfamily A, polypeptide 8); member of CYP78A	-1.521	4.02E-06
At1g01200	ATRAB-A3 (ARABIDOPSIS RAB GTPASE HOMOLOG A3)	-1.178	1.14E-06
At1g01250	Integrase-type DNA-binding superfamily protein; encodes a member of the DREB subfamily A-4 of ERF/AP2	-1.629	3.46E-06
At1g01300	Eukaryotic aspartyl protease family protein	-1.047	8.01E-05
At1g01340	ATCNGC10 (cyclic nucleotide gated channel 10); member of Cyclic nucleotide gated channel family	1.456	7.95E-06
At1g01390	UDP-Glycosyltransferase superfamily protein	-1.655	1.71E-05
At1g01420	UGT72B3 (UDP-glucosyl transferase 72B3)	-1.435	1.42E-07
At1g01430	TBL25 (TRICHOME BIREFRINGENCE-LIKE 25); Encodes a member of the TBL (TRICHOME BIREFRIN(	-1.257	1.84E-08
At1g01520	Homeodomain-like superfamily protein	1.21	0.0005
At1g01560	ATMPK11 (MAP kinase 11); member of MAP Kinase	4.247	2.94E-07
At1g01720	ANAC002 (Arabidopsis NAC domain containing protein 2); Belongs to a large family of putative transcriptior	2.066	1.00E-08
At1g01930	zinc finger protein-related	1.31	1.81E-08
At1g01940	Cyclophilin-like peptidyl-prolyl cis-trans isomerase family protein	2.746	3.80E-08
At1g02000	GAE2 (UDP-D-glucuronate 4-epimerase 2); UDP-D-glucuronate 4-epimerase	1.272	3.37E-07
At1g02090	ATCSN7 (ARABIDOPSIS THALIANA COP9 SIGNALOSOME SUBUNIT 7); encodes a phosphoprotein tha	2.374	2.49E-09
At1g02170	AMC1 (metacaspase 1); Metacaspase AtMCP1b. Arginine/lysine-specific cysteine protease activity. Induces a	1.347	3.64E-08
At1g02220	ANAC003 (NAC domain containing protein 3)	1.395	8.43E-05
At1g02230	ANAC004 (NAC domain containing protein 4)	2.209	2.46E-05
At1g02260	Divalent ion symporter	-1.105	2.73E-07
At1g02300	Cysteine proteinases superfamily protein	-1.091	5.77E-06
At1g02340	HFR1 (LONG HYPOCOTYL IN FAR-RED); Encodes a light-inducible, nuclear bHLH protein involved in phy	-1.553	9.22E-06
At1g02400	ATGA2OX4 (Arabidopsis thaliana gibberellin 2-oxidase 4); Encodes a gibberellin 2-oxidase that acts on C19	2.877	2.08E-09
At1g02450	NIMIN1 (NIM1-interacting 1); NIMIN1 modulates PR gene expression according the following model: NPR1	-1.347	0.00714
At1g02470	Polyketide cyclase/dehydrase and lipid transport superfamily protein	-1.73	6.12E-06
At1g02590	Aldehyde oxidase/xanthine dehydrogenase, molybdopterin binding protein	1.63	1.94E-08
At1g02630	Nucleoside transporter family protein	-1.207	0.00151
At1g02640	ATBXL2 (BETA-XYLOSIDASE 2); encodes a protein similar to a beta-xylosidase located in the extracellular	-1.564	3.07E-07
At1g02700	unknown protein	4.353	1.24E-12
At1g02710	glycine-rich protein	-1.392	2.12E-05
At1g02750	Drought-responsive family protein	1.437	3.52E-08
At1g02860	BAH1 (BENZOIC ACID HYPERSENSITIVE 1); Encodes a likely ubiquitin E3 ligase with RING and SPX do	1.092	8.93E-07
At1g02880	TPK1 (thiamin pyrophosphokinase1); Encodes a thiamine pyrophosphokinase capable of producing thiamine p	1.139	3.35E-06
At1g02890	AAA-type ATPase family protein	1.449	8.07E-07
At1g02920	ATGST11 (ARABIDOPSIS GLUTATHIONE S-TRANSFERASE 11); Encodes glutathione transferase belong	2.149	6.32E-05
At1g02930	ATGST1 (ARABIDOPSIS GLUTATHIONE S-TRANSFERASE 1); Encodes glutathione transferase belongin	1.806	1.82E-05
At1g02980	CUL2 (cullin 2); encodes an Arabidopsis cullin	3.817	6.86E-09
At1g03010	Phototropic-responsive NPH3 family protein	-1.842	6.59E-08
At1g03055	unknown protein	1.101	0.00077
At1g03070	Bax inhibitor-1 family protein	8.217	1.53E-13
At1g03180	unknown protein	2.373	2.90E-09
At1g03190	ATXPD (ARABIDOPSIS THALIANA XERODERMA PIGMENTOSUM GROUP D); UV damage and heat ii	1.135	1.78E-07
At1g03220	Eukaryotic aspartyl protease family protein	2.453	2.93E-09
At1g03230	Eukaryotic aspartyl protease family protein	2.041	1.57E-09
At1g03300	ATDUF1 (DOMAIN OF UNKNOWN FUNCTION 724 1); Member of the plant-specific DUF724 protein fam	-1.079	4.23E-06
At1g03520	Core-2/I-branching beta-1,6-N-acetylglucosaminyltransferase family protein	-1.162	1.10E-06
At1g03580	pseudogene, meprin and TRAF homology (MATH) domain protein -related, weak similarity to ubiquitin-speci	-2.136	1.42E-07
At1g03620	ELMO/CED-12 family protein	-1.473	1.17E-08
At1g03750	CHR9 (CHROMATIN REMODELING 9)	1.29	3.64E-08
At1g03820	unknown protein	-1.561	6.85E-06
At1g03860	ATPHB2 (prohibitin 2); prohibitin 2	1.008	7.02E-05
At1g03870	FLA9 (FASCICLIN-like arabinoogalactan 9); fasciclin-like arabinogalactan-protein 9 (Fla9)	-1.359	7.80E-06
At1g03905	P-loop containing nucleoside triphosphate hydrolases superfamily protein	2.938	7.88E-11
At1g03970	GBF4 (G-box binding factor 4); encodes a basic leucine zipper G-box binding factor that can bind to G-box m	1.474	6.26E-09
At1g03980	ATPCS2 (phytochelatin synthase 2); Encodes a protein with phytochelatin synthase activity which binds Cd2+	1.198	2.32E-05
At1g03982	PAK-box/P21-Rho-binding family protein	3.008	2.16E-11
At1g04010	PSAT1 (phospholipid sterol acyl transferase 1)	1.774	9.58E-09
At1g04050	SDG13 (SET DOMAIN PROTEIN 13); Encodes SUVR1, one of the four closely related Arabidopsis SUVR pı	2.72	5.35E-10
At1g04090	Plant protein of unknown function (DUF946)	1.755	7.21E-07
At1g04130	Tetratricopeptide repeat (TPR)-like superfamily protein	2.921	8.43E-10
At1g04160	ATXIB (ARABIDOPSIS THALIANA MYOSIN XI B); Encodes a member of the type XI myosin protein fami	1.42	2.99E-06
At1g04240	IAA3 (indole-3-acetic acid inducible 3); SHY2/IAA3 regulates multiple auxin responses in roots. It is induced	-1.844	6.75E-07
At1g04400	CRY2 (cryptochrome 2); Blue light receptor mediating blue-light regulated cotyledon expansion and flowering	-1.045	9.42E-08
At1g04570	Major facilitator superfamily protein	5.027	1.75E-11
At1g04600	XIA (myosin XI A); member of Myosin-like proteins	1.126	4.79E-05

At1g04770	Tetratricopeptide repeat (TPR)-like superfamily protein	2.243	9.61E-10
At1g04800	glycine-rich protein	-1.138	0.02203
At1g04960	Protein of unknown function (DUF1664)	1.846	1.68E-08
At1g05170	Galactosyltransferase family protein	2.787	1.03E-10
At1g05200	ATGLR3.4 (glutamate receptor 3.4); member of Putative ligand-gated ion channel subunit family	-1.204	1.29E-07
At1g05300	ZIP5 (zinc transporter 5 precursor); member of Fe(II) transporter isolog family	-1.361	3.77E-06
At1g05320	FUNCTIONS IN: molecular_function unknown; INVOLVED IN: biological_process unknown; LOCATED IN	-1.228	1.05E-07
At1g05340	unknown protein	1.783	6.07E-05
At1g05420	ATOFP12 (ARABIDOPSIS THALIANA OVATE FAMILY PROTEIN 12)	-1.127	1.25E-05
At1g05530	UGT2 (UDP-GLUCOSYL TRANSFERASE 2); Encodes a protein with glucosyltransferase activity with high s	1.35	2.14E-05
At1g05560	UGT1 (UDP-GLUCOSE TRANSFERASE 1); A UDP-glucose transferase localized in the phragmoplast. It ha	1.913	7.26E-08
At1g05575	unknown protein	1.113	1.63E-05
At1g05590	ATHEX3 (BETA-HEXOSAMINIDASE 3); Encodes a protein with β-hexosaminidase activity (the enzy	-1.009	4.35E-07
At1g05610	APS2 (ADP-glucose pyrophosphorylase small subunit 2); Encodes the small subunit of ADP-glucose pyrophos	-1.063	2.42E-05
At1g05680	UGT74E2 (Uridine diphosphate glycosyltransferase 74E2); Encodes a UDP-glucosyltransferase, UGT74E2, tl	4.204	1.26E-07
At1g05700	Leucine-rich repeat transmembrane protein kinase protein	4.186	1.39E-09
At1g05805	basic helix-loop-helix (bHLH) DNA-binding superfamily protein	-1.434	4.78E-09
At1g05894	unknown protein	1.227	2.37E-07
At1g06030	pfkB-like carbohydrate kinase family protein	3.766	1.15E-11
At1g06080	ADS1 (delta 9 desaturase 1); homologous to delta 9 acyl-lipid desaturases of cyanobacteria and acyl-CoA desa	-1.715	2.75E-06
At1g06100	Fatty acid desaturase family protein	-1.477	0.00039
At1g06430	FTSH8 (FTSH protease 8); encodes a FtsH protease that is localized to the chloroplast	1.303	3.54E-07
At1g06460	ACD31.2 (ALPHA-CRYSTALLIN DOMAIN 31.2); ACD32.1 encodes an alpha-crystallin domain containing	-1.628	5.90E-08
At1g06520	GPAT1 (glycerol-3-phosphate acyltransferase 1); Encodes a membrane associated mitochondrial localized prot	-1.225	4.74E-06
At1g06570	HPD (4-HYDROXYPHENYLPYRUVATE DIOXYGENASE); Mutation of the PDS1 locus disrupts the activi	1.342	4.41E-07
At1g06800	PLA-I{gamma}1 (phospholipase A I gamma 1); Encodes a lipase that hydrolyzes phosphatidylcholine, glycoli	2.115	4.38E-08
At1g06870	Peptidase S24/S26A/S26B/S26C family protein	1.073	3.90E-07
At1g06980	unknown protein	-1.508	1.12E-06
At1g07000	ATEXO70B2 (exocyst subunit exo70 family protein B2); A member of EXO70 gene family, putative exocyst s	2.686	6.41E-08
At1g07010	Calcineurin-like metallo-phosphoesterase superfamily protein	2.08	3.89E-09
At1g07050	CCT motif family protein	-3.555	4.38E-09
At1g07160	Protein phosphatase 2C family protein	4.979	3.46E-10
At1g07180	ATNDI1 (ARABIDOPSIS THALIANA INTERNAL NON-PHOSPHORYLATING NAD (P) H DEHYDROG	3.294	5.94E-10
At1g07190	BEST Arabidopsis thaliana protein match is: lon protease 1 (TAIR:AT5G26860.1)	1.23	1.35E-08
At1g07280	Tetratricopeptide repeat (TPR)-like superfamily protein	-1.013	1.80E-05
At1g07300	josephin protein-related	1.487	7.43E-09
At1g07330	unknown protein	6.509	6.14E-13
At1g07340	ATSTP2 (sugar transporter 2)	3.017	4.24E-11
At1g07350	RNA-binding (RRM/RBD/RNP motifs) family protein	4.221	2.20E-12
At1g07400	HSP20-like chaperones superfamily protein	8.628	8.24E-12
At1g07410	ATRAB-A2B (ARABIDOPSIS RAB GTPASE HOMOLOG A2B)	3.743	7.45E-12
At1g07420	ATSMO2 (STEROL 4-ALPHA-METHYL-OXIDASE 2); Arabidopsis thaliana sterol 4-alpha-methyl-oxidase r	1.445	5.33E-07
At1g07450	NAD(P)-binding Rossmann-fold superfamily protein	1.137	3.06E-06
At1g07470	Transcription factor IIA, alpha/beta subunit	1.161	6.83E-09
At1g07480	Transcription factor IIA, alpha/beta subunit	1.572	6.99E-08
At1g07500	unknown protein	7.503	3.99E-13
At1g07510	ftsh10 (FTSH protease 10); encodes an FtsH protease that is localized to the mitochondrion	1.089	1.91E-07
At1g07530	ATGRAS2 (ARABIDOPSIS THALIANA GRAS (GAI, RGA, SCR) 2); Encodes a member of the GRAS fami	1.004	5.71E-06
At1g07640	OBP2 (Dof-type zinc finger DNA-binding family protein); A member of the DOF transcription factors. Promin	-1.049	1.50E-05
At1g07700	Thioredoxin superfamily protein	1.036	9.79E-08
At1g07720	KCS3 (3-ketoacyl-CoA synthase 3); Encodes KCS3, a member of the 3-ketoacyl-CoA synthase family involved	-1.329	5.51E-07
At1g07860	BEST Arabidopsis thaliana protein match is: Protein kinase superfamily protein (TAIR:AT1G07870.2)	2.208	2.00E-10
At1g07870	Protein kinase superfamily protein	1.08	4.27E-05
At1g07900	LBD1 (LOB domain-containing protein 1)	1.565	0.00014
At1g07985	Expressed protein	1.477	2.05E-05
At1g08090	ATNRT2.1 (NITRATE TRANSPORTER 2.1); High-affinity nitrate transporter. Up-regulated by nitrate. Funct	-1.73	0.00014
At1g08105	hAT-like transposase family (hobo/Ac/Tam3), has a 1.0e-298 P-value blast match to GB:AAD24567 transposa	3.36	4.59E-11
At1g08165	unknown protein	-1.933	5.12E-05
At1g08300	NVL (NO VEIN-LIKE)	1.371	1.83E-05
At1g08310	alpha/beta-Hydrolases superfamily protein	-1.678	0.00137
At1g08500	ENODL18 (early nodulin-like protein 18)	-1.079	8.02E-07
At1g08570	ACHT4 (atypical CYS HIS rich thioredoxin 4); Encodes a member of the thioredoxin family protein. Located	1.239	4.34E-08
At1g08810	AtMYB60 (myb domain protein 60); putative transcription factor of the R2R3-MYB gene family. Transcript in	-1.589	7.20E-06
At1g08860	BON3 (BONZAI 3); Encodes a copine-like protein, which is a member of a newly identified class of calcium-c	1.212	0.00084
At1g08930	ERD6 (EARLY RESPONSE TO DEHYDRATION 6); encodes a putative sucrose transporter whose gene expr	2.046	1.95E-09
At1g08940	Phosphoglycerate mutase family protein	2.24	1.55E-09
At1g09070	(AT)SRC2 (SOYBEAN GENE REGULATED BY COLD-2); SRC2 specifically binds the peptide PIEPPPHH,	1.978	3.30E-08

At1g09080	BIP3 (Heat shock protein 70 (Hsp 70) family protein)	2.158	0.005
At1g09140	ATSRP30 (SERINE-ARGININE PROTEIN 30); Encodes a serine-arginine rich RNA binding protein involved	3.214	1.81E-10
At1g09157	Protein of unknown function (DUF679)	3.859	2.47E-11
At1g09240	ATNAS3 (ARABIDOPSIS THALIANA NICOTIANAMINE SYNTHASE 3); Encodes a nicotianamine syntha	-2.98	1.96E-05
At1g09250	basic helix-loop-helix (bHLH) DNA-binding superfamily protein	-1.023	0.0002
At1g09340	CRB (chloroplast RNA binding); Encodes CHLOROPLAST RNA BINDING (CRB), a putative RNA-binding	1.403	2.15E-06
At1g09350	AtGolS3 (galactinol synthase 3)	-1.743	2.84E-06
At1g09500	NAD(P)-binding Rossmann-fold superfamily protein; similar to Eucalyptus gunnii alcohol dehydrogenase of u	1.5	7.16E-05
At1g09740	Adenine nucleotide alpha hydrolases-like superfamily protein	1.605	4.14E-08
At1g09750	Eukaryotic aspartyl protease family protein	-1.255	2.13E-06
At1g09780	Phosphoglycerate mutase, 2,3-bisphosphoglycerate-independent	-1.205	1.19E-06
At1g09932	Phosphoglycerate mutase family protein	1.096	0.0097
At1g09940	HEMA2 (Glutamyl-tRNA reductase family protein); Encodes glutamyl-tRNA reductase. Involved in heme bio:	2.691	2.03E-10
At1g09970	RLK7 (receptor-like kinase 7); RLK7 belongs to a leucine-rich repeat class of receptor-likekinase (LRR-RLKs)	1.036	2.10E-06
At1g10040	alpha/beta-Hydrolases superfamily protein	5.119	6.56E-13
At1g10050	glycosyl hydrolase family 10 protein / carbohydrate-binding domain-containing protein; Encodes a putative gly	3.257	3.75E-11
At1g10060	ATBCAT-1 (branched-chain amino acid transaminase 1); encodes a mitochondrial branched-chain amino acid	-1.214	0.0003
At1g10155	ATPP2-A10 (phloem protein 2-A10)	1.971	2.64E-08
At1g10220	BEST Arabidopsis thaliana protein match is: ZCF37 (TAIR:AT1G59590.1)	2.407	4.68E-08
At1g10230	ASK18 (SKP1-like 18)	2.071	1.26E-09
At1g10300	Nucleolar GTP-binding protein	1.685	1.30E-05
At1g10320	Zinc finger C-x8-C-x5-C-x3-H type family protein	1.163	8.69E-07
At1g10370	ATGSTU17 (GLUTATHIONE S-TRANSFERASE TAU 17)	2.098	2.77E-07
At1g10550	XET (XYLOGLUCAN:XYLOGLUCOSYL TRANSFERASE 33); Encodes a membrane-localized protein that	-1.619	3.73E-05
At1g10585	basic helix-loop-helix (bHLH) DNA-binding superfamily protein	1.508	0.0005
At1g10620	Protein kinase superfamily protein	1.014	4.92E-06
At1g10760	SEX1 (STARCH EXCESS 1); Encodes an α-glucan, water dikinase required for starch degradation. Inv	-1.89	1.11E-09
At1g10770	Plant invertase/pectin methylesterase inhibitor superfamily protein; Encodes a putative pectin methylesterase/i	-1.962	2.95E-08
At1g10960	ATFD1 (ferredoxin 1)	1.308	3.22E-08
At1g11050	Protein kinase superfamily protein	1.196	6.12E-06
At1g11100	SNF2 domain-containing protein / helicase domain-containing protein / zinc finger protein-related	4.376	2.50E-12
At1g11160	Transducin/WD40 repeat-like superfamily protein	1.219	2.55E-06
At1g11210	Protein of unknown function (DUF761)	-2.067	1.22E-05
At1g11260	ATSTP1 (SUGAR TRANSPORTER 1); Encodes a H+/hexose cotransporter.	-2.009	1.06E-05
At1g11530	ATCXXS1 (C-terminal cysteine residue is changed to a serine 1); Encodes a monocysteinic thioredoxin, thior	-1.375	3.46E-06
At1g11540	Sulfite exporter TauE/SafE family protein	-2.304	5.09E-11
At1g11545	XTH8 (xyloglucan endotransglucosylase/hydrolase 8)	-1.35	1.22E-05
At1g11660	heat shock protein 70 (Hsp 70) family protein	2.607	7.99E-11
At1g11700	Protein of unknown function, DUF584	-1.434	8.22E-08
At1g11740	ankyrin repeat family protein	-2.11	8.05E-08
At1g11803	pseudogene, auxin responsive protein, blastp match of 74% identity and 1.2e-15 P-value to GP|3043536|dbj|B	-1.937	2.97E-07
At1g11850	unknown protein	-1.589	2.25E-07
At1g11880	transferases, transferring hexosyl groups	1.608	1.58E-09
At1g12010	2-oxoglutarate (2OG) and Fe(II)-dependent oxygenase superfamily protein; Encodes a protein that appears to	-1.464	8.22E-05
At1g12030	Protein of unknown function (DUF506)	5.383	3.35E-08
At1g12060	ATBAG5 (BCL-2-associated athanogene 5); A member of Arabidopsis BAG (Bcl-2-associated athanogene) pr	3.004	9.80E-11
At1g12064	unknown protein	3.776	4.89E-09
At1g12080	Vacuolar calcium-binding protein-related	-1.784	3.69E-05
At1g12090	ELP (extensin-like protein); extensin-like protein (ELP)	-1.643	4.72E-07
At1g12110	ATNRT1 (ARABIDOPSIS THALIANA NITRATE TRANSPORTER 1); Encodes NRT1.1 (CHL1), a dual-affi	-1.118	0.0005
At1g12120	Plant protein of unknown function (DUF863)	-1.138	5.24E-08
At1g12150	Plant protein of unknown function (DUF827)	-2.379	2.33E-05
At1g12360	KEU (keule); encodes a Sec1 protein and expressed throughout the plant. physically interacts with Syntaxin1	1.699	5.90E-09
At1g12480	CDI3 (CARBON DIOXIDE INSENSITIVE 3); Encodes a membrane protein with 10 predicted transmembrane	-1.31	7.91E-06
At1g12740	CYP87A2 (cytochrome P450, family 87, subfamily A, polypeptide 2); encodes a protein with cytochrome P45	1.707	2.59E-08
At1g12845	unknown protein	-1.165	1.28E-05
At1g12940	ATNRT2.5 (nitrate transporter2.5); member of High affinity nitrate transporter family	-1.328	0.00989
At1g13080	CYP71B2 (cytochrome P450, family 71, subfamily B, polypeptide 2); cytochrome P450 monooxygenase	1.851	1.28E-08
At1g13110	CYP71B7 (cytochrome P450, family 71 subfamily B, polypeptide 7); member of CYP71B	-1.233	3.99E-06
At1g13195	RING/U-box superfamily protein	2.509	2.23E-11
At1g13245	DVL4 (DEVIL 4)	-1.474	6.61E-08
At1g13250	GATL3 (galacturonosyltransferase-like 3); Encodes a protein with putative galacturonosyltransferase activity.	-1.21	4.02E-07
At1g13300	HRS1 (HYPERSENSITIVITY TO LOW PI-ELICITED PRIMARY ROOT SHORTENING 1); Overexpressio	-2.904	1.84E-05
At1g13310	Endosomal targeting BRO1-like domain-containing protein	3.017	9.17E-09
At1g13340	Regulator of Vps4 activity in the MVB pathway protein	3.254	3.62E-05
At1g13430	ATST4C (ARABIDOPSIS THALIANA SULFOTRANSFERASE 4C); Encodes a sulfotransferase. Unlike the	-1.354	0.00014
At1g13448	other RNA; Potential natural antisense gene, locus overlaps with AT1G13450	-1.548	4.53E-08

At1g13470	Protein of unknown function (DUF1262)	-1.127	0.0217
At1g13620	RGF2 (root meristem growth factor 2); Encodes a root meristem growth factor (RGF). Belongs to a family of f	1.505	1.01E-07
At1g13670	unknown protein	-1.281	6.21E-05
At1g13690	ATE1 (ATPase E1); AtE1 - stimulates the ATPase activity of DnaK/DnaJ	1.135	1.54E-06
At1g13700	PGL1 (6-phosphogluconolactonase 1)	-1.762	5.33E-07
At1g13750	Purple acid phosphatases superfamily protein	-1.55	9.49E-05
At1g13890	SNAP30 (soluble N-ethylmaleimide-sensitive factor adaptor protein 30); Encodes a member of a gene family h	1.056	0.01188
At1g13990	unknown protein	1.645	3.15E-06
At1g14048	GCK domain-containing protein	1.308	9.19E-09
At1g14070	FUT7 (fucosyltransferase 7); member of Xyloglucan fucosyltransferase family	1.014	6.82E-05
At1g14190	Glucose-methanol-choline (GMC) oxidoreductase family protein	-1.465	1.77E-06
At1g14200	RING/U-box superfamily protein	4.251	3.68E-11
At1g14205	Ribosomal L18p/L5e family protein	1.304	0.0014
At1g14210	Ribonuclease T2 family protein	-1.29	1.36E-06
At1g14260	RING/FYVE/PHD zinc finger superfamily protein	1.188	4.40E-05
At1g14280	PKS2 (phytochrome kinase substrate 2); Encodes phytochrome kinase substrate 2. PKS proteins are critical fo	-1.256	3.42E-05
At1g14360	UTR3 (UDP-galactose transporter 3)	2.013	5.04E-10
At1g14370	APK2A (protein kinase 2A); Encodes protein kinase APK2a.	2.08	3.68E-08
At1g14420	AT59 (Pectate lyase family protein)	1.554	7.78E-09
At1g14540	Peroxidase superfamily protein	4.387	4.60E-09
At1g14640	SWAP (Suppressor-of-White-APricot)/surp domain-containing protein	1.341	4.74E-07
At1g14700	PAP3 (purple acid phosphatase 3)	-1.411	1.02E-07
At1g14780	MAC/Perforin domain-containing protein	1.224	8.20E-06
At1g14870	PCR2 (PLANT CADMIUM RESISTANCE 2)	2.142	0.0003
At1g14920	GAI (GIBBERELLIC ACID INSENSITIVE); Similar to a putative transcription factor and transcriptional coac	-1.338	8.43E-09
At1g14970	O-fucosyltransferase family protein	1.474	7.80E-09
At1g15010	unknown protein	-1.184	0.01402
At1g15040	Class I glutamine amidotransferase-like superfamily protein	-1.895	0.0011
At1g15110	phosphatidyl serine synthase family protein	1.502	8.66E-08
At1g15170	MATE efflux family protein	1.521	3.75E-08
At1g15190	Fasciclin-like arabinogalactan family protein	2.205	8.43E-09
At1g15260	unknown protein	-1.378	1.25E-07
At1g15300	hAT-like transposase family (hobo/Ac/Tam3), has a 5.1e-59 P-value blast match to GB:CAA29005 ORFa of M	1.418	1.29E-06
At1g15380	Lactoylglutathione lyase / glyoxalase I family protein	-1.436	0.01155
At1g15415	FUNCTIONS IN: molecular_function unknown; INVOLVED IN: biological_process unknown; LOCATED IN	3.39	1.68E-09
At1g15430	Protein of unknown function (DUF1644)	3.318	3.18E-11
At1g15520	ABCG40 (ATP-BINDING CASSETTE FAMILY G40); ABC transporter family involved in ABA transport an	3.846	0.00115
At1g15530	Concanavalin A-like lectin protein kinase family protein	1.554	1.35E-08
At1g15540	2-oxoglutarate (2OG) and Fe(II)-dependent oxygenase superfamily protein	3.913	3.59E-09
At1g15580	AUX2-27 (AUXIN-INDUCIBLE 2-27); auxin induced protein	-1.478	1.50E-07
At1g16022	unknown protein	1.853	1.57E-08
At1g16030	Hsp70b (heat shock protein 70B)	7.272	4.43E-13
At1g16040	FUNCTIONS IN: molecular_function unknown; INVOLVED IN: GPI anchor biosynthetic process; LOCATED	2.841	8.36E-11
At1g16060	ADAP (ARIA-interacting double AP2 domain protein); Encodes ADAP, an AP2-domain protein that interacts	1.578	8.73E-10
At1g16090	WAKL7 (wall associated kinase-like 7); WAK-like kinase	2.516	1.34E-07
At1g16130	WAKL2 (wall associated kinase-like 2); wall-associated kinase like	1.464	1.90E-05
At1g16290	FUNCTIONS IN: molecular_function unknown; INVOLVED IN: biological_process unknown; LOCATED IN	1.467	1.02E-06
At1g16370	ATOCT6 (ARABIDOPSIS THALIANA ORGANIC CATION/CARNITINE TRANSPORTER 6)	-1.004	0.00012
At1g16400	CYP79F2 (cytochrome P450, family 79, subfamily F, polypeptide 2); Encodes cytochrome P450 CYP79F2.	-2.722	3.17E-09
At1g16410	BUS1 (BUSHY 1); member of CYP79F	-4.39	3.00E-09
At1g16420	ATMC8 (ARABIDOPSIS THALIANA METACASPASE 8); Encodes a metacaspase (cysteine-type endopepti	4.798	2.01E-07
At1g16540	ABA3 (ABA DEFICIENT 3); Encodes molybdenum cofactor sulfurase. Involved in Moco biosynthesis. Involv	1.277	5.50E-09
At1g16630	unknown protein	1.687	7.66E-07
At1g16640	AP2/B3-like transcriptional factor family protein	1.767	1.73E-08
At1g16670	Protein kinase superfamily protein	1.686	1.56E-08
At1g16730	UP6 (unknown protein 6)	2.16	2.90E-07
At1g16750	Protein of unknown function, DUF547	-1.457	8.56E-09
At1g16960	Ubiquitin domain-containing protein	-1.486	3.18E-07
At1g16980	ATTPS2 (trehalose-phosphatase/synthase 2); Encodes an enzyme putatively involved in trehalose biosynthesis	1.264	4.59E-06
At1g17050	SPS2 (solanesyl diphosphate synthase 2); Encodes a protein with solanesyl diphosphate synthase activity.	1.576	1.12E-07
At1g17170	ATGSTU24 (glutathione S-transferase TAU 24); Encodes glutathione transferase belonging to the tau class of	3.265	1.38E-06
At1g17180	ATGSTU25 (glutathione S-transferase TAU 25); Encodes glutathione transferase belonging to the tau class of	2.976	4.14E-05
At1g17240	AtRLP2 (receptor like protein 2); Encodes a CLAVATA2 (CLV2)-related gene. Complements the clv2 mutant	1	0.00165
At1g17330	Metal-dependent phosphohydrolase	1.398	4.32E-07
At1g17430	alpha/beta-Hydrolases superfamily protein	-1.137	3.76E-06
At1g17490	unknown protein	-1.039	4.00E-08
At1g17610	Disease resistance protein (TIR-NBS class)	1.508	5.64E-07

At1g17665	unknown protein	-1.436	1.70E-05
At1g17700	PRA1.F1 (prenylated RAB acceptor 1.F1)	-1.457	5.48E-07
At1g17710	Pyridoxal phosphate phosphatase-related protein	-2.36	0.00129
At1g17870	ATEGY3 (ETHYLENE-DEPENDENT GRAVITROPISM-DEFICIENT AND YELLOW-GREEN-LIKE 3); S2	5.794	3.39E-12
At1g18000	Major facilitator superfamily protein	-1.18	5.10E-07
At1g18200	AtRABA6b (RAB GTPase homolog A6B)	2.392	1.18E-09
At1g18300	atnudt4 (nudix hydrolase homolog 4)	1.386	1.46E-07
At1g18330	EPR1 (EARLY-PHYTOCHROME-RESPONSIVE1); EARLY-PHYTOCHROME-RESPONSIVE1	2.213	5.31E-10
At1g18350	ATMKK7 (MAP kinase kinase 7); MAP kinase kinase7. Member of plant mitogen-activated protein kinase ki	-1.666	1.56E-06
At1g18380	NA	2.178	1.41E-09
At1g18382	other RNA; Potential natural antisense gene, locus overlaps with AT1G18380	1.201	9.31E-09
At1g18390	Protein kinase superfamily protein	1.635	2.13E-08
At1g18400	BEE1 (BR enhanced expression 1)	-1.339	2.85E-08
At1g18420	Aluminium activated malate transporter family protein	1.335	2.71E-06
At1g18510	TET16 (tetraspanin 16); Member of TETRASPANIN family	2.308	3.20E-07
At1g18590	ATSOT17 (SULFOTRANSFERASE 17); encodes a desulfoglucosinolate sulfotransferase, involved in the fina	-2.966	1.10E-08
At1g18620	unknown protein	-1.163	3.86E-06
At1g18710	AtMYB47 (myb domain protein 47); Member of the R2R3 factor gene family.	-1.622	4.84E-05
At1g18750	AGL65 (AGAMOUS-like 65); Encodes a member of the MIKC (MADS box, Keratin binding domain, and C t	1.157	4.77E-06
At1g18870	ATICS2 (ARABIDOPSIS ISOCHORISMATE SYNTHASE 2); Encodes a protein with isochorismate synthase	2.366	1.39E-07
At1g18890	ATCDPK1 (calcium-dependent protein kinase 1); encodes a calcium-dependent protein kinase whose gene exp	1.87	7.84E-09
At1g18960	myb-like HTH transcriptional regulator family protein	2.154	1.23E-06
At1g19020	unknown protein	4.947	5.81E-10
At1g19025	DNA repair metallo-beta-lactamase family protein	2.008	7.30E-08
At1g19180	JAZ1 (jasmonate-zim-domain protein 1); JAZ1 is a nuclear-localized protein involved in jasmonate signaling.	1.187	0.00077
At1g19190	alpha/beta-Hydrolases superfamily protein	-1.004	4.12E-06
At1g19200	Protein of unknown function (DUF581)	-2.845	3.79E-06
At1g19230	Riboflavin synthase-like superfamily protein	1.047	0.00025
At1g19340	Methyltransferase MT-A70 family protein	-1.059	3.75E-07
At1g19370	unknown protein	-1.45	5.77E-09
At1g19540	NmrA-like negative transcriptional regulator family protein	2.14	1.98E-09
At1g19610	LCR78 (LOW-MOLECULAR-WEIGHT CYSTEINE-RICH 78); Predicted to encode a PR (pathogenesis-relat	1.29	0.00465
At1g19620	unknown protein	-1.53	9.63E-06
At1g19640	JMT (jasmonic acid carboxyl methyltransferase); Encodes a S-adenosyl-L-methionine:jasmonic acid carboxyl	1.209	0.0002
At1g19740	ATP-dependent protease La (LON) domain protein	-1.039	2.90E-05
At1g19920	ASA1 (ATP SULFURYLASE ARABIDOPSIS 1); encodes a chloroplast form of ATP sulfurylase	-1.042	4.14E-07
At1g19980	cytomatrix protein-related	1.226	2.09E-07
At1g20010	TUB5 (tubulin beta-5 chain); beta tubulin	-1.074	6.49E-05
At1g20070	unknown protein	-2.121	4.49E-08
At1g20120	GDSL-like Lipase/Acylhydrolase superfamily protein	3.191	3.87E-10
At1g20190	ATEXPA11 (expansin 11); member of Alpha-Expansin Gene Family. Naming convention from the Expansin V	-2.978	1.04E-09
At1g20310	unknown protein	4.223	1.84E-09
At1g20320	Haloacid dehalogenase-like hydrolase (HAD) superfamily protein	1.085	0.00306
At1g20350	ATTIM17-1 (translocase inner membrane subunit 17-1); mitochondrial inner membrane translocase	1.664	1.14E-06
At1g20400	Protein of unknown function (DUF1204)	1.014	0.00381
At1g20460	unknown protein	1.374	3.96E-09
At1g20470	SAUR-like auxin-responsive protein family	1.105	8.28E-05
At1g20515	other RNA; Potential natural antisense gene, locus overlaps with AT1G20520	-1.046	2.75E-06
At1g20620	CAT3 (catalase 3); Catalase, catalyzes the breakdown of hydrogen peroxide (H2O2) into water and oxygen.	-1.773	1.75E-06
At1g20640	Plant regulator RWP-RK family protein	2.717	1.97E-10
At1g20696	HMGB3 (high mobility group B3); Encodes a protein belonging to the subgroup of HMGB (high mobility grou	-1.093	1.06E-06
At1g20840	TMT1 (tonoplast monosaccharide transporter1); The protein encoded by this gene is found in the tonoplast (va	-1.281	1.51E-07
At1g20925	Auxin efflux carrier family protein	2.159	4.60E-06
At1g21010	unknown protein	2.345	4.66E-09
At1g21110	O-methyltransferase family protein	2.972	3.10E-07
At1g21120	O-methyltransferase family protein	2.385	4.76E-08
At1g21210	WAK4 (wall associated kinase 4); cell wall-associated ser/thr kinase involved in cell elongation and lateral roo	1.293	1.08E-08
At1g21410	SKP2A (F-box/RNI-like superfamily protein); AtSKP2;1 is a homolog of human SKP2, the human F-box prote	1.97	1.09E-09
At1g21440	Phosphoenolpyruvate carboxylase family protein	-1.131	4.45E-08
At1g21450	SCL1 (SCARECROW-like 1); Encodes a scarecrow-like protein (SCL1). Member of GRAS gene family.	1.002	9.19E-08
At1g21530	AMP-dependent synthetase and ligase family protein	2.003	0.00018
At1g21540	AMP-dependent synthetase and ligase family protein	-1.849	8.89E-06
At1g21550	Calcium-binding EF-hand family protein	7.181	4.62E-12
At1g21600	PTAC6 (plastid transcriptionally active 6); Present in transcriptionally active plastid chromosomes. Involved in	-1.037	1.22E-06
At1g21830	unknown protein	-1.219	3.82E-08
At1g21840	UREF (urease accessory protein F); Encodes a urease accessory protein which is essential for the activation of	-1.061	1.84E-06
At1g21910	Integrase-type DNA-binding superfamily protein; encodes a member of the DREB subfamily A-5 of ERF/AP2	-2.026	1.56E-05

At1g21920	Histone H3 K4-specific methyltransferase SET7/9 family protein	-1.178	9.59E-08
At1g21940	unknown protein	2.013	4.60E-09
At1g22150	SULTR1.3 (sulfate transporter 1.3); sulfate transporter Sultr1;3	-1.024	0.01696
At1g22190	Integrase-type DNA-binding superfamily protein	1.043	3.67E-06
At1g22210	Haloacid dehalogenase-like hydrolase (HAD) superfamily protein	2.906	6.42E-11
At1g22280	PAPP2C (phytochrome-associated protein phosphatase type 2C); Encodes a phytochrome-associated protein, P	1.311	2.53E-07
At1g22310	MBD8 (methyl-CPG-binding domain 8); Protein containing methyl-CpG-binding domain.Has sequence simila	1.273	1.09E-07
At1g22330	RNA-binding (RRM/RBD/RNP motifs) family protein	-1.494	1.00E-06
At1g22340	AtUGT85A7 (UDP-glucosyl transferase 85A7)	-1.154	4.26E-06
At1g22370	AtUGT85A5 (UDP-glucosyl transferase 85A5)	-1.486	6.13E-07
At1g22470	unknown protein	1.885	2.45E-05
At1g22500	RING/U-box superfamily protein	-1.937	1.86E-05
At1g22510	RING/U-box protein with domain of unknown function (DUF 1232)	1.5	5.85E-08
At1g22530	PATL2 (PATELLIN 2)	-1.014	0.0009
At1g22550	Major facilitator superfamily protein	-1.03	5.01E-05
At1g22570	Major facilitator superfamily protein	-1.967	3.84E-09
At1g22640	ATMYB3 (ARABIDOPSIS THALIANA MYB DOMAIN PROTEIN 3); MYB-type transcription factor (MYB	-1.294	1.38E-07
At1g22710	ATSUC2 (ARABIDOPSIS THALIANA SUCROSE-PROTON SYMPORTER 2); Encodes for a high-affinity	-1.973	2.00E-06
At1g22740	RABG3B (RAB GTPase homolog G3B); GTP-binding protein Rab7	-1.259	7.58E-05
At1g22750	unknown protein	1.011	1.76E-06
At1g22770	GI (GIGANTEA); Together with CONSTANTS (CO) and FLOWERING LOCUS T (FT), GIGANTEA promo	-1.264	5.65E-07
At1g22810	Integrase-type DNA-binding superfamily protein; encodes a member of the DREB subfamily A-5 of ERF/AP2	6.374	7.25E-13
At1g22950	2-oxoglutarate (2OG) and Fe(II)-dependent oxygenase superfamily protein	1.285	2.24E-07
At1g22985	Integrase-type DNA-binding superfamily protein; encodes a member of the ERF (ethylene response factor) sub	2.256	9.40E-11
At1g22990	Heavy metal transport/detoxification superfamily protein	-1.835	0.00815
At1g23050	hydroxyproline-rich glycoprotein family protein	-1.831	7.46E-08
At1g23060	BEST Arabidopsis thaliana protein match is: TPX2 (targeting protein for Xklp2) protein family (TAIR:AT1G7	-1.396	2.14E-07
At1g23080	ATPIN7 (ARABIDOPSIS PIN-FORMED 7); Encodes a novel component of auxin efflux that is located apical	-1.85	3.35E-09
At1g23110	unknown protein	-2.621	0.00091
At1g23120	Polyketide cyclase/dehydrase and lipid transport superfamily protein	-1.21	1.77E-05
At1g23180	ARM repeat superfamily protein	1.567	5.29E-09
At1g23310	AOAT1 (ALANINE-2-OXOGLUTARATE AMINOTRANSFERASE 1); Identified by cloning the gene that co	1.091	3.75E-05
At1g23330	alpha/beta-Hydrolases superfamily protein	2.102	3.10E-11
At1g23390	Kelch repeat-containing F-box family protein	-1.401	9.69E-06
At1g23440	Peptidase C15, pyroglutamyl peptidase I-like	1.117	1.32E-05
At1g23500	GDSL-like Lipase/Acylhydrolase superfamily protein	1.296	2.21E-06
At1g23540	AtPERK12 (proline-rich extensin like receptor kinase); Encodes a member of the PERK family of putative rec	2.801	5.03E-11
At1g23550	similar to RCD one 2 (SRO2); FUNCTIONS IN: NAD+ ADP-ribosyltransferase activity; INVOLVED IN: biol	4.659	5.86E-12
At1g23710	Protein of unknown function (DUF1645)	2.844	1.18E-08
At1g23730	ATBCA3 (BETA CARBONIC ANHYDRASE 3)	1.948	6.46E-07
At1g23740	Oxidoreductase, zinc-binding dehydrogenase family protein	1.5	7.28E-08
At1g23830	unknown protein	1.741	8.25E-09
At1g24090	RNase H family protein	1.134	5.49E-07
At1g24100	UGT74B1 (UDP-glucosyl transferase 74B1); Encodes a UDP-glucose:thiohydroximate S-glucosyltransferase, i	-2.005	4.66E-09
At1g24140	Matrixin family protein	2.948	6.16E-06
At1g24145	unknown protein	1.543	0.0005
At1g24148	unknown protein	-1.208	0.0002
At1g24150	ATFH4 (FORMIN HOMOLOGUE 4); Encodes a group I formin. Localized to cell junctions. Polymerizes actin	2.338	7.46E-08
At1g24260	AGL9 (AGAMOUS-like 9); Member of the MADs box transcription factor family. SEP3 is redundant with SE	-2.977	4.62E-06
At1g24270	unknown protein	-2.198	5.50E-05
At1g24280	G6PD3 (glucose-6-phosphate dehydrogenase 3); Encodes a plastidic glucose-6-phosphate dehydrogenase that	-2.001	1.40E-06
At1g24400	AATL2 (AMINO ACID TRANSPORTER-LIKE PROTEIN 2); High-affinity transporter for neutral and acidic	-1.417	1.37E-07
At1g24530	Transducin/WD40 repeat-like superfamily protein	-1.228	1.63E-07
At1g24570	Protein of unknown function (DUF707)	1.965	4.95E-06
At1g24577	unknown protein	-2.309	4.29E-08
At1g24625	ZFP7 (zinc finger protein 7); Encodes a zinc finger protein containing only a single zinc finger.	-1.18	1.62E-07
At1g25230	Calcineurin-like metallo-phosphoesterase superfamily protein	-1.57	4.49E-08
At1g25370	Protein of unknown function (DUF1639)	1.512	2.98E-08
At1g25400	unknown protein	1.155	0.0005
At1g25440	B-box type zinc finger protein with CCT domain	-1.901	2.66E-06
At1g25450	CER60 (ECERIFERUM 60); Encodes KCS5, a member of the 3-ketoacyl-CoA synthase family involved in the	-1.258	5.56E-05
At1g25560	EDF1 (ETHYLENE RESPONSE DNA BINDING FACTOR 1); Encodes a member of the RAV transcription f	-1.506	2.55E-07
At1g25682	Family of unknown function (DUF572)	1.182	3.11E-08
At1g26210	ATSOFL1 (SOB FIVE-LIKE 1); AtSOFL1 acts redundantly with AtSOFL2 as positive regulator of cytokinin le	-1.845	5.68E-08
At1g26300	BSD domain-containing protein	1.082	2.84E-07
At1g26390	FAD-binding Berberine family protein	-1.612	0.00974
At1g26400	FAD-binding Berberine family protein	-1.098	0.0016

At1g26420	FAD-binding Berberine family protein	1.477	3.39E-05
At1g26600	CLE9 (CLAVATA3/ESR-RELATED 9); Member of a large family of putative ligands homologous to the Clav	-1.236	1.57E-07
At1g26690	emp24/gp25L/p24 family/GOLD family protein	1.328	1.45E-08
At1g26762	unknown protein	-1.112	6.11E-06
At1g26790	Dof-type zinc finger DNA-binding family protein	1.009	0.0019
At1g26800	RING/U-box superfamily protein	3.752	1.18E-10
At1g26920	unknown protein	-1.612	3.27E-07
At1g26945	KDR (KIDARI); Encodes a basic helix-loop-helix (bHLH) protein involved in blue/far-red light signaling. Phy	-1.508	5.08E-07
At1g27020	unknown protein	-1.497	0.0005
At1g27100	Actin cross-linking protein	1.526	2.46E-07
At1g27170	transmembrane receptors	2.295	2.23E-10
At1g27330	Ribosome associated membrane protein RAMP4	1.881	7.25E-09
At1g27350	Ribosome associated membrane protein RAMP4	2.118	2.48E-10
At1g27610	unknown protein	1.03	5.06E-06
At1g27660	basic helix-loop-helix (bHLH) DNA-binding superfamily protein	-1.013	2.48E-06
At1g27670	unknown protein	-1.209	5.40E-07
At1g27700	Syntaxin/t-SNARE family protein	1.812	1.86E-08
At1g27720	TAF4 (TBP-associated factor 4)	4.733	8.52E-13
At1g27730	STZ (salt tolerance zinc finger); Related to Cys2/His2-type zinc-finger proteins found in higher plants. Compe	2.962	1.95E-08
At1g27760	ATSAT32 (SALT-TOLERANCE 32); Encodes a protein with similarity to human interferon-related developm	1.087	6.27E-06
At1g27820	Polynucleotidyl transferase, ribonuclease H-like superfamily protein	2.821	2.74E-09
At1g27890	Polynucleotidyl transferase, ribonuclease H-like superfamily protein	3.078	7.43E-10
At1g27930	Protein of unknown function (DUF579); Function unknown. Interacts with eIF3.	-1.056	1.05E-06
At1g28010	PGP14 (P-glycoprotein 14)	-1.519	4.62E-07
At1g28050	B-box type zinc finger protein with CCT domain	-1.134	8.44E-08
At1g28190	unknown protein	1.572	4.41E-08
At1g28230	PUP1 (purine permease 1); Encodes a transporter that transports purines,cytokinins and other adenine derivati	-1.401	1.95E-05
At1g28330	DRM1 (DORMANCY-ASSOCIATED PROTEIN 1); dormancy-associated protein (DRM1)	-2.596	2.55E-08
At1g28380	NSL1 (necrotic spotted lesions 1); This gene is predicted to encode a protein involved in negatively regulating	1.632	2.40E-07
At1g28400	unknown protein	-1.117	0.0001
At1g28440	HSL1 (HAESA-like 1)	-1.111	4.79E-06
At1g28660	GDSL-like Lipase/Acylhydrolase superfamily protein	-1.899	2.76E-07
At1g28670	ARAB-1 (GDSL-like Lipase/Acylhydrolase superfamily protein); Arabidopsis thaliana lipase	-1.016	6.36E-08
At1g28710	Nucleotide-diphospho-sugar transferase family protein	-1.025	8.46E-06
At1g29150	ATS9 (non-ATPase subunit 9); specifically interacts with FUS6/COP11 via the C-terminal domain of FUS6/C	1.727	1.18E-08
At1g29195	unknown protein	1.255	8.00E-06
At1g29220	transcriptional regulator family protein	1.144	1.89E-07
At1g29330	AERD2 (ARABIDOPSIS ENDOPLASMIC RETICULUM RETENTION DEFECTIVE 2); Encodes a protein s	1.739	7.65E-09
At1g29340	ATPUB17 (ARABIDOPSIS THALIANA PLANT U-BOX 17); Encodes a protein containing a UND, a U-box,	1.349	9.14E-07
At1g29395	COR413-TM1 (COLD REGULATED 314 THYLAKOID MEMBRANE 1); Integral membrane protein in the i	-2.965	2.14E-09
At1g29420	SAUR-like auxin-responsive protein family	-1.152	9.31E-07
At1g29430	SAUR-like auxin-responsive protein family	-1.589	5.69E-07
At1g29440	SAUR-like auxin-responsive protein family	-2.086	8.33E-07
At1g29450	SAUR-like auxin-responsive protein family	-2.066	1.68E-06
At1g29460	SAUR-like auxin-responsive protein family	-2.83	1.06E-09
At1g29500	SAUR-like auxin-responsive protein family	-1.817	3.98E-07
At1g29510	SAUR68 (SMALL AUXIN UPREGULATED 68)	-1.483	1.86E-06
At1g29640	Protein of unknown function, DUF584	2.05	3.40E-08
At1g29660	GDSL-like Lipase/Acylhydrolase superfamily protein	-1.97	9.25E-07
At1g29670	GDSL-like Lipase/Acylhydrolase superfamily protein	-1.037	0.0012
At1g29680	Protein of unknown function (DUF1264)	5.49	6.56E-13
At1g29690	CAD1 (constitutively activated cell death 1); Encodes a protein containing a domain with significant homology	3.073	2.24E-10
At1g29724	NA	1.237	4.37E-05
At1g29770	Haloacid dehalogenase-like hydrolase (HAD) superfamily protein	1.46	2.81E-08
At1g29785	other RNA; Potential natural antisense gene, locus overlaps with AT1G29780	1.182	4.79E-05
At1g29810	Transcriptional coactivator/pterin dehydratase	1.855	1.53E-10
At1g29820	Magnesium transporter CorA-like family protein	2	3.84E-08
At1g30060	COP1-interacting protein-related	2.582	1.76E-06
At1g30070	SGS domain-containing protein	5.413	3.67E-12
At1g30210	TCP24 (TEOSINTE BRANCHED 1, cycloidea, and PCF family 24); TCP family protein involved in heterochr	-1.111	3.39E-07
At1g30280	Chaperone DnaJ-domain superfamily protein	-1.857	2.40E-08
At1g30320	Remorin family protein	1.433	5.13E-09
At1g30360	ERD4 (early-responsive to dehydration 4)	-1.35	5.05E-06
At1g30370	alpha/beta-Hydrolases superfamily protein	2.227	0.00173
At1g30390	non-LTR retrotransposon family (LINE), has a 1.9e-17 P-value blast match to GB:AAB41224 ORF2 (LINE-el	1.069	1.47E-05
At1g30455	transcription regulators	1.824	9.49E-09
At1g30473	Heavy metal transport/detoxification superfamily protein	1.192	0.0462

At1g30475	BEST Arabidopsis thaliana protein match is: embryo defective 1303 (TAIR:AT1G56200.1)	3.257	1.55E-10
At1g30480	DRT111 (DNA-DAMAGE-REPAIR/TOLERATION PROTEIN 111); recombination and DNA-damage resista	1.722	3.41E-08
At1g30570	HERK2 (hercules receptor kinase 2); Encodes HERCULES2 (HERK2), a receptor kinase regulated by Brassin	-1.018	4.89E-07
At1g30620	HSR8 (HIGH SUGAR RESPONSE8); encodes a type-II membrane protein that catalyzes 4-epimerization of U	1.131	5.19E-08
At1g30700	FAD-binding Berberine family protein	2.642	3.02E-05
At1g30720	FAD-binding Berberine family protein	1.047	0.00025
At1g30755	Protein of unknown function (DUF668)	3.617	2.81E-12
At1g30810	Transcription factor jumonji (jmj) family protein / zinc finger (C5HC2 type) family protein	1.339	1.09E-06
At1g30840	ATPUP4 (purine permease 4); Member of a family of proteins related to PUP1, a purine transporter. May be ir	-1.484	1.94E-06
At1g31130	unknown protein	1.166	1.60E-05
At1g31173	MIR167D (microRNA167D); Encodes a microRNA that targets ARF family members ARF6 and ARF8. Micr	-1.517	1.18E-05
At1g31230	AK-HSDH (ASPARTATE KINASE-HOMOSERINE DEHYDROGENASE); Encodes a bifunctional aspartate	-1.25	5.11E-06
At1g31240	Bromodomain transcription factor	-1.116	2.81E-06
At1g31280	AGO2 (argonaute 2); An Argonaute gene	2.405	1.43E-09
At1g31290	AGO3 (ARGONAUTE 3)	1.907	3.54E-06
At1g31335	unknown protein	2.3	6.78E-09
At1g31350	KUF1 (KAR-UP F-box 1)	2.145	2.42E-08
At1g31370	Ubiquitin-specific protease family C19-related protein	3.163	2.16E-11
At1g31490	HXXXD-type acyl-transferase family protein	-2	1.00E-06
At1g31550	GDSL-like Lipase/Acylhydrolase superfamily protein	-1.1	5.28E-07
At1g31580	ECS1; Encodes cell wall protein. ECS1 is not a Xcc750 resistance gene, but the genetic data indicate that ECS	1.196	0.00018
At1g31690	Copper amine oxidase family protein	-1.093	6.23E-05
At1g32330	ATHSFA1D (heat shock transcription factor A1D); Member of Heat Stress Transcription Factor (Hsf) family.	2.905	9.22E-11
At1g32350	AOX1D (alternative oxidase 1D)	2.185	0.0255
At1g32375	F-box/RNI-like/FBD-like domains-containing protein	1.198	2.29E-05
At1g32540	LOL1 (lsd one like 1); Encodes a protein with 3 plant-specific zinc finger domains that acts as a positive regul	-1.955	1.32E-08
At1g32780	GroES-like zinc-binding dehydrogenase family protein	1.002	0.00013
At1g32870	ANAC013 (Arabidopsis NAC domain containing protein 13)	2.168	4.67E-10
At1g32880	ARM repeat superfamily protein	3.412	1.54E-08
At1g32920	unknown protein	1.45	6.85E-06
At1g32928	unknown protein	2.29	2.05E-08
At1g32940	SBT3.5 (Subtilase family protein)	1.608	8.75E-06
At1g32950	Subtilase family protein	1.044	7.52E-05
At1g32960	SBT3.3 (Subtilase family protein)	2.983	1.47E-07
At1g32970	Subtilisin-like serine endopeptidase family protein	2.002	8.88E-07
At1g33030	O-methyltransferase family protein	2.354	1.14E-06
At1g33090	MATE efflux family protein	1.355	1.10E-07
At1g33160	pseudogene, similar to actin, blastp match of 74% identity and 8.3e-48 P-value to GP|9965319|gb|AAG10041.	2.589	1.08E-08
At1g33430	Galactosyltransferase family protein	2.225	6.45E-09
At1g33440	Major facilitator superfamily protein	-1.107	1.35E-05
At1g33500	unknown protein	4.074	6.32E-12
At1g33510	pseudogene, hypothetical protein	3.053	2.93E-11
At1g33560	ADR1 (ACTIVATED DISEASE RESISTANCE 1); Encodes a NBS-LRR disease resistance protein that posse	1.083	0.0001
At1g33700	Beta-glucosidase, GBA2 type family protein	-1.29	5.35E-07
At1g33720	CYP76C6 (cytochrome P450, family 76, subfamily C, polypeptide 6); member of CYP76C	-1.413	0.0003
At1g33811	GDSL-like Lipase/Acylhydrolase superfamily protein	-1.528	3.19E-05
At1g33855	MuDr-element domain (Mutator-like transposase family, has a 1.7e-18 P-value blast match to Q9SKL7 /23-18	5.74	6.82E-11
At1g34042	unknown protein	4.889	4.63E-12
At1g34260	FAB1D (FORMS APLOID AND BINUCLEATE CELLS 1A); Encodes a protein that is predicted to act as a pl	1.429	1.36E-07
At1g34575	FAD-binding Berberine family protein	1.501	0.00453
At1g34580	Major facilitator superfamily protein	-1.152	0.00012
At1g34640	peptidases	-1.069	2.76E-06
At1g35140	EXL7 (EXORDIUM LIKE 7); At1g35140 (At1g35140/T32G9_32) mRNA, complete cds	1.189	0.0382
At1g35210	unknown protein	2.065	1.15E-06
At1g35612	expressed protein (transposable element gene); pseudogene of Ulp1 protease family protein	-1.932	2.90E-08
At1g35660	unknown protein	2.576	6.51E-10
At1g35730	APUM9 (pumilio 9); Encodes a member of the Arabidopsis Pumilio (APUM) proteins containing PUF domair	2.231	1.83E-08
At1g35910	Haloacid dehalogenase-like hydrolase (HAD) superfamily protein	2.628	1.39E-08
At1g36340	UBC31 (ubiquitin-conjugating enzyme 31)	2.204	1.69E-09
At1g36370	SHM7 (serine hydroxymethyltransferase 7); Encodes a putative serine hydroxymethyltransferase.	3.682	3.67E-10
At1g36380	unknown protein	1.015	1.92E-08
At1g36622	unknown protein	1.351	2.21E-05
At1g36940	unknown protein	-1.486	7.29E-08
At1g42490	pseudogene, glutamate dehydrogenase (fragment), blastp match of 78% identity and 1.6e-94 P-value to GP|15(	-1.038	9.34E-06
At1g42590	pseudogene, hypothetical protein (transposable element gene)	2.246	2.36E-06
At1g42970	GAPB (glyceraldehyde-3-phosphate dehydrogenase B subunit); Encodes chloroplast localized glyceraldehyde-	1.132	1.53E-05
At1g42980	Actin-binding FH2 (formin homology 2) family protein	1.967	0.0058

At1g42990	ATBZIP60 (basic region/leucine zipper motif 60); AtbZIP60 consists of a bZIP DNA binding domain followe	2.25	1.58E-10
At1g43000	PLATZ transcription factor family protein	4.805	6.81E-12
At1g44100	AAP5 (amino acid permease 5); amino acid permease 5	-1.336	2.08E-05
At1g44160	HSP40/DnaJ peptide-binding protein	-1.05	0.00016
At1g44350	ILL6 (IAA-leucine resistant (ILR)-like gene 6); encodes a protein similar to IAA amino acid conjugate hydrola	2.519	1.21E-08
At1g44382	pseudogene, similar to putative AP endonuclease/reverse transcriptase, blastp match of 48% identity and 5.3e-	-1.159	4.64E-05
At1g44414	unknown protein	7.399	1.00E-12
At1g44800	nodulin MtN21 /EamA-like transporter family protein	-1.526	4.50E-07
At1g44830	Integrase-type DNA-binding superfamily protein; encodes a member of the DREB subfamily A-5 of ERF/AP2	-1.129	4.56E-05
At1g44970	Peroxidase superfamily protein	1.758	6.65E-05
At1g45145	ATH5 (THIOREDOXIN H-TYPE 5); encodes a cytosolic thioredoxin that reduces disulfide bridges of target p	1.608	0.0008
At1g45249	ABF2 (abscisic acid responsive elements-binding factor 2); Leucine zipper transcription factor that binds to th	-1.337	6.25E-08
At1g45616	AtRLP6 (receptor like protein 6)	-1.829	0.00025
At1g46554	other RNA	-1.587	1.93E-08
At1g46768	RAP2.1 (related to AP2 1); encodes a member of the DREB subfamily A-5 of ERF/AP2 transcription factor fa	-1.708	1.74E-06
At1g47510	5PTASE11 (inositol polyphosphate 5-phosphatase 11); Encodes a phosphatidylinositol polyphosphate 5-phos	1.942	1.28E-06
At1g47630	CYP96A7 (cytochrome P450, family 96, subfamily A, polypeptide 7); member of CYP96A	1.125	1.43E-05
At1g47655	Dof-type zinc finger DNA-binding family protein	-1.031	2.21E-06
At1g48040	Protein phosphatase 2C family protein	1.544	1.85E-08
At1g48260	CIPK17 (CBL-interacting protein kinase 17); Encodes a member of the SNF1-related kinase (SnRK) gene fam	-1.065	0.00019
At1g48330	unknown protein	-1.343	5.05E-07
At1g48405	Kinase interacting (KIP1-like) family protein	4.902	1.30E-08
At1g48700	2-oxoglutarate (2OG) and Fe(II)-dependent oxygenase superfamily protein	4.138	1.02E-11
At1g48720	unknown protein	6.228	4.81E-11
At1g48745	unknown protein	1.911	1.37E-07
At1g48870	Transducin/WD40 repeat-like superfamily protein	2.334	8.89E-10
At1g49050	Eukaryotic aspartyl protease family protein	1.138	9.85E-06
At1g49130	B-box type zinc finger protein with CCT domain	-2.237	0.0016
At1g49200	RING/U-box superfamily protein	-1.772	2.71E-07
At1g49210	RING/U-box superfamily protein	-2.202	8.71E-08
At1g49220	RING/U-box superfamily protein	-1.37	1.69E-06
At1g49230	RING/U-box superfamily protein	-2.104	7.97E-10
At1g49250	ATP-dependent DNA ligase	1.171	9.14E-07
At1g49310	unknown protein	-1.084	0.00059
At1g49370	pseudogene, similar to OSJNBa0072F16.8, blastp match of 42% identity and 6.6e-73 P-value to GP|21739229	-1.977	4.08E-09
At1g49560	Homeodomain-like superfamily protein	1.252	6.88E-07
At1g49570	Peroxidase superfamily protein	1.429	0.00149
At1g49620	KRP7 (KIP-RELATED PROTEIN 7); Kip-related protein (KRP) gene, encodes CDK (cyclin-dependent kinase	1.781	1.88E-08
At1g49650	alpha/beta-Hydrolases superfamily protein	-1.066	2.94E-05
At1g49660	AtCXE5 (carboxyesterase 5); Encodes a protein with carboxylesterase whose activity was tested using pNA.	-1.119	5.17E-07
At1g49710	FUT12 (fucosyltransferase 12); Encodes a protein with core α1,3-fucosyltransferase activity.	1.826	6.08E-10
At1g49790	F-box associated ubiquitination effector family protein	3.416	1.56E-11
At1g49860	ATGSTF14 (glutathione S-transferase (class phi) 14); Encodes glutathione transferase belonging to the phi cla	-1.443	0.00135
At1g50140	P-loop containing nucleoside triphosphate hydrolases superfamily protein	1.184	4.72E-07
At1g50400	Eukaryotic porin family protein	3.058	7.79E-08
At1g50630	Protein of unknown function (DUF3537)	1.718	5.72E-09
At1g50640	ERF3 (ethylene responsive element binding factor 3); encodes a member of the ERF (ethylene response factor)	1.022	8.02E-05
At1g50740	Transmembrane proteins 14C	2.75	1.03E-08
At1g51270	structural molecules	1.443	3.47E-06
At1g51340	MATE efflux family protein	1.931	6.79E-08
At1g51440	alpha/beta-Hydrolases superfamily protein; Encodes a lipase that hydrolyzes phosphatidylcholine, glycolipids a	-1.692	5.15E-09
At1g51530	RNA-binding (RRM/RBD/RNP motifs) family protein	1.319	1.80E-06
At1g51610	Cation efflux family protein	-1.128	4.98E-08
At1g51620	Protein kinase superfamily protein	2.053	4.73E-07
At1g51670	unknown protein	1.334	0.0004
At1g51790	Leucine-rich repeat protein kinase family protein	-1.125	0.00199
At1g51820	Leucine-rich repeat protein kinase family protein	1.396	0.00016
At1g51830	Leucine-rich repeat protein kinase family protein	-1.304	0.00155
At1g51940	protein kinase family protein / peptidoglycan-binding LysM domain-containing protein	-1.405	3.42E-06
At1g52190	Major facilitator superfamily protein	-2.125	9.70E-08
At1g52290	Protein kinase superfamily protein	-1.075	1.50E-06
At1g52560	HSP20-like chaperones superfamily protein	10.796	1.66E-13
At1g52565	unknown protein	1.27	1.16E-07
At1g52690	Late embryogenesis abundant protein (LEA) family protein	4.586	0.00018
At1g52720	unknown protein	-1.176	8.77E-06
At1g52750	alpha/beta-Hydrolases superfamily protein	-1.041	0.00013
At1g52790	2-oxoglutarate (2OG) and Fe(II)-dependent oxygenase superfamily protein; encodes a putative oxidoreductase,	3.01	1.93E-05

At1g52800	2-oxoglutarate (2OG) and Fe(II)-dependent oxygenase superfamily protein	-1.154	0.00083
At1g52830	IAA6 (indole-3-acetic acid 6); An extragenic dominant suppressor of the hy2 mutant phenotype. Also exhibits	-1.605	0.0002
At1g52870	Peroxisomal membrane 22 kDa (Mpv17/PMP22) family protein	2.671	1.73E-08
At1g52890	ANAC019 (NAC domain containing protein 19); encodes a NAC transcription factor whose expression is indu	-2.327	2.48E-05
At1g52940	PAP5 (purple acid phosphatase 5)	-2.383	0.00099
At1g53035	unknown protein	-1.868	1.19E-08
At1g53040	Protein of unknown function (DUF616)	-1.19	8.09E-07
At1g53100	Core-2/I-branching beta-1,6-N-acetylglucosaminyltransferase family protein	-2.064	3.06E-05
At1g53180	unknown protein	1.535	2.28E-06
At1g53230	TCP3 (TEOSINTE BRANCHED 1, cycloidea and PCF transcription factor 3); Encodes a member of a recently	-1.233	6.56E-06
At1g53430	Leucine-rich repeat transmembrane protein kinase	1.793	4.20E-08
At1g53540	HSP20-like chaperones superfamily protein	11.45	3.21E-12
At1g53590	NTMC2T6.1 (Calcium-dependent lipid-binding (CaLB domain) family protein)	1.225	1.96E-08
At1g53625	unknown protein	1.967	8.46E-05
At1g53840	ATPME1 (pectin methylesterase 1); encodes a pectin methylesterase	-1.107	2.56E-07
At1g53870	Protein of unknown function (DUF567)	-2.978	1.73E-09
At1g53885	Protein of unknown function (DUF581)	-1.613	0.00052
At1g53980	Ubiquitin-like superfamily protein	2.189	1.52E-06
At1g54050	HSP20-like chaperones superfamily protein	7.358	5.82E-13
At1g54070	Dormancy/auxin associated family protein	3.824	1.41E-10
At1g54120	unknown protein	-1	5.66E-06
At1g54410	dehydrin family protein	-1.013	1.40E-05
At1g54570	Esterase/lipase/thioesterase family protein	-1.008	0.0005
At1g54660	pseudogene, similar to vetispiradiene synthase, blastp match of 54% identity and 3.0e-79 P-value to GP|53606	-1.262	0.0008
At1g54820	Protein kinase superfamily protein	-1.313	4.31E-07
At1g55152	unknown protein	-1.25	1.54E-05
At1g55230	Family of unknown function (DUF716)	3.587	2.36E-09
At1g55250	HUB2 (histone mono-ubiquitination 2); Encodes one of two orthologous E3 ubiquitin ligases in Arabidopsis th	1.053	4.22E-07
At1g55255	NA	1.058	3.60E-07
At1g55290	2-oxoglutarate (2OG) and Fe(II)-dependent oxygenase superfamily protein; encodes a protein whose sequence	3.227	3.05E-07
At1g55300	TAF7 (TBP-associated factor 7)	1.765	3.00E-10
At1g55310	SCL33 (SC35-LIKE SPLICING FACTOR 33); Encodes a SR spliceosome protein that is localized to nuclear s	2.077	8.40E-11
At1g55380	Cysteine/Histidine-rich C1 domain family protein	-1.834	1.52E-07
At1g55450	S-adenosyl-L-methionine-dependent methyltransferases superfamily protein	1.714	1.37E-05
At1g55525	other RNA; Unknown gene	1.636	9.63E-08
At1g55530	RING/U-box superfamily protein	4.175	2.20E-12
At1g55620	CLC-F (chloride channel F); Encodes a chloride channel protein that has been localized to the chloroplast and	1.459	4.29E-09
At1g55800	Domain of unknown function (DUF2431)	2.753	4.54E-06
At1g55940	CYP708A1 (cytochrome P450, family 708, subfamily A, polypeptide 1); member of CYP708A	-1.029	0.00096
At1g56010	anac021 (Arabidopsis NAC domain containing protein 21); Encodes a transcription factor involved in shoot ap	-1.84	4.28E-06
At1g56060	unknown protein	4.765	3.92E-05
At1g56140	Leucine-rich repeat transmembrane protein kinase	2.019	1.45E-10
At1g56170	NF-YC2 (nuclear factor Y, subunit C2); Encodes a protein with similarity to a subunit of the CCAAT promote	2.141	1.01E-07
At1g56220	Dormancy/auxin associated family protein	-1.64	3.45E-07
At1g56240	AtPP2-B13 (phloem protein 2-B13)	2.202	0.001
At1g56250	AtPP2-B14 (phloem protein 2-B14)	1.955	7.22E-05
At1g56320	BEST Arabidopsis thaliana protein match is: Glycine-rich protein family (TAIR:AT5G49350.2)	3.585	2.72E-09
At1g56350	Peptide chain release factor 2	1.006	6.77E-08
At1g56410	ERD2 (EARLY-RESPONSIVE TO DEHYDRATION 2); encodes a heat shock protein whose gene expression	1.691	4.80E-07
At1g56440	Tetratricopeptide repeat (TPR)-like superfamily protein	2.175	6.54E-10
At1g56600	AtGolS2 (galactinol synthase 2)	1.271	4.23E-05
At1g56710	Pectin lyase-like superfamily protein	-1.83	5.74E-07
At1g57630	Toll-Interleukin-Resistance (TIR) domain family protein	2.434	0.01714
At1g57765	unknown protein	1.098	5.58E-08
At1g57810	pseudogene, similar to putative AP endonuclease/reverse transcriptase, blastp match of 29% identity and 1.1e-	1.732	1.33E-07
At1g57990	ATPUP18 (purine permease 18); Member of a family of proteins related to PUP1, a purine transporter. May be	1.88	1.30E-06
At1g58130	pseudogene, putative heat shock transcription factor HSF30	2.649	5.03E-08
At1g58170	Disease resistance-responsive (dirigent-like protein) family protein	2.078	5.93E-10
At1g58420	Uncharacterised conserved protein UCP031279	1.407	0.0001
At1g58450	Tetratricopeptide repeat (TPR)-like superfamily protein	1.207	1.70E-08
At1g59590	ZCF37; ZCF37 mRNA, complete cds	2.161	8.62E-08
At1g59660	Nucleoporin autopeptidase	1.131	0.00029
At1g59700	ATGSTU16 (glutathione S-transferase TAU 16); Encodes glutathione transferase belonging to the tau class of	1.011	1.55E-05
At1g59850	ARM repeat superfamily protein	1.171	0.00056
At1g59860	HSP20-like chaperones superfamily protein	8.335	5.62E-13
At1g59910	Actin-binding FH2 (formin homology 2) family protein	1.913	1.64E-08
At1g59920	MADS-box family protein	-1.077	5.93E-05

At1g59930	MADS-box family protein	-1.033	1.57E-05
At1g59950	NAD(P)-linked oxidoreductase superfamily protein	1.552	4.56E-05
At1g59980	ARL2 (ARG1-like 2)	2.275	4.65E-10
At1g60010	unknown protein	-1.151	2.47E-07
At1g60140	ATTPS10 (trehalose phosphate synthase); Encodes an enzyme putatively involved in trehalose biosynthesis. T	-1.049	5.08E-06
At1g60190	ARM repeat superfamily protein	1.406	6.89E-07
At1g60450	AtGolS7 (galactinol synthase 7)	-1.347	0.01701
At1g60470	AtGolS4 (galactinol synthase 4)	1.462	6.59E-08
At1g60680	NAD(P)-linked oxidoreductase superfamily protein	-1.134	2.61E-07
At1g60730	NAD(P)-linked oxidoreductase superfamily protein	1.858	2.39E-05
At1g60740	Thioredoxin superfamily protein	1.504	0.00581
At1g60750	NAD(P)-linked oxidoreductase superfamily protein	3.254	2.21E-09
At1g60800	NIK3 (NSP-interacting kinase 3)	-1.033	3.08E-07
At1g60970	SNARE-like superfamily protein	2.967	5.74E-11
At1g61070	LCR66 (low-molecular-weight cysteine-rich 66); Predicted to encode a PR (pathogenesis-related) protein. Bel	-1.399	8.40E-05
At1g61120	GES (GERANYLLINALOOL SYNTHASE); Encodes a geranyllinalool synthase that produces a precursor to T	-1.078	0.03852
At1g61140	EDA16 (embryo sac development arrest 16)	1.977	0.0003
At1g61240	Protein of unknown function (DUF707)	-1.08	4.14E-07
At1g61250	SC3 (secretory carrier 3); Encodes a putative secretory carrier membrane protein (SC3).	1.081	6.18E-07
At1g61255	BEST Arabidopsis thaliana protein match is: glycine-rich protein (TAIR:AT4G21620.2)	-1.161	6.05E-05
At1g61280	Phosphatidylinositol N-acetylglucosaminyltransferase, GPI19/PIG-P subunit	1.312	3.36E-05
At1g61360	S-locus lectin protein kinase family protein	1.086	2.67E-05
At1g61415	unknown protein	1.763	1.01E-08
At1g61460	S-locus protein kinase, putative	1.614	1.49E-08
At1g61470	Polynucleotidyl transferase, ribonuclease H-like superfamily protein	2.908	7.92E-10
At1g61550	S-locus lectin protein kinase family protein	2.268	2.15E-07
At1g61560	ATMLO6 (MILDEW RESISTANCE LOCUS O 6); A member of a large family of seven-transmembrane doma	3.948	2.84E-12
At1g61610	S-locus lectin protein kinase family protein	1.348	4.26E-05
At1g61720	BAN (BANYULS); Negative regulator of flavonoid biosynthesis, mutants accumulate flavonoid pigments in th	2.16	5.81E-10
At1g61740	Sulfite exporter TauE/SafE family protein	-2.396	6.83E-12
At1g62000	unknown protein	1.064	0.0014
At1g62010	Mitochondrial transcription termination factor family protein	1.357	3.22E-07
At1g62030	Cysteine/Histidine-rich C1 domain family protein	-1.068	1.09E-06
At1g62080	unknown protein	2.114	7.29E-07
At1g62090	pseudogene, protein kinase family, similar to light repressible receptor protein kinase (LRRPK) (Arabidopsis t	3.257	3.44E-11
At1g62180	APR2 (5'adenylylphosphosulfate reductase 2); encodes a adenosine 5'-phosphosulfate reductase, involved in su	1.255	7.01E-07
At1g62190	Kua-ubiquitin conjugating enzyme hybrid localisation domain	1.428	2.54E-05
At1g62390	Octicosapeptide/Phox/Bem1p (PB1) domain-containing protein / tetratricopeptide repeat (TPR)-containing pro	1.021	7.06E-07
At1g62400	HT1 (high leaf temperature 1)	-1.778	1.97E-08
At1g62420	Protein of unknown function (DUF506)	1.61	2.40E-08
At1g62480	Vacuolar calcium-binding protein-related	-1.193	0.00014
At1g62560	FMO GS-OX3 (flavin-monooxygenase glucosinolate S-oxygenase 3); belongs to the flavin-monooxygenase (FI	-3.066	1.79E-11
At1g62570	FMO GS-OX4 (flavin-monooxygenase glucosinolate S-oxygenase 4); belongs to the flavin-monooxygenase (FI	1.452	1.01E-05
At1g62600	Flavin-binding monooxygenase family protein	1.875	1.07E-09
At1g62730	Terpenoid synthases superfamily protein	1.947	6.49E-09
At1g62740	stress-inducible protein, putative	2.5	5.48E-10
At1g62770	Plant invertase/pectin methylesterase inhibitor superfamily protein	-2.449	8.56E-08
At1g62800	ASP4 (aspartate aminotransferase 4); Encodes aspartate aminotransferase (Asp4).	-1.836	2.39E-08
At1g62835	pseudogene, similar to putative AP endonuclease/reverse transcriptase, blastp match of 39% identity and 1.2e-	-1.377	5.06E-08
At1g62840	Protein of unknown function (DUF1442)	1.034	1.11E-05
At1g62930	Tetratricopeptide repeat (TPR)-like superfamily protein	-1.019	7.81E-07
At1g63130	Tetratricopeptide repeat (TPR)-like superfamily protein; Transacting siRNA generating locus. Its derived siR9	-1.34	2.97E-07
At1g63250	DEA(D/H)-box RNA helicase family protein	1.072	1.41E-06
At1g63340	Flavin-containing monooxygenase family protein	1.513	4.91E-07
At1g63350	Disease resistance protein (CC-NBS-LRR class) family	1.353	1.86E-07
At1g63360	Disease resistance protein (CC-NBS-LRR class) family	1.201	3.86E-06
At1g63370	Flavin-binding monooxygenase family protein	1.613	1.08E-09
At1g63390	FAD/NAD(P)-binding oxidoreductase family protein	1.583	2.79E-09
At1g63400	Pentatricopeptide repeat (PPR) superfamily protein	-1.013	6.91E-07
At1g63530	BEST Arabidopsis thaliana protein match is: hydroxyproline-rich glycoprotein family protein (TAIR:AT1G635	2.395	2.86E-06
At1g63710	CYP86A7 (cytochrome P450, family 86, subfamily A, polypeptide 7); Encodes a member of the CYP86A subf	-1.171	0.00018
At1g63720	BEST Arabidopsis thaliana protein match is: hydroxyproline-rich glycoprotein family protein (TAIR:AT5G524	1.886	1.22E-07
At1g63750	Disease resistance protein (TIR-NBS-LRR class) family	1.358	0.0001
At1g63820	CCT motif family protein	2.326	4.13E-08
At1g63830	PLAC8 family protein	1.08	3.20E-07
At1g63840	RING/U-box superfamily protein	2.116	4.53E-10
At1g63880	Disease resistance protein (TIR-NBS-LRR class) family; Encodes a TIR-NBS-LRR class of disease resistance	-1.092	6.13E-08

At1g63930	ROH1 (from the Czech 'roh' meaning 'corner')	1.942	1.45E-09
At1g64065	Late embryogenesis abundant (LEA) hydroxyproline-rich glycoprotein family	2.365	0.0015
At1g64107	Putative membrane lipoprotein; Encodes a defensin-like (DEFL) family protein.	1.04	3.02E-07
At1g64110	P-loop containing nucleoside triphosphate hydrolases superfamily protein	2.166	3.53E-07
At1g64195	Defensin-like (DEFL) family protein; Encodes a defensin-like (DEFL) family protein.	5.228	3.78E-10
At1g64200	VHA-E3 (vacuolar H+-ATPase subunit E isoform 3)	2.546	7.16E-11
At1g64330	myosin heavy chain-related	-1.054	1.16E-07
At1g64460	Protein kinase superfamily protein	1.788	4.78E-09
At1g64470	Ubiquitin-like superfamily protein	1.601	1.85E-07
At1g64500	Glutaredoxin family protein	2.089	1.23E-06
At1g64530	Plant regulator RWP-RK family protein	1.152	3.12E-08
At1g64561	unknown protein	2.574	1.59E-09
At1g64563	other RNA	1.582	8.59E-07
At1g64640	ENODL8 (early nodulin-like protein 8)	-1.504	3.90E-08
At1g64690	BLT (BRANCHLESS TRICHOME); Encodes BRANCHLESS TRICHOME (BLT) involved in trichome devel	-1.079	7.81E-06
At1g64730	pseudogene, putative NADH dehydrogenase subunit F, blastp match of 68% identity and 5.3e-08 P-value to G	-1.228	0.00019
At1g64780	AMT1.2 (ammonium transporter 1.2); encodes an ammonium transporter protein believed to act as a high affi	2.224	1.44E-06
At1g64820	MATE efflux family protein	2.212	5.08E-07
At1g64930	CYP89A7 (cytochrome P450, family 87, subfamily A, polypeptide 7); member of CYP89A	1.237	4.15E-07
At1g64940	CYP89A6 (cytochrome P450, family 87, subfamily A, polypeptide 6); member of CYP89A	1.493	1.77E-08
At1g64950	CYP89A5 (cytochrome P450, family 89, subfamily A, polypeptide 5); member of CYP89A	1.782	1.60E-08
At1g64970	G-TMT (gamma-tocopherol methyltransferase); gamma-tocopherol methyltransferase (g-TMT) mRNA, nuclea	1.525	1.16E-07
At1g65010	INVOLVED IN: flower development (Plant protein of unknown function (DUF827)); Putative role in flower de	-1.163	6.25E-08
At1g65040	RING/U-box superfamily protein	1.349	1.90E-09
At1g65240	Eukaryotic aspartyl protease family protein	2.209	3.43E-05
At1g65370	TRAF-like family protein	-1.103	0.00045
At1g65390	ATPP2-A5 (phloem protein 2 A5)	2.376	1.01E-06
At1g65481	unknown protein	-1.227	0.01619
At1g65790	ARK1 (receptor kinase 1); An alternatively spliced gene that encodes a functional transmembrane receptor ser	-2.159	7.31E-07
At1g65860	FMO GS-OX1 (flavin-monooxygenase glucosinolate S-oxygenase 1); belongs to the flavin-monooxygenase (F	-3.797	2.86E-10
At1g65980	TPX1 (thioredoxin-dependent peroxidase 1); thioredoxin-dependent peroxidase	1.041	4.19E-08
At1g65985	Plant protein of unknown function (DUF247)	-1.261	7.79E-08
At1g66060	Family of unknown function (DUF577)	2.355	9.66E-08
At1g66080	unknown protein	4.61	1.19E-11
At1g66090	Disease resistance protein (TIR-NBS class)	5.918	8.17E-08
At1g66160	CMPG1 (CYS, MET, PRO, and GLY protein 1)	2.274	8.80E-08
At1g66180	Eukaryotic aspartyl protease family protein	-1.461	1.24E-05
At1g66345	Pentatricopeptide repeat (PPR) superfamily protein	-1.03	1.98E-08
At1g66350	RGL1 (RGA-like 1); Negative regulator of GA responses, member of GRAS family of transcription factors. A	-1.255	1.18E-07
At1g66480	PMI2 (plastid movement impaired 2); Involved in chloroplast avoidance movement under intermediate and hig	1.17	1.30E-05
At1g66500	Pre-mRNA cleavage complex II	3.674	1.63E-09
At1g66510	AAR2 protein family	3.761	9.15E-12
At1g66550	WRKY67 (WRKY DNA-binding protein 67); member of WRKY Transcription Factor; Group III	1.443	0.00108
At1g66580	RPL10C (ribosomal protein L10 C)	2.457	1.87E-10
At1g66600	ABO3 (ABA overly sensitive mutant 3); A member of WRKY Transcription Factor; Group III. Involved in the	4.348	4.89E-08
At1g66700	PXMT1 (S-adenosyl-L-methionine-dependent methyltransferases superfamily protein); A member of the Arabi	2.374	0.0068
At1g66725	MIR163 (microRNA163); Encodes a microRNA that targets several SAMT family members. miR163, is highl	2.863	7.80E-09
At1g66870	Carbohydrate-binding X8 domain superfamily protein	-1.021	0.02577
At1g66890	FUNCTIONS IN: molecular_function unknown; INVOLVED IN: biological_process unknown; LOCATED IN	-1.238	2.43E-06
At1g67050	unknown protein	-1.285	1.79E-07
At1g67060	unknown protein	1.476	7.52E-08
At1g67100	LBD40 (LOB domain-containing protein 40)	1.316	0.00213
At1g67105	other RNA	1.077	0.00062
At1g67265	DVL3 (DEVIL 3)	-3.25	2.09E-09
At1g67300	Major facilitator superfamily protein	1.219	0.0056
At1g67330	Protein of unknown function (DUF579)	-1.234	4.40E-08
At1g67360	Rubber elongation factor protein (REF)	4.334	3.10E-11
At1g67365	other RNA; Potential natural antisense gene, locus overlaps with AT1G67370	4.712	1.31E-12
At1g67370	ASY1 (ASYNAPTIC 1); meiotic asynaptic mutant 1 (ASY1). ASY1 protein is initially distributed as numerou	2.14	3.46E-07
At1g67390	F-box family protein	1.059	8.96E-07
At1g67440	emb1688 (embryo defective 1688)	1.103	1.77E-07
At1g67460	Minichromosome maintenance (MCM2/3/5) family protein	1.784	4.88E-07
At1g67470	Protein kinase superfamily protein	1.06	4.75E-06
At1g67510	Leucine-rich repeat protein kinase family protein	-1.306	1.05E-08
At1g67600	Acid phosphatase/vanadium-dependent haloperoxidase-related protein	-1.066	7.22E-05
At1g67710	ARR11 (response regulator 11); Encodes an Arabidopsis response regulator (ARR) protein that acts in concert	-1.239	3.19E-07
At1g67800	Copine (Calcium-dependent phospholipid-binding protein) family	1.049	2.07E-05

At1g67810	SUFE2 (sulfur E2); Encodes a protein capable of stimulating the cysteine desulfurase activity of CpNifS (AT1	1.183	0.00027
At1g67850	Protein of unknown function (DUF707)	1.314	2.43E-09
At1g67856	RING/U-box superfamily protein	1.956	2.73E-08
At1g67910	unknown protein	-1.396	4.88E-08
At1g67920	unknown protein	3.838	4.00E-10
At1g68020	TPS6 (TREHALOSE -6-PHOSPHATASE SYNTHASE S6); Encodes an enzyme putatively involved in trehalo	-1.07	1.04E-06
At1g68050	FKF1 (flavin-binding, kelch repeat, f box 1); Encodes FKF1, a flavin-binding kelch repeat F box protein, is clo	-1.006	4.47E-07
At1g68130	AtIDD14 (indeterminate(ID)-domain 14)	-1.34	1.18E-07
At1g68140	Protein of unknown function (DUF1644)	2.042	8.44E-11
At1g68150	WRKY9 (WRKY DNA-binding protein 9); member of WRKY Transcription Factor; Group II-b	1.308	2.44E-06
At1g68190	B-box zinc finger family protein	-1.031	1.56E-05
At1g68200	Zinc finger C-x8-C-x5-C-x3-H type family protein	1.529	1.70E-08
At1g68238	unknown protein	-1.664	8.56E-08
At1g68280	Thioesterase superfamily protein	1.113	1.37E-06
At1g68300	Adenine nucleotide alpha hydrolases-like superfamily protein	2.399	9.25E-10
At1g68310	Protein of unknown function (DUF59); Encodes a protein that has been shown to specifically interact with a se	2.319	2.30E-10
At1g68320	AtMYB62 (myb domain protein 62); putative transcription factor: R2R3-MYB transcription family. Involved	2.018	1.91E-08
At1g68330	unknown protein	1.237	2.75E-06
At1g68350	unknown protein	-1.021	4.08E-05
At1g68360	C2H2 and C2HC zinc fingers superfamily protein	-1.201	9.94E-07
At1g68390	Core-2/I-branching beta-1,6-N-acetylglucosaminyltransferase family protein	2.141	1.41E-08
At1g68440	unknown protein	1.762	2.44E-08
At1g68450	VQ motif-containing protein	2.035	3.47E-08
At1g68500	unknown protein	-1.503	1.37E-06
At1g68520	B-box type zinc finger protein with CCT domain	-1.72	0.0028
At1g68560	ATXYL1 (ALPHA-XYLOSIDASE 1); Encodes a bifunctional alpha-l-arabinofuranosidase/beta-d-xylosidase t	-1.448	7.24E-09
At1g68620	alpha/beta-Hydrolases superfamily protein	3.367	4.51E-06
At1g68630	PLAC8 family protein	2.738	1.31E-06
At1g68690	Protein kinase superfamily protein	1.541	6.54E-07
At1g68740	PHO1 (EXS (ERD1/XPR1/SYG1) family protein); Encodes PHO1;H1, a member of the PHO1 family. Involv	-1.527	9.56E-06
At1g68800	BRC2 (BRANCHED 2); Encodes a TCP transcription factor, closely related to teosinte branched1, arrests axil	-1.392	1.39E-06
At1g69040	ACR4 (ACT domain repeat 4); ACT-domain containing protein involved in feedback regulation of amino acid	-1.184	4.80E-06
At1g69330	RING/U-box superfamily protein	-1.126	2.20E-08
At1g69410	ATELF5A-3 (EUKARYOTIC ELONGATION FACTOR 5A-3); Encodes eIF5A-2, a putative eukaryotic transl	1.18	3.00E-05
At1g69530	ATEXPA1 (expansin A1); Member of Alpha-Expansin Gene Family. Naming convention from the Expansin W	-2.143	2.21E-09
At1g69545	NA	-1.326	1.11E-07
At1g69572	other RNA; Potential natural antisense gene, locus overlaps with AT1G69570	-2.4	1.55E-06
At1g69580	Homeodomain-like superfamily protein	-1.103	5.90E-06
At1g69600	ATHB29 (ARABIDOPSIS THALIANA HOMEOBOX PROTEIN 29); Encodes ZFHD1, a member of the zinc	1.269	7.95E-07
At1g69640	SBH1 (sphingoid base hydroxylase 1); Encodes one of the two redundant sphingoid base hydroxylases (SBH).	1.418	1.65E-07
At1g69650	TRAF-like family protein	1.613	5.24E-06
At1g69660	TRAF-like family protein	1.777	1.83E-08
At1g69680	Mog1/PsbP/DUF1795-like photosystem II reaction center PsbP family protein	1.596	2.06E-07
At1g69730	Wall-associated kinase family protein	2.802	6.45E-10
At1g69750	ATCOX19-2 (A. THALIANA CYTOCHROME C OXIDASE 19-2)	1.025	2.76E-07
At1g69790	Protein kinase superfamily protein	1.619	3.57E-07
At1g69820	GGT3 (gamma-glutamyl transpeptidase 3); Note that conflicting nomenclature exists in the literature: At1g698	1.82	1.08E-07
At1g69830	AMY3 (alpha-amylase-like 3); Encodes a plastid-localized α-amylase. Expression is reduced in the SEX	-1.115	6.41E-07
At1g69840	SPFH/Band 7/PHB domain-containing membrane-associated protein family	1.487	0.0122
At1g69870	NRT1.7 (nitrate transporter 1.7); Encodes a low affinity nitrate transporter NRT1.7. Expressed in phloem. Re	-1.819	2.33E-05
At1g69900	Actin cross-linking protein	1.434	5.60E-06
At1g69920	ATGSTU12 (ARABIDOPSIS THALIANA GLUTATHIONE S-TRANSFERASE TAU 12); Encodes glutathio	2.042	3.79E-05
At1g70020	Protein of unknown function (DUF1163)	-1.004	4.53E-05
At1g70140	ATFH8 (formin 8); Encodes a group I formin. Binds to F-actin barbed ends. Has severing actin filaments activ	1.24	1.55E-05
At1g70290	TPS8 (trehalose-6-phosphatase synthase S8); Encodes an enzyme putatively involved in trehalose biosynthesis	-1.392	2.06E-05
At1g70300	KUP6 (K+ uptake permease 6); potassium transporter	1.478	2.85E-07
At1g70530	CRK3 (cysteine-rich RLK (RECEPTOR-like protein kinase) 3); Encodes a cysteine-rich receptor-like protein k	1.614	1.43E-08
At1g70560	SAV3 (SHADE AVOIDANCE 3); TAA1 is involved in the shade-induced production of indole-3-pyruvate (IP	-1.015	5.20E-08
At1g70590	F-box family protein	1.313	8.65E-08
At1g70610	ATTAP1 (transporter associated with antigen processing protein 1); member of TAP subfamily	1.147	0.0029
At1g70690	HWI1 (HOPW1-1-INDUCED GENE1); Encodes a plasmodesmal protein that may be involved in the intercell	-1.282	5.64E-05
At1g70820	phosphoglucomutase, putative / glucose phosphomutase, putative	-1.82	1.93E-08
At1g70890	MLP43 (MLP-like protein 43)	-1.21	2.26E-06
At1g70920	ATHB18 (homeobox-leucine zipper protein 18)	-1.163	1.79E-07
At1g70940	ATPIN3 (ARABIDOPSIS PIN-FORMED 3); A regulator of auxin efflux and involved in differential growth. P	-1.283	5.40E-07
At1g71000	Chaperone DnaJ-domain superfamily protein	8.672	6.22E-09
At1g71030	ATMYBL2 (ARABIDOPSIS MYB-LIKE 2); Encodes a putative myb family transcription factor. In contrast t	-1.916	3.21E-06

At1g71170	6-phosphogluconate dehydrogenase family protein	1.246	1.59E-08
At1g71230	CSN5B (COP9-signalosome 5B); Encodes a subunit of the COP9 complex, similar to JAB1, a specific mamm	2.428	4.43E-10
At1g71250	GDSL-like Lipase/Acylhydrolase superfamily protein	2.912	7.16E-11
At1g71390	AtRLP11 (receptor like protein 11)	-1.241	0.01952
At1g71520	Integrase-type DNA-binding superfamily protein; encodes a member of the DREB subfamily A-5 of ERF/AP2	5.396	3.43E-11
At1g71530	Protein kinase superfamily protein	2.864	8.37E-09
At1g71697	ATCK1 (choline kinase 1); Encodes choline kinase. mRNA levels are increased in response to wounding.	1.871	1.20E-07
At1g71710	DNAse I-like superfamily protein	-1.085	2.24E-07
At1g71770	PAB5 (poly(A)-binding protein 5); Encodes a Class I polyA-binding protein. Expressed in floral organs. Binds	2.216	5.35E-07
At1g71910	unknown protein	-1.171	6.96E-06
At1g71970	unknown protein	-1.69	9.99E-09
At1g72060	serine-type endopeptidase inhibitors	1.174	8.98E-05
At1g72070	Chaperone DnaJ-domain superfamily protein	-1.067	2.53E-05
At1g72100	late embryogenesis abundant domain-containing protein / LEA domain-containing protein	3.445	4.10E-12
At1g72130	Major facilitator superfamily protein	-1.224	3.47E-07
At1g72141	unknown protein	1.815	3.63E-07
At1g72150	PATL1 (PATELLIN 1); novel cell-plate-associated protein that is related in sequence to proteins involved in m	-1.941	1.61E-05
At1g72230	Cupredoxin superfamily protein	-1.388	2.08E-05
At1g72280	AERO1 (endoplasmic reticulum oxidoreductins 1); endoplasmic reticulum oxidoreductin	2.259	9.17E-09
At1g72416	Chaperone DnaJ-domain superfamily protein	2.622	8.66E-10
At1g72530	plastid developmental protein DAG, putative	1.284	1.62E-07
At1g72600	hydroxyproline-rich glycoprotein family protein	-1.218	1.05E-06
At1g72620	alpha/beta-Hydrolases superfamily protein	-1.268	4.15E-05
At1g72660	P-loop containing nucleoside triphosphate hydrolases superfamily protein	8.028	1.31E-14
At1g72680	ATCAD1 (CINNAMYL ALCOHOL DEHYDROGENASE 1)	1.227	1.52E-06
At1g72750	ATTIM23-2 (translocase inner membrane subunit 23-2)	1.493	5.48E-09
At1g72760	Protein kinase superfamily protein	1.015	6.87E-06
At1g72770	HAB1 (homology to ABI1); mutant has ABA hypersensitive inhibition of seed germination; Protein Phosphata	1.001	9.29E-06
At1g72800	RNA-binding (RRM/RBD/RNP motifs) family protein	1.043	0.00034
At1g72890	Disease resistance protein (TIR-NBS class)	-1.482	2.83E-07
At1g72930	TIR (toll/interleukin-1 receptor-like); Toll/interleukin-1 receptor-like protein (TIR) mRNA,	1.375	5.53E-05
At1g72940	Toll-Interleukin-Resistance (TIR) domain-containing protein	2.443	7.60E-08
At1g73010	ATPS2 (phosphate starvation-induced gene 2)	-2.209	0.0026
At1g73040	Mannose-binding lectin superfamily protein	1.025	0.00397
At1g73080	ATPEPR1 (PEP1 RECEPTOR 1); Encodes a leucine-rich repeat receptor kinase. Functions as a receptor for A	1.022	4.70E-07
At1g73220	1-Oct (organic cation/carnitine transporter1)	-2.258	0.00189
At1g73250	ATFX (ACTIVATING TRANSCRIPTION FACTOR 5); encodes a bifunctional 3, 5-epimerase-4-reductase in	1.507	6.03E-09
At1g73260	ATKTI1 (ARABIDOPSIS THALIANA KUNITZ TRYPSIN INHIBITOR 1); Encodes a trypsin inhibitor involv	-1.888	0.00981
At1g73340	Cytochrome P450 superfamily protein	2.836	8.37E-12
At1g73380	unknown protein	1.489	3.31E-08
At1g73480	alpha/beta-Hydrolases superfamily protein	1.424	7.78E-06
At1g73600	S-adenosyl-L-methionine-dependent methyltransferases superfamily protein	-1.824	3.09E-07
At1g73670	ATMPK15 (MAP kinase 15); member of MAP Kinase	1.115	2.39E-05
At1g73690	AT.CDKD.1 (CYCLIN-DEPENDENT KINASE D1); cyclin dependent kinase activator CDKD;1. Nuclear loca	2.227	1.24E-09
At1g73700	MATE efflux family protein	1.263	2.84E-06
At1g73730	ATSLIM (ARABIDOPSIS THALIANA SULFUR LIMITATION 1); Encodes a putative transcription factor in	1.491	1.40E-08
At1g73740	UDP-Glycosyltransferase superfamily protein	3.775	2.25E-11
At1g73750	Uncharacterised conserved protein UCP031088, alpha/beta hydrolase	1.015	0.00015
At1g73810	Core-2/I-branching beta-1,6-N-acetylglucosaminyltransferase family protein	3.228	0.00016
At1g73830	BEE3 (BR enhanced expression 3)	-1.324	1.60E-07
At1g74055	unknown protein	-1.209	7.45E-06
At1g74080	ATMYB122 (MYB DOMAIN PROTEIN 122); Encodes a putative transcription factor, member of the R2R3 f	-1.081	0.0456
At1g74090	ATSOT18 (DESULFO-GLUCOSINOLATE SULFOTRANSFERASE 18); encodes a desulfoglucosinolate sulf	-2.952	3.22E-11
At1g74200	AtRLP16 (receptor like protein 16)	-1.023	2.53E-07
At1g74240	Mitochondrial substrate carrier family protein	1.331	3.57E-08
At1g74310	ATHSP101 (heat shock protein 101); Encodes ClpB1, which belongs to the Casein lytic proteinase/heat shock	5.876	8.14E-12
At1g74320	Protein kinase superfamily protein; encodes a choline kinase, whose expression is induced by high salt and ma	2.613	3.69E-10
At1g74330	Protein kinase superfamily protein	2.1	1.41E-09
At1g74360	Leucine-rich repeat protein kinase family protein	1.404	0.00049
At1g74450	Protein of unknown function (DUF793)	1.081	7.10E-06
At1g74458	unknown protein	-1.417	6.82E-07
At1g74590	ATGSTU10 (GLUTATHIONE S-TRANSFERASE TAU 10); Encodes glutathione transferase belonging to the	1.767	0.00062
At1g74660	MIF1 (mini zinc finger 1); Constitutive overexpression of MIF1 caused dramatic developmental defects, seedli	-1.277	2.21E-07
At1g74670	Gibberellin-regulated family protein	-2.424	0.0002
At1g74740	CDPK1A (CALCIUM-DEPENDENT PROTEIN KINASE 1A); member of Calcium Dependent Protein Kinase	1.317	2.51E-08
At1g74810	BOR5 (HCO3- transporter family)	1.363	5.39E-05
At1g74870	RING/U-box superfamily protein	2.027	2.45E-07

At1g74930	ORA47 (Integrase-type DNA-binding superfamily protein); encodes a member of the DREB subfamily A-5 of	-1.415	5.81E-06
At1g75020	LPAT4 (lysophosphatidyl acyltransferase 4)	1.012	0.0002
At1g75270	DHAR2 (dehydroascorbate reductase 2)	3.046	3.28E-09
At1g75280	NmrA-like negative transcriptional regulator family protein; isoflavone reductase, putative, identical to SP:P52	3.252	9.54E-11
At1g75300	NmrA-like negative transcriptional regulator family protein; encodes a protein whose sequence is similar to an	1.632	6.36E-06
At1g75360	unknown protein	-1.232	1.48E-06
At1g75380	ATBBD1 (bifunctional nuclease in basal defense response 1); Encodes a nucleases AtBBD1 involved in ABA-	-1.11	0.0004
At1g75500	WAT1 (Walls Are Thin 1); An Arabidopsis thaliana homolog of Medicago truncatula NODULIN21 (MtN21).	-1.001	0.0003
At1g75780	TUB1 (tubulin beta-1 chain); beta tubulin gene downregulated by phytochrome A (phyA)-mediated far-red ligl	-1.037	0.0037
At1g75810	unknown protein	1.648	3.80E-10
At1g75820	CLV1 (CLAVATA 1); Putative receptor kinase with an extracellular leucine-rich domain. Controls shoot and f	-1.008	2.07E-06
At1g75880	SGNH hydrolase-type esterase superfamily protein	-2.314	2.60E-07
At1g75900	GDSL-like Lipase/Acylhydrolase superfamily protein	-1.543	6.80E-05
At1g75960	AMP-dependent synthetase and ligase family protein	-1.37	0.0006
At1g76070	unknown protein	4.738	2.13E-12
At1g76160	sks5 (SKU5 similar 5)	-1.144	8.53E-05
At1g76430	PHT1.9 (phosphate transporter 1.9); Encodes Pht1;9, a member of the Pht1 family of phosphate transporters w	-1.137	0.0032
At1g76470	NAD(P)-binding Rossmann-fold superfamily protein	2.031	1.68E-05
At1g76530	Auxin efflux carrier family protein	2.347	1.69E-09
At1g76600	unknown protein	1.718	1.94E-06
At1g76640	Calcium-binding EF-hand family protein	1.309	0.02293
At1g76650	CML38 (calmodulin-like 38)	1.316	0.00594
At1g76680	ATOPR1 (ARABIDOPSIS 12-OXOPHYTODIENOATE REDUCTASE 1); Encodes a member of an alpha/bet	3.391	2.13E-08
At1g76690	ATOPR2 (ARABIDOPSIS 12-OXOPHYTODIENOATE REDUCTASE 2); Encodes one of the closely related	1.418	1.02E-05
At1g76700	DNAJ heat shock N-terminal domain-containing protein	1.465	9.85E-10
At1g76760	ATY1 (thioredoxin Y1); Encodes a y-type thioredoxin (Trx-y1) localized in chloroplast stroma.	1.167	1.40E-06
At1g76800	Vacuolar iron transporter (VIT) family protein	-1.286	6.06E-07
At1g76878	other RNA; Potential natural antisense gene, locus overlaps with AT1G76880	2.392	4.32E-09
At1g76890	GT2 (Duplicated homeodomain-like superfamily protein); encodes a plant trihelix DNA-binding protein	-1.374	3.59E-07
At1g76900	AtTLP1 (tubby like protein 1); Member of TLP family	1.065	1.29E-07
At1g76965	FUNCTIONS IN: molecular_function unknown; INVOLVED IN: biological_process unknown; LOCATED IN	4.443	7.74E-12
At1g76970	Target of Myb protein 1	2.298	3.27E-10
At1g76980	BEST Arabidopsis thaliana protein match is: embryo defective 2170 (TAIR:AT1G21390.1)	1.888	3.30E-07
At1g77000	ATSKP2.2 (ARABIDOPSIS HOMOLOG OF HOMOLOG OF HUMAN SKP2 2); AtSKP2;2 is a homolog of h	2.355	0.0185
At1g77145	Protein of unknown function (DUF506)	-1.012	0.00038
At1g77210	AtSTP14 (sugar transport protein 14); AtSTP14 belongs to the family of sugar transport proteins (AtSTPs)in v	-2.507	1.68E-08
At1g77330	2-oxoglutarate (2OG) and Fe(II)-dependent oxygenase superfamily protein; similar to 1-aminocyclopropane-1-	-1.02	4.44E-05
At1g77390	CYCA1 (CYCLIN A1); Encodes a core cell cycle gene involved in meiosis II during microsporogenesis. Reces	1.285	6.44E-08
At1g77410	BGAL16 (beta-galactosidase 16)	3.06	8.23E-11
At1g77450	anac032 (NAC domain containing protein 32)	2.801	3.64E-09
At1g77460	Armadillo/beta-catenin-like repeat	2.531	7.07E-07
At1g77530	O-methyltransferase family protein	2.249	2.54E-08
At1g77570	Winged helix-turn-helix transcription repressor DNA-binding	3.09	4.04E-09
At1g77590	LACS9 (long chain acyl-CoA synthetase 9); Encodes major plastidic long chain acyl-CoA synthetase with a sli	1.258	1.02E-07
At1g77640	Integrase-type DNA-binding superfamily protein; encodes a member of the DREB subfamily A-5 of ERF/AP2	-1.53	6.68E-06
At1g77760	NIA1 (nitrate reductase 1); Encodes the cytosolic minor isoform of nitrate reductase (NR). Involved in the first	-1.41	3.27E-07
At1g77770	Protein of unknown function (DUF1644)	1.065	9.36E-08
At1g77870	MUB5 (membrane-anchored ubiquitin-fold protein 5 precursor)	-1.153	9.81E-07
At1g78000	SEL1 (SELENATE RESISTANT 1); Encodes a sulfate transporter that can restore sulfate uptake capacity of a	1.303	0.0021
At1g78160	APUM7 (pumilio 7); Encodes a member of the Arabidopsis Pumilio (APUM) proteins containing PUF domain	1.494	2.22E-05
At1g78170	unknown protein	-1.361	7.06E-08
At1g78230	Outer arm dynein light chain 1 protein	-1.64	0.0002
At1g78240	QUA2 (QUASIMODO2); Encodes TSD2 (TUMOROUS SHOOT DEVELOPMENT2), a putative methyltransf	-1.1	1.16E-06
At1g78310	VQ motif-containing protein	2.297	1.73E-09
At1g78340	ATGSTU22 (glutathione S-transferase TAU 22); Encodes glutathione transferase belonging to the tau class of	1.137	0.00063
At1g78370	ATGSTU20 (glutathione S-transferase TAU 20); Encodes glutathione transferase belonging to the tau class of	-1.037	9.30E-07
At1g78380	ATGSTU19 (A. THALIANA GLUTATHIONE S-TRANSFERASE TAU 19); Encodes a glutathione transferas	1.781	2.54E-08
At1g78390	ATNCED9 (NINE-CIS-EPOXYCAROTENOID DIOXYGENASE 9); Encodes 9-cis-epoxycarotenoid dioxyge	2.396	5.28E-10
At1g78400	Pectin lyase-like superfamily protein	2.356	6.97E-08
At1g78410	VQ motif-containing protein	2.712	3.96E-07
At1g78450	SOUL heme-binding family protein	-1.539	1.02E-05
At1g78510	SPS1 (solanesyl diphosphate synthase 1); Encodes a protein with solanesyl diphosphate synthase activity.	1.016	3.90E-06
At1g78530	Protein kinase superfamily protein	-1.433	5.47E-05
At1g78830	Curculin-like (mannose-binding) lectin family protein	-1.373	9.56E-05
At1g78895	Reticulon family protein	1.501	2.50E-09
At1g78970	ATLUP1 (ARABIDOPSIS THALIANA LUPEOL SYNTHASE 1); Lupeol synthase. Converts oxidosqualene t	-1.935	2.28E-07
At1g78995	unknown protein	-1.337	6.86E-08

At1g79110	zinc ion binding	-1.446	1.03E-06
At1g79245	expressed protein, Several heterogeneous transcript sequences map to this position; pseudogene of unknown p	1.266	7.75E-05
At1g79270	ECT8 (evolutionarily conserved C-terminal region 8)	1.177	0.0001
At1g79460	ATKS (ARABIDOPSIS THALIANA ENT-KAURENE SYNTHASE); Encodes for a protein with ent-kaurene	-1.192	4.73E-06
At1g79510	Uncharacterized conserved protein (DUF2358)	1.131	1.77E-06
At1g79550	PGK (phosphoglycerate kinase); Encodes cytosolic phosphoglycerate kinase (PGK).	1.368	1.76E-06
At1g79680	WAKL10 (WALL ASSOCIATED KINASE (WAK)-LIKE 10)	3.112	6.21E-07
At1g79700	Integrase-type DNA-binding superfamily protein	-1.117	0.0002
At1g79710	Major facilitator superfamily protein	1.086	1.07E-06
At1g79770	Protein of unknown function (DUF1677)	-2.006	1.12E-07
At1g79800	ENODL7 (early nodulin-like protein 7)	1.207	0.0047
At1g79920	Heat shock protein 70 (Hsp 70) family protein	2.228	1.06E-09
At1g80120	Protein of unknown function (DUF567)	1.185	4.11E-06
At1g80130	Tetratricopeptide repeat (TPR)-like superfamily protein	-1.355	0.0035
At1g80310	sulfate transmembrane transporters	-1.106	1.18E-06
At1g80440	Galactose oxidase/kelch repeat superfamily protein	-1.569	3.73E-06
At1g80470	F-box/RNI-like/FBD-like domains-containing protein	1.997	5.00E-08
At1g80650	RTL1 (RNAse THREE-like protein 1)	-1.007	2.20E-06
At1g80670	Transducin/WD40 repeat-like superfamily protein; This gene is predicted to encode a protein with a DWD mo	1.089	0.0006
At1g80820	CCR2 (cinnamoyl coa reductase); Encodes an cinnamoyl CoA reductase isoform. Involved in lignin biosynthe	2.941	2.40E-07
At1g80840	WRKY40 (WRKY DNA-binding protein 40); Pathogen-induced transcription factor. Binds W-box sequences	1.479	0.0081
At1g80920	J8 (Chaperone DnaJ-domain superfamily protein); A nuclear encoded soluble protein found in the chloroplast s	-1.132	6.03E-06
At1g80940	unknown protein	-1.006	3.40E-07
At2g01020	5SrRNA (rRNA)	-1.474	0.00888
At2g01180	ATPAP1 (phosphatidic acid phosphatase 1); Encodes phosphatidate phosphatase. Up-regulated by genotoxic s	2.374	1.31E-07
At2g01200	IAA32 (indole-3-acetic acid inducible 32); Belongs to auxin inducible gene family.	1.224	5.01E-06
At2g01370	DNA-binding storekeeper protein-related transcriptional regulator	1.366	1.24E-07
At2g01530	MLP329 (MLP-like protein 329)	-2	0.00133
At2g01600	ENTH/ANTH/VHS superfamily protein	1.275	1.00E-06
At2g01670	atnudt17 (nudix hydrolase homolog 17)	-1.455	1.83E-08
At2g01740	Tetratricopeptide repeat (TPR)-like superfamily protein	1.578	2.26E-07
At2g01880	ATPAP7 (PURPLE ACID PHOSPHATASE 7)	-1.164	0.00105
At2g01890	ATPAP8 (PURPLE ACID PHOSPHATASE 8); Encodes a purple acid phosphatase (PAP) belonging to the lov	-1.594	2.24E-06
At2g02010	GAD4 (glutamate decarboxylase 4)	2.524	4.04E-07
At2g02020	Major facilitator superfamily protein	-1.637	1.03E-07
At2g02080	AtIDD4 (indeterminate(ID)-domain 4)	-1.412	1.34E-08
At2g02100	LCR69 (low-molecular-weight cysteine-rich 69); Predicted to encode a PR (pathogenesis-related) protein. Bel	-1.417	6.43E-06
At2g02130	LCR68 (low-molecular-weight cysteine-rich 68); Predicted to encode a PR (pathogenesis-related) protein. Bel	-1.007	1.01E-05
At2g02220	ATPSKR1 (PHYTOSULFOKIN RECEPTOR 1); Encodes a protein interacting with phytosulfokine, a five ami	1.996	4.85E-09
At2g02750	Pentatricopeptide repeat (PPR) superfamily protein	1.453	3.28E-06
At2g02860	ATSUC3 (ARABIDOPSIS THALIANA SUCROSE TRANSPORTER 3); encodes a sucrose transporter in siev	1.397	7.68E-07
At2g02950	PKS1 (phytochrome kinase substrate 1); Encodes a basic soluble protein which can independently bind to eith	-1.452	9.06E-07
At2g02980	OTP85 (ORGANELLE TRANSCRIPT PROCESSING 85); Encodes a chloroplast RNA editing factor.	-1.098	1.31E-06
At2g02990	ATRNS1 (RIBONUCLEASE 1); member of the ribonuclease T2 family, responds to inorganic phosphate starv	-1.524	0.02978
At2g03290	emp24/gp25L/p24 family/GOLD family protein	1.152	0.00032
At2g03310	unknown protein	-1.942	3.53E-07
At2g03440	NRP1 (nodulin-related protein 1); Induced at the transcriptional level by Pseudomonas syringae pv. tomato inf	-1.253	5.71E-07
At2g03505	Carbohydrate-binding X8 domain superfamily protein	1.501	8.83E-07
At2g03570	unknown protein	-1.888	8.29E-06
At2g03750	P-loop containing nucleoside triphosphate hydrolases superfamily protein	-1.103	2.33E-06
At2g03760	AtSOT12 (sulphotransferase 12); Encodes a brassinosteroid sulfotransferase. In vitro experiements show that t	1.713	8.92E-05
At2g03890	ATPI4K GAMMA 7 (phosphoinositide 4-kinase gamma 7); Phosphoinositide kinase which undergo autophos	-1.267	1.07E-07
At2g03980	GDSL-like Lipase/Acylhydrolase superfamily protein	-1.104	1.63E-06
At2g04039	unknown protein	1.407	2.87E-07
At2g04160	AIR3 (AUXIN-INDUCED IN ROOT CULTURES 3); isolated from differential screening of a cDNA library fr	-1.074	0.0005
At2g04250	pseudogene, hypothetical protein	1.339	1.21E-09
At2g04350	LACS8 (long-chain acyl-CoA synthetase 8)	1.921	1.32E-08
At2g04380	unknown protein	2.492	1.35E-10
At2g04520	Nucleic acid-binding, OB-fold-like protein	1.371	4.41E-07
At2g04570	GDSL-like Lipase/Acylhydrolase superfamily protein	-1.39	0.00013
At2g04600	similar to unknown protein [Arabidopsis thaliana] (TAIR:AT2G13865.1)	2.785	7.29E-06
At2g04622	unknown protein	-1.026	0.04395
At2g05050	Protein phosphatase 2C family protein	1.492	7.75E-09
At2g05160	CCCH-type zinc fingerfamily protein with RNA-binding domain	-1.5	9.38E-07
At2g05180	CYP705A6 (cytochrome P450, family 705, subfamily A, polypeptide 6); member of CYP705A	1.497	1.20E-07
At2g05300	pseudogene of the F-box protein family ; pseudogene of the F-box protein family	1.684	4.61E-08
At2g05518	Unknown gene; Unknown gene	-1.856	6.37E-07

At2g05540	Glycine-rich protein family	-1.774	2.99E-05
At2g06002	other RNA	-1.967	3.15E-05
At2g06530	VPS2.1 (SNF7 family protein)	1.511	1.85E-07
At2g06820	similar to unknown protein [Arabidopsis thaliana] (TAIR:AT4G09700.1); similar to 80C09_29 [Brassica rapa	-1.023	0.00447
At2g06850	EXGT-A1 (endoxyloglucan transferase A1); endoxyloglucan transferase (EXGT-A1) gene	-1.273	0.0002
At2g07600	pseudogene, NADH dehydrogenase, blastp match of 71% identity and 8.1e-159 P-value to GP|17932795|emb|	-1.298	2.82E-06
At2g07680	ATMRP11 (multidrug resistance-associated protein 11); member of MRP subfamily	-1.188	1.44E-07
At2g07719	Putative membrane lipoprotein	1.122	0.00365
At2g07721	unknown protein	1.07	6.39E-07
At2g07731	pseudogene, similar to NADH-ubiquinone oxidoreductase chain 6 (EC 1.6.5.3). (Field mustard), blastp match	4.417	1.19E-10
At2g07750	DEA(D/H)-box RNA helicase family protein	1.123	2.27E-06
At2g07768	Cytochrome C assembly protein	1.078	0.00049
At2g07774	unknown protein	-1.326	6.69E-07
At2g07786	pseudogene, similar to NADH dehydrogenase (ubiquinone), blastp match of 91% identity and 9.4e-39 P-value	1.128	0.00066
At2g07815	BEST Arabidopsis thaliana protein match is: cytochrome C biogenesis 452 (TAIR:ATMG00180.1)	-1.351	9.31E-08
At2g09992	pseudogene of disease-resistance protein; pseudogene of disease-resistance protein	1.503	1.75E-05
At2g09994	pseudogene of U-box domain-containing protein / armadillo/beta-catenin repeat family protein; pseudogene of	1.826	3.89E-07
At2g10606	MIR396A (microRNA396A); Encodes a microRNA that targets several GRF family members. MicroRNAs are	-1.504	1.29E-07
At2g11150	pseudogene, expressed protein (transposable element gene); pseudogene of putative replication protein A1	-1.064	2.70E-05
At2g11270	citrate synthase-related	1.401	2.06E-07
At2g11280	pseudogene, hypothetical protein	1.643	2.81E-09
At2g11810	MGD3 (MONOGALACTOSYL DIACYLGLYCEROL SYNTHASE 3); MGD3 is the major enzyme for galac	-2.805	0.00022
At2g13790	ATSERK4 (SOMATIC EMBRYOGENESIS RECEPTOR-LIKE KINASE 4)	2.023	3.18E-06
At2g13820	Bifunctional inhibitor/lipid-transfer protein/seed storage 2S albumin superfamily protein	-1.102	1.01E-05
At2g13851	unknown pseudogene; unknown pseudogene	-1.206	8.55E-07
At2g14270	Cytochrome C oxidase polypeptide VIB family protein	1.029	0.0173
At2g14550	pseudogene, putative pre-mRNA splicing factor, non-consensus splice donor TA at exon 5	1.769	2.15E-08
At2g14580	ATPRB1 (basic pathogenesis-related protein 1); pathogenesis related protein, encodes a basic PR1-like protein	-1.594	8.56E-06
At2g14610	ATPR1 (PATHOGENESIS-RELATED GENE 1); PR1 gene expression is induced in response to a variety of p	-2.447	0.00491
At2g14750	AKN1 (APS KINASE 1); Encodes adenosine-5'-phosphosulfate kinase. Provides activated sulfate for sulfation	-1.303	6.47E-06
At2g14890	AGP9 (arabinogalactan protein 9); putative proline-rich protein (At2g14890) mRNA, complete	-1.058	4.27E-05
At2g15020	unknown protein	2.745	1.04E-06
At2g15040	AtRLP18 (receptor like protein 18)	-1.129	0.00025
At2g15050	LTP (lipid transfer protein); Predicted to encode a PR (pathogenesis-related) protein. Belongs to the lipid trans	-1.103	1.57E-06
At2g15080	AtRLP19 (receptor like protein 19)	-1.471	2.42E-07
At2g15120	pseudogene, disease-resistance family protein / fatty acid elongase-related, similar to fatty acid elongase 1 (FA	-1.069	3.07E-05
At2g15390	FUT4 (fucosyltransferase 4); Predicted fucosyltransferase, based on similarity to FUT1, but not functionally re	1.758	1.39E-06
At2g15480	UGT73B5 (UDP-glucosyl transferase 73B5)	2.594	4.97E-09
At2g15490	UGT73B4 (UDP-glycosyltransferase 73B4)	3.972	9.79E-10
At2g15620	ATHNIR (ARABIDOPSIS THALIANA NITRITE REDUCTASE); Involved in the second step of nitrate assim	-1.527	3.61E-06
At2g15680	Calcium-binding EF-hand family protein	-1.271	1.01E-07
At2g15790	CYP40 (CYCLOPHILIN 40); SQN encodes the Arabidopsis homolog of cyclophilin 40 (CyP40). It is specific	1.306	3.00E-05
At2g15830	unknown protein	-1.249	5.23E-07
At2g15890	MEE14 (maternal effect embryo arrest 14)	-2.128	3.35E-07
At2g15970	ATCOR413-PM1 (ARABIDOPSIS THALIANA COLD-REGULATED413 PLASMA MEMBRANE 1); encod	-1.667	2.99E-07
At2g16367	Encodes a defensin-like (DEFL) family protein.; Encodes a defensin-like (DEFL) family protein.	1.759	0.00293
At2g16430	PAP10 (purple acid phosphatase 10)	-1.17	1.35E-06
At2g16505	Maternally expressed gene (MEG) family protein; Encodes a Maternally expressed gene (MEG) family protein	2.205	9.48E-08
At2g16750	Protein kinase protein with adenine nucleotide alpha hydrolases-like domain	-1.887	1.09E-08
At2g16900	Arabidopsis phospholipase-like protein (PEARLI 4) family	1.305	4.10E-08
At2g17010	Mechanosensitive ion channel family protein	2.634	2.32E-11
At2g17120	LYM2 (lysm domain GPI-anchored protein 2 precursor)	1.498	0.0001
At2g17140	Pentatricopeptide repeat (PPR) superfamily protein	-1.029	1.40E-07
At2g17180	C2H2-like zinc finger protein	2.071	1.21E-09
At2g17220	Protein kinase superfamily protein	1.147	1.23E-07
At2g17330	CYP51A1 (CYTOCHROME P450 51A1); putative obtusifoliol 14-alpha demethylase. Expressed pseudogene.	2.828	2.38E-11
At2g17430	MLO7 (MILDEW RESISTANCE LOCUS O 7); A member of a large family of seven-transmembrane domain	1.921	1.38E-09
At2g17450	RHA3A (RING-H2 finger A3A); Encodes a putative RING-H2 finger protein RHA3a.	-1.406	9.73E-09
At2g17480	ATMLO8 (MILDEW RESISTANCE LOCUS O 8); A member of a large family of seven-transmembrane doma	1.043	1.43E-06
At2g17500	Auxin efflux carrier family protein	1.134	9.10E-05
At2g17705	unknown protein	1.478	5.59E-08
At2g17787	unknown protein	1.186	0.0001
At2g17880	Chaperone DnaJ-domain superfamily protein	-2.043	7.42E-08
At2g17900	SDG37 (SET domain group 37); Homology Subgroup S-ET - Protein containing an interrupted SET domain.	3.718	6.64E-12
At2g17950	WUS (WUSCHEL); Homeobox gene controlling the stem cell pool. Expressed in the stem cell organizing cent	1.366	9.31E-08
At2g18130	ATPAP11 (PURPLE ACID PHOSPHATASE 11)	-1.559	3.86E-06
At2g18193	P-loop containing nucleoside triphosphate hydrolases superfamily protein	1.004	0.00421

At2g18230	AtPPa2 (pyrophosphorylase 2); Encodes a protein that might have inorganic pyrophosphatase activity.	1.289	1.51E-05
At2g18300	basic helix-loop-helix (bHLH) DNA-binding superfamily protein	-2.626	5.94E-10
At2g18480	Major facilitator superfamily protein	-1.408	2.49E-06
At2g18510	emb2444 (embryo defective 2444)	1.667	1.37E-07
At2g18560	UDP-Glycosyltransferase superfamily protein	-1.655	1.04E-06
At2g18570	UDP-Glycosyltransferase superfamily protein	-1.432	3.27E-06
At2g18650	MEE16 (maternal effect embryo arrest 16)	-1.04	6.79E-07
At2g18680	unknown protein	2.14	5.50E-07
At2g18690	unknown protein	1.268	0.0481
At2g18780	F-box and associated interaction domains-containing protein	2.765	3.40E-11
At2g18870	VEL3 (vernalization5/VIN3-like)	1.107	7.95E-07
At2g18969	BEST Arabidopsis thaliana protein match is: sequence-specific DNA binding transcription factors	-1.259	0.00027
At2g18980	Peroxidase superfamily protein	-1.344	0.00018
At2g19110	ATHMA4 (ARABIDOPSIS HEAVY METAL ATPASE 4); Encodes a protein with similarity to Zn ATPase. C	-1.353	1.89E-08
At2g19270	CONTAINS InterPro DOMAIN/s: Mitotic checkpoint protein PRCC, C-terminal (InterPro:IPR018800); Has 9:	1.16	1.96E-07
At2g19310	HSP20-like chaperones superfamily protein	3.697	3.90E-12
At2g19560	EER5 (ENHANCED ETHYLENE RESPONSE 5); encodes a protein with a PAM domain involved in ethylene	1.13	1.22E-06
At2g19580	TET2 (tetraspanin2); Member of TETRASPANIN family	1.56	1.66E-09
At2g19582	other RNA; Potential natural antisense gene, locus overlaps with AT2G19580	1.977	4.85E-09
At2g19610	RING/U-box superfamily protein	1.567	1.56E-05
At2g19630	F-box and associated interaction domains-containing protein	1.976	4.83E-11
At2g19660	Cysteine/Histidine-rich C1 domain family protein	-1.861	4.45E-08
At2g19710	Regulator of Vps4 activity in the MVB pathway protein	1.336	4.36E-07
At2g19800	MIOX2 (myo-inositol oxygenase 2); Encodes a myo-inositol oxygenase family gene.	-1.709	0.00155
At2g19890	BEST Arabidopsis thaliana protein match is: Putative endonuclease or glycosyl hydrolase (TAIR:AT3G62210.	1.581	8.57E-10
At2g19990	PR-1-LIKE (pathogenesis-related protein-1-like); Encodes a PR-1-like protein homolog that is differentially ex	-1.499	6.21E-07
At2g20150	unknown protein	1.9	4.22E-06
At2g20208	LCR60 (low-molecular-weight cysteine-rich 60); Encodes a member of a family of small,secreted, cysteine ricl	1.73	6.82E-07
At2g20320	DENN (AEX-3) domain-containing protein	1.283	3.28E-08
At2g20440	Ypt/Rab-GAP domain of gyp1p superfamily protein	-1.118	4.53E-06
At2g20560	DNAJ heat shock family protein	6.642	1.02E-13
At2g20562	unknown protein	2.82	1.55E-08
At2g20570	ATGLK1 (ARABIDOPSIS GOLDEN2-LIKE 1); Encodes GLK1, Golden2-like 1, one of a pair of partially red	-1.086	1.82E-05
At2g20610	ALF1 (ABERRANT LATERAL ROOT FORMATION 1); Confers auxin overproduction. Mutants have an ove	-1.801	1.65E-07
At2g20670	Protein of unknown function (DUF506)	-1.743	4.24E-05
At2g20700	LLG2 (LORELEI-LIKE-GPI ANCHORED PROTEIN 2)	2.451	8.30E-09
At2g20720	Pentatricopeptide repeat (PPR) superfamily protein	3.836	1.20E-12
At2g20825	ULT2 (ULTRAPETALA 2)	1.679	2.88E-09
At2g20900	DGK5 (diacylglycerol kinase 5)	1.01	1.54E-07
At2g20940	Protein of unknown function (DUF1279)	1.931	7.22E-11
At2g20950	Arabidopsis phospholipase-like protein (PEARLI 4) family	1.281	3.68E-08
At2g21050	LAX2 (like AUXIN RESISTANT 2); Encodes LAX2 (LIKE AUXIN RESISTANT), a member of the AUX1 L	-1.078	2.09E-06
At2g21080	unknown protein	-2.413	3.26E-10
At2g21185	unknown protein	-1.228	7.18E-06
At2g21188	other RNA; Unknown gene	-1.13	7.61E-05
At2g21200	SAUR-like auxin-responsive protein family	-1.167	9.10E-07
At2g21210	SAUR-like auxin-responsive protein family ; Putative auxin-regulated protein whose expression is downregula	-2.319	6.11E-10
At2g21330	FBA1 (fructose-bisphosphate aldolase 1)	1.51	5.68E-05
At2g21380	Kinesin motor family protein	-1.002	1.42E-07
At2g21430	Papain family cysteine protease	-1.483	5.74E-08
At2g21620	RD2 (Adenine nucleotide alpha hydrolases-like superfamily protein); Encodes gene that is induced in response	1.334	2.36E-07
At2g21640	unknown protein; Encodes a protein of unknown function that is a marker for oxidative stress response.	1.432	6.35E-07
At2g21650	ATRL2 (ARABIDOPSIS RAD-LIKE 2); RSM1 is a member of a small sub-family of single MYB transcriptio	-1.598	7.07E-05
At2g21660	ATGRP7 (GLYCINE RICH PROTEIN 7); Encodes a small glycine-rich RNA binding protein that is part of a	-1.59	0.0039
At2g21820	unknown protein	1.709	0.0292
At2g21880	ATRAB7A (RAB GTPase homolog 7A)	2.303	4.14E-08
At2g21940	SK1 (shikimate kinase 1)	3.549	2.86E-11
At2g22090	UBA1A (RNA-binding (RRM/RBD/RNP motifs) family protein); encodes a nuclear protein that binds to RNA	1.572	1.53E-08
At2g22122	unknown protein	-1.53	1.05E-06
At2g22190	Haloacid dehalogenase-like hydrolase (HAD) superfamily protein	-1.533	1.04E-05
At2g22200	Integrase-type DNA-binding superfamily protein; encodes a member of the DREB subfamily A-6 of ERF/AP2	-2.03	3.96E-07
At2g22240	ATIPS2 (INOSITOL 3-PHOSPHATE SYNTHASE 2); ** Referred to as MIPS1 in Mitsuhashi et al 2008. Myo	4.514	4.82E-12
At2g22330	CYP79B3 (cytochrome P450, family 79, subfamily B, polypeptide 3); Encodes a cytochrome P450. Involved i	-2.646	9.40E-11
At2g22335	pseudogene of cytochrome P450 family protein; pseudogene of cytochrome P450 family protein	-2.244	2.58E-09
At2g22360	DNAJ heat shock family protein	1.331	5.69E-09
At2g22450	riboflavin biosynthesis protein, putative	-1.593	8.65E-07
At2g22496	MIR779A (microRNA779A); Encodes a microRNA of unknown function. MicroRNAs are regulatory RNAs w	2.022	5.99E-07

At2g22560	Kinase interacting (KIP1-like) family protein	1.943	1.49E-08
At2g22795	unknown protein	-1.029	0.0002
At2g22800	HAT9 (Homeobox-leucine zipper protein family); Encodes homeobox protein HAT9.	-1.371	7.35E-08
At2g22810	ACC4 (1-AMINOCYCLOPROPANE-1-CARBOXYLIC ACID SYNTHASE POLYPEPTIDE); key regulatory	-1.846	8.72E-07
At2g22860	ATPSK2 (phytosulfokine 2 precursor); Phytosulfokine 2 precursor, coding for a unique plant peptide growth fa	1.574	7.31E-06
At2g22880	VQ motif-containing protein	1.07	0.00279
At2g22930	UDP-Glycosyltransferase superfamily protein	-3.489	4.45E-08
At2g23000	scpl10 (serine carboxypeptidase-like 10)	1.252	2.75E-05
At2g23110	Late embryogenesis abundant protein, group 6	3.08	2.80E-08
At2g23130	AGP17 (arabinogalactan protein 17); AGP17 is a lysine-rich arabinogalactan-protein (AGP) and part of a mult	-1.017	0.0002
At2g23140	RING/U-box superfamily protein with ARM repeat domain	1.124	2.54E-07
At2g23170	GH3.3 (Auxin-responsive GH3 family protein); encodes an IAA-amido synthase that conjugates Asp and other	-1.536	7.09E-06
At2g23180	CYP96A1 (cytochrome P450, family 96, subfamily A, polypeptide 1); member of CYP96A	-1.41	1.00E-08
At2g23320	WRKY15 (WRKY DNA-binding protein 15); Encodes WRKY DNA-binding protein 15 (WRKY15).	2.359	9.62E-10
At2g23420	NAPRT2 (nicotinate phosphoribosyltransferase 2)	1.862	5.84E-09
At2g23430	KRP1 (KIP-RELATED PROTEIN 1); Encodes a cyclin-dependent kinase inhibitor protein that functions as a n	-2.188	2.11E-08
At2g23560	ATMES7 (ARABIDOPSIS THALIANA METHYL ESTERASE 7); Encodes a protein shown to have carboxyl	-1.05	1.81E-05
At2g23755	unknown protein	-1.224	4.62E-08
At2g23780	RING/U-box superfamily protein	1.074	3.88E-08
At2g23810	TET8 (tetraspanin8); Member of TETRASPANIN family	2.136	2.53E-06
At2g23910	NAD(P)-binding Rossmann-fold superfamily protein	-1.343	0.00054
At2g24040	Low temperature and salt responsive protein family	1.522	2.93E-07
At2g24100	unknown protein	3.886	1.13E-12
At2g24110	40S ribosomal protein S11, expressed pseudogene, similar to GB:J05216	1.789	6.55E-08
At2g24320	alpha/beta-Hydrolases superfamily protein	2.362	5.77E-11
At2g24540	AFR (ATTENUATED FAR-RED RESPONSE)	1.565	3.45E-05
At2g24600	Ankyrin repeat family protein	2.032	1.33E-06
At2g24680	transcriptional factor B3 family protein	2.333	3.55E-10
At2g24760	non-LTR retrotransposon family (LINE), has a 4.9e-16 P-value blast match to GB:NP_038605 L1 repeat, Tf su	1.488	3.99E-09
At2g24762	AtGDU4 (glutamine dumper 4); Encodes a member of the GDU (glutamine dumper) family proteins involved	-1.688	4.55E-07
At2g25140	CLPB-M (CASEIN LYTIC PROTEINASE B-M); Encodes ClpB4, which belongs to the Casein lytic proteinase	4.759	1.28E-11
At2g25150	HXXXD-type acyl-transferase family protein	-1.04	0.00049
At2g25200	Plant protein of unknown function (DUF868)	-1.739	8.23E-07
At2g25450	2-oxoglutarate (2OG) and Fe(II)-dependent oxygenase superfamily protein; encodes a protein whose sequence	-1.146	9.43E-06
At2g25460	CONTAINS InterPro DOMAIN/s: C2 calcium-dependent membrane targeting (InterPro:IPR000008); BEST A	1.241	0.00015
At2g25470	AtRLP21 (receptor like protein 21)	1.086	0.00504
At2g25482	LOCATED IN: endomembrane system (Protein of unknown function (DUF784)); Encodes a ECA1 gametogen	1.445	1.02E-07
At2g25500	Inosine triphosphate pyrophosphatase family protein	2.269	7.46E-09
At2g25735	unknown protein	1.225	2.02E-05
At2g25780	Protein of unknown function (DUF1677)	-2.071	5.46E-07
At2g25900	ATTZF1 (A. THALIANA TANDEM ZINC FINGER PROTEIN 1); putative Cys3His zinc finger protein (ATC	-1.129	2.13E-06
At2g26000	BRIZ2 (BRAP2 RING ZnF UBP domain-containing protein 2)	2.304	4.88E-09
At2g26140	ftsh4 (FTSH protease 4); encodes an FtsH protease that is localized to the mitochondrion	1.811	5.38E-09
At2g26150	ATHSFA2 (heat shock transcription factor A2); member of Heat Stress Transcription Factor (Hsf) family. Invo	9.49	6.56E-13
At2g26190	calmodulin-binding family protein	1.332	8.48E-06
At2g26250	FDH (FIDDLEHEAD); epidermis-specific, encodes KCS10, a putative 3-ketoacyl-CoA synthase. probably inv	-1.007	1.25E-06
At2g26360	Mitochondrial substrate carrier family protein	-1.428	7.17E-07
At2g26400	ARD (ACIREDUCTONE DIOXYGENASE); Encodes a protein predicted to belong to the acireductone dioxyg	-1.61	0.02646
At2g26440	Plant invertase/pectin methylesterase inhibitor superfamily	-1.21	0.00023
At2g26530	AR781 (Protein of unknown function (DUF1645)); unknown function	2.286	4.25E-08
At2g26560	PLA IIA (PHOSPHOLIPASE A 2A); Encodes a lipid acyl hydrolase with wide substrate specificity that accum	1.092	0.0381
At2g26570	Plant protein of unknown function (DUF827); Encodes a coiled-coil protein WEB1 (weak chloroplast moveme	1.491	5.62E-08
At2g26590	RPN13 (regulatory particle non-ATPase 13)	1.109	2.84E-07
At2g26640	KCS11 (3-ketoacyl-CoA synthase 11); Encodes KCS11, a member of the 3-ketoacyl-CoA synthase family invo	-1.143	1.35E-07
At2g26695	Ran BP2/NZF zinc finger-like superfamily protein	-1.776	1.83E-06
At2g26730	Leucine-rich repeat protein kinase family protein	-1.23	7.64E-08
At2g26800	Aldolase superfamily protein	1.277	2.86E-07
At2g26975	Ctr copper transporter family	1.089	4.13E-07
At2g27120	TIL2 (TILTED 2); Encodes a protein with similarity to DNA polymerase epsilon catalytic subunit. Based on ye	1.936	1.29E-07
At2g27190	ATPAP1 (ARABIDOPSIS THALIANA PURPLE ACID PHOSPHATASE 1); secreted purple acid phosphatase	-1.298	0.0002
At2g27200	P-loop containing nucleoside triphosphate hydrolases superfamily protein	1.237	1.48E-08
At2g27260	Late embryogenesis abundant (LEA) hydroxyproline-rich glycoprotein family	1.054	4.25E-05
At2g27270	BEST Arabidopsis thaliana protein match is: Late embryogenesis abundant (LEA) hydroxyproline-rich glycopr	3.738	9.75E-12
At2g27280	Coiled-coil domain-containing protein 55 (DUF2040)	3.336	1.13E-08
At2g27395	pseudogene of cysteine protease-related; pseudogene of cysteine protease-related	1.568	0.013
At2g27420	Cysteine proteinases superfamily protein	1.84	0.00169
At2g27550	ATC (centroradialis); encodes a protein similar to TFL1. overexpression leads to similar phenotype as TFL1 ov	1.204	0.0117

At2g27570	P-loop containing nucleoside triphosphate hydrolases superfamily protein	1.513	9.10E-07
At2g27580	A20/AN1-like zinc finger family protein	3.313	3.92E-11
At2g27920	SCPL51 (serine carboxypeptidase-like 51)	-1.079	5.29E-06
At2g28040	Eukaryotic aspartyl protease family protein	2.848	1.20E-09
At2g28056	MIR172 (MICRORNA 172); Encodes a microRNA that targets several genes containing AP2 domains includi	3.783	3.26E-12
At2g28180	CHX8 (CATION/H+ EXCHANGER 8); member of Putative Na+/H+ antiporter family	2.067	3.87E-06
At2g28210	ACA2 (alpha carbonic anhydrase 2)	3.856	2.89E-07
At2g28410	unknown protein	-1.297	2.91E-06
At2g28630	KCS12 (3-ketoacyl-CoA synthase 12); Encodes KCS12, a member of the 3-ketoacyl-CoA synthase family invo	-2.735	2.79E-10
At2g28650	ATEXO70H8 (exocyst subunit exo70 family protein H8); A member of EXO70 gene family, putative exocyst	1.431	0.00012
At2g28660	Chloroplast-targeted copper chaperone protein	-1.389	1.65E-08
At2g28710	C2H2-type zinc finger family protein	1.074	2.66E-05
At2g28720	Histone superfamily protein	-1.202	6.18E-07
At2g28740	HIS4 (histone H4); histone 4	-1.18	3.71E-06
At2g28810	Dof-type zinc finger DNA-binding family protein	-1.299	9.61E-08
At2g28870	unknown protein	-1.796	1.67E-08
At2g28900	ATOEP16-1 (outer plastid envelope protein 16-1); Encodes AtOEP16, a 16-KDa plastid outer membrane prote	-1.53	9.03E-07
At2g28930	APK1B (protein kinase 1B)	-1.103	1.59E-05
At2g28950	ATEXP6 (ARABIDOPSIS THALIANA TEXPANSIN 6); Encodes an expansin. Naming convention from the l	-1.157	3.64E-06
At2g29045	LCR62 (low-molecular-weight cysteine-rich 62); Encodes a member of a family of small,secreted, cysteine ricl	-1.36	0.0156
At2g29090	CYP707A2 (cytochrome P450, family 707, subfamily A, polypeptide 2); Encodes a protein with ABA 8'-hydro	-1.655	6.13E-07
At2g29170	NAD(P)-binding Rossmann-fold superfamily protein	-1.342	1.02E-05
At2g29280	pseudogene, tropinone reductase, blastp match of 46% identity and 2.4e-09 P-value to SP|P50165|TRNH_DA	-2.111	1.34E-06
At2g29300	NAD(P)-binding Rossmann-fold superfamily protein	-1.193	0.00013
At2g29310	NAD(P)-binding Rossmann-fold superfamily protein	-1.11	1.34E-06
At2g29340	NAD-dependent epimerase/dehydratase family protein	1.43	5.44E-07
At2g29420	ATGSTU7 (glutathione S-transferase tau 7); Encodes glutathione transferase belonging to the tau class of GST	1.609	1.62E-08
At2g29450	ATGSTU1 (ARABIDOPSIS THALIANA GLUTATHIONE S-TRANSFERASE TAU 1); Encodes a member	3.033	9.66E-10
At2g29460	ATGSTU4 (glutathione S-transferase tau 4); Encodes glutathione transferase belonging to the tau class of GST	3.04	0.00024
At2g29470	ATGSTU3 (glutathione S-transferase tau 3); Encodes glutathione transferase belonging to the tau class of GST	1.729	0.01835
At2g29480	ATGSTU2 (glutathione S-transferase tau 2); Encodes glutathione transferase belonging to the tau class of GST	3.46	3.06E-09
At2g29490	ATGSTU1 (glutathione S-transferase TAU 1); Encodes glutathione transferase belonging to the tau class of GS	1.978	5.71E-07
At2g29500	HSP20-like chaperones superfamily protein	7.57	1.32E-11
At2g29600	Galactose oxidase/kelch repeat superfamily protein	2.747	1.11E-08
At2g29720	CTF2B (FAD/NAD(P)-binding oxidoreductase family protein); Encodes CTF2B.	3.652	1.49E-12
At2g29740	UGT71C2 (UDP-glucosyl transferase 71C2)	4.32	6.48E-10
At2g29990	NDA2 (alternative NAD(P)H dehydrogenase 2)	2.61	8.15E-10
At2g30000	PHF5-like protein	3.838	9.19E-13
At2g30020	Protein phosphatase 2C family protein; Encodes AP2C1. Belongs to the clade B of the PP2C-superfamily. Ac	1.599	1.31E-09
At2g30140	UDP-Glycosyltransferase superfamily protein	1.632	4.50E-07
At2g30210	LAC3 (laccase 3); putative laccase, a member of laccase family of genes (17 members in Arabidopsis).	-1.356	0.03813
At2g30240	CHX13 (CATION/H+ EXCHANGER 13); Encodes a plasma membrane localized potassium transporter.	1.367	3.37E-06
At2g30250	WRKY25 (WRKY DNA-binding protein 25); member of WRKY Transcription Factor; Group I. Located in nu	1.944	6.27E-08
At2g30424	TCL2 (TRICHOMELESS 2); In a tandem repeat with AT2g30432 (TCL1) and AT2g30420 (ETC2)	-1.669	1.59E-08
At2g30480	unknown protein	2.292	1.84E-09
At2g30550	alpha/beta-Hydrolases superfamily protein; Encodes a lipase that hydrolyzes phosphatidylcholine, glycolipids a	1.248	3.81E-07
At2g30600	BTB/POZ domain-containing protein	-1.54	7.43E-06
At2g30670	NAD(P)-binding Rossmann-fold superfamily protein	-1.309	5.38E-05
At2g30840	2-oxoglutarate (2OG) and Fe(II)-dependent oxygenase superfamily protein; encodes a protein whose sequence	-1.361	9.22E-05
At2g30860	ATGSTF9 (glutathione S-transferase PHI 9); Encodes glutathione transferase belonging to the phi class of GS	-1.284	7.69E-06
At2g30930	unknown protein	-1.287	6.43E-06
At2g31090	unknown protein	1.187	6.05E-06
At2g31200	ADF6 (actin depolymerizing factor 6); Encodes actin depolymerizing factor 6 (ADF6).	1.049	1.32E-06
At2g31230	ATERF15 (ethylene-responsive element binding factor 15); encodes a member of the ERF (ethylene response f	1.179	1.18E-05
At2g31260	APG9 (autophagy 9); Involved in autophagy, the process of vacuolar bulk degradation of cytoplasmic compone	1.097	9.43E-06
At2g31290	Ubiquitin carboxyl-terminal hydrolase family protein	-1.015	6.79E-07
At2g31380	STH (salt tolerance homologue); a B-box zinc finger protein that interacts with COP1. contains a novel 11 ami	1.194	6.41E-07
At2g31425	Plant invertase/pectin methylesterase inhibitor superfamily protein	1.176	7.19E-06
At2g31750	UGT74D1 (UDP-glucosyl transferase 74D1)	1.291	2.54E-07
At2g31790	UDP-Glycosyltransferase superfamily protein	-1.601	1.07E-09
At2g31860	pseudogene, poly (ADP-ribose) glycohydrolase, putative, contains Pfam domain, PF05028: poly (ADP-ribose)	1.603	1.58E-07
At2g31865	PARG2 (poly(ADP-ribose) glycohydrolase 2)	2.26	7.15E-06
At2g31880	EVR (EVERSHED); Encodes a putative leucine rich repeat transmembrane protein that is expressed in respon	1.452	2.93E-05
At2g31900	ATMYO5 (MYOSIN 5); Encodes an novel myosin isoform.	-1.179	2.14E-07
At2g31920	Plant protein of unknown function (DUF936)	1.148	2.87E-06
At2g31945	unknown protein	3.248	2.22E-08
At2g31955	CNX2 (cofactor of nitrate reductase and xanthine dehydrogenase 2); COFACTOR OF NITRATE REDUCTAS	1.271	0.0006

At2g31990	Exostosin family protein	1.292	9.66E-07
At2g32020	Acyl-CoA N-acyltransferases (NAT) superfamily protein	2.314	5.77E-09
At2g32030	Acyl-CoA N-acyltransferases (NAT) superfamily protein	3.679	2.99E-10
At2g32050	Family of unknown function (DUF572)	2.301	4.05E-07
At2g32100	ATOFP16 (RABIDOPSIS THALIANA OVATE FAMILY PROTEIN 16)	-1.933	4.81E-06
At2g32120	HSP70T-2 (heat-shock protein 70T-2)	7.364	5.43E-13
At2g32130	Plant protein of unknown function (DUF641)	4.156	9.19E-09
At2g32140	transmembrane receptors	3.895	1.92E-09
At2g32190	unknown protein	2.197	2.35E-05
At2g32200	unknown protein	2.206	1.26E-05
At2g32210	unknown protein	2.64	7.79E-06
At2g32290	BAM6 (beta-amylase 6)	1.593	6.35E-06
At2g32550	Cell differentiation, Rcd1-like protein	1.028	0.0002
At2g32660	AtRLP22 (receptor like protein 22)	-2.067	0.00012
At2g32970	unknown protein	-1.005	9.55E-08
At2g33100	ATCSLD1 (cellulose synthase-like D1); encodes a gene similar to cellulose synthase	4.89	3.40E-11
At2g33110	ATVAMP723 (vesicle-associated membrane protein 723); member of VAMP72 Gene Family	1.742	3.85E-08
At2g33170	Leucine-rich repeat receptor-like protein kinase family protein	-1.251	3.90E-06
At2g33240	ATXID (MYOSIN XI D); member of Myosin-like proteins	1.17	1.56E-06
At2g33380	CLO-3 (CALEOSIN 3); Encodes a calcium binding protein whose mRNA is induced upon treatment with NaC	3.159	1.59E-08
At2g33460	RIC1 (ROP-interactive CRIB motif-containing protein 1); encodes a member of a novel protein family that cor	2.23	3.79E-10
At2g33490	hydroxyproline-rich glycoprotein family protein	1.013	4.40E-06
At2g33580	Protein kinase superfamily protein	1.668	1.40E-05
At2g33585	unknown protein	1.229	1.54E-07
At2g33590	NAD(P)-binding Rossmann-fold superfamily protein	4.047	7.08E-11
At2g33670	ATMLO5 (MILDEW RESISTANCE LOCUS O 5); A member of a large family of seven-transmembrane doma	1.68	6.30E-06
At2g33700	Protein phosphatase 2C family protein	2.521	6.81E-12
At2g33750	ATPUP2 (purine permease 2); Member of a family of proteins related to PUP1, a purine transporter. May be ir	-1.496	2.26E-07
At2g33830	Dormancy/auxin associated family protein	-3.229	5.58E-06
At2g33850	unknown protein	-1.084	0.00096
At2g34010	unknown protein	-2.125	1.66E-07
At2g34020	Calcium-binding EF-hand family protein	2.812	5.25E-07
At2g34030	Calcium-binding EF-hand family protein	4.328	9.38E-10
At2g34040	Apoptosis inhibitory protein 5 (API5)	1.362	1.87E-06
At2g34210	Transcription elongation factor Spt5	-1.52	0.0181
At2g34260	transducin family protein / WD-40 repeat family protein	1.095	3.72E-06
At2g34355	Major facilitator superfamily protein	1.695	1.37E-08
At2g34360	MATE efflux family protein	2.166	6.88E-10
At2g34430	LHB1B1 (light-harvesting chlorophyll-protein complex II subunit B1); Photosystem II type I chlorophyll	-1.526	0.0058
At2g34460	NAD(P)-binding Rossmann-fold superfamily protein	1.36	1.11E-07
At2g34490	CYP710A2 (cytochrome P450, family 710, subfamily A, polypeptide 2); Encodes a protein with C22-sterol de	-1.181	4.90E-06
At2g34580	CONTAINS InterPro DOMAIN/s: Herpesvirus UL139, cytomegalovirus (InterPro:IPR021042); Has 30201 Bla	3.248	6.78E-10
At2g34590	Transketolase family protein	1.21	2.15E-07
At2g34660	ATMRP2 (multidrug resistance-associated protein 2); encodes a multidrug resistance-associated protein that is	2.287	2.06E-09
At2g34770	ATFAH1 (ARABIDOPSIS FATTY ACID HYDROXYLASE 1); encodes a fatty acid hydroxylase, required for	1.156	1.93E-05
At2g34930	disease resistance family protein / LRR family protein	-1.034	0.00099
At2g35035	URED (urease accessory protein D); Encodes a urease accessory protein which is essential for the activation of	2.562	6.89E-10
At2g35075	unknown protein	-1.065	1.75E-05
At2g35290	unknown protein	-1.126	1.96E-05
At2g35570	pseudogene, serpin (serine protease inhibitor) family, similar to phloem serpin-1 (Cucurbita maxima) GI:9937	1.639	0.0001
At2g35658	unknown protein	3.196	8.75E-09
At2g35660	CTF2A (FAD/NAD(P)-binding oxidoreductase family protein); Encodes a member of a novel gene family with	1.137	0.0004
At2g35740	ATINT3 (NOSITOL TRANSPORTER 3)	2.452	5.88E-08
At2g35890	CPK25 (calcium-dependent protein kinase 25); member of Calcium Dependent Protein Kinase	1.179	0.01749
At2g35930	PUB23 (plant U-box 23); Encodes a cytoplasmically localized U-box domain containing E3 ubiquitin ligase th	2.358	2.44E-10
At2g36050	ATOFP15 (ARABIDOPSIS THALIANA OVATE FAMILY PROTEIN 15)	-1.26	5.30E-06
At2g36053	unknown protein	-1.079	2.78E-06
At2g36110	Polynucleotidyl transferase, ribonuclease H-like superfamily protein	-1.003	1.26E-06
At2g36120	DOT1 (DEFECTIVELY ORGANIZED TRIBUTARIES 1); Encodes a glycine rich protein that is involved in l	-1.09	0.0003
At2g36220	unknown protein	2.068	2.51E-07
At2g36390	BE3 (BRANCHING ENZYME 3); Encodes a starch branching enzyme (EC.2.4.1.18) similar to SBE2 from ma	-1.14	2.00E-07
At2g36410	Family of unknown function (DUF662)	-1.275	1.85E-06
At2g36430	Plant protein of unknown function (DUF247)	-1.147	2.40E-05
At2g36460	Aldolase superfamily protein	1.207	3.06E-06
At2g36470	Plant protein of unknown function (DUF868)	-1.031	8.30E-06
At2g36630	Sulfite exporter TauE/SafE family protein	-1.609	4.42E-07
At2g36695	unknown protein	1.266	8.24E-05

At2g36750	UGT73C1 (UDP-glucosyl transferase 73C1)	2.494	7.95E-07
At2g36770	UDP-Glycosyltransferase superfamily protein	1.218	1.22E-06
At2g36780	UDP-Glycosyltransferase superfamily protein	1.306	6.14E-05
At2g36790	UGT73C6 (UDP-glucosyl transferase 73C6); The At2g36790 gene encodes a UDP-glucose:flavonol-3-O-glycc	2.145	1.19E-06
At2g36800	DOGT1 (don-glucosyltransferase 1); Encodes a DON-Glucosyltransferase. The UGT73C5 glucosylates both b	1.421	6.04E-06
At2g36881	unknown protein	-1.161	0.00051
At2g36895	unknown protein	1.433	5.23E-06
At2g36950	Heavy metal transport/detoxification superfamily protein	1.973	2.19E-08
At2g37030	SAUR-like auxin-responsive protein family	-1.151	0.01451
At2g37110	PLAC8 family protein	1.222	2.91E-07
At2g37130	Peroxidase superfamily protein	-2.067	1.11E-06
At2g37180	PIP2.3 (PLASMA MEMBRANE INTRINSIC PROTEIN 2.3); a member of the plasma membrane intrinsic prc	2.255	3.47E-08
At2g37240	Thioredoxin superfamily protein	1.492	3.64E-07
At2g37280	ATPDR5 (PLEIOTROPIC DRUG RESISTANCE 5)	-1.249	8.13E-06
At2g37310	Pentatricopeptide repeat (PPR) superfamily protein	-1.05	1.82E-05
At2g37330	ALS3 (ALUMINUM SENSITIVE 3); Encodes an ABC transporter-like protein, without an ATPase domain, re	-1.058	8.47E-06
At2g37380	unknown protein	-1.154	1.96E-07
At2g37430	C2H2 and C2HC zinc fingers superfamily protein	4.289	2.23E-10
At2g37460	nodulin MtN21 /EamA-like transporter family protein	-1.338	1.19E-06
At2g37530	unknown protein	-1.192	3.86E-07
At2g37540	NAD(P)-binding Rossmann-fold superfamily protein	1.488	1.24E-07
At2g37640	ATEXP3 (EXPANSIN 3); member of Alpha-Expansin Gene Family. Naming convention from the Expansin W	-1.679	5.09E-07
At2g37740	ATZFP10 (ZINC-FINGER PROTEIN 10)	-1.212	0.00478
At2g37760	NAD(P)-linked oxidoreductase superfamily protein	1.513	1.83E-06
At2g37770	NAD(P)-linked oxidoreductase superfamily protein	1.338	0.00011
At2g37940	AtIPCS2 (Arabidopsis Inositol phosphorylceramide synthase 2); I	1.274	7.93E-06
At2g37950	RING/FYVE/PHD zinc finger superfamily protein	-1.554	2.28E-05
At2g37970	SOUL-1 (SOUL heme-binding family protein)	3.787	8.53E-11
At2g37980	O-fucosyltransferase family protein	1.052	9.10E-06
At2g38120	AUX1 (AUXIN RESISTANT 1); Encodes an auxin influx transporter. AUX1 resides at the apical plasma mer	-1.538	1.05E-08
At2g38170	CAX1 (cation exchanger 1); Encodes a high affinity vacuolar calcium antiporter. The residue His 338 is critic	-1.131	9.22E-06
At2g38195	RING/U-box superfamily protein	1.084	1.99E-06
At2g38230	ATPDX1.1 (ARABIDOPSIS THALIANA PYRIDOXINE BIOSYNTHESIS 1.1); Encodes a protein predicted	1.565	5.19E-07
At2g38250	Homeodomain-like superfamily protein	2.853	7.83E-09
At2g38255	Protein of Unknown Function (DUF239)	3.182	3.65E-10
At2g38290	AMT2 (ammonium transporter 2); encodes a high-affinity ammonium transporter, which is expressed in shoot	1.029	2.38E-06
At2g38300	myb-like HTH transcriptional regulator family protein	-2.469	2.48E-08
At2g38340	Integrase-type DNA-binding superfamily protein; encodes a member of the DREB subfamily A-2 of ERF/AP2	6.293	1.29E-10
At2g38350	unknown protein	1.127	0.0004
At2g38360	PRA1.B4 (prenylated RAB acceptor 1.B4)	1.108	1.85E-06
At2g38470	ATWRKY33 (WRKY DNA-BINDING PROTEIN 33); Member of the plant WRKY transcription factor family	3.266	6.41E-09
At2g38500	2-oxoglutarate (2OG) and Fe(II)-dependent oxygenase superfamily protein	1.368	1.58E-08
At2g38520	copia-like retrotransposon family, has a 8.4e-60 P-value blast match to dbj|BAA78427.1| polyprotein (AtRE2-2	2.034	2.20E-06
At2g38560	TFIIS (transcript elongation factor IIS); Encodes RNA polymerase II transcript elongation factor TFIIS. Comp	1.066	5.14E-07
At2g38830	Ubiquitin-conjugating enzyme/RWD-like protein	1.916	9.54E-09
At2g38940	ATPT2 (ARABIDOPSIS THALIANA PHOSPHATE TRANSPORTER 2); Encodes Pht1;4, a member of the P	-2.086	0.0005
At2g39010	PIP2.6 (PLASMA MEMBRANE INTRINSIC PROTEIN 2.6)	-1.061	0.0002
At2g39050	hydroxyproline-rich glycoprotein family protein	1.363	8.38E-06
At2g39420	alpha/beta-Hydrolases superfamily protein	1.461	3.48E-07
At2g39510	nodulin MtN21 /EamA-like transporter family protein	-1.423	0.0003
At2g39650	Protein of unknown function (DUF506)	3.059	7.58E-09
At2g39730	RCA (rubisco activase); Rubisco activase, a nuclear-encoded chloroplast protein that consists of two isoforms	1.095	0.0002
At2g39782	unknown protein	-1.124	4.62E-05
At2g39850	Subtilisin-like serine endopeptidase family protein	-1.087	0.0005
At2g39900	GATA type zinc finger transcription factor family protein	-1.008	1.55E-05
At2g39920	HAD superfamily, subfamily IIIB acid phosphatase	-2.372	5.39E-08
At2g40000	ATHSPRO2 (ARABIDOPSIS ORTHOLOG OF SUGAR BEET HS1 PRO-1 2)	1.54	8.10E-05
At2g40080	ELF4 (EARLY FLOWERING 4); Encodes a novel nuclear 111 amino-acid phytochrome-regulated component	-2.129	6.00E-08
At2g40090	ATATH9 (ABC2 homolog 9); member of ATH subfamily	1.835	3.19E-08
At2g40095	Alpha/beta hydrolase related protein	1.932	6.16E-08
At2g40100	LHCB4.3 (light harvesting complex photosystem II); Lhcb4:3 protein (Lhcb4.3, light harvesting complex of pl	1.416	2.33E-06
At2g40113	Pollen Ole e 1 allergen and extensin family protein	1.195	9.63E-05
At2g40130	Double Clp-N motif-containing P-loop nucleoside triphosphate hydrolases superfamily protein	-1.663	7.88E-07
At2g40180	ATHPP2C5 (phosphatase 2C5); Encodes PP2C5, a member of the PP2C family phosphatases. PP2C5 acts as a	2.488	1.96E-07
At2g40200	basic helix-loop-helix (bHLH) DNA-binding superfamily protein	2.491	9.84E-08
At2g40260	Homeodomain-like superfamily protein	-1.431	1.66E-06
At2g40330	PYL6 (PYR1-like 6); Encodes a member of the PYR (pyrabactin resistance)/PYL(PYR1-like)/RCAR (regulat	-1.081	5.14E-05

At2g40340	DREB2C (Integrase-type DNA-binding superfamily protein); Encodes a member of the DREB subfamily A-2 c	2.69	6.38E-09
At2g40350	Integrase-type DNA-binding superfamily protein; encodes a member of the DREB subfamily A-2 of ERF/AP2	1.537	0.00023
At2g40530	unknown protein	-1.812	4.38E-06
At2g40610	ATEXPA8 (expansin A8); member of Alpha-Expansin Gene Family. Naming convention from the Expansin W	-2.538	6.47E-06
At2g40670	ARR16 (response regulator 16); response regulator 16	-2.101	2.01E-08
At2g40730	Protein kinase family protein with ARM repeat domain	1.017	0.0041
At2g40740	ATWRKY55 (WRKY DNA-BINDING PROTEIN 55); member of WRKY Transcription Factor; Group III	1.021	0.00382
At2g40750	ATWRKY54 (WRKY DNA-BINDING PROTEIN 54); member of WRKY Transcription Factor; Group III	-1.623	0.00016
At2g40830	RHC1A (RING-H2 finger C1A); Encodes a putative RING-H2 finger protein RHC1a.	1.009	1.85E-05
At2g40850	ATPI4K GAMMA 1 (phosphoinositide 4-kinase gamma 1)	2.329	3.08E-10
At2g40860	protein kinase family protein / protein phosphatase 2C (PP2C) family protein	1.279	1.28E-06
At2g40900	nodulin MtN21 /EamA-like transporter family protein	-1.708	3.36E-06
At2g40940	ERS (ETHYLENE RESPONSE SENSOR); Ethylene receptor, subfamily 1. Has histidine kinase activity.	-1.07	1.88E-07
At2g41010	ATCAMBP25 (calmodulin (CAM)-binding protein of 25 kDa); Encodes a novel calmodulin binding protein w	2.158	2.95E-08
At2g41070	EEL (ENHANCED EM LEVEL); Transcription factor homologous to ABI5. Regulates AtEm1 expression by b	1.389	2.65E-07
At2g41100	ATCAL4 (ARABIDOPSIS THALIANA CALMODULIN LIKE 4); encodes a calmodulin-like protein, with six	2.895	3.37E-09
At2g41110	CAM2 (calmodulin 2); Encodes a touch-inducible calmodulin that has higher affinity to kinesin-like calmoduli	2.021	3.44E-10
At2g41120	unknown protein	2.353	8.77E-09
At2g41160	Ubiquitin-associated (UBA) protein	2.771	1.36E-11
At2g41190	Transmembrane amino acid transporter family protein	1.198	6.86E-08
At2g41240	BHLH100 (basic helix-loop-helix protein 100); Encodes a member of the basic helix-loop-helix transcription f	2.086	0.00354
At2g41250	Haloacid dehalogenase-like hydrolase (HAD) superfamily protein	1.474	4.22E-06
At2g41280	M10; Encodes a hydrophilic protein similar to Late Embryogenesis Activated (LEA) proteins expressed during	1.304	4.48E-07
At2g41342	unknown protein	1.308	5.15E-06
At2g41440	unknown protein	1.018	1.17E-06
At2g41480	Peroxidase superfamily protein	-1.274	0.00141
At2g41510	ATCKX1 (CYTOKININ OXIDASE/DEHYDROGENASE 1); It encodes a protein whose sequence is similar t	-1.057	0.0018
At2g41540	GPDHC1 (6-phosphogluconate dehydrogenase family protein); Encodes a protein with NAD-dependent glycer	-1.035	2.31E-06
At2g41560	ACA4 (autoinhibited Ca(2+)-ATPase, isoform 4); encodes a calmodulin-regulated Ca(2+)-ATPase that improv	-1.733	1.95E-08
At2g41630	TFIIB (transcription factor IIB); Encodes the transcription factor TFIIB.	1.485	1.03E-07
At2g41640	Glycosyltransferase family 61 protein	1.645	5.02E-05
At2g41730	unknown protein	2.684	1.23E-06
At2g41835	zinc finger (C2H2 type, AN1-like) family protein	3.419	1.27E-11
At2g41880	GK-1 (guanylate kinase 1); Guanylate kinase. Involved in nucleotide metabolism.	1.054	3.88E-06
At2g41905	BEST Arabidopsis thaliana protein match is: arabinogalactan protein 23 (TAIR:AT3G57690.1)	1.203	2.67E-06
At2g41940	ZFP8 (zinc finger protein 8); Encodes a zinc finger protein containing only a single zinc finger.	-1.366	1.11E-07
At2g42005	Transmembrane amino acid transporter family protein	1.131	2.95E-06
At2g42150	DNA-binding bromodomain-containing protein	3.045	8.80E-11
At2g42170	Actin family protein	-1.361	2.76E-06
At2g42320	nucleolar protein gar2-related	-1.13	7.36E-07
At2g42330	GC-rich sequence DNA-binding factor-like protein with Tuftelin interacting domain	1.059	1.88E-05
At2g42350	RING/U-box superfamily protein	1.177	2.35E-06
At2g42380	BZIP34 (Basic-leucine zipper (bZIP) transcription factor family protein); Encodes a member of the BZIP famil	-1.305	8.29E-06
At2g42388	other RNA; Unknown gene	-1.234	9.14E-06
At2g42480	TRAF-like family protein	2.302	6.39E-11
At2g42530	COR15B (cold regulated 15b)	-2.693	6.06E-08
At2g42760	unknown protein	-1.003	6.05E-06
At2g42870	HLH1 (HELIX-LOOP-HELIX 1); Encodes PHYTOCHROME RAPIDLY REGULATED1 (PAR1), an atypical	-1.988	7.51E-07
At2g42900	Plant basic secretory protein (BSP) family protein	-1.989	6.23E-08
At2g42950	Magnesium transporter CorA-like family protein	1.883	6.06E-09
At2g42975	unknown protein	1.212	5.36E-05
At2g42990	GDSL-like Lipase/Acylhydrolase superfamily protein	-1.34	9.43E-06
At2g43000	anac042 (NAC domain containing protein 42)	1.129	0.00877
At2g43010	PIF4 (phytochrome interacting factor 4); Isolated as a semidominant mutation defective in red -light responses	-1.74	3.17E-07
At2g43060	IBH1 (ILI1 binding bHLH 1)	-1.142	1.18E-06
At2g43100	IPMI2 (isopropylmalate isomerase 2)	-2.114	1.03E-08
At2g43120	RmlC-like cupins superfamily protein	2.457	1.74E-07
At2g43160	ENTH/VHS family protein	1.28	6.29E-07
At2g43320	S-adenosyl-L-methionine-dependent methyltransferases superfamily protein	1.546	8.10E-07
At2g43445	F-box and associated interaction domains-containing protein	-1.508	8.73E-08
At2g43500	Plant regulator RWP-RK family protein	2.918	1.03E-10
At2g43530	Scorpion toxin-like knottin superfamily protein; Encodes a defensin-like (DEFL) family protein.	-1.068	6.96E-07
At2g43535	Scorpion toxin-like knottin superfamily protein; Encodes a defensin-like (DEFL) family protein.	-1.115	0.01549
At2g43550	Scorpion toxin-like knottin superfamily protein; Encodes a defensin-like (DEFL) family protein.	-2.079	3.14E-09
At2g43570	CHI (chitinase, putative)	-1.394	0.0251
At2g43590	Chitinase family protein	-1.217	0.005
At2g43600	Chitinase family protein	-1.106	0.00017

At2g43620	Chitinase family protein	-1.083	0.02405
At2g43630	FUNCTIONS IN: molecular_function unknown; INVOLVED IN: biological_process unknown; LOCATED IN	1.236	6.85E-08
At2g43880	Pectin lyase-like superfamily protein	-1.212	8.71E-05
At2g43900	Endonuclease/exonuclease/phosphatase family protein	1.782	1.03E-08
At2g44170	ATNMT2 (ARABIDOPSIS N-MYRISTOYLTRANSFERASE 2)	2.455	4.81E-09
At2g44195	CBF1-interacting co-repressor CIR, N-terminal	1.357	3.18E-05
At2g44230	Plant protein of unknown function (DUF946)	-1.333	3.30E-06
At2g44460	BGLU28 (beta glucosidase 28)	5.374	1.27E-09
At2g44570	AtGH9B12 (glycosyl hydrolase 9B12)	-1.134	0.00031
At2g44600	unknown protein	1.349	1.55E-06
At2g44740	CYCP4.1 (cyclin p4.1)	-1.811	4.25E-08
At2g44840	ATERF13 (ETHYLENE-RESPONSIVE ELEMENT BINDING FACTOR 13); encodes a member of the ERF (	1.674	0.00222
At2g44970	alpha/beta-Hydrolases superfamily protein	1.385	2.02E-08
At2g45130	ATSPX3 (ARABIDOPSIS THALIANA SPX DOMAIN GENE 3)	-2.065	0.00456
At2g45135	RING/U-box superfamily protein	-1.22	0.02647
At2g45180	Bifunctional inhibitor/lipid-transfer protein/seed storage 2S albumin superfamily protein	-1.542	5.71E-08
At2g45450	ZPR1 (LITTLE ZIPPER 1); ZPR1, a small leucine zipper-containing protein that interacts with REV HD-ZIPII	-1.329	6.05E-09
At2g45500	AAA-type ATPase family protein	1.001	0.0495
At2g45720	ARM repeat superfamily protein	-1.001	0.0002
At2g45840	Arabidopsis thaliana protein of unknown function (DUF821)	2.289	1.93E-09
At2g46230	PIN domain-like family protein	1.376	2.95E-09
At2g46240	ATBAG6 (ARABIDOPSIS THALIANA BCL-2-ASSOCIATED ATHANOGENE 6); A member of Arabidopsi	5.307	3.40E-11
At2g46320	Mitochondrial substrate carrier family protein	1.082	2.74E-07
At2g46340	SPA1 (SUPPRESSOR OF PHYA-105 1); Encodes a member of the SPA (suppressor of phyA-105) protein fan	1.053	2.12E-06
At2g46400	ATWRKY46 (WRKY DNA-BINDING PROTEIN 46); member of WRKY Transcription Factor; Group III	3.413	2.07E-06
At2g46530	ARF11 (auxin response factor 11)	-1.118	9.43E-06
At2g46570	LAC6 (laccase 6); putative laccase, a member of laccase family of genes (with 17 members in Arabidopsis).	-1.076	1.45E-05
At2g46640	unknown protein	-1.641	1.22E-07
At2g46650	ATCB5-C (ARABIDOPSIS CYTOCHROME B5 ISOFORM C); member of Cytochromes b5	-2.649	3.69E-10
At2g46670	CCT motif family protein	1.972	7.46E-08
At2g46690	SAUR-like auxin-responsive protein family	-1.832	1.90E-08
At2g46735	unknown protein	3.643	6.05E-10
At2g46790	APRR9 (Arabidopsis pseudo-response regulator 9); Pseudo-response regulator PRR9. Involved in clock functi	1.624	0.0016
At2g46830	CCA1 (circadian clock associated 1); Encodes a transcriptional repressor that performs overlapping functions	4.167	1.81E-11
At2g46870	NGA1 (NGATHA1)	-1.413	6.86E-06
At2g46880	PAP14 (purple acid phosphatase 14)	-2.92	0.0052
At2g46915	Protein of unknown function (DUF3754)	1.524	3.33E-08
At2g47050	Plant invertase/pectin methylesterase inhibitor superfamily protein	1.074	6.75E-05
At2g47060	Protein kinase superfamily protein	1.618	4.77E-07
At2g47130	NAD(P)-binding Rossmann-fold superfamily protein	2.046	4.49E-07
At2g47140	NAD(P)-binding Rossmann-fold superfamily protein	3.079	7.28E-10
At2g47150	NAD(P)-binding Rossmann-fold superfamily protein	1.902	5.26E-07
At2g47180	AtGolS1 (galactinol synthase 1)	6.294	1.47E-12
At2g47270	sequence-specific DNA binding transcription factors	-1.166	0.007
At2g47280	Pectin lyase-like superfamily protein	1.831	2.17E-09
At2g47440	Tetratricopeptide repeat (TPR)-like superfamily protein	-1.25	0.00015
At2g47520	HRE2 (HYPOXIA RESPONSIVE ERF (ETHYLENE RESPONSE FACTOR) 2); encodes a member of the ER	2.683	1.65E-05
At2g47720	FUNCTIONS IN: molecular_function unknown; INVOLVED IN: biological_process unknown; LOCATED IN	1.253	0.00016
At2g47730	ATGSTF5 (GLUTATHIONE S-TRANSFERASE (CLASS PHI) 5); Encodes glutathione transferase belonging	2.172	0.0001
At2g47770	ATTSPO (TSPO(outer membrane tryptophan-rich sensory protein)-related); Encodes a membrane-bound prote	2.582	0.0049
At2g47800	ATMRP4 (multidrug resistance-associated protein 4); Encodes a plasma membrane localized ATPase transpor	1.372	4.70E-06
At2g48150	ATGPX4 (glutathione peroxidase 4); Encodes glutathione peroxidase.	1.831	4.61E-05
At3g01060	unknown protein	2.092	3.88E-09
At3g01070	ENODL16 (early nodulin-like protein 16)	4.493	2.55E-12
At3g01080	ATWRKY58 (WRKY DNA-BINDING PROTEIN 58); member of WRKY Transcription Factor; Group I	1.762	3.78E-07
At3g01175	Protein of unknown function (DUF1666)	2.68	1.43E-07
At3g01220	ATHB20 (homeobox protein 20); Encodes a homeodomain leucine zipper class I (HD-Zip I) protein. Expresse	1.029	3.39E-06
At3g01290	SPFH/Band 7/PHB domain-containing membrane-associated protein family	1.168	1.46E-05
At3g01311	Protein of unknown function (DUF569)	2.221	1.62E-08
At3g01322	ECA1-like gametogenesis related family protein; Encodes a ECA1 gametogenesis related family protein	1.601	4.68E-09
At3g01350	Major facilitator superfamily protein	-1.431	4.64E-09
At3g01400	ARM repeat superfamily protein	1.497	2.95E-08
At3g01420	ALPHA-DOX1 (alpha-dioxygenase 1); Encodes an alpha-dioxygenase involved in protection against oxidative	-1.868	0.0012
At3g01490	Protein kinase superfamily protein	-1.043	4.16E-06
At3g01500	ATBCA1 (BETA CARBONIC ANHYDRASE 1); Encodes a putative beta-carbonic anhydrase betaCA1. Toge	1.877	7.69E-05
At3g01550	ATPPT2 (PHOSPHOENOLPYRUVATE (PEP)/PHOSPHATE TRANSLOCATOR 2)	1.627	9.06E-06
At3g01690	alpha/beta-Hydrolases superfamily protein	-1.06	1.72E-06

At3g01750	Ankyrin repeat family protein	-1.023	2.90E-06
At3g01820	P-loop containing nucleoside triphosphate hydrolases superfamily protein	1.426	6.16E-07
At3g01830	Calcium-binding EF-hand family protein	2.119	0.0164
At3g01940	Protein of unknown function (DUF 3339)	-1.073	3.45E-06
At3g01960	unknown protein	-1.44	2.88E-05
At3g02020	AK3 (aspartate kinase 3); encodes a monofunctional aspartate kinase	-4.592	2.05E-11
At3g02040	SRG3 (senescence-related gene 3)	-1.476	0.0027
At3g02140	AFP4 (ABI FIVE BINDING PROTEIN 4); Encodes a protein that acts in the nucleus and is an important nega	1.563	3.39E-08
At3g02170	LNG2 (LONGIFOLIA2); Encodes LONGIFOLIA2 (LNG2). Regulates leaf morphology by promoting cell exp	-1.126	1.68E-05
At3g02380	ATCOL2 (CONSTANS-LIKE 2); homologous to the flowering-time gene CONSTANS (CO) encoding zinc-fi	1.29	8.67E-05
At3g02480	Late embryogenesis abundant protein (LEA) family protein	3.255	0.00867
At3g02555	unknown protein	1.28	2.30E-07
At3g02800	Tyrosine phosphatase family protein	1.05	0.001
At3g02840	ARM repeat superfamily protein	4.744	1.44E-09
At3g02860	zinc ion binding	1.829	8.64E-10
At3g02885	GASA5 (GAST1 protein homolog 5)	-1.015	0.00649
At3g02990	ATHSFA1E (heat shock transcription factor A1E); member of Heat Stress Transcription Factor (Hsf) family	4.851	1.30E-10
At3g03060	P-loop containing nucleoside triphosphate hydrolases superfamily protein	2.024	6.16E-08
At3g03110	XPO1B (exportin 1B); Encodes a member of the exportin family (XPO1B)which function as receptors for nuc	1.062	2.08E-05
At3g03190	ATGSTF11 (glutathione S-transferase F11); Encodes glutathione transferase belonging to the phi class of GST	-3.17	1.20E-09
At3g03240	alpha/beta-Hydrolases superfamily protein	1.583	2.81E-07
At3g03260	HDG8 (homeodomain GLABROUS 8); Encodes a homeobox-leucine zipper family protein belonging to the H	3.191	1.33E-07
At3g03270	Adenine nucleotide alpha hydrolases-like superfamily protein	2.192	2.22E-07
At3g03310	ATLCAT3 (ARABIDOPSIS LECITHIN:CHOLESTEROL ACYLTRANSFERASE 3)	1.882	1.89E-09
At3g03320	RNA-binding ASCH domain protein	1.376	7.98E-08
At3g03341	unknown protein	2.476	0.00051
At3g03440	ARM repeat superfamily protein	2.489	3.66E-09
At3g03470	CYP89A9 (cytochrome P450, family 87, subfamily A, polypeptide 9); member of CYP89A	-1.1	6.14E-05
At3g03510	Phototropic-responsive NPH3 family protein	1.458	2.92E-08
At3g03750	SDG20 (SET domain protein 20)	1.395	7.54E-09
At3g03820	SAUR-like auxin-responsive protein family	-1.674	3.72E-06
At3g03840	SAUR-like auxin-responsive protein family	-1.1	1.74E-05
At3g03850	SAUR-like auxin-responsive protein family	-1.597	1.13E-05
At3g03855	expressed protein, similar to GB:AAC72977 (Arabidopsis thaliana); pseudogene of disease resistance protein	3.446	3.31E-11
At3g03950	ECT1 (evolutionarily conserved C-terminal region 1); Physically interacts with CIPK1. Located in the nucleus	1.396	1.52E-07
At3g04000	NAD(P)-binding Rossmann-fold superfamily protein	1.284	1.61E-06
At3g04010	O-Glycosyl hydrolases family 17 protein	2.259	1.69E-09
At3g04050	Pyruvate kinase family protein	1.288	7.78E-08
At3g04100	AGL57 (AGAMOUS-like 57)	1.733	2.84E-08
At3g04110	GLR1 (GLUTAMATE RECEPTOR 1); putative glutamate receptor (GLR1.1). Contains a functional cation - p	1.509	6.13E-07
At3g04120	GAPC (GLYCERALDEHYDE-3-PHOSPHATE DEHYDROGENASE C SUBUNIT); encodes cytosolic GAD	1.033	0.0002
At3g04130	Tetratricopeptide repeat (TPR)-like superfamily protein	1.628	4.44E-09
At3g04200	RmlC-like cupins superfamily protein	3.83	9.43E-09
At3g04220	Disease resistance protein (TIR-NBS-LRR class) family	1.577	8.42E-07
At3g04300	RmlC-like cupins superfamily protein	2.222	2.92E-08
At3g04330	Kunitz family trypsin and protease inhibitor protein	2.333	9.07E-11
At3g04360	Calcium-dependent lipid-binding (CaLB domain) family protein	1.47	4.20E-06
At3g04380	SDG31 (SET DOMAIN PROTEIN 31); Encodes SUVR4, a nucleolar histone methyltransferase with preferen	1.238	7.22E-08
At3g04410	NAC (No Apical Meristem) domain transcriptional regulator superfamily protein	1.1	1.14E-06
At3g04530	PEPCK2 (PHOSPHOENOLPYRUVATE CARBOXYLASE KINASE 2); Encodes a second Arabidopsis phos	-1.226	0.03217
At3g04550	unknown protein	-1.29	2.73E-08
At3g04620	Alba DNA/RNA-binding protein	1.416	8.99E-07
At3g04710	ankyrin repeat family protein	3.375	7.58E-11
At3g04717	pseudogene, similar to pathogen- and wound-inducible antifungal protein CBP20 precursor, similar to pathoge	-1.493	0.00062
At3g04720	HEL (HEVEIN-LIKE); Encodes a protein similar to the antifungal chitin-binding protein hevein from rubber t	-1.407	5.42E-05
At3g04760	Pentatricopeptide repeat (PPR-like) superfamily protein	-1.075	1.12E-07
At3g04910	WNK1 (with no lysine (K) kinase 1); Serine/threonine protein kinase, whose transcription is regulated by circ	-1.643	1.94E-08
At3g04980	DNAJ heat shock N-terminal domain-containing protein	-1.153	1.32E-07
At3g05030	NHX2 (sodium hydrogen exchanger 2); member of Sodium proton exchanger family	2.595	1.01E-10
At3g05140	RBK2 (ROP binding protein kinases 2)	-1.358	3.74E-06
At3g05150	Major facilitator superfamily protein	-1.083	0.0007
At3g05160	Major facilitator superfamily protein	-1.522	3.24E-09
At3g05165	Major facilitator superfamily protein	1.567	2.12E-07
At3g05200	ATL6 (RING/U-box superfamily protein); Encodes a putative RING-H2 zinc finger protein ATL6 (ATL6).	1.122	2.39E-05
At3g05220	Heavy metal transport/detoxification superfamily protein	-1.011	0.0011
At3g05360	AtRLP30 (receptor like protein 30)	2.942	2.21E-07
At3g05390	FUNCTIONS IN: molecular_function unknown; INVOLVED IN: biological_process unknown; LOCATED IN	1.889	9.54E-09

At3g05400	Major facilitator superfamily protein	3.032	9.08E-10
At3g05430	Tudor/PWWP/MBT superfamily protein	1.076	1.37E-05
At3g05500	Rubber elongation factor protein (REF)	1.328	4.41E-06
At3g05580	Calcineurin-like metallo-phosphoesterase superfamily protein	2.128	3.68E-10
At3g05630	PDLZ2 (PHOSPHOLIPASE D ZETA 2); Encodes a member of the PXPH-PLD subfamily of phospholipase D	-1.606	0.00017
At3g05770	unknown protein	1.122	9.03E-05
At3g05800	AIF1 (AtBS1(activation-tagged BRI1 suppressor 1)-interacting factor 1)	-1.232	9.38E-06
At3g05880	RCI2A (RARE-COLD-INDUCIBLE 2A); Induced by low temperatures, dehydration and salt stress and ABA.	-1.743	7.58E-06
At3g05905	other RNA; Potential natural antisense gene, locus overlaps with AT3G05900	2.67	1.00E-09
At3g06070	unknown protein	-1.981	6.29E-07
At3g06420	ATG8H (autophagy 8h)	2.882	3.46E-10
At3g06540	REP (Rab escort protein)	1.332	1.20E-08
At3g06660	PAPA-1-like family protein / zinc finger (HIT type) family protein	1.447	1.15E-07
At3g06750	hydroxyproline-rich glycoprotein family protein	-1.285	2.42E-06
At3g06760	Drought-responsive family protein	1.623	8.30E-08
At3g06780	glycine-rich protein	1.054	9.99E-06
At3g06985	LCR44 (low-molecular-weight cysteine-rich 44); Encodes a member of a family of small,secreted, cysteine ricl	2.18	1.39E-08
At3g06995	Encodes a Defensin-like (DEFL) family protein [pseudogene]; Encodes a Defensin-like (DEFL) family protein	1.716	1.35E-07
At3g07000	Cysteine/Histidine-rich C1 domain family protein	-1.157	5.13E-06
At3g07010	Pectin lyase-like superfamily protein	-1.603	2.74E-07
At3g07090	PPPDE putative thiol peptidase family protein	4.375	2.31E-12
At3g07130	ATPAP15 (PURPLE ACID PHOSPHATASE 15); Encodes PAP15, a purple acid phosphatase with phytase ac	-1.175	1.86E-07
At3g07150	unknown protein	6.45	2.65E-14
At3g07195	RPM1-interacting protein 4 (RIN4) family protein	2.708	5.48E-08
At3g07340	basic helix-loop-helix (bHLH) DNA-binding superfamily protein	-1.206	5.61E-05
At3g07370	ATCHIP (CARBOXYL TERMINUS OF HSC70-INTERACTING PROTEIN); Encodes AtCHIP, a new class c	1.715	6.46E-09
At3g07460	Protein of unknown function, DUF538	-1.046	2.74E-06
At3g07490	AGD11 (ARF-GAP domain 11); A member of ARF GAP domain (AGD), A thaliana has 15 members, groupe	3.213	4.07E-11
At3g07565	Protein of unknown function (DUF3755)	1.019	3.18E-07
At3g07650	COL9 (CONSTANS-like 9); This gene belongs to the CO (CONSTANS) gene family. This gene family is divi	-1.255	3.59E-06
At3g07720	Galactose oxidase/kelch repeat superfamily protein	1.321	7.31E-06
At3g07770	AtHsp90-6 (HEAT SHOCK PROTEIN 90-6)	1.394	8.60E-06
At3g07780	OBE1 (OBERON1); Encodes a nuclear PHD finger protein that is functionally redundant with OBE2 and play:	1.191	2.60E-08
At3g07870	F-box and associated interaction domains-containing protein	1.471	6.79E-07
At3g07890	Ypt/Rab-GAP domain of gyp1p superfamily protein	1.156	4.15E-06
At3g07990	SCPL27 (serine carboxypeptidase-like 27)	-1.064	3.65E-07
At3g08490	BEST Arabidopsis thaliana protein match is: Late embryogenesis abundant protein, group 2 (TAIR:AT3G246(	-1.275	4.42E-08
At3g08505	zinc finger (CCCH-type/C3HC4-type RING finger) family protein	1.189	8.19E-08
At3g08570	Phototropic-responsive NPH3 family protein	2.01	2.81E-08
At3g08590	Phosphoglycerate mutase, 2,3-bisphosphoglycerate-independent	2.543	2.84E-08
At3g08660	Phototropic-responsive NPH3 family protein	-1.967	1.67E-08
At3g08690	UBC11 (ubiquitin-conjugating enzyme 11)	3.357	4.83E-11
At3g08700	UBC12 (ubiquitin-conjugating enzyme 12)	4.226	0.00011
At3g08710	ATH9 (thioredoxin H-type 9); Associated to plasma membrane. Moves cell to cell, suggesting a role in interce	1.931	4.42E-09
At3g08720	ATPK19 (Arabidopsis thaliana protein kinase 19); Encodes a ribosomal-protein S6 kinase. Gene expression is	1.102	3.77E-05
At3g08730	ATPK1 (ARABIDOPSIS THALIANA PROTEIN-SERINE KINASE 1); Encodes a protein-serine kinase that p	2.054	1.98E-09
At3g08860	PYD4 (PYRIMIDINE 4); Encodes a protein that is predicted to have beta-alanine aminotransferase activity.	1.401	0.002
At3g08960	ARM repeat superfamily protein	1.805	0.0015
At3g08970	TMS1 (THERMOSENSITIVE MALE STERILE 1); J domain protein localized in ER lumen. Can compensate	6.603	5.86E-12
At3g09000	proline-rich family protein	1.543	8.70E-09
At3g09010	Protein kinase superfamily protein	2.235	5.02E-09
At3g09030	BTB/POZ domain-containing protein	1.148	4.42E-08
At3g09085	Protein of unknown function (DUF962)	1.957	2.72E-09
At3g09220	LAC7 (laccase 7); putative laccase, a member of laccase family of genes (17 members in Arabidopsis).	-2.033	5.60E-07
At3g09230	AtMYB1 (myb domain protein 1); member of MYB3R- and R2R3- type MYB- encoding genes	2.225	1.51E-10
At3g09280	unknown protein	1.393	3.71E-05
At3g09290	TAC1 (telomerase activator1); encodes activation factor TAC1 which mediates telomerase activity	-1.104	0.0013
At3g09350	Fes1A (Fes1A); Encodes one of the Arabidopsis orthologs of the human Hsp70-binding protein 1 (HspBP-1) a	5.714	7.06E-13
At3g09370	AtMYB3R3 (myb domain protein 3R3); putative c-myb-like transcription factor (MYB3R3) mRNA,	1.545	0.003
At3g09385	pseudogene, hypothetical protein	1.306	5.15E-09
At3g09400	PLL3 (pol-like 3); Similar to POLTERGEIST (POL) protein phosphatase 2C. No phenotype observed in plants	1.278	6.90E-08
At3g09440	Heat shock protein 70 (Hsp 70) family protein	2.203	6.88E-07
At3g09450	CONTAINS InterPro DOMAIN/s: Fusaric acid resistance protein, conserved region (InterPro:IPR006726); BE	2.95	1.09E-08
At3g09490	Tetratricopeptide repeat (TPR)-like superfamily protein	1.401	1.58E-06
At3g09520	ATEXO70H4 (exocyst subunit exo70 family protein H4); A member of EXO70 gene family, putative exocyst :	5.242	5.91E-12
At3g09550	Ankyrin repeat family protein	-1.448	3.51E-08
At3g09560	ATPAH1 (PHOSPHATIDIC ACID PHOSPHOHYDROLASE 1)	1.871	2.99E-10

At3g09570	Lung seven transmembrane receptor family protein	1.125	3.74E-08
At3g09580	FAD/NAD(P)-binding oxidoreductase family protein	-1.116	1.69E-07
At3g09600	Homeodomain-like superfamily protein	2.344	3.35E-08
At3g09640	APX1B (ASCORBATE PEROXIDASE 1B); Encodes a cytosolic ascorbate peroxidase APX2. Ascorbate perc	10.595	1.84E-12
At3g09830	Protein kinase superfamily protein	1.437	0.0042
At3g09840	CDC48 (cell division cycle 48); Encodes a cell division cycle protein, a member of AAA-type ATPases gene fa	1.151	2.76E-06
At3g09870	SAUR-like auxin-responsive protein family	2.263	4.31E-06
At3g09922	ATIPS1 (A. THALIANA INDUCED BY PHOSPHATE STARVATION1); AF236376 Arabidopsis thaliana IP	-1.825	0.01653
At3g10020	unknown protein	1.941	2.81E-07
At3g10080	RmlC-like cupins superfamily protein	-1.26	6.13E-07
At3g10114	pseudogene of glycosyl hydrolase family 81 protein; pseudogene of glycosyl hydrolase family 81 protein	3.125	6.21E-11
At3g10150	PAP16 (purple acid phosphatase 16)	-1.418	0.00013
At3g10250	Plant protein 1589 of unknown function	1.367	9.63E-09
At3g10450	SCPL7 (serine carboxypeptidase-like 7)	1.122	6.49E-06
At3g10500	anac053 (NAC domain containing protein 53)	1.607	4.22E-08
At3g10510	Galactose oxidase/kelch repeat superfamily protein	1.602	2.42E-06
At3g10520	AHB2 (haemoglobin 2); class 2 non-symbiotic hemoglobin	-1.259	1.42E-06
At3g10525	LGO (LOSS OF GIANT CELLS FROM ORGANS); Encodes LGO (loss of giant cells from organs) required fo	-1.131	2.92E-05
At3g10640	VPS60.1 (SNF7 family protein)	2.752	4.44E-12
At3g10800	BZIP28 (Basic-leucine zipper (bZIP) transcription factor family protein); Encodes bZIP28, a putative membran	1.346	1.70E-07
At3g10815	RING/U-box superfamily protein	3.391	2.13E-09
At3g10820	Transcription elongation factor (TFIIS) family protein	3.008	1.11E-10
At3g10830	NA	1.968	4.45E-09
At3g10985	ATWI-12 (ARABIDOPSIS THALIANA WOUND-INDUCED PROTEIN 12); A senescence-associated gene w	2.039	1.85E-07
At3g11010	AtRLP34 (receptor like protein 34)	1.181	0.00207
At3g11020	DREB2 (DEHYDRATION-RESPONSIVE ELEMENT BINDING PROTEIN 2); encodes a member of the DRI	1.974	5.80E-08
At3g11080	AtRLP35 (receptor like protein 35)	2.018	1.26E-06
At3g11150	2-oxoglutarate (2OG) and Fe(II)-dependent oxygenase superfamily protein	-1.039	5.58E-05
At3g11310	unknown protein	2.068	2.06E-11
At3g11320	Nucleotide-sugar transporter family protein	1.046	3.25E-05
At3g11440	ATMYB65 (MYB DOMAIN PROTEIN 65); Member of the R2R3-MYB gene family. Similar to GA-induced	1.573	5.20E-09
At3g11660	NHL1 (NDR1/HIN1-like 1); encodes a protein whose sequence is similar to tobacco hairpin-induced gene (HI	-1.186	0.0001
At3g11700	FLA18 (FASCICLIN-like arabinogalactan protein 18 precursor)	-1.024	7.82E-06
At3g11820	ATSYR1 (SYNTAXIN RELATED PROTEIN 1); Encodes a syntaxin localized at the plasma membrane (SYR	3.201	2.81E-10
At3g11840	PUB24 (plant U-box 24); Encodes a U-box-domain-containing E3 ubiquitin ligase that acts as a negative regul	1.127	0.0056
At3g11900	ANT1 (aromatic and neutral transporter 1); encodes an amino acid transporter that transports aromatic and neu	1.316	3.56E-08
At3g12040	DNA-3-methyladenine glycosylase (MAG)	2.98	1.15E-11
At3g12050	Aha1 domain-containing protein	3.592	3.70E-12
At3g12060	TBL1 (TRICHOME BIREFRINGENCE-LIKE 1); Encodes a member of the TBL (TRICHOME BIREFRINGE	1.122	0.00036
At3g12190	BEST Arabidopsis thaliana protein match is: Frigida-like protein (TAIR:AT5G27220.1)	2.248	0.00122
At3g12300	unknown protein	1.212	3.51E-07
At3g12320	unknown protein	3.518	2.84E-11
At3g12510	MADS-box family protein	2.609	2.05E-09
At3g12520	SULTR4.2 (sulfate transporter 4.2); Encodes a sulfate transporter that in induced under sulfate limitation.	2.227	3.27E-10
At3g12580	ATHSP70 (ARABIDOPSIS HEAT SHOCK PROTEIN 70)	6.38	2.70E-12
At3g12610	DRT100 (DNA-DAMAGE REPAIR/TOLERATION 100); Plays role in DNA-damage repair/toleration. Partial	-1.571	1.22E-06
At3g12630	A20/AN1-like zinc finger family protein	1.101	9.17E-08
At3g12710	DNA glycosylase superfamily protein	-1.128	5.41E-06
At3g12740	ALIS1 (ALA-interacting subunit 1); Physically interacts with ALA3, and is required for the phospholipid trans	2.639	2.01E-10
At3g12750	ZIP1 (zinc transporter 1 precursor); A member of Zrt- and Irt-related protein (ZIP) family. transcript is induce	-1.169	1.45E-07
At3g12770	MEF22 (mitochondrial editing factor 22); Encodes a pentatricopeptide repeat protein (PPR) protein involved i	-1.024	2.15E-05
At3g12820	AtMYB10 (myb domain protein 10); Member of the R2R3 factor gene family.	-1.633	4.90E-07
At3g12830	SAUR-like auxin-responsive protein family	-1.027	2.33E-06
At3g12910	NAC (No Apical Meristem) domain transcriptional regulator superfamily protein	4.451	2.88E-08
At3g12915	Ribosomal protein S5/Elongation factor G/III/V family protein	-1.122	4.82E-06
At3g12920	SBP (S-ribonuclease binding protein) family protein	-1.465	3.75E-08
At3g13061	other RNA; Potential natural antisense gene, locus overlaps with AT3G13060	-1.517	0.0005
At3g13080	ATMRP3 (multidrug resistance-associated protein 3); encodes an ATP-dependent MRP-like ABC transporter a	1.192	0.0002
At3g13130	unknown protein	1.709	2.51E-07
At3g13227	serine-rich protein-related	2.794	1.64E-08
At3g13229	Plant protein of unknown function (DUF868)	2.462	2.92E-09
At3g13235	ubiquitin family protein	1.777	5.83E-08
At3g13330	PA200 (proteasome activating protein 200); Encodes a protein that interacts with the 26S proteasome. Mutant	1.534	0.0002
At3g13470	TCP-1/cpn60 chaperonin family protein	1.326	1.33E-06
At3g13610	2-oxoglutarate (2OG) and Fe(II)-dependent oxygenase superfamily protein	2.304	0.00465
At3g13672	TRAF-like superfamily protein	-1.272	3.12E-05
At3g13682	LDL2 (LSD1-like2); Encodes a homolog of human Lysine-Specific Demethylase1. Involved in H3K4 methyla	-1.053	3.32E-07

At3g13720	PRA1.F3 (PRENYLATED RAB ACCEPTOR 1.F3)	-1.04	0.0015
At3g13730	CYP90D1 (cytochrome P450, family 90, subfamily D, polypeptide 1); Encodes a cytochrome P-450 gene that i	-1.081	7.30E-06
At3g13740	Ribonuclease III family protein	-1.014	4.76E-07
At3g13750	BGAL1 (beta galactosidase 1); beta-galactosidase, glycosyl hydrolase family 35	-1.614	3.56E-07
At3g13820	F-box and associated interaction domains-containing protein	-1.055	1.96E-07
At3g13850	LBD22 (LOB domain-containing protein 22)	1.263	1.80E-05
At3g13910	Protein of unknown function (DUF3511)	1.345	4.48E-07
At3g13980	unknown protein	-1.16	1.46E-06
At3g14075	Mono-/di-acylglycerol lipase, N-terminal	1.163	0.003
At3g14200	Chaperone DnaJ-domain superfamily protein	3.96	4.02E-12
At3g14210	ESM1 (epithiospecifier modifier 1); A semidominant QTL which has an epistatic effect on the Epithiospecifier	-1.409	8.27E-06
At3g14225	GLIP4 (GDSL-motif lipase 4); Contains lipase signature motif and GDSL domain.	1.594	9.07E-09
At3g14280	unknown protein	-1.488	6.01E-06
At3g14300	ATPME26 (A. THALIANA PECTIN METHYLESTERASE 26)	1.958	1.83E-06
At3g14310	ATPME3 (pectin methylesterase 3); encodes a pectin methylesterase, targeted by a cellulose binding protein (C	-1.388	2.47E-06
At3g14370	WAG2 (Protein kinase superfamily protein); The WAG2 and its homolog, WAG1 each encodes protein-serine	-2.017	5.40E-08
At3g14420	Aldolase-type TIM barrel family protein	1.362	4.26E-05
At3g14560	unknown protein	-1.882	2.92E-09
At3g14620	CYP72A8 (cytochrome P450, family 72, subfamily A, polypeptide 8); putative cytochrome P450	1.772	1.15E-07
At3g14630	CYP72A9 (cytochrome P450, family 72, subfamily A, polypeptide 9); putative cytochrome P450	2.21	8.29E-09
At3g14730	Pentatricopeptide repeat (PPR) superfamily protein	-1.04	3.97E-07
At3g14820	GDSL-like Lipase/Acylhydrolase superfamily protein	1.33	0.00043
At3g14850	TBL41 (TRICHOME BIREFRINGENCE-LIKE 41); Encodes a member of the TBL (TRICHOME BIREFRINC	1.277	2.41E-06
At3g15060	AtRABA1g (RAB GTPase homolog A1G)	1.284	3.84E-09
At3g15080	Polynucleotidyl transferase, ribonuclease H-like superfamily protein	1.11	5.11E-07
At3g15340	PPI2 (proton pump interactor 2); Hypothetical protein similar to PPI1, a protein that interacts with the plasma	5.03	8.74E-12
At3g15356	Legume lectin family protein	1.821	3.02E-06
At3g15440	BEST Arabidopsis thaliana protein match is: RING/U-box superfamily protein (TAIR:AT3G15740.1)	-1.855	2.19E-06
At3g15450	Aluminium induced protein with YGL and LRDR motifs	-2.443	7.26E-08
At3g15500	ANAC055 (NAC domain containing protein 55); Encodes an ATAF-like NAC-domain transcription factor tha	-1.91	3.00E-05
At3g15510	ANAC056 (Arabidopsis NAC domain containing protein 56); Note of caution: not to be confused with another	-1.14	0.0005
At3g15518	unknown protein	4.337	9.00E-11
At3g15570	Phototropic-responsive NPH3 family protein	-1.281	3.12E-07
At3g15630	unknown protein	-2.289	1.22E-06
At3g15720	Pectin lyase-like superfamily protein	-1.51	2.56E-06
At3g15770	unknown protein	1.189	6.41E-08
At3g15840	PIFI (post-illumination chlorophyll fluorescence increase); Encodes a chloroplast-targeted protein localized in	-1.493	4.09E-05
At3g16030	CES101 (CALLUS EXPRESSION OF RBCS 101)	1.347	3.10E-07
At3g16040	Translation machinery associated TMA7	1.716	0.00135
At3g16050	ATPDX1.2 (ARABIDOPSIS THALIANA PYRIDOXINE BIOSYNTHESIS 1.2); Encodes a protein with pyrid	4.433	3.76E-12
At3g16175	Thioesterase superfamily protein	1.789	3.51E-07
At3g16180	Major facilitator superfamily protein	-1.934	8.67E-09
At3g16240	DELTA-TIP (delta tonoplast integral protein); Delta tonoplast intrinsic protein, functions as a water channel ar	-1.09	1.33E-05
At3g16370	GDSL-like Lipase/Acylhydrolase superfamily protein	-1.54	3.56E-06
At3g16510	Calcium-dependent lipid-binding (CaLB domain) family protein	2.468	2.30E-08
At3g16530	Legume lectin family protein; Lectin like protein whose expression is induced upon treatment with chitin oligo	1.439	1.58E-05
At3g16570	ATRALF23 (ARABIDOPSIS RAPID ALKALINIZATION FACTOR 23); Encodes RALF23, a member of a di	-1.201	6.23E-05
At3g16690	Nodulin MtN3 family protein	-1.174	1.63E-05
At3g16770	ATEBP (ethylene-responsive element binding protein); Encodes a member of the ERF (ethylene response facto	-1.334	2.40E-07
At3g17010	AP2/B3-like transcriptional factor family protein; transcriptional factor B3 family protein, contains Pfam profil	1.124	9.39E-07
At3g17050	Arabidopsis thaliana (pseudogene, glycine-rich protein, similar to glycine-rich protein TIGR:At1g53620.1)	-1.512	5.51E-06
At3g17070	Peroxidase family protein	-1.876	1.69E-05
At3g17110	pseudogene, glycine-rich protein	1.73	0.0025
At3g17185	TAS3 (trans-acting siRNA3); Encodes a trans-acting siRNA (tasi-RNA) that regulates the expression of auxin	-1.012	1.38E-05
At3g17190	unknown protein	-1.053	2.94E-07
At3g17210	ATHS1 (A. THALIANA HEAT STABLE PROTEIN 1); Encodes a heat stable protein with antimicrobial and a	1.013	0.0003
At3g17330	ECT6 (evolutionarily conserved C-terminal region 6)	1.657	1.78E-08
At3g17400	F-box family protein	5.419	3.26E-12
At3g17410	Protein kinase superfamily protein	1.605	1.06E-07
At3g17520	Late embryogenesis abundant protein (LEA) family protein	1.458	0.03449
At3g17609	HYH (HY5-homolog); Encodes a homolog of HY5 (HYH). Involved in phyB signaling pathway.	2.55	6.21E-09
At3g17611	ATRBL14 (RHOMBOID-like protein 14)	4.219	4.08E-12
At3g17700	ATCNGC20 (CYCLIC NUCLEOTIDE-GATED CHANNEL 20); cyclic nucleotide-binding transporter 1, mem	1.834	4.64E-09
At3g17710	F-box and associated interaction domains-containing protein	1.447	3.23E-06
At3g17720	Pyridoxal phosphate (PLP)-dependent transferases superfamily protein	2.806	6.70E-10
At3g17800	Protein of unknown function (DUF760); mRNA level of the MEB5.2 gene (At3g17800) remains unchanged af	1.946	1.76E-07
At3g17980	Calcium-dependent lipid-binding (CaLB domain) family protein	3.645	6.27E-10

At3g18050	unknown protein	-1.096	2.31E-06
At3g18080	BGLU44 (B-S glucosidase 44)	-1.121	1.57E-06
At3g18290	BTS (BRUTUS); Encodes BRUTUS (BTS), a putative E3 ligase protein with metal ion binding and DNA binc	1.556	3.04E-05
At3g18295	Protein of unknown function (DUF1639)	1.481	1.96E-07
At3g18320	F-box and associated interaction domains-containing protein	-1.049	8.70E-06
At3g18370	ATSYTF (C2 domain-containing protein)	1.31	3.58E-07
At3g18400	anac058 (NAC domain containing protein 58)	1.024	5.02E-05
At3g18570	Oleosin family protein	1.048	3.63E-05
At3g18710	ATPUB29 (ARABIDOPSIS THALIANA PLANT U-BOX 29); Encodes a protein containing a U-box and an A	1.271	9.50E-06
At3g18850	LPAT5 (lysophosphatidyl acyltransferase 5)	1.065	1.15E-05
At3g19010	2-oxoglutarate (2OG) and Fe(II)-dependent oxygenase superfamily protein	1.48	5.56E-07
At3g19030	unknown protein	-1.328	1.28E-06
At3g19040	HAF2 (histone acetyltransferase of the TAFII250 family 2); Encodes a protein similar to TATA-binding protei	1.868	8.72E-08
At3g19160	ATIPT8 (ATP/ADP isopentenyltransferases); Encodes cytokinin synthase.	2.557	2.31E-06
At3g19240	Vacuolar import/degradation, Vid27-related protein	1.027	0.0001
At3g19260	LAG1 HOMOLOG 2 (LONGEVITY ASSURANCE GENE1 HOMOLOG 2); LAG1 homolog . Loss of functio	1.853	1.20E-08
At3g19270	CYP707A4 (cytochrome P450, family 707, subfamily A, polypeptide 4); Encodes a protein with ABA 8'-hydro	1.878	1.12E-06
At3g19350	MPC (maternally expressed pab C-terminal); Encodes a the C-terminal domain of poly(A) binding proteins. M	-2.185	2.87E-07
At3g19400	Cysteine proteinases superfamily protein	-1.027	3.10E-06
At3g19710	BCAT4 (branched-chain aminotransferase4); Belongs to the branched-chain amino acid aminotransferase gene	-2.851	1.61E-11
At3g19830	NTMC2T5.2 (Calcium-dependent lipid-binding (CaLB domain) family protein)	1.607	6.00E-08
At3g19850	Phototropic-responsive NPH3 family protein	-2.646	2.93E-10
At3g19960	ATM1 (myosin 1); member of Myosin-like proteins	-1.203	2.01E-06
At3g19970	alpha/beta-Hydrolases superfamily protein	1.393	1.52E-06
At3g20015	Eukaryotic aspartyl protease family protein	-1.188	2.35E-06
At3g20180	Copper transport protein family	4.48	2.03E-10
At3g20190	Leucine-rich repeat protein kinase family protein	1.947	2.45E-09
At3g20210	DELTA-VPE (delta vacuolar processing enzyme); Encodes a vacuolar processing enzyme with caspase-1-like a	-1.103	0.0063
At3g20470	GRP-5 (GLYCINE-RICH PROTEIN 5); encodes a glycine-rich protein that is expressed more abundantly in ir	-1.118	0.0013
At3g20520	SVL3 (SHV3-like 3)	1.085	0.00189
At3g20810	2-oxoglutarate (2OG) and Fe(II)-dependent oxygenase superfamily protein	-1.878	2.43E-09
At3g20820	Leucine-rich repeat (LRR) family protein	-1.409	9.85E-09
At3g21070	ATNADK-1 (NAD KINASE 1); Encodes a protein with NAD(H) kinase activity.	2.171	3.70E-09
At3g21150	BBX32 (B-box 32); Encodes a protein with a B-box domain predicted to act as a transcription factor. Expressi	1.719	0.00068
At3g21310	Core-2/I-branching beta-1,6-N-acetylglucosaminyltransferase family protein	-1.081	3.14E-06
At3g21460	Glutaredoxin family protein	-1.657	0.0002
At3g21500	DXPS1 (1-deoxy-D-xylulose 5-phosphate synthase 1); Encodes a protein postulated to have 1-deoxy-D-xylulos	-2.123	0.00014
At3g21530	DNAse I-like superfamily protein	-1.402	2.68E-06
At3g21620	ERD (early-responsive to dehydration stress) family protein	-1.857	0.00059
At3g21630	CERK1 (chitin elicitor receptor kinase 1); LysM receptor-like kinase. Essential in the perception and transduct	1.523	3.22E-09
At3g21680	unknown protein	-1.173	9.68E-06
At3g21700	SGP2 (Ras-related small GTP-binding family protein); Monomeric G protein. Expressed in root epidermal cell	3.76	2.75E-11
At3g21780	UGT71B6 (UDP-glucosyl transferase 71B6); Encodes a protein with UDP-glucosyl transferase activity that wa	1.78	4.64E-07
At3g21781	other RNA; Potential natural antisense gene, locus overlaps with AT3G21780	1.158	9.21E-06
At3g21860	ASK10 (SKP1-like 10)	1.692	7.62E-05
At3g21870	CYCP2.1 (cyclin p2.1)	1.166	0.0006
At3g21890	B-box type zinc finger family protein	1.007	0.00104
At3g21900	Receptor-like protein kinase-related family protein	3.279	2.61E-07
At3g21920	Domain of unknown function (DUF26)	3.269	4.22E-09
At3g21930	Domain of unknown function (DUF26)	3.242	9.89E-09
At3g22060	Receptor-like protein kinase-related family protein; contains Pfam profile: PF01657 Domain of unknown funct	3.461	1.59E-08
At3g22090	unknown protein	3.233	4.38E-11
At3g22100	basic helix-loop-helix (bHLH) DNA-binding superfamily protein	2.482	2.89E-09
At3g22142	Bifunctional inhibitor/lipid-transfer protein/seed storage 2S albumin superfamily protein; Encodes a Protease i	-1.891	1.62E-06
At3g22160	VQ motif-containing protein	1.19	0.00013
At3g22260	Cysteine proteinases superfamily protein	1.348	2.30E-07
At3g22360	AOX1B (alternative oxidase 1B); encodes an alternative oxidase whose expression is limited to flowers and flo	2.434	3.43E-08
At3g22370	AOX1A (alternative oxidase 1A); Encodes AOX1a, an isoform of alternative oxidase that is expressed in roset	3.152	9.08E-09
At3g22510	Pre-rRNA-processing protein TSR2, conserved region	3.331	2.20E-09
At3g22740	HMT3 (homocysteine S-methyltransferase 3); homocysteine S-methyltransferase (HMT3)	-4.794	4.81E-10
At3g22750	Protein kinase superfamily protein	-1.081	1.08E-05
At3g22820	allergen-related	-1.164	2.63E-05
At3g22830	AT-HSFA6B (ARABIDOPSIS THALIANA HEAT SHOCK TRANSCRIPTION FACTOR A6B); member of H	-1.106	1.97E-06
At3g22840	ELIP1 (EARLY LIGHT-INDUCABLE PROTEIN); Encodes an early light-inducible protein.	5.882	6.15E-12
At3g22890	APS1 (ATP sulfurylase 1); encodes ATP sulfurylase, the first enzyme in the sulfate assimilation pathway of Ar	1.037	1.23E-05
At3g22910	ATPase E1-E2 type family protein / haloacid dehalogenase-like hydrolase family protein	1.968	0.01289
At3g22920	Cyclophilin-like peptidyl-prolyl cis-trans isomerase family protein	3.928	5.10E-09

At3g22930	CML11 (calmodulin-like 11); Encodes a calmodulin-like protein.	2.986	3.33E-09
At3g22950	ARFC1 (ADP-ribosylation factor C1); A member of ARF GTPase family. A thaliana has 21 members of this fa	1.017	1.97E-07
At3g23000	CIPK7 (CBL-interacting protein kinase 7); Encodes a serine/threonine protein kinase with similarities to CBL-	1.03	8.25E-07
At3g23020	Tetratricopeptide repeat (TPR)-like superfamily protein	-1.194	1.22E-06
At3g23030	IAA2 (indole-3-acetic acid inducible 2); auxin inducible gene expressed in the nucleus	-1.86	2.16E-08
At3g23050	AXR2 (AUXIN RESISTANT 2); Transcription regulator acting as repressor of auxin-inducible gene expressio	-1.155	2.33E-06
At3g23110	AtRLP37 (receptor like protein 37)	-1.122	3.45E-05
At3g23120	AtRLP38 (receptor like protein 38)	-1.926	0.00224
At3g23230	Integrase-type DNA-binding superfamily protein; encodes a member of the ERF (ethylene response factor) sub	4.932	3.23E-09
At3g23250	ATMYB15 (MYB DOMAIN PROTEIN 15); Member of the R2R3 factor gene family.	1.571	9.09E-06
At3g23450	unknown protein	-1.137	0.0046
At3g23470	Cyclopropane-fatty-acyl-phospholipid synthase	-1.066	0.0207
At3g23480	Cyclopropane-fatty-acyl-phospholipid synthase	-2.273	4.95E-09
At3g23870	Protein of unknown function (DUF803)	-2.018	4.73E-06
At3g23880	F-box and associated interaction domains-containing protein	-1.728	3.44E-06
At3g23920	BAM1 (beta-amylase 1); Encodes a chloroplast beta-amylase. Is necessary for leaf starch breakdown in the abs	2.011	4.40E-09
At3g23980	BLI (BLISTER); Encodes a protein that interacts with the Polycomb-group (Pc-G) histone methyltransferase C	1.069	1.07E-07
At3g23990	HSP60 (heat shock protein 60); mitochondrial chaperonin HSP. assist in rapid assembly of the oligomeric pro	1.905	2.36E-07
At3g24005	pseudogene, chaperonin hsp60 (fragment), blastp match of 100% identity and 8.1e-06 P-value to GP|2275832	2.003	3.63E-08
At3g24090	glutamine-fructose-6-phosphate transaminase (isomerizing)s	2.637	2.83E-10
At3g24100	Uncharacterised protein family SERF	1.688	3.35E-07
At3g24140	FMA (FAMA); Encodes a basic helix-loop-helix transcription factor whose activity is required to promote diff	-1.503	2.56E-07
At3g24170	ATGR1 (glutathione-disulfide reductase); Encodes a cytosolic glutathione reductase.	1.799	6.50E-09
At3g24230	Pectate lyase family protein	-1.239	5.30E-06
At3g24420	alpha/beta-Hydrolases superfamily protein	2.601	1.35E-07
At3g24495	ATMSH7 (ARABIDOPSIS THALIANA MUTS HOMOLOG 7); encodes a DNA mismatch repair homolog of	1.676	3.92E-09
At3g24500	MBF1C (multiprotein bridging factor 1C); One of three genes in A. thaliana encoding multiprotein bridging fa	5.966	4.16E-12
At3g24520	HSFC1 (heat shock transcription factor C1); member of Heat Stress Transcription Factor (Hsf) family	-1.305	5.71E-07
At3g24715	Protein kinase superfamily protein with octicosapeptide/Phox/Bem1p domain	-1.333	2.91E-07
At3g24982	ATRLP40 (receptor like protein 40)	-2.059	0.01649
At3g25010	AtRLP41 (receptor like protein 41)	-1.969	0.00371
At3g25013	Synaptobrevin family protein	1.088	1.46E-06
At3g25070	RIN4 (RPM1 interacting protein 4); Encodes a member of the R protein complex and may represent a virulenc	1.12	1.06E-07
At3g25110	AtFaTA (fatA acyl-ACP thioesterase); Encodes a FatA acyl-ACP thioesterase	1.984	1.29E-08
At3g25130	unknown protein	-1.557	3.68E-07
At3g25180	CYP82G1 (cytochrome P450, family 82, subfamily G, polypeptide 1); member of CYP82G	-1.789	0.00295
At3g25230	FKBP62 (FK506 BINDING PROTEIN 62); Encodes a a high molecular weight member of the FK506 binding	3.28	2.90E-11
At3g25250	OXI1 (oxidative signal-inducible1); Arabidopsis protein kinase	4.596	5.17E-11
At3g25560	NIK2 (NSP-interacting kinase 2)	-1.124	8.17E-08
At3g25570	Adenosylmethionine decarboxylase family protein	-1.387	2.66E-06
At3g25610	ATPase E1-E2 type family protein / haloacid dehalogenase-like hydrolase family protein	2.006	2.09E-07
At3g25690	CHUP1 (CHLOROPLAST UNUSUAL POSITIONING 1); actin binding protein required for normal chloropla	-1.425	3.80E-07
At3g25710	BHLH32 (basic helix-loop-helix 32); Encodes a basic helixûloopûhelix transcription factor that is expressed i	-1.24	2.39E-06
At3g25717	DVL6 (DEVIL 6)	-1.746	7.45E-09
At3g25760	AOC1 (allene oxide cyclase 1); encodes allene oxide cyclase. One of four genes in Arabidopsis that encode th	-1.335	0.0005
At3g25790	myb-like transcription factor family protein	-2.76	3.55E-05
At3g25990	Homeodomain-like superfamily protein	1.834	2.83E-10
At3g26020	Protein phosphatase 2A regulatory B subunit family protein; Encodes protein phosphatase 2A (PP2A) B'eta sul	1.588	6.17E-09
At3g26040	HXXXD-type acyl-transferase family protein	2.753	3.22E-10
At3g26050	TPX2 (targeting protein for Xklp2) protein family	1.02	2.60E-06
At3g26165	CYP71B18 (cytochrome P450, family 71, subfamily B, polypeptide 18); putative cytochrome P450.	-1.3	4.75E-06
At3g26190	CYP71B21 (cytochrome P450, family 71, subfamily B, polypeptide 21); putative cytochrome P450	1.681	3.64E-08
At3g26235	unknown protein	-1.036	3.01E-05
At3g26280	CYP71B4 (cytochrome P450, family 71, subfamily B, polypeptide 4); cytochrome P450 monooxygenase	-2.484	9.66E-08
At3g26295	CYP71B33 (cytochrome P450, family 71, subfamily B, polypeptide 33); putative cytochrome P450.	2.961	2.94E-06
At3g26500	PIRL2 (plant intracellular ras group-related LRR 2); Encodes PIRL2, a member of the Plant Intracellular Ras-g	2.018	3.13E-09
At3g26510	Octicosapeptide/Phox/Bem1p family protein	1.116	0.0245
At3g26520	SITIP (SALT-STRESS INDUCIBLE TONOPLAST INTRINSIC PROTEIN); gamma tonoplast intrinsic protei	-1.18	0.0003
At3g26580	Tetratricopeptide repeat (TPR)-like superfamily protein	-1.129	1.13E-06
At3g26610	Pectin lyase-like superfamily protein	1.005	2.66E-05
At3g26620	LBD23 (LOB domain-containing protein 23)	3.056	3.22E-11
At3g26680	ATSNM1 (SENSITIVE TO NITROGEN MUSTARD 1); involved in a SNM-dependent recombinational repa	1.109	5.19E-07
At3g26690	ATNUDT13 (ARABIDOPSIS THALIANA NUDIX HYDROLASE HOMOLOG 13); Encodes AtNUDT13, a	1.229	1.00E-07
At3g26740	CCL (CCR-like); transcripts are differentially regulated at the level of mRNA stability at different times of da	-2.466	7.44E-08
At3g26760	NAD(P)-binding Rossmann-fold superfamily protein	-1.371	1.50E-05
At3g26830	PAD3 (PHYTOALEXIN DEFICIENT 3); Mutations in pad3 are defective in biosynthesis of the indole derivec	2.027	0.00986
At3g26910	hydroxyproline-rich glycoprotein family protein	2.89	5.58E-09

At3g26980	MUB4 (membrane-anchored ubiquitin-fold protein 4 precursor)	2.248	4.20E-11
At3g27025	unknown protein	1.505	6.18E-06
At3g27030	unknown protein	-1.253	1.08E-07
At3g27070	TOM20-1 (translocase outer membrane 20-1); Form of TOM20, which is a component of the TOM complex, i	1.375	2.21E-07
At3g27210	unknown protein	-1.657	1.23E-07
At3g27360	Histone superfamily protein	-1.062	2.68E-05
At3g27400	Pectin lyase-like superfamily protein	-1.107	5.57E-05
At3g27440	UKL5 (uridine kinase-like 5); One of the homologous genes predicted to encode proteins with UPRT domains	1.622	1.81E-06
At3g27500	Cysteine/Histidine-rich C1 domain family protein	-1.701	6.67E-07
At3g27540	beta-1,4-N-acetylglucosaminyltransferase family protein	-1.182	1.48E-05
At3g27550	RNA-binding CRS1 / YhbY (CRM) domain protein	1.478	1.62E-07
At3g27570	Sucrase/ferredoxin-like family protein	1.082	4.01E-07
At3g27600	SWAP (Suppressor-of-White-APricot)/surp RNA-binding domain-containing protein	1.73	2.30E-07
At3g27920	ATMYB0 (myb domain protein 0); Encodes a Myb-like protein that is required for induction of trichome deve	1.084	0.00056
At3g27930	unknown protein	1.741	1.89E-08
At3g27960	Tetratricopeptide repeat (TPR)-like superfamily protein	-1.06	9.86E-07
At3g27997	expressed protein; pseudogene of unknown protein	1.637	4.74E-06
At3g28060	Nodulin MtN21 /EamA-like transporter family protein	-1.087	8.55E-06
At3g28070	nodulin MtN21 /EamA-like transporter family protein	-1.342	7.59E-06
At3g28130	nodulin MtN21 /EamA-like transporter family protein	-1.2	2.48E-07
At3g28180	ATCSLC04 (Cellulose-synthase-like C4); encodes a gene similar to cellulose synthase	-1.376	1.75E-06
At3g28210	SAP12 (STRESS-ASSOCIATED PROTEIN 12); Encodes a putative zinc finger protein (PMZ).	5.755	2.79E-09
At3g28220	TRAF-like family protein	1.217	0.00474
At3g28270	Protein of unknown function (DUF677)	1.841	7.84E-05
At3g28290	AT14A (Protein of unknown function (DUF677)); Encodes a protein with sequence similarity to integrins. Loc	-1.477	0.00017
At3g28310	Protein of unknown function (DUF677)	-1.016	5.75E-07
At3g28340	GATL10 (galacturonosyltransferase-like 10); Encodes a protein with putative galacturonosyltransferase activi	1.237	0.0042
At3g28350	similar to unknown protein [Arabidopsis thaliana] (TAIR:AT3G28370.1); similar to hypothetical protein OsI_(	1.162	5.56E-06
At3g28420	Putative membrane lipoprotein	-1.091	2.39E-05
At3g28510	P-loop containing nucleoside triphosphate hydrolases superfamily protein	-3.344	0.00036
At3g28540	P-loop containing nucleoside triphosphate hydrolases superfamily protein	-2.162	0.0002
At3g28740	CYP81D1 (Cytochrome P450 superfamily protein); Encodes a member of the cytochrome p450 family. Expres	3.525	1.85E-09
At3g28850	Glutaredoxin family protein	1.137	1.15E-07
At3g28860	ABCB19 (ATP binding cassette subfamily B19); Belongs to the family of ATP-binding cassette (ABC) transpo	-1.068	1.50E-06
At3g28910	MYB30 (myb domain protein 30); transcription factor myb homologue	-1.602	8.80E-08
At3g28920	AtHB34 (homeobox protein 34)	-1.198	9.56E-08
At3g28930	AIG2 (AVRRPT2-INDUCED GENE 2); avrRpt2-induced gene that exhibits RPS2- and avrRpt2-dependent ind	1.157	4.26E-06
At3g28960	Transmembrane amino acid transporter family protein	-2.505	5.46E-10
At3g29000	Calcium-binding EF-hand family protein	2.064	5.06E-08
At3g29030	ATEXP5 (ARABIDOPSIS THALIANA EXPANSIN 5); Encodes an expansin. Naming convention from the Ex	-1.109	0.0003
At3g29035	ANAC059 (Arabidopsis NAC domain containing protein 59); Encodes a protein with transcription factor activ	-1.129	0.0001
At3g29250	NAD(P)-binding Rossmann-fold superfamily protein	-2.039	0.02221
At3g29252	pseudogene of short-chain dehydrogenase/reductase (SDR) family; pseudogene of short-chain dehydrogenase/r	-1.222	0.00897
At3g29320	Glycosyl transferase, family 35; Encodes a plastidic alpha-glucan phosphorylase. In vitro, the enzyme has a pr	-1.308	5.06E-07
At3g29370	unknown protein	-1.884	8.54E-06
At3g29810	COBL2 (COBRA-like protein 2 precursor)	1.665	2.42E-08
At3g29970	B12D protein	1.518	0.01183
At3g30122	expressed protein; pseudogene similar to peptide chain release factor protein	1.099	0.0065
At3g30720	QQS (QUA-QUINE STARCH)	-1.084	0.00221
At3g30725	AtGDU6 (glutamine dumper 6); Encodes a member of the GDU (glutamine dumper) family proteins involved	-1.382	0.00181
At3g32966	pseudogene, hypothetical protein (transposable element gene)	1.819	2.78E-08
At3g32990	pseudogene, ATP synthase C subunit, blastp match of 86% identity and 9.3e-27 P-value to SP|P06286|ATPH_'	-1.19	0.00073
At3g42130	glycine-rich protein	-1.255	0.0001
At3g42800	unknown protein	-2.65	5.39E-08
At3g42850	Mevalonate/galactokinase family protein	-1.55	0.00116
At3g43110	unknown protein	-2.575	0.00044
At3g43160	MEE38 (maternal effect embryo arrest 38)	3.902	0.0218
At3g43250	Family of unknown function (DUF572)	2.13	3.14E-06
At3g43430	RING/U-box superfamily protein	-1.5	1.00E-07
At3g43670	Copper amine oxidase family protein	-2.375	2.17E-09
At3g43720	Bifunctional inhibitor/lipid-transfer protein/seed storage 2S albumin superfamily protein	-1.179	3.06E-06
At3g43790	ZIFL2 (zinc induced facilitator-like 2)	-1.255	7.37E-07
At3g44110	ATJ3 (DNAJ homologue 3); homologous to the co-chaperon DNAJ protein from E coli	2.04	2.88E-09
At3g44120	F-box and associated interaction domains-containing protein	-1.083	2.66E-06
At3g44190	FAD/NAD(P)-binding oxidoreductase family protein	2.558	1.07E-09
At3g44260	Polynucleotidyl transferase, ribonuclease H-like superfamily protein	-1.769	3.92E-07
At3g44300	AtNIT2 (nitrilase 2); Encodes an enzyme that catalyzes the hydrolysis of indole-3-acetonitrile (IAN) to indole-	1.062	0.0272

At3g44320	AtNIT3 (NITRILASE 3); This enzyme catalyzes the hydrolysis of indole-3-acetonitrile (IAN) to indole-3-aceti	1.149	3.52E-07
At3g44400	Disease resistance protein (TIR-NBS-LRR class) family	1.12	3.33E-05
At3g44420	pseudogene, hypothetical protein (transposable element gene)	-1.207	2.81E-08
At3g44470	transposable element gene	2.106	1.76E-08
At3g44510	alpha/beta-Hydrolases superfamily protein	-1.618	0.0004
At3g44630	Disease resistance protein (TIR-NBS-LRR class) family	1.653	8.28E-07
At3g44716	unknown protein	1.27	1.80E-05
At3g44820	Phototropic-responsive NPH3 family protein	-1.258	1.26E-06
At3g44830	Lecithin:cholesterol acyltransferase family protein	2.036	3.72E-05
At3g45040	phosphatidate cytidylyltransferase family protein; Encodes a putative dolichol kinase.	1.424	1.42E-08
At3g45160	Putative membrane lipoprotein	-1.181	9.03E-06
At3g45230	hydroxyproline-rich glycoprotein family protein	-1.001	5.71E-07
At3g45260	C2H2-like zinc finger protein	-1.307	1.08E-07
At3g45640	ATMPK3 (mitogen-activated protein kinase 3); Encodes a mitogen-activated kinase whose mRNA levels incre	1.191	0.00017
At3g45680	Major facilitator superfamily protein	1.082	3.41E-06
At3g45780	NPH1 (NONPHOTOTROPIC HYPOCOTYL 1); Blue-light photoreceptor. Contains a light activated serine-thr	-1.344	2.90E-05
At3g45850	P-loop containing nucleoside triphosphate hydrolases superfamily protein	-1.025	7.60E-08
At3g45860	CRK4 (cysteine-rich RLK (RECEPTOR-like protein kinase) 4); Encodes a cysteine-rich receptor-like protein k	-2.203	0.0002
At3g45930	Histone superfamily protein	-1.073	3.23E-05
At3g45940	Glycosyl hydrolases family 31 protein	-1.61	4.85E-06
At3g45960	ATEXLA3 (expansin-like A3); member of EXPANSIN-LIKE. Naming convention from the Expansin Working	-1.432	0.0002
At3g45970	ATEXLA1 (expansin-like A1); member of EXPANSIN-LIKE. Naming convention from the Expansin Working	-1.853	6.48E-05
At3g46020	RNA-binding (RRM/RBD/RNP motifs) family protein	1.034	0.0017
At3g46070	C2H2-type zinc finger family protein	3.415	8.79E-09
At3g46080	C2H2-type zinc finger family protein	3.049	0.00047
At3g46090	ZAT7 (C2H2 and C2HC zinc fingers superfamily protein)	3.989	1.14E-05
At3g46110	LOCATED IN: plasma membrane (Domain of unknown function (DUF966))	2.002	2.21E-07
At3g46120	ATPAP19 (PURPLE ACID PHOSPHATASE 19)	-1.622	0.00106
At3g46200	aTNUDT9 (nudix hydrolase homolog 9)	1.928	2.42E-10
At3g46230	ATHSP17.4 (ARABIDOPSIS THALIANA HEAT SHOCK PROTEIN 17.4); member of the class I small heat-	9.907	4.18E-12
At3g46280	protein kinase-related	-1.675	0.00437
At3g46300	unknown protein	-1.086	0.00211
At3g46320	Histone superfamily protein	-1.306	3.89E-06
At3g46640	LUX (LUX ARRHYTHMO); Encodes a myb family transcription factor with a single Myb DNA-binding doma	-1.434	2.76E-07
At3g46658	other RNA; Potential natural antisense gene, locus overlaps with AT3G46660	-1.106	3.19E-06
At3g46730	NB-ARC domain-containing disease resistance protein	1.406	2.49E-06
At3g46750	unknown protein	1.227	1.12E-05
At3g46870	Pentatricopeptide repeat (PPR) superfamily protein	2.061	2.58E-09
At3g46880	unknown protein	-1.39	4.40E-05
At3g46930	Protein kinase superfamily protein	2.077	6.77E-09
At3g46970	ATPHS2 (ALPHA-GLUCAN PHOSPHORYLASE 2); Encodes a cytosolic alpha-glucan phosphorylase. In vitr	-1.663	4.89E-07
At3g47380	Plant invertase/pectin methylesterase inhibitor superfamily protein	-1.04	3.55E-06
At3g47480	Calcium-binding EF-hand family protein	-2.091	0.00431
At3g47510	unknown protein	-1.316	8.80E-06
At3g47550	RING/FYVE/PHD zinc finger superfamily protein	2.14	3.98E-10
At3g47570	Leucine-rich repeat protein kinase family protein	-1.008	1.01E-06
At3g47600	ATMYB94 (myb domain protein 94); Encodes a putative transcription factor (MYB94).	-1.16	1.39E-06
At3g47730	ABCA2 (ATP-binding cassette A2); member of ATH subfamily	1.16	5.08E-07
At3g47780	ATATH6 (A. THALIANA ABC2 HOMOLOG 6); member of ATH subfamily	1.63	7.33E-06
At3g47800	Galactose mutarotase-like superfamily protein	-1.342	1.06E-05
At3g47820	PUB39 (PLANT U-BOX 39)	1.418	2.98E-07
At3g47850	unknown protein	1.115	5.71E-07
At3g47980	Integral membrane HPP family protein	-1.359	2.77E-06
At3g48070	RING/U-box superfamily protein	2.17	1.39E-09
At3g48080	alpha/beta-Hydrolases superfamily protein	-1.704	0.00183
At3g48100	ARR5 (response regulator 5); Encodes a transcription repressor that mediates a negative feedback loop in cyto	-1.033	1.64E-05
At3g48240	Octicosapeptide/Phox/Bem1p family protein	3.752	8.37E-12
At3g48260	WNK3 (with no lysine (K) kinase 3); Encodes a member of the WNK family (9 members in all) of protein kina	-1.826	1.52E-08
At3g48360	BT2 (BTB and TAZ domain protein 2); encodes a protein (BT2) that is an essential component of the TAC1-m	-3.762	1.07E-08
At3g48410	alpha/beta-Hydrolases superfamily protein	-1.05	2.11E-06
At3g48490	unknown protein	-1.171	1.06E-05
At3g48640	unknown protein	3.071	0.0006
At3g48650	pseudogene, At14a-related protein, similar to At14a (GI:11994571 and GI:11994573) (Arabidopsis thaliana)	3.733	1.70E-06
At3g48720	HXXXD-type acyl-transferase family protein	-2.366	1.29E-06
At3g48740	Nodulin MtN3 family protein	-1.854	3.82E-06
At3g48790	Pyridoxal phosphate (PLP)-dependent transferases superfamily protein	3.555	3.07E-09
At3g48840	RNA-binding (RRM/RBD/RNP motifs) family protein	1.162	5.22E-07

At3g48970	Heavy metal transport/detoxification superfamily protein	-1.923	1.60E-08
At3g49055	unknown protein	2.366	7.95E-10
At3g49160	pyruvate kinase family protein; Expression of the gene is downregulated in the presence of paraquat, an induce	1.892	2.79E-08
At3g49260	iqd21 (IQ-domain 21)	-1.023	1.75E-05
At3g49380	iqd15 (IQ-domain 15)	4.054	8.80E-11
At3g49530	ANAC062 (NAC domain containing protein 62); Transcription factor that serves as a molecular link between c	1.658	1.39E-08
At3g49540	unknown protein	4.007	6.08E-10
At3g49570	LSU3 (RESPONSE TO LOW SULFUR 3)	4.483	7.67E-10
At3g49580	LSU1 (RESPONSE TO LOW SULFUR 1)	3.159	1.38E-07
At3g49630	2-oxoglutarate (2OG) and Fe(II)-dependent oxygenase superfamily protein	-2.589	5.26E-05
At3g49670	BAM2 (BARELY ANY MERISTEM 2); Encodes a CLAVATA1-related receptor kinase-like protein required	-1.071	1.42E-05
At3g49744	unknown protein	1.318	5.56E-07
At3g49860	ARLA1B (ADP-ribosylation factor-like A1B); A member of ARF-like GTPase family. A thaliana has 21 meml	1.738	4.89E-07
At3g49870	ARLA1C (ADP-ribosylation factor-like A1C); A member of ARF-like GTPase family. A thaliana has 21 meml	1.073	4.09E-06
At3g49940	LBD38 (LOB domain-containing protein 38)	-1.397	4.79E-07
At3g49970	Phototropic-responsive NPH3 family protein	-1.633	1.75E-07
At3g50010	Cysteine/Histidine-rich C1 domain family protein	-1.488	0.00019
At3g50120	Plant protein of unknown function (DUF247)	-2.637	2.02E-10
At3g50140	Plant protein of unknown function (DUF247)	-1.867	3.17E-09
At3g50260	CEJ1 (cooperatively regulated by ethylene and jasmonate 1); Encodes a member of the DREB subfamily A-5 c	1.608	5.77E-06
At3g50270	HXXXD-type acyl-transferase family protein	-1.611	6.41E-09
At3g50310	MAPKKK20 (mitogen-activated protein kinase kinase kinase 20); member of MEKK subfamily	-1.669	7.34E-08
At3g50340	unknown protein	-1.137	3.23E-07
At3g50450	HR1 (homolog of RPW8 1); Homolog of RPW8	-1.581	3.39E-07
At3g50480	HR4 (homolog of RPW8 4); Homolog of RPW8	1.101	0.0021
At3g50570	hydroxyproline-rich glycoprotein family protein	-1.426	3.01E-07
At3g50700	AtIDD2 (indeterminate(ID)-domain 2); zinc finger protein, similar to maize Indeterminate1 (ID1)	-1.35	1.68E-06
At3g50740	UGT72E1 (UDP-glucosyl transferase 72E1); UGT72E1 is an UDPG:coniferyl alcohol glucosyltransferase whic	-1.752	1.13E-06
At3g50750	BEH1 (BES1/BZR1 homolog 1)	-1.082	3.18E-07
At3g50770	CML41 (calmodulin-like 41)	-1.531	0.00724
At3g50840	Phototropic-responsive NPH3 family protein	1.538	4.64E-07
At3g50850	Putative methyltransferase family protein	1.6	3.50E-07
At3g50910	unknown protein	2.692	5.24E-10
At3g50930	BCS1 (cytochrome BC1 synthesis)	1.011	0.0027
At3g50940	P-loop containing nucleoside triphosphate hydrolases superfamily protein	1.712	4.03E-07
At3g50950	ZAR1 (HOPZ-ACTIVATED RESISTANCE 1)	1.008	0.0005
At3g51210	P-loop containing nucleoside triphosphate hydrolases superfamily protein	-1.098	0.00072
At3g51240	F3H (flavanone 3-hydroxylase); Encodes flavanone 3-hydroxylase that is coordinately expressed with chalcone	1.313	0.0008
At3g51400	Arabidopsis protein of unknown function (DUF241)	-1.764	7.94E-09
At3g51470	Protein phosphatase 2C family protein	-1.073	1.53E-05
At3g51520	diacylglycerol acyltransferase family	1.097	2.13E-07
At3g51600	LTP5 (lipid transfer protein 5); Predicted to encode a PR (pathogenesis-related) protein. Belongs to the lipid tı	-1.33	1.49E-06
At3g51660	Tautomerase/MIF superfamily protein	-1.062	3.79E-05
At3g51710	D-mannose binding lectin protein with Apple-like carbohydrate-binding domain	-1.434	1.10E-08
At3g51790	ATG1 (transmembrane protein G1P-related 1); putative transmembrane protein G1p (AtG1) mRNA, complete	1.012	2.48E-06
At3g51860	CAX3 (cation exchanger 3)	-1.025	0.04178
At3g51890	Clathrin light chain protein	1.967	1.25E-08
At3g51910	AT-HSFA7A (ARABIDOPSIS THALIANA HEAT SHOCK TRANSCRIPTION FACTOR A7A); member of	2.992	4.62E-11
At3g51920	CAM9 (calmodulin 9); encodes a divergent member of calmodulin, which is an EF-hand family of Ca2+-bindi	1.562	1.40E-07
At3g52310	ABC-2 type transporter family protein	3.226	1.40E-09
At3g52360	unknown protein	2.166	5.94E-10
At3g52400	SYP122 (syntaxin of plants 122); syntaxin protein, involved in the negative regulation of defense pathways suc	3.849	7.97E-10
At3g52430	ATPAD4 (ARABIDOPSIS PHYTOALEXIN DEFICIENT 4); Encodes a lipase-like gene that is important for s	1.102	0.0269
At3g52450	PUB22 (plant U-box 22); Encodes a cytoplasmically localized U-box domain E3 ubiquitin ligase protein that is	1.308	2.61E-05
At3g52720	ACA1 (alpha carbonic anhydrase 1); Encodes a putative alpha carbonic anhydrase (CAH1) located in the chlor	-1.721	2.57E-05
At3g52800	A20/AN1-like zinc finger family protein	1.605	9.58E-07
At3g52840	BGAL2 (beta-galactosidase 2)	-1.459	1.57E-07
At3g53000	AtPP2-A15 (phloem protein 2-A15)	1.394	2.42E-08
At3g53200	AtMYB27 (myb domain protein 27); Member of the R2R3 factor gene family.	1.087	9.74E-07
At3g53230	ATPase, AAA-type, CDC48 protein	3.82	7.31E-12
At3g53250	SAUR-like auxin-responsive protein family	-2.558	8.51E-08
At3g53280	CYP71B5 (cytochrome p450 71b5); cytochrome P450 monooxygenase	1.579	7.23E-08
At3g53290	CYP71B30P (cytochrome P450, family 71, subfamily B, polypeptide 30 pseudogene); missing N-term 80 AA	1.399	2.13E-08
At3g53365	Unknown gene; Unknown gene	-1.077	4.40E-05
At3g53668	CPuORF51 (conserved peptide upstream open reading frame 51); Upstream open reading frames (uORFs) are	1.003	7.00E-05
At3g53720	ATCHX20 (cation/H+ exchanger 20); member of Putative Na+/H+ antiporter family. Involved in the osmoreg	-1.711	2.98E-07
At3g53800	Fes1B (Fes1B); Encodes one of the Arabidopsis orthologs of the human Hsp70-binding protein 1 (HspBP-1) aı	-1.832	8.59E-08

At3g53810	Concanavalin A-like lectin protein kinase family protein	1.643	6.06E-08
At3g53830	Regulator of chromosome condensation (RCC1) family protein	4.108	8.39E-12
At3g53850	Uncharacterised protein family (UPF0497)	-1.016	4.14E-05
At3g54030	Protein kinase protein with tetratricopeptide repeat domain	-1.034	1.24E-07
At3g54100	O-fucosyltransferase family protein	1.046	2.62E-07
At3g54130	Josephin family protein	2.143	6.66E-09
At3g54150	S-adenosyl-L-methionine-dependent methyltransferases superfamily protein	2.531	9.64E-05
At3g54160	RNI-like superfamily protein	1.605	6.99E-08
At3g54260	TBL36 (TRICHOME BIREFRINGENCE-LIKE 36); Encodes a member of the TBL (TRICHOME BIREFRIN(	-1.204	3.04E-05
At3g54320	ASML1 (ACTIVATOR OF SPO(MIN)::LUC1); WRINKLED1 encodes transcription factor of the AP2/ERWE	1.57	2.88E-07
At3g54340	AP3 (APETALA 3); Floral homeotic gene encoding a MADS domain protein homologous to SRF transcriptior	-2.063	0.00032
At3g54420	ATCHITIV (CHITINASE CLASS IV); encodes an EP3 chitinase that is expressed during somatic embryogene	1.686	1.65E-08
At3g54500	BEST Arabidopsis thaliana protein match is: dentin sialophosphoprotein-related (TAIR:AT5G64170.1)	2.596	1.63E-08
At3g54600	Class I glutamine amidotransferase-like superfamily protein	-2.288	1.14E-07
At3g54620	ATBZIP25 (ARABIDOPSIS THALIANA BASIC LEUCINE ZIPPER 25); bZIP transcription factor-like protei	1.344	7.15E-09
At3g54700	PHT1.7 (phosphate transporter 1.7); Encodes Pht1;7, a member of the Pht1 family of phosphate transporters w	-1.18	0.02308
At3g54780	Zinc finger (C3HC4-type RING finger) family protein	-1.431	3.96E-08
At3g54830	Transmembrane amino acid transporter family protein	-1.574	2.34E-05
At3g55240	Plant protein 1589 of unknown function; Overexpression leads to PEL (Pseudo-Etiolation in Light) phenotype.	-1.997	5.51E-06
At3g55420	unknown protein	-1.05	5.10E-05
At3g55470	Calcium-dependent lipid-binding (CaLB domain) family protein	1.906	3.21E-10
At3g55550	Concanavalin A-like lectin protein kinase family protein	1.041	0.00043
At3g55580	Regulator of chromosome condensation (RCC1) family protein	2	1.64E-08
At3g55700	UDP-Glycosyltransferase superfamily protein	1.591	4.70E-07
At3g55760	unknown protein	-1.15	1.96E-07
At3g55840	Hs1pro-1 protein	2.319	3.12E-07
At3g56040	UGP3 (UDP-glucose pyrophosphorylase 3)	-1.676	3.10E-06
At3g56200	Transmembrane amino acid transporter family protein; Encodes a putative amino acid transporter.	2.556	1.87E-09
At3g56250	unknown protein	3.028	7.34E-11
At3g56275	expressed protein; pseudogene of unknown protein	2.185	4.50E-05
At3g56290	unknown protein	3.246	2.17E-09
At3g56380	ARR17 (response regulator 17); response regulator 17	-1.649	1.98E-06
At3g56500	serine-rich protein-related	2.002	5.99E-10
At3g56600	Protein kinase superfamily protein	2.223	0.0191
At3g56710	SIB1 (sigma factor binding protein 1); Sig1 binding protein; interacts with Sig1R4. As well as Sig1, SibI is im	2.941	1.94E-08
At3g56730	Putative endonuclease or glycosyl hydrolase	2.101	2.65E-10
At3g56740	Ubiquitin-associated (UBA) protein	1.114	2.53E-06
At3g56970	ORG2 (OBP3-RESPONSIVE GENE 3); Encodes a member of the basic helix-loop-helix transcription factor fa	1.21	0.02598
At3g56980	ORG3 (OBP3-RESPONSIVE GENE 3); Encodes a member of the basic helix-loop-helix transcription factor p	1.591	0.02086
At3g57010	Calcium-dependent phosphotriesterase superfamily protein	1.369	7.45E-07
At3g57157	other RNA	-2.878	1.89E-05
At3g57280	Transmembrane proteins 14C	1.012	8.01E-06
At3g57370	Cyclin family protein	1.715	1.26E-07
At3g57380	Glycosyltransferase family 61 protein	2.878	9.99E-10
At3g57430	OTP84 (ORGANELLE TRANSCRIPT PROCESSING 84); Encodes a chloroplast RNA editing factor.	-1.121	1.23E-06
At3g57450	unknown protein	1.574	4.43E-07
At3g57480	zinc finger (C2H2 type, AN1-like) family protein	1.407	2.31E-08
At3g57530	CPK32 (calcium-dependent protein kinase 32); Calcium-dependent Protein Kinase. ABA signaling component	2.917	3.89E-10
At3g57550	AGK2 (guanylate kinase); guanylate kinase	1.437	5.05E-05
At3g57640	Protein kinase superfamily protein	1.549	2.25E-07
At3g57700	Protein kinase superfamily protein	1.05	0.0002
At3g57730	Protein kinase superfamily protein	1.331	1.41E-06
At3g57740	Protein kinase superfamily protein	2.139	3.17E-09
At3g57760	Protein kinase superfamily protein	1.035	3.93E-06
At3g57790	Pectin lyase-like superfamily protein	-1.204	3.82E-07
At3g57810	Cysteine proteinases superfamily protein	1.903	1.83E-09
At3g57880	Calcium-dependent lipid-binding (CaLB domain) plant phosphoribosyltransferase family protein	2.422	1.15E-09
At3g58000	VQ motif-containing protein	1.25	1.49E-06
At3g58070	GIS (GLABROUS INFLORESCENCE STEMS); Putative transcription factor, contains C2H2 domain, regulat	-1.101	1.72E-07
At3g58490	Phosphatidic acid phosphatase (PAP2) family protein	2.387	1.10E-09
At3g58600	Adaptin ear-binding coat-associated protein 1 NECAP-1	1.064	2.53E-06
At3g58850	PAR2 (PHY RAPIDLY REGULATED 2); Encodes PHYTOCHROME RAPIDLY REGULATED2 (PAR2), ar	-1.808	1.89E-07
At3g58860	F-box/RNI-like superfamily protein	-1.039	4.39E-05
At3g58990	IPMI1 (isopropylmalate isomerase 1)	-4.127	8.42E-11
At3g59150	F-box/RNI-like superfamily protein	2.149	5.53E-10
At3g59250	F-box/RNI-like superfamily protein	-1.562	8.70E-07
At3g59270	FBD-like domain family protein	3.355	3.22E-10

At3g59280	TXR1 (THAXTOMIN A RESISTANT 1); mutant exhibited resistance to growth on media containing thaxtom	1.939	2.43E-10
At3g59290	ENTH/VHS family protein	1.173	6.33E-06
At3g59430	unknown protein	2.451	0.0104
At3g59520	ATRBL13 (RHOMBOID-like protein 13)	2.896	2.24E-10
At3g59530	LAP3 (LESS ADHERENT POLLEN 3)	4.494	2.61E-12
At3g59580	Plant regulator RWP-RK family protein	-1.298	2.81E-06
At3g59710	NAD(P)-binding Rossmann-fold superfamily protein	-1	1.30E-05
At3g59820	LETM1-like protein	-1.034	1.96E-05
At3g59880	unknown protein	2.208	7.46E-08
At3g59900	ARGOS (AUXIN-REGULATED GENE INVOLVED IN ORGAN SIZE); Encodes ARGOS (Auxin-Regulated	-1.577	4.74E-08
At3g59940	Galactose oxidase/kelch repeat superfamily protein	-1.421	6.71E-07
At3g60010	ASK13 (SKP1-like 13)	1.542	1.39E-05
At3g60140	BGLU30 (BETA GLUCOSIDASE 30); Encodes a protein similar to beta-glucosidase and is a member of glyc	4.68	1.07E-05
At3g60176	other RNA	1.368	1.74E-05
At3g60250	CKB3 (casein kinase II beta chain 3); Regulatory (beta) subunit of the protein kinase CK2. Involved in regula	1.03	1.14E-06
At3g60290	2-oxoglutarate (2OG) and Fe(II)-dependent oxygenase superfamily protein	-2.262	2.60E-07
At3g60530	GATA4 (GATA transcription factor 4); Encodes a member of the GATA factor family of zinc finger transcript	-1.129	8.08E-06
At3g60630	ATHAM2 (ARABIDOPSIS THALIANA HAIRY MERISTEM 2)	-1.047	5.65E-07
At3g60650	unknown protein	-1.266	0.0007
At3g60700	Protein of unknown function (DUF1163)	-1.171	0.00037
At3g61060	AtPP2-A13 (phloem protein 2-A13)	-1.133	0.0002
At3g61090	Putative endonuclease or glycosyl hydrolase	1.482	1.16E-09
At3g61111	Zinc-binding ribosomal protein family protein	4.063	1.09E-08
At3g61190	BAP1 (BON association protein 1); Encodes a protein with a C2 domain that binds to BON1 in yeast two hybr	1.89	0.00047
At3g61210	S-adenosyl-L-methionine-dependent methyltransferases superfamily protein	-1.327	2.22E-07
At3g61220	NAD(P)-binding Rossmann-fold superfamily protein	1.313	2.77E-07
At3g61280	Arabidopsis thaliana protein of unknown function (DUF821)	-1.01	0.0001
At3g61320	Bestrophin-like protein	1.778	1.90E-07
At3g61410	BEST Arabidopsis thaliana protein match is: U-box domain-containing protein kinase family protein (TAIR:A	-1.432	0.00119
At3g61520	Pentatricopeptide repeat (PPR) superfamily protein	-1.127	6.03E-07
At3g61550	RING/U-box superfamily protein	-1.021	2.75E-07
At3g61630	CRF6 (cytokinin response factor 6); CRF6 encodes one of the six cytokinin response factors. CRF5 belongs to	2.107	1.57E-08
At3g61678	unknown protein	-1.197	8.98E-06
At3g61760	ADL1B (DYNAMIN-like 1B)	1.741	8.93E-09
At3g61880	CYP78A9 (cytochrome p450 78a9); Encodes a cytochrome p450 monooxygenase. Overexpression of this gen	-1.123	1.74E-06
At3g61920	unknown protein	-1.344	1.49E-08
At3g61930	unknown protein	-1.247	0.0031
At3g62040	Haloacid dehalogenase-like hydrolase (HAD) superfamily protein	-1.344	7.19E-06
At3g62090	PIF6 (PHYTOCHROME-INTERACTING FACTOR 6); encodes a novel Myc-related bHLH transcription facto	-1.147	1.13E-06
At3g62110	Pectin lyase-like superfamily protein	-1.167	1.46E-06
At3g62150	PGP21 (P-glycoprotein 21)	1.041	1.35E-06
At3g62260	Protein phosphatase 2C family protein	5.3	1.96E-12
At3g62320	Polynucleotidyl transferase, ribonuclease H-like superfamily protein	1.065	6.27E-05
At3g62340	WRKY68 (WRKY family transcription factor); member of WRKY Transcription Factor; Group II-c	2.352	2.83E-06
At3g62350	BEST Arabidopsis thaliana protein match is: F-box family protein (TAIR:AT1G71320.1)	1.506	1.48E-06
At3g62510	protein disulfide isomerase-related	-1.115	9.27E-06
At3g62550	Adenine nucleotide alpha hydrolases-like superfamily protein	-2.586	3.27E-08
At3g62630	Protein of unknown function (DUF1645)	-1.302	2.90E-07
At3g62660	GATL7 (galacturonosyltransferase-like 7); Encodes a protein with putative galacturonosyltransferase activity.	-1.583	1.34E-07
At3g62690	ATL5 (AtL5); Encodes a RING-H2 zinc finger protein related to ATL2. The ATL gene family is represented b	-1.112	6.69E-07
At3g62750	BGLU8 (beta glucosidase 8)	-1.06	9.86E-06
At3g62760	ATGSTF13 (Glutathione S-transferase family protein); Encodes glutathione transferase belonging to the phi cl	2.175	3.26E-08
At3g62770	AtATG18a (Transducin/WD40 repeat-like superfamily protein); Required for autophagosome formation during	2.038	1.73E-08
At3g62780	Calcium-dependent lipid-binding (CaLB domain) family protein	3.504	4.90E-10
At3g62820	Plant invertase/pectin methylesterase inhibitor superfamily protein	-1.723	5.50E-07
At3g62860	alpha/beta-Hydrolases superfamily protein	-1.239	1.74E-06
At3g62930	Thioredoxin superfamily protein	-1.943	1.35E-07
At3g62950	Thioredoxin superfamily protein	-2.81	3.06E-06
At3g63110	ATIPT3 (isopentenyltransferase 3); Encodes cytokinin synthase involved in cytokinin biosynthesis. IPT3 subc	-1.439	4.64E-06
At3g63160	FUNCTIONS IN: molecular_function unknown; INVOLVED IN: biological_process unknown; LOCATED IN	-2.007	5.43E-08
At3g63210	MARD1 (MEDIATOR OF ABA-REGULATED DORMANCY 1); encodes a novel zinc-finger protein with a	-1.12	1.53E-06
At3g63310	BIL4 (BRZ-INSENSITIVE-LONG HYPOCOTYLS 4); Mediates cell elongation in brassinosteroid signaling.	2.979	3.29E-11
At3g63320	Protein phosphatase 2C family protein	2.679	6.09E-10
At3g63340	Protein phosphatase 2C family protein	1.69	9.41E-09
At3g63350	HSFA7B (HEAT SHOCK TRANSCRIPTION FACTOR A7B); member of Heat Stress Transcription Factor (I	5.432	1.34E-10
At3g63380	ATPase E1-E2 type family protein / haloacid dehalogenase-like hydrolase family protein	1.157	0.00239
At4g00165	Bifunctional inhibitor/lipid-transfer protein/seed storage 2S albumin superfamily protein	-1.246	7.04E-07

At4g00236	pseudogene, similar to leaf senescence-associated receptor-like protein kinase, blastp match of 56% identity ar	2.155	1.02E-08
At4g00300	fringe-related protein	1.193	1.52E-06
At4g00342	unknown protein	4.107	9.27E-07
At4g00500	alpha/beta-Hydrolases superfamily protein	1.424	6.51E-09
At4g00540	ATMYB3R2 (myb domain protein 3r2); Encodes a putative c-myb-like transcription factor. Member of a class	6.364	1.52E-14
At4g00550	DGD2 (digalactosyl diacylglycerol deficient 2); encodes a UDP-galactose-dependent digalactosyldiacylglycero	3.352	2.49E-07
At4g00590	N-terminal nucleophile aminohydrolases (Ntn hydrolases) superfamily protein	1.152	5.32E-07
At4g00670	Remorin family protein	2.108	1.01E-06
At4g00760	APRR8 (pseudo-response regulator 8); Encodes a response-regulator like protein.	1.982	1.35E-09
At4g00820	iqd17 (IQ-domain 17)	-1.604	7.22E-08
At4g00870	basic helix-loop-helix (bHLH) DNA-binding superfamily protein	-1.678	3.58E-07
At4g00940	Dof-type zinc finger DNA-binding family protein	2.597	1.19E-09
At4g00950	MEE47 (maternal effect embryo arrest 47)	-1.091	6.02E-07
At4g01010	ATCNGC13 (CYCLIC NUCLEOTIDE-GATED CHANNEL 13); member of Cyclic nucleotide gated channel f	2.089	1.72E-07
At4g01070	UGT72B1 (UDP-GLUCOSE-DEPENDENT GLUCOSYLTRANSFERASE 72 B1); the glycosyltransferase (U	1.045	2.71E-05
At4g01130	GDSL-like Lipase/Acylhydrolase superfamily protein	-1.435	1.45E-05
At4g01250	WRKY22 (WRKY family transcription factor); member of WRKY Transcription Factor; Group II-e	2.228	2.38E-07
At4g01280	Homeodomain-like superfamily protein	1.416	1.28E-06
At4g01320	ATSTE24 (Peptidase family M48 family protein); CAAX protease with broad substrate specificity. Localized (	1.121	2.69E-07
At4g01330	Protein kinase superfamily protein	-1.392	4.02E-06
At4g01360	unknown protein	1.348	0.01131
At4g01430	nodulin MtN21 /EamA-like transporter family protein	-1.838	8.33E-07
At4g01450	nodulin MtN21 /EamA-like transporter family protein	-1.078	7.03E-08
At4g01540	ANAC068 (Arabidopsis NAC domain containing protein 68); Encodes a membrane-bound NAC (for NAM, A	1.093	0.00013
At4g01550	anac069 (NAC domain containing protein 69)	1.427	6.93E-07
At4g01600	GRAM domain family protein	-1.281	1.70E-06
At4g01670	unknown protein	-1.151	3.87E-05
At4g01720	WRKY47 (WRKY family transcription factor); member of WRKY Transcription Factor; Group II-b	-1.176	0.0001
At4g01740	Cysteine/Histidine-rich C1 domain family protein	-1.097	3.90E-06
At4g01870	tolB protein-related	2.556	1.77E-07
At4g01895	systemic acquired resistance (SAR) regulator protein NIMIN-1-related	1.333	1.10E-06
At4g02050	STP7 (sugar transporter protein 7)	-1.795	3.43E-07
At4g02090	unknown protein	2.347	1.17E-08
At4g02140	unknown protein	2.325	4.55E-11
At4g02195	SYP42 (syntaxin of plants 42); member of SYP4 Gene Family	2.379	5.84E-11
At4g02200	Drought-responsive family protein	2.664	9.05E-10
At4g02290	AtGH9B13 (glycosyl hydrolase 9B13)	-1.309	0.0009
At4g02380	AtLEA5 (Arabidopsis thaliana late embryogenensis abundant like 5); Encodes AtLEA5 (late embryogenesis ab	1.484	9.98E-06
At4g02410	Concanavalin A-like lectin protein kinase family protein	2.009	5.62E-08
At4g02425	unknown protein	2.962	5.86E-11
At4g02550	unknown protein	3.282	2.50E-12
At4g02655	unknown protein	1.185	5.59E-08
At4g02690	Bax inhibitor-1 family protein	3.25	7.21E-07
At4g02730	Transducin/WD40 repeat-like superfamily protein	1.73	4.26E-09
At4g02740	F-box/RNI-like superfamily protein	1.183	6.46E-09
At4g02810	Protein of unknown function (DUF3049)	-1.04	5.24E-06
At4g02920	unknown protein	1.066	1.60E-07
At4g02940	oxidoreductase, 2OG-Fe(II) oxygenase family protein	1.193	7.86E-07
At4g02950	Ubiquitin family protein	4.767	1.11E-12
At4g02980	ABP1 (endoplasmic reticulum auxin binding protein 1); Auxin binding protein involved in cell elongation and	2.205	1.34E-10
At4g03010	RNI-like superfamily protein	-1.015	2.82E-05
At4g03038	other RNA; Unknown gene	-2.919	8.41E-09
At4g03060	similar to AOP3 (2-oxoglutarate?dependent dioxygenase 3), oxidoreductase, acting on paired donors, with inco	-4.311	4.68E-11
At4g03110	AtRBP-DR1 (RNA-binding protein-defense related 1); Encodes a putative RNA-binding protein that is located	1.163	2.47E-06
At4g03113	unknown protein	1.158	2.38E-07
At4g03230	S-locus lectin protein kinase family protein	1.21	1.80E-07
At4g03240	FH (frataxin homolog); Encodes AtFH, a frataxin homolog. Frataxin is required for the biogenesis of mitochon	1.02	3.34E-06
At4g03292	Polynucleotidyl transferase, ribonuclease H-like superfamily protein	1.447	4.33E-05
At4g03320	tic20-IV (translocon at the inner envelope membrane of chloroplasts 20-IV)	2.157	6.18E-08
At4g03330	SYP123 (syntaxin of plants 123); member of SYP12 Gene Family	1.098	1.52E-05
At4g03360	Ubiquitin family protein	3.136	1.81E-09
At4g03420	Protein of unknown function (DUF789)	-1.004	3.32E-07
At4g03430	EMB2770 (EMBRYO DEFECTIVE 2770); Encodes a nuclear protein similar to the human U5 small ribonucle	1.436	8.37E-08
At4g03450	Ankyrin repeat family protein	3.333	0.00016
At4g03510	RMA1 (RING membrane-anchor 1); RMA1 encodes a novel 28 kDa protein with a RING finger motif and a C	-1.065	0.0177
At4g03965	RING/U-box superfamily protein	1.09	2.24E-06
At4g04020	FIB (fibrillin); Fibrillin precursor protein. The fibrillin preprotein, but not the mature protein interacts with AE	3.532	9.40E-10

At4g04293	pseudogene, similar to P0703B11.15, blastp match of 38% identity and 2.2e-25 P-value to GP|18844826|dbj|B	-1.822	1.43E-06
At4g04330	Chaperonin-like RbcX protein	-3.292	9.75E-08
At4g04340	ERD (early-responsive to dehydration stress) family protein	-1.575	1.05E-07
At4g04490	CRK36 (cysteine-rich RLK (RECEPTOR-like protein kinase) 36); Encodes a cysteine-rich receptor-like protei	1.002	0.04003
At4g04540	CRK39 (cysteine-rich RLK (RECEPTOR-like protein kinase) 39); Encodes a cysteine-rich receptor-like protei	4.112	4.78E-12
At4g04610	APR1 (APS reductase 1); Encodes a protein disulfide isomerase-like (PDIL) protein, a member of a multigene	2.49	7.81E-09
At4g04655	non-LTR retrotransposon family (LINE), has a 1.0e-19 P-value blast match to GB:NP_038602 L1 repeat, Tf su	1.411	3.67E-08
At4g04745	unknown protein	-1.355	0.00328
At4g04750	Major facilitator superfamily protein	-1.351	1.01E-06
At4g04800	ATMSRB3 (methionine sulfoxide reductase B3)	1.581	1.35E-10
At4g04830	ATMSRB5 (methionine sulfoxide reductase B5)	1.139	3.16E-05
At4g04955	ALN (allantoinase); Encodes an allantoinase which is involved in allantoin degradation and assimilation. Gene	-1.058	1.26E-05
At4g04970	ATGSL01 (GLUCAN SYNTHASE LIKE 1); encodes a gene similar to callose synthase	-1.043	1.40E-05
At4g05010	F-box family protein	1.494	1.17E-08
At4g05018	unknown protein	1.805	9.34E-06
At4g05020	NDB2 (NAD(P)H dehydrogenase B2)	3.467	1.64E-08
At4g05130	ENT4 (equilibrative nucleoside transporter 4)	-2.025	1.35E-08
At4g05490	RNI-like superfamily protein	1.094	2.14E-06
At4g06521	non-LTR retrotransposon family (LINE), has a 1.8e-23 P-value blast match to GB:NP_038604 L1 repeat, Tf su	-2.136	2.08E-06
At4g07675	unknown protein	2.691	6.85E-09
At4g08230	glycine-rich protein	2.374	2.10E-09
At4g08290	nodulin MtN21 /EamA-like transporter family protein	-1.348	5.20E-07
At4g08300	nodulin MtN21 /EamA-like transporter family protein	-2.791	7.67E-07
At4g08320	Tetratricopeptide repeat (TPR)-like superfamily protein	2.721	3.36E-12
At4g08330	unknown protein	1.201	7.14E-09
At4g08500	MEKK1 (MAPK/ERK kinase kinase 1); Encodes a member of the A1 subgroup of the MEKK (MAPK/ERK ki	1.119	2.79E-07
At4g08555	unknown protein	2.622	2.52E-06
At4g08590	ORL1 (ORTH-LIKE 1)	1.044	3.21E-06
At4g08685	SAH7 (Pollen Ole e 1 allergen and extensin family protein); Encodes a protein, expressed in leaves, with simil	-1.104	8.30E-07
At4g08920	BLU1 (BLUE LIGHT UNINHIBITED 1); Encodes CRY1, a flavin-type blue-light photoreceptor with ATP bin	-1.249	7.72E-08
At4g09020	ISA3 (isoamylase 3); Encodes an isoamylase-like protein. Mutant studies show that the gene is strongly involv	-1.253	2.03E-05
At4g09080	ATTOC75-IV (TRANSLOCON OUTER MEMBRANE COMPLEX 75-IV); Component of the translocon oute	2.001	4.85E-09
At4g09150	T-complex protein 11	4.224	3.17E-12
At4g09300	LisH and RanBPM domains containing protein	2.274	6.24E-08
At4g09420	Disease resistance protein (TIR-NBS class)	-1.178	1.61E-05
At4g09570	CPK4 (calcium-dependent protein kinase 4); Encodes a member of Calcium Dependent Protein Kinase (CDPK	1.991	5.70E-10
At4g09750	NAD(P)-binding Rossmann-fold superfamily protein	1.698	0.00013
At4g09760	Protein kinase superfamily protein; encodes a choline synthase whose gene expression is induced by high salt a	-1.335	2.11E-06
At4g09890	Protein of unknown function (DUF3511)	-1.447	1.25E-07
At4g10040	CYTC-2 (cytochrome c-2); Encodes cytochrome c. Promoter directs preferential expression in vascular tissues	2.601	3.37E-09
At4g10110	RNA-binding (RRM/RBD/RNP motifs) family protein	1.142	6.18E-07
At4g10120	ATSPS4F (Sucrose-phosphate synthase family protein); Encodes a protein with putative sucrose-phosphate syn	-1.497	0.00014
At4g10150	RING/U-box superfamily protein	-1.247	0.00054
At4g10160	RING/U-box superfamily protein	-1.29	0.00031
At4g10240	B-box zinc finger family protein	3.582	1.03E-10
At4g10250	ATHSP22.0 (HSP20-like chaperones superfamily protein); Columbia endomembrane-localized small heat shoc	9.933	4.23E-10
At4g10280	RmlC-like cupins superfamily protein	-1.303	3.12E-06
At4g10310	HKT1 (high-affinity K+ transporter 1); encodes a sodium transporter (HKT1) expressed in xylem parenchyma	-1.631	2.11E-07
At4g10410	Leucine-rich repeat (LRR) family protein	2.484	3.66E-10
At4g10420	FBD / Leucine Rich Repeat domains containing protein	1.28	1.54E-07
At4g10430	TMPIT-like protein	1.187	1.34E-07
At4g10440	S-adenosyl-L-methionine-dependent methyltransferases superfamily protein	1.055	2.32E-07
At4g10500	2-oxoglutarate (2OG) and Fe(II)-dependent oxygenase superfamily protein	-2.795	0.00485
At4g10910	unknown protein	-1.514	4.35E-05
At4g11000	Ankyrin repeat family protein	1.097	2.03E-05
At4g11220	BTI2 (VIRB2-interacting protein 2)	2.068	2.19E-10
At4g11330	ATMPK5 (MAP kinase 5); MAP kinase	1.321	1.15E-07
At4g11350	Protein of unknown function (DUF604)	1.353	7.48E-06
At4g11370	RHA1A (RING-H2 finger A1A); Encodes a putative RING-H2 finger protein RHA1a.	1.454	1.14E-06
At4g11460	CRK30 (cysteine-rich RLK (RECEPTOR-like protein kinase) 30); Encodes a cysteine-rich receptor-like protei	-1.91	5.21E-07
At4g11470	CRK31 (cysteine-rich RLK (RECEPTOR-like protein kinase) 31); Encodes a cysteine-rich receptor-like protei	2.186	1.99E-08
At4g11650	ATOSM34 (osmotin 34); osmotin-like protein	-2.244	0.0266
At4g11660	HSFB2B (HEAT SHOCK TRANSCRIPTION FACTOR B2B); member of Heat Stress Transcription Factor (I	3.515	2.50E-12
At4g11740	SAY1 (Ubiquitin-like superfamily protein); Isolated as a suppressor of a dominant mutant in the Ara4 gene tha	2.868	9.53E-12
At4g11910	INVOLVED IN: biological_process unknown	-2.124	2.90E-06
At4g12030	BASS5 (BILE ACID:SODIUM SYMPORTER FAMILY PROTEIN 5); Required for the biosynthesis of methi	-3.653	1.83E-10
At4g12090	Cornichon family protein	-2.25	2.92E-06

At4g12120	SEC1B (Sec1/munc18-like (SM) proteins superfamily); member of KEULE Gene Family	4.298	2.48E-12
At4g12320	CYP706A6 (cytochrome P450, family 706, subfamily A, polypeptide 6); member of CYP706A	-2.093	2.22E-06
At4g12334	Cytochrome P450 superfamily protein	1.391	2.32E-06
At4g12390	PME1 (pectin methylesterase inhibitor 1)	-1.286	8.03E-05
At4g12400	stress-inducible protein, putative	7.205	1.43E-12
At4g12410	SAUR-like auxin-responsive protein family	3.822	6.55E-11
At4g12420	SKU5 (Cupredoxin superfamily protein); Encodes a protein of unknown function involved in directed root tip	-1.01	2.13E-07
At4g12470	AZI1 (azelaic acid induced 1); Encodes AZI1 (AZELAIC ACID INDUCED 1). Involved in the priming of sali	-1.813	0.00457
At4g12480	pEARLI 1 (Bifunctional inhibitor/lipid-transfer protein/seed storage 2S albumin superfamily protein); a putativ	-1.679	0.04013
At4g12510	Bifunctional inhibitor/lipid-transfer protein/seed storage 2S albumin superfamily protein	-4.67	2.17E-07
At4g12520	Bifunctional inhibitor/lipid-transfer protein/seed storage 2S albumin superfamily protein	-1.923	1.44E-06
At4g12610	RAP74 (transcription activators;DNA binding;RNA polymerase II transcription factors;catalytics;transcription	1.769	0.0045
At4g12720	AtNUDT7 (Arabidopsis thaliana Nudix hydrolase homolog 7); Encodes a protein with ADP-ribose hydrolase a	3.011	1.59E-08
At4g12735	unknown protein	3.5	9.30E-07
At4g12830	alpha/beta-Hydrolases superfamily protein	1.35	8.75E-06
At4g12900	Gamma interferon responsive lysosomal thiol (GILT) reductase family protein	-1.283	2.52E-06
At4g13010	Oxidoreductase, zinc-binding dehydrogenase family protein	1.375	4.48E-09
At4g13110	BSD domain-containing protein	1.345	2.77E-06
At4g13180	NAD(P)-binding Rossmann-fold superfamily protein	3.37	8.25E-11
At4g13250	NYC1 (NON-YELLOW COLORING 1); Encodes a chlorophyll b reductase involved in the degradation of chl	-1.571	1.76E-07
At4g13280	TPS12 (terpenoid synthase 12); Catalyzes the conversion of farnesyl diphosphate to (Z)-gamma-bisabolene and	-1.063	0.01357
At4g13300	TPS13 (terpenoid synthase 13); Catalyzes the conversion of farnesyl diphosphate to (Z)-gamma-bisabolene and	-1.222	0.00232
At4g13345	MEE55 (maternal effect embryo arrest 55)	-1.172	1.65E-06
At4g13493	MIR850A (microRNA850A); Encodes a microRNA of unknown function. MicroRNAs are regulatory RNAs w	-1.275	4.89E-07
At4g13494	MIR863A (microRNA863A); Encodes a microRNA of unknown function. MicroRNAs are regulatory RNAs w	-1.323	2.23E-08
At4g13495	other RNA; Unknown gene	-1.204	2.57E-06
At4g13510	AMT1.1 (ammonium transporter 1.1); Encodes a plasma membrane localized ammonium transporter. Contain	1.059	1.95E-06
At4g13550	triglyceride lipases	2.366	2.34E-10
At4g13575	unknown protein	-1.03	2.98E-06
At4g13620	Integrase-type DNA-binding superfamily protein; encodes a member of the DREB subfamily A-6 of ERF/AP2	2.454	2.28E-09
At4g13650	Pentatricopeptide repeat (PPR) superfamily protein	-1.027	1.18E-06
At4g13660	PRR2 (pinoresinol reductase 2); Encodes a pinoresinol reductase involved in lignan biosynthesis. Expressed st	-1.131	0.00011
At4g13700	PAP23 (purple acid phosphatase 23)	-1.42	0.00059
At4g13770	CYP83A1 (cytochrome P450, family 83, subfamily A, polypeptide 1); Encodes a cytochrome p450 enzyme tha	-3.505	5.43E-13
At4g13810	AtRLP47 (receptor like protein 47)	-1.1	5.51E-05
At4g13840	HXXXD-type acyl-transferase family protein	-1.152	4.13E-06
At4g14030	SBP1 (selenium-binding protein 1)	1.095	0.0122
At4g14040	EDA38 (EMBRYO SAC DEVELOPMENT ARREST 38)	-1.277	6.59E-08
At4g14220	RHF1A (RING-H2 group F1A); encodes a RING-type E3 ubiquitin ligase implicated in gametogenesis. RHF1.	1.69	1.32E-07
At4g14225	A20/AN1-like zinc finger family protein	1.613	1.94E-06
At4g14230	CBS domain-containing protein with a domain of unknown function (DUF21)	-1.095	5.24E-07
At4g14250	structural constituent of ribosome	-1.165	0.00048
At4g14300	RNA-binding (RRM/RBD/RNP motifs) family protein	1.805	1.43E-08
At4g14365	XBAT34 (XB3 ortholog 4 in Arabidopsis thaliana)	1.888	3.45E-05
At4g14370	Disease resistance protein (TIR-NBS-LRR class) family	2.075	5.67E-06
At4g14380	unknown protein	-1.346	2.29E-07
At4g14385	FUNCTIONS IN: molecular_function unknown; INVOLVED IN: biological_process unknown; LOCATED IN	1.161	2.74E-06
At4g14480	Protein kinase superfamily protein	-2.093	6.46E-08
At4g14540	NF-YB3 (nuclear factor Y, subunit B3)	-1.425	2.84E-07
At4g14548	other RNA	-1.757	1.50E-07
At4g14560	AXR5 (AUXIN RESISTANT 5); auxin (indole-3-acetic acid) induced gene (IAA1) encoding a short-lived nuc	-1.566	5.99E-07
At4g14650	unknown protein	-1.274	6.59E-06
At4g14690	ELIP2 (EARLY LIGHT-INDUCIBLE PROTEIN 2); Encodes an early light-induced protein. ELIPs are though	3.533	2.55E-09
At4g14695	Uncharacterised protein family (UPF0041)	1.036	1.48E-06
At4g14750	IQD19 (IQ-domain 19)	-1.141	1.12E-05
At4g15130	CCT2 (phosphorylcholine cytidylyltransferase2)	1.053	1.37E-07
At4g15210	ATBETA-AMY (ARABIDOPSIS THALIANA BETA-AMYLASE); cytosolic beta-amylase expressed in roset	-1.795	0.0069
At4g15248	B-box type zinc finger family protein	3.91	4.65E-07
At4g15280	UGT71B5 (UDP-glucosyl transferase 71B5)	1.671	1.08E-09
At4g15350	CYP705A2 (cytochrome P450, family 705, subfamily A, polypeptide 2); member of CYP705A	1.147	0.00109
At4g15410	PUX5 (serine/threonine protein phosphatase 2A 55 kDa regulatory subunit B prime gamma)	1.443	8.51E-05
At4g15420	Ubiquitin fusion degradation UFD1 family protein	3.064	1.15E-06
At4g15470	Bax inhibitor-1 family protein	1.004	8.76E-06
At4g15540	EamA-like transporter family	-1.362	2.51E-06
At4g15660	Thioredoxin superfamily protein	-2.237	4.70E-07
At4g15670	Thioredoxin superfamily protein	-2.334	3.33E-08
At4g15680	Thioredoxin superfamily protein	-2.604	1.27E-07

At4g15690	Thioredoxin superfamily protein	-1.689	0.0036
At4g15700	Thioredoxin superfamily protein	-2.774	5.37E-09
At4g15880	ESD4 (EARLY IN SHORT DAYS 4); EARLY IN SHORT DAYS 4 Arabidopsis mutant shows extreme early f	1.405	6.75E-08
At4g15975	RING/U-box superfamily protein	2.836	7.84E-09
At4g16000	unknown protein	-1.118	0.00064
At4g16008	unknown protein	-1.535	8.22E-05
At4g16146	cAMP-regulated phosphoprotein 19-related protein	-2.214	4.92E-08
At4g16215	unknown protein	1.607	1.83E-07
At4g16460	unknown protein	1.738	6.30E-06
At4g16500	Cystatin/monellin superfamily protein	1.532	9.08E-09
At4g16563	Eukaryotic aspartyl protease family protein	-1.64	0.00016
At4g16580	Protein phosphatase 2C family protein	1.028	1.13E-06
At4g16660	heat shock protein 70 (Hsp 70) family protein	2.288	3.43E-09
At4g16680	P-loop containing nucleoside triphosphate hydrolases superfamily protein	2.698	3.45E-07
At4g16690	ATMES16 (ARABIDOPSIS THALIANA METHYL ESTERASE 16); Encodes a protein shown to have carbox	1.777	3.75E-08
At4g16760	ACX1 (acyl-CoA oxidase 1); Encodes a medium to long-chain acyl-CoA oxidase. Catalyzes the first step of fat	1.056	8.36E-06
At4g16820	PLA-I{beta]2 (phospholipase A I beta 2); Encodes a lipase that hydrolyzes phosphatidylcholine, glycolipids as	4.41	4.83E-11
At4g16940	Disease resistance protein (TIR-NBS-LRR class) family	1.924	1.62E-08
At4g17030	ATEXLB1 (expansin-like B1); Encodes EXLB1 (expansin-like B1), a member of the expansin family.	-1.062	0.0007
At4g17070	peptidyl-prolyl cis-trans isomerases	1.57	4.70E-09
At4g17098	other RNA; Potential natural antisense gene, locus overlaps with AT4G17100	2.4	1.52E-08
At4g17100	CONTAINS InterPro DOMAIN/s: Endoribonuclease XendoU (InterPro:IPR018998); Has 943 Blast hits to 770	-1.118	2.11E-06
At4g17160	ATRABB1A (RAB GTPase homolog B1A)	2.08	1.95E-07
At4g17250	unknown protein	4.322	6.61E-12
At4g17260	Lactate/malate dehydrogenase family protein	1.476	7.66E-07
At4g17280	Auxin-responsive family protein	-1.192	0.0006
At4g17460	HAT1 (Homeobox-leucine zipper protein 4 (HB-4) / HD-ZIP protein); Encodes homeobox protein HAT1.	-1.485	1.90E-07
At4g17490	ATERF6 (ethylene responsive element binding factor 6); Encodes a member of the ERF (ethylene response fac	2.391	2.80E-07
At4g17530	ATRAB1C (RAB GTPase homolog 1C)	1.509	2.91E-08
At4g17580	Bax inhibitor-1 family protein	1.725	1.25E-05
At4g17585	Aluminium activated malate transporter family protein	1.006	6.30E-07
At4g17615	ATCBL1 (ARABIDOPSIS THALIANA CALCINEURIN B-LIKE PROTEIN); Member of AtCBL (Calcineurin	2.38	4.34E-11
At4g17616	Pentatricopeptide repeat (PPR) superfamily protein	1.657	1.25E-08
At4g17650	Polyketide cyclase / dehydrase and lipid transport protein	1.682	1.42E-09
At4g17840	FUNCTIONS IN: molecular_function unknown; INVOLVED IN: biological_process unknown; LOCATED IN	1.981	9.13E-07
At4g17900	PLATZ transcription factor family protein	2.322	3.27E-10
At4g18240	ATSS4 (ARABIDOPSIS THALIANA STARCH SYNTHASE 4)	-1.234	6.57E-08
At4g18250	receptor serine/threonine kinase, putative	1.403	3.45E-05
At4g18340	Glycosyl hydrolase superfamily protein	-1.664	3.13E-08
At4g18450	Integrase-type DNA-binding superfamily protein; encodes a member of the ERF (ethylene response factor) sub	2.446	5.39E-10
At4g18610	LSH9 (LIGHT SENSITIVE HYPOCOTYLS 9)	-1.132	4.14E-06
At4g18630	Protein of unknown function (DUF688)	-1.353	1.11E-06
At4g18823	Defensin-like (DEFL) family protein; Encodes a defensin-like (DEFL) family protein.	1.339	1.19E-07
At4g18880	AT-HSFA4A (ARABIDOPSIS THALIANA HEAT SHOCK TRANSCRIPTION FACTOR A4A); member of	2.242	1.09E-09
At4g18890	BEH3 (BES1/BZR1 homolog 3)	1.544	2.88E-08
At4g18930	RNA ligase/cyclic nucleotide phosphodiesterase family protein	-1.428	3.51E-07
At4g18950	Integrin-linked protein kinase family	1.278	3.41E-06
At4g19038	LCR15 (low-molecular-weight cysteine-rich 15); Encodes a member of a family of small,secreted, cysteine rich	1.729	3.17E-09
At4g19120	ERD3 (early-responsive to dehydration 3)	-2.079	1.01E-09
At4g19170	CCD4 (carotenoid cleavage dioxygenase 4); chloroplast-targeted member of a family of enzymes similar to nin	-3.13	1.65E-08
At4g19380	Long-chain fatty alcohol dehydrogenase family protein	-1.203	2.19E-05
At4g19460	UDP-Glycosyltransferase superfamily protein	1.145	3.49E-06
At4g19470	Leucine-rich repeat (LRR) family protein	1.84	4.57E-10
At4g19515	NA	1.922	1.07E-05
At4g19520	disease resistance protein (TIR-NBS-LRR class) family	2.135	2.17E-05
At4g19633	pseudogene of heat shock factor related protein; pseudogene of heat shock factor related protein	1.21	0.00016
At4g19640	ATRAB-F2B (ARABIDOPSIS RAB GTPASE HOMOLOG F2B); Encodes Ara7.	1.12	1.17E-07
At4g19700	RING (SBP (S-ribonuclease binding protein) family protein)	-1.304	1.10E-08
At4g19810	Glycosyl hydrolase family protein with chitinase insertion domain	-1.412	0.01114
At4g19820	Glycosyl hydrolase family protein with chitinase insertion domain	-1.153	3.88E-05
At4g19970	CONTAINS InterPro DOMAIN/s: Nucleotide-diphospho-sugar transferase, predicted (InterPro:IPR005069); B	-1.316	8.68E-07
At4g20000	VQ motif-containing protein	-1.445	0.00066
At4g20160	LOCATED IN: chloroplast	1.834	4.93E-06
At4g20230	Terpenoid cyclases/Protein prenyltransferases superfamily protein	-2.514	1.23E-09
At4g20270	BAM3 (BARELY ANY MERISTEM 3); Encodes a CLAVATA1-related receptor kinase-like protein required	-1.067	6.67E-05
At4g20300	Protein of unknown function (DUF1639)	1.008	7.01E-08
At4g20330	Transcription initiation factor TFIIE, beta subunit	1.23	7.11E-07

At4g20380	LSD1 (LESION SIMULATING DISEASE); LSD1 monitors a superoxide-dependent signal and negatively reg	1.162	1.13E-06
At4g20460	NAD(P)-binding Rossmann-fold superfamily protein	1.127	3.01E-06
At4g20690	unknown protein	1.243	5.51E-06
At4g20780	CML42 (calmodulin like 42); Calcium sensor involved in trichome branching.	2.48	5.39E-08
At4g20820	FAD-binding Berberine family protein	1.175	0.0446
At4g20830	FAD-binding Berberine family protein	1.689	4.94E-07
At4g20860	FAD-binding Berberine family protein	2.223	8.10E-09
At4g20990	ACA4 (alpha carbonic anhydrase 4)	3.023	1.81E-08
At4g21090	ATMFDX2 (ARABIDOPSIS MITOCHONDRIAL FERREDOXIN 2)	2.426	1.30E-09
At4g21120	AAT1 (amino acid transporter 1); Encodes a member of the cationic amino acid transporter (CAT) subfamily c	2.386	1.05E-06
At4g21200	ATGA2OX8 (ARABIDOPSIS THALIANA GIBBERELLIN 2-OXIDASE 8); Encodes a protein with gibberell	-1.268	1.53E-06
At4g21215	unknown protein	-1.473	1.52E-06
At4g21310	Protein of unknown function (DUF1218)	1.592	5.74E-09
At4g21320	HSA32 (HEAT-STRESS-ASSOCIATED 32); Encodes heat-stress-associated 32-kD protein. Up-regulated by l	6.669	2.33E-12
At4g21323	Subtilase family protein	5.792	1.49E-12
At4g21330	DYT1 (DYSFUNCTIONAL TAPETUM 1); Encodes a bHLH transcription factor strongly expressed in the tap	2.357	4.83E-11
At4g21390	B120 (S-locus lectin protein kinase family protein)	4.1	2.31E-10
At4g21580	oxidoreductase, zinc-binding dehydrogenase family protein	2.077	2.03E-08
At4g21590	ENDO3 (endonuclease 3); Encodes a putative endonuclease but no demonstrable endonuclease activity, either	2.029	6.14E-07
At4g21620	glycine-rich protein	-1.087	1.85E-06
At4g21650	Subtilase family protein	-1.247	2.40E-07
At4g21810	DER2.1 (DERLIN-2.1)	1.516	2.86E-09
At4g21865	unknown protein	1.899	2.13E-08
At4g21920	unknown protein	1.18	0.00745
At4g21970	Protein of unknown function, DUF584	-1.232	3.95E-06
At4g21990	APR3 (APS reductase 3); Encodes a protein disulfide isomerase-like (PDIL) protein, a member of a multigene	3.685	8.80E-11
At4g22050	Eukaryotic aspartyl protease family protein	-1.016	0.00113
At4g22230	Arabidopsis defensin-like protein; Encodes a defensin-like (DEFL) family protein.	-1.196	0.00013
At4g22240	Plastid-lipid associated protein PAP / fibrillin family protein	1.049	2.53E-07
At4g22285	Ubiquitin C-terminal hydrolases superfamily protein	1.077	9.26E-08
At4g22340	CDS2 (cytidinediphosphate diacylglycerol synthase 2)	1.88	3.06E-09
At4g22350	Ubiquitin C-terminal hydrolases superfamily protein	2.3	2.00E-10
At4g22470	protease inhibitor/seed storage/lipid transfer protein (LTP) family protein	-2.336	0.00036
At4g22485	Bifunctional inhibitor/lipid-transfer protein/seed storage 2S albumin superfamily protein; Encodes a Protease i	-1.403	0.00108
At4g22505	Bifunctional inhibitor/lipid-transfer protein/seed storage 2S albumin superfamily protein	-1.226	0.00131
At4g22530	S-adenosyl-L-methionine-dependent methyltransferases superfamily protein	2.15	1.90E-08
At4g22540	ORP2A (OSBP(oxysterol binding protein)-related protein 2A)	-1.231	1.21E-06
At4g22700	LBD32 (LOB domain-containing protein 32)	1.021	7.47E-07
At4g22730	Leucine-rich repeat protein kinase family protein	-1.38	2.42E-08
At4g22740	glycine-rich protein	2.126	5.43E-11
At4g22780	ACR7 (ACT domain repeat 7); Member of a family of ACT domain containing proteins . ACT domains are inv	1.306	0.0002
At4g22790	MATE efflux family protein	-1.808	1.90E-08
At4g22820	A20/AN1-like zinc finger family protein	1.166	2.68E-07
At4g22960	Protein of unknown function (DUF544)	1.578	1.95E-06
At4g22980	FUNCTIONS IN: molecular_function unknown; INVOLVED IN: biological_process unknown; LOCATED IN	1.947	4.45E-07
At4g23010	UTR2 (UDP-galactose transporter 2)	1.147	5.44E-07
At4g23130	CRK5 (cysteine-rich RLK (RECEPTOR-like protein kinase) 5); Encodes a receptor-like protein kinase. Namin	-1.85	2.31E-06
At4g23140	CRK6 (cysteine-rich RLK (RECEPTOR-like protein kinase) 6); Arabidopsis thaliana receptor-like protein kina	-1.915	0.0003
At4g23150	CRK7 (cysteine-rich RLK (RECEPTOR-like protein kinase) 7); Encodes a cysteine-rich receptor-like protein k	-1.876	0.01108
At4g23180	CRK10 (cysteine-rich RLK (RECEPTOR-like protein kinase) 10); Encodes a receptor-like protein kinase. Nam	1.002	0.00026
At4g23190	AT-RLK3 (RECEPTOR LIKE PROTEIN KINASE 3); Encodes putative receptor-like protein kinase that is inc	3.099	8.03E-10
At4g23215	pseudogene of cysteine-rich receptor-like protein kinase family protein; pseudogene of cysteine-rich receptor-l	3.114	3.01E-08
At4g23220	CRK14 (cysteine-rich RLK (RECEPTOR-like protein kinase) 14); Encodes a cysteine-rich receptor-like protei	1.394	3.05E-05
At4g23240	CRK16 (cysteine-rich RLK (RECEPTOR-like protein kinase) 16); Encodes a cysteine-rich receptor-like protei	-1.967	8.36E-08
At4g23280	CRK20 (cysteine-rich RLK (RECEPTOR-like protein kinase) 20); Encodes a cysteine-rich receptor-like protei	1.035	6.31E-05
At4g23300	CRK22 (cysteine-rich RLK (RECEPTOR-like protein kinase) 22); Encodes a cysteine-rich receptor-like protei	-1.836	1.34E-07
At4g23340	2-oxoglutarate (2OG) and Fe(II)-dependent oxygenase superfamily protein	-1.238	7.52E-05
At4g23450	RING/U-box superfamily protein	2.594	2.61E-09
At4g23470	PLAC8 family protein	1.068	3.55E-05
At4g23493	unknown protein	6.822	9.73E-13
At4g23550	WRKY29 (WRKY family transcription factor); Encodes WRKY DNA-binding protein 29 (WRKY29).	-1.207	4.94E-07
At4g23570	SGT1A (phosphatase-related); Closely related to SGT1B, may function in SCF(TIR1) mediated protein degrad	3.005	5.81E-11
At4g23700	ATCHX17 (cation/H+ exchanger 17); member of Putative Na+/H+ antiporter family	-1.796	2.79E-05
At4g23720	Protein of unknown function (DUF1191)	-1.088	5.31E-05
At4g23850	LACS4 (long-chain acyl-CoA synthetase 4)	1.336	5.20E-08
At4g23860	PHD finger protein-related	1.032	1.50E-06
At4g23880	unknown protein	2.143	8.32E-11

At4g23885	unknown protein	2.515	3.20E-10
At4g23895	Pleckstrin homology (PH) domain-containing protein	1.049	1.17E-06
At4g23990	ATCSLG3 (ARABIDOPSIS THALIANA CELLULOSE SYNTHASE-LIKE G3); encodes a protein similar to	1.301	2.62E-06
At4g24000	ATCSLG2 (ARABIDOPSIS THALIANA CELLULOSE SYNTHASE LIKE G2); encodes a protein similar to	-1.46	0.0007
At4g24020	NLP7 (NIN like protein 7); Encodes NIN Like Protein 7 (NLP7). Modulates nitrate sensing and metabolism.	1.545	5.99E-08
At4g24110	unknown protein	1.317	0.01262
At4g24120	ATYSL1 (YELLOW STRIPE LIKE 1); Member of a small family of oligopeptide transporters similar to the ye	-1.725	8.38E-06
At4g24130	Protein of unknown function, DUF538	-1.21	3.88E-06
At4g24160	alpha/beta-Hydrolases superfamily protein; Encodes a soluble lysophosphatidic acid acyltransferase with addit	3.624	1.30E-10
At4g24170	ATP binding microtubule motor family protein	2.099	3.70E-08
At4g24370	unknown protein	1	9.12E-07
At4g24380	INVOLVED IN: 10-formyltetrahydrofolate biosynthetic process, folic acid and derivative biosynthetic process	3.262	6.56E-09
At4g24410	unknown protein	2.031	3.03E-06
At4g24415	MIR824A (microRNA824A); Encodes a microRNA that targets AGL16. MicroRNAs are regulatory RNAs wit	3.573	3.38E-10
At4g24420	RNA-binding (RRM/RBD/RNP motifs) family protein	1.937	3.23E-07
At4g24430	Rhamnogalacturonate lyase family protein	2.145	0.00023
At4g24510	CER2 (ECERIFERUM 2); Involved in C28 to C30 fatty acid elongation.	-1.102	0.0164
At4g24570	DIC2 (dicarboxylate carrier 2); Encodes one of the mitochondrial dicarboxylate carriers (DIC): DIC1 (AT2G2	1.45	0.00041
At4g24760	alpha/beta-Hydrolases superfamily protein	1.225	9.17E-09
At4g24890	ATPAP24 (ARABIDOPSIS THALIANA PURPLE ACID PHOSPHATASE 24)	-1.7	4.18E-05
At4g24920	secE/sec61-gamma protein transport protein	1.663	1.34E-08
At4g25000	AMY1 (alpha-amylase-like); Predicted to be secreted protein based on signalP prediction. Involved in starch m	-1.643	0.00057
At4g25030	unknown protein	1.915	3.78E-09
At4g25090	Riboflavin synthase-like superfamily protein	2.708	8.08E-11
At4g25110	AtMC2 (metacaspase 2)	-1.007	0.0032
At4g25130	PMSR4 (peptide met sulfoxide reductase 4); Encodes a chloroplast-localized methionine sulfoxide reductase tl	1.179	8.16E-08
At4g25190	Family of unknown function (DUF566)	2.169	2.02E-06
At4g25200	ATHSP23.6-MITO (mitochondrion-localized small heat shock protein 23.6); AtHSP23.6-mito mRNA, nuclear	10.493	4.81E-11
At4g25225	unknown protein	1.76	8.67E-08
At4g25260	Plant invertase/pectin methylesterase inhibitor superfamily protein	-2.034	2.43E-07
At4g25290	DNA photolyases	1.021	9.23E-06
At4g25340	FKBP53 (FK506 BINDING PROTEIN 53); Encodes a member of the FKBP-type immunophilin family that fu	1.946	2.75E-07
At4g25350	SHB1 (SHORT HYPOCOTYL UNDER BLUE1); SHB1 encodes a nuclear and cytosolic protein that has moti	3.327	8.73E-10
At4g25380	SAP10 (stress-associated protein 10)	7.129	9.57E-12
At4g25470	CBF2 (C-repeat/DRE binding factor 2); Encodes a member of the DREB subfamily A-1 of ERF/AP2 transcript	-2.003	4.87E-08
At4g25480	CBF3 (C-REPEAT BINDING FACTOR 3); encodes a member of the DREB subfamily A-1 of ERF/AP2 trans	-2.388	2.13E-09
At4g25490	CBF1 (C-repeat/DRE binding factor 1); Transcriptional activator that binds to the DRE/CRT regulatory elemer	-2.675	4.66E-09
At4g25540	MSH3 (homolog of DNA mismatch repair protein MSH3); encodes a DNA mismatch repair homolog of humar	1.576	0.00056
At4g25560	AtMYB18 (myb domain protein 18); Member of the R2R3 factor gene family.	3.894	3.29E-11
At4g25690	unknown protein	1.39	1.66E-08
At4g25700	BCH1 (BETA CAROTENOID HYDROXYLASE 1); Converts beta-carotene to zeaxanthin via cryptoxanthin.	1.538	4.73E-07
At4g25780	CAP (Cysteine-rich secretory proteins, Antigen 5, and Pathogenesis-related 1 protein) superfamily protein	-1.237	1.76E-05
At4g25810	XTH23 (xyloglucan endotransglucosylase/hydrolase 23); xyloglucan endotransglycosylase-related protein (XT	2.78	3.87E-05
At4g25820	XTH14 (xyloglucan endotransglucosylase/hydrolase 14); Encodes a xyloglucan endotransglycosylase with a cl	1.454	0.02433
At4g25835	P-loop containing nucleoside triphosphate hydrolases superfamily protein	-1.221	2.22E-06
At4g25900	Galactose mutarotase-like superfamily protein	1.031	7.51E-07
At4g25990	CIL (CCT motif family protein); chloroplast import apparatus CIA2-like. CIA2 is a transcription factor which	-1.109	6.00E-06
At4g26095	other RNA; Potential natural antisense gene, locus overlaps with AT4G26090	-1.18	1.20E-06
At4g26140	BGAL12 (beta-galactosidase 12); putative beta-galactosidase	2.819	8.37E-11
At4g26200	ACS7 (1-amino-cyclopropane-1-carboxylate synthase 7); Member of a family of proteins in Arabidopsis that e	2.319	0.00012
At4g26255	other RNA; Unknown gene	-1.852	1.01E-06
At4g26270	PFK3 (phosphofructokinase 3)	3.251	5.50E-09
At4g26320	AGP13 (arabinogalactan protein 13)	-1.025	0.001
At4g26450	unknown protein	1.87	8.27E-05
At4g26488	other RNA; Potential natural antisense gene, locus overlaps with AT4G26490	2.259	7.57E-09
At4g26490	Late embryogenesis abundant (LEA) hydroxyproline-rich glycoprotein family	-1.029	5.50E-08
At4g26530	Aldolase superfamily protein	-1.211	0.0188
At4g26540	Leucine-rich repeat receptor-like protein kinase family protein	-1.258	1.10E-05
At4g26580	RING/U-box superfamily protein	1.133	5.77E-05
At4g26670	Mitochondrial import inner membrane translocase subunit Tim17/Tim22/Tim23 family protein	-1.298	4.92E-05
At4g26740	ATPXG1 (ARABIDOPSIS THALIANA PEROXYGENASE 1); Encodes caleosin, a 27-kDa protein found wit	1.996	4.45E-09
At4g26750	hydroxyproline-rich glycoprotein family protein	1.325	4.60E-06
At4g26780	AR192 (Co-chaperone GrpE family protein); unknown function	1.322	5.44E-07
At4g26790	GDSL-like Lipase/Acylhydrolase superfamily protein	2.445	2.34E-08
At4g26830	O-Glycosyl hydrolases family 17 protein	-1.41	1.52E-05
At4g26950	Protein of unknown function, DUF584	1.235	0.00071
At4g26990	unknown protein	3.344	4.00E-10

At4g27020	unknown protein	1.427	4.08E-06
At4g27030	FAD4 (FATTY ACID DESATURASE 4); Encodes an unusual palmitate desaturase that is highly substrate spe	2.789	8.10E-09
At4g27350	Protein of unknown function (DUF1223)	2.544	1.53E-11
At4g27360	Dynein light chain type 1 family protein	5.545	4.81E-10
At4g27370	ATVIIIB (MYOSIN VIII B); member of Myosin-like proteins	1.417	2.66E-08
At4g27410	ANAC072 (Arabidopsis NAC domain containing protein 72); Encodes a NAC transcription factor induced in r	1.037	0.00147
At4g27440	PORB (protochlorophyllide oxidoreductase B); light-dependent NADPH:protochlorophyllide oxidoreductase E	-1.827	0.0167
At4g27580	unknown protein	2.806	2.92E-09
At4g27595	Plant protein of unknown function (DUF827)	-1.152	9.19E-07
At4g27652	unknown protein	3.129	9.40E-11
At4g27657	unknown protein	3.584	8.20E-09
At4g27660	unknown protein	1.585	1.38E-07
At4g27670	HSP21 (heat shock protein 21); chloroplast located small heat shock protein.	11.576	2.40E-11
At4g27680	P-loop containing nucleoside triphosphate hydrolases superfamily protein	1.804	6.60E-10
At4g27790	Calcium-binding EF hand family protein	1.942	2.19E-08
At4g27830	BGLU10 (beta glucosidase 10)	1.423	1.45E-08
At4g27890	HSP20-like chaperones superfamily protein	3.079	6.04E-09
At4g27940	ATMTM1 (ARABIDOPSIS MANGANESE TRACKING FACTOR FOR MITOCHONDRIAL SOD2)	2.742	1.41E-11
At4g27980	Domain of unknown function (DUF3444)	1.12	1.06E-05
At4g28000	P-loop containing nucleoside triphosphate hydrolases superfamily protein	-1.115	2.04E-06
At4g28040	nodulin MtN21 /EamA-like transporter family protein	-2.101	0.0005
At4g28085	unknown protein	1.296	0.0007
At4g28240	Wound-responsive family protein	-1.696	2.36E-07
At4g28270	RMA2 (RING membrane-anchor 2); Encodes a RING finger E3 ubiquitin ligase. Binds and ubiquitinates ABP	-1.11	7.01E-05
At4g28350	Concanavalin A-like lectin protein kinase family protein	4.592	9.33E-12
At4g28380	Leucine-rich repeat (LRR) family protein	1.376	3.48E-06
At4g28390	AAC3 (ADP/ATP carrier 3); Encodes a mitochondrial ADP/ATP carrier protein. Shown in heterologous syster	3.308	2.59E-10
At4g28395	A7 (ANTHER 7); related to lipid transfer proteins	1.617	7.52E-09
At4g28400	Protein phosphatase 2C family protein	2.833	2.99E-11
At4g28460	unknown protein	1.72	4.33E-05
At4g28480	DNAJ heat shock family protein	3.087	2.22E-11
At4g28700	AMT1.4 (ammonium transporter 1.4)	1.166	3.61E-05
At4g28703	RmlC-like cupins superfamily protein	1.253	0.0005
At4g28780	GDSL-like Lipase/Acylhydrolase superfamily protein	-1.429	9.73E-07
At4g29030	Putative membrane lipoprotein	-1.334	5.17E-05
At4g29070	Phospholipase A2 family protein	2.278	7.47E-10
At4g29180	RHS16 (root hair specific 16)	1.523	1.16E-05
At4g29260	HAD superfamily, subfamily IIIB acid phosphatase	-1.031	2.93E-08
At4g29330	DER1 (DERLIN-1)	2.047	8.89E-10
At4g29340	PRF4 (profilin 4); Profilin is a low-molecular weight, actin monomer-binding protein that regulates the organiz	5.889	2.47E-13
At4g29420	F-box/RNI-like superfamily protein	1.374	3.01E-08
At4g29450	Leucine-rich repeat protein kinase family protein	1.091	5.11E-05
At4g29470	Phospholipase A2 family protein	1.816	1.85E-08
At4g29520	LOCATED IN: endoplasmic reticulum, plasma membrane	1.05	5.05E-06
At4g29610	Cytidine/deoxycytidylate deaminase family protein	-1.142	0.00013
At4g29770	unknown protein; Target of trans acting-siR480/255.	3.323	8.26E-10
At4g29780	unknown protein	1.602	0.00032
At4g29800	PLP8 (PATATIN-like protein 8)	1.605	1.08E-07
At4g29905	unknown protein	1.64	5.14E-06
At4g30020	PA-domain containing subtilase family protein	-1.104	1.05E-06
At4g30080	ARF16 (auxin response factor 16); Involved in root cap cell differentiation. Gene expression is regulated by m	-1.059	2.06E-06
At4g30170	Peroxidase family protein	-2.203	4.76E-06
At4g30180	sequence-specific DNA binding transcription factors	-1.707	3.87E-05
At4g30190	AHA2 (H(+)-ATPase 2); belongs to the P-type ATPase superfamily of cation-transporting ATPases, pumps pro	-1.686	2.41E-07
At4g30210	ATR2 (P450 reductase 2); Encodes NADPH-cytochrome P450 reductase that catalyzes the first oxidative step	2.228	2.74E-09
At4g30240	Syntaxin/t-SNARE family protein	1.644	7.79E-08
At4g30280	ATXTH18 (XYLOGLUCAN ENDOTRANSGLUCOSYLASE/HYDROLASE 18); Encodes a xyloglucan endo	1.038	0.00158
At4g30290	ATXTH19 (XYLOGLUCAN ENDOTRANSGLUCOSYLASE/HYDROLASE 19); Encodes a xyloglucan endo	-2.069	2.51E-06
At4g30300	ATNAP15 (ARABIDOPSIS THALIANA NON-INTRINSIC ABC PROTEIN 15); member of NAP subfamily	1.887	1.33E-08
At4g30340	ATDGK7 (diacylglycerol kinase 7); encodes a diacylglycerol kinase. Applying a specific diacylglycerol kinase	1.028	1.13E-06
At4g30390	unknown protein	2.546	5.05E-10
At4g30450	glycine-rich protein	1.09	2.67E-06
At4g30460	glycine-rich protein	2.259	2.26E-07
At4g30490	AFG1-like ATPase family protein	1.425	1.10E-07
At4g30510	ATATG18B (ARABIDOPSIS THALIANA HOMOLOG OF YEAST AUTOPHAGY 18 (ATG18) B)	1.643	1.75E-08
At4g30530	Class I glutamine amidotransferase-like superfamily protein	1.068	4.03E-06
At4g30650	Low temperature and salt responsive protein family	-2.008	7.98E-08

At4g30660	Low temperature and salt responsive protein family	-1.853	1.55E-07
At4g30670	Putative membrane lipoprotein	-1.286	0.00023
At4g30690	Translation initiation factor 3 protein	-1.242	3.48E-07
At4g30710	Family of unknown function (DUF566)	-1.834	1.89E-09
At4g30850	HHP2 (heptahelical transmembrane protein2); heptahelical transmembrane protein homologous to human adi[illegible]	1.95	1.17E-07
At4g31050	Biotin/lipoate A/B protein ligase family	-1.159	1.70E-06
At4g31060	Integrase-type DNA-binding superfamily protein; encodes a member of the DREB subfamily A-5 of ERF/AP2	-1.05	3.79E-06
At4g31248	other RNA; Potential natural antisense gene, locus overlaps with AT4G31250	-1.634	4.29E-06
At4g31398	other RNA; Potential natural antisense gene, locus overlaps with AT4G31400	1.688	3.56E-07
At4g31500	ATR4 (ALTERED TRYPTOPHAN REGULATION 4); Encodes an oxime-metabolizing enzyme in the biosyn[illegible]	-2.091	1.53E-08
At4g31520	SDA1 family protein	1.001	0.00014
At4g31550	WRKY11 (WRKY DNA-binding protein 11); member of WRKY Transcription Factor; Group II-d; negative re[illegible]	1.269	2.54E-06
At4g31615	Transcriptional factor B3 family protein	4.242	1.75E-11
At4g31670	UBP18 (ubiquitin-specific protease 18)	1.572	3.51E-08
At4g31730	GDU1 (glutamine dumper 1); Glutamine dumper1 is a putative transmembrane protein. It is involved in glutar[illegible]	-1.026	0.0006
At4g31820	ENP (ENHANCER OF PINOID); A member of the NPY family genes (NPY1/AT4G31820, NPY2/AT2G1482	-1.528	4.45E-08
At4g31850	PGR3 (proton gradient regulation 3); encodes a protein containing 27 pentatrico-peptide repeat (PPR) motifs	-1.199	9.15E-07
At4g31860	Protein phosphatase 2C family protein	1.599	1.17E-09
At4g31870	ATGPX7 (GLUTATHIONE PEROXIDASE 7); Encodes glutathione peroxidase.	1.126	0.00016
At4g31877	MIR156C (microRNA156C); Encodes a microRNA that targets several SPL family members, including SPL3,	2.637	9.49E-09
At4g31950	CYP82C3 (cytochrome P450, family 82, subfamily C, polypeptide 3); member of CYP82C	2.846	2.05E-06
At4g31970	CYP82C2 (cytochrome P450, family 82, subfamily C, polypeptide 2); member of CYP82C	1.439	3.22E-05
At4g32208	heat shock protein 70 (Hsp 70) family protein	2.403	7.63E-09
At4g32270	Ubiquitin-like superfamily protein	-1.036	5.23E-06
At4g32280	IAA29 (indole-3-acetic acid inducible 29); Auxin inducible protein.	-3.356	2.50E-08
At4g32290	Core-2/I-branching beta-1,6-N-acetylglucosaminyltransferase family protein	-1.181	3.84E-06
At4g32340	Tetratricopeptide repeat (TPR)-like superfamily protein	-3.15	1.76E-09
At4g32460	Protein of unknown function, DUF642	-1.291	7.51E-06
At4g32630	ArfGap/RecO-like zinc finger domain-containing protein	1.176	1.09E-05
At4g32770	ATSDX1 (SUCROSE EXPORT DEFECTIVE 1); Tocopherol cyclase involved in tocopherol (vitamin E)synth[illegible]	2.403	6.06E-09
At4g32810	CCD8 (carotenoid cleavage dioxygenase 8); Encodes a protein with similarity to carotenoid cleaving deoxyger[illegible]	-2.016	0.0002
At4g32880	ATHB-8 (homeobox gene 8); member of homeodomain-leucine zipper family, acting as a differentiation-prom[illegible]	-1.06	5.65E-08
At4g32890	GATA9 (GATA transcription factor 9); Encodes a member of the GATA factor family of zinc finger transcript[illegible]	-1.131	7.15E-08
At4g32990	Transducin/WD40 repeat-like superfamily protein	-1.116	1.55E-06
At4g33040	Thioredoxin superfamily protein	2.401	1.60E-08
At4g33070	Thiamine pyrophosphate dependent pyruvate decarboxylase family protein	1.496	0.03471
At4g33220	ATPME44 (A. THALIANA PECTIN METHYLESTERASE 44)	-1.577	1.41E-08
At4g33440	Pectin lyase-like superfamily protein	2.651	6.51E-10
At4g33490	Eukaryotic aspartyl protease family protein	-1.694	2.44E-08
At4g33625	FUNCTIONS IN: molecular_function unknown; INVOLVED IN: biological_process unknown; LOCATED IN	-1.359	1.32E-08
At4g33660	unknown protein	2.812	1.64E-08
At4g33740	unknown protein	-1.028	6.99E-06
At4g33790	CER4 (ECERIFERUM 4); Encodes an alcohol-forming fatty acyl-CoA reductase, involved in cuticular wax bi[illegible]	-1.115	0.00119
At4g33860	Glycosyl hydrolase family 10 protein	1.418	2.75E-08
At4g33905	Peroxisomal membrane 22 kDa (Mpv17/PMP22) family protein	2.131	1.03E-09
At4g33920	Protein phosphatase 2C family protein	2.469	1.89E-09
At4g33930	Cupredoxin superfamily protein; Encodes a protein with 14.6% glycine residues, similar to hyphally regulated	1.519	0.00014
At4g33940	RING/U-box superfamily protein	1.677	6.48E-08
At4g33950	ATOST1 (OPEN STOMATA 1); Encodes calcium-independent ABA-activated protein kinase, a member of S[illegible]	1.127	7.40E-07
At4g33980	BEST Arabidopsis thaliana protein match is: cold regulated gene 27 (TAIR:AT5G42900.2)	-2.238	7.65E-09
At4g34100	RING/U-box superfamily protein	1.149	1.43E-05
At4g34103	pseudogene of protein binding / zinc ion binding protein; pseudogene of protein binding / zinc ion binding pro	1.032	1.95E-06
At4g34131	UGT73B3 (UDP-glucosyl transferase 73B3)	1.647	4.83E-06
At4g34135	UGT73B2 (UDP-glucosyltransferase 73B2); The At4g34135 gene encodes a flavonol 7-O-glucosyltransferase	1.836	9.22E-06
At4g34150	Calcium-dependent lipid-binding (CaLB domain) family protein	3.733	1.10E-09
At4g34180	Cyclase family protein	2.086	6.10E-10
At4g34240	ALDH3 (aldehyde dehydrogenase 3); Aldehyde dehydrogenase induced by ABA and dehydration	1.018	7.00E-07
At4g34300	Cupredoxin superfamily protein; Encodes protein with 14.7% glycine residues, similar to auxin response facto	2.951	2.09E-08
At4g34310	alpha/beta-Hydrolases superfamily protein	1.185	7.81E-08
At4g34332	other RNA; Potential natural antisense gene, locus overlaps with AT4G34330	1.066	3.24E-06
At4g34390	XLG2 (extra-large GTP-binding protein 2)	1.858	1.69E-07
At4g34440	Protein kinase superfamily protein	1.399	9.55E-09
At4g34460	AGB1 (GTP binding protein beta 1); Encodes the heterotrimeric G-protein beta subunit and is involved in org[illegible]	1.543	6.78E-09
At4g34510	KCS17 (3-ketoacyl-CoA synthase 17); Encodes KCS17, a member of the 3-ketoacyl-CoA synthase family inv[illegible]	1.344	2.76E-07
At4g34560	unknown protein	-1.009	1.19E-05
At4g34630	unknown protein	1.219	1.05E-07
At4g34750	SAUR-like auxin-responsive protein family	-1.313	8.27E-05

At4g34760	SAUR-like auxin-responsive protein family	-1.41	7.66E-06
At4g34770	SAUR-like auxin-responsive protein family	-2.113	1.37E-07
At4g34790	SAUR-like auxin-responsive protein family	-2.08	8.33E-09
At4g34810	SAUR-like auxin-responsive protein family	-1.98	2.84E-06
At4g34850	LAP5 (LESS ADHESIVE POLLEN 5)	1.929	3.82E-09
At4g34880	Amidase family protein	2.211	1.95E-07
At4g34950	Major facilitator superfamily protein	-1.704	8.56E-07
At4g34980	SLP2 (subtilisin-like serine protease 2); Serine protease similar to subtilisin.	-1.363	1.58E-06
At4g35030	Protein kinase superfamily protein	-1.124	1.17E-06
At4g35110	Arabidopsis phospholipase-like protein (PEARLI 4) family	2.516	2.94E-08
At4g35180	LHT7 (LYS/HIS transporter 7)	-1.009	0.01758
At4g35250	NAD(P)-binding Rossmann-fold superfamily protein	1.211	2.18E-07
At4g35270	Plant regulator RWP-RK family protein	-1.018	2.62E-06
At4g35600	CONNEXIN 32 (Protein kinase superfamily protein); plant-type connexin (gap junction-type protein), a compo	1.258	2.20E-07
At4g35640	ATSERAT3.2 (serine acetyltransferase 3.2); Encodes a cytosolic serine O-acetyltransferase involved in sulfur a	2.961	9.02E-09
At4g35690	Arabidopsis protein of unknown function (DUF241)	2.173	7.67E-10
At4g35720	Arabidopsis protein of unknown function (DUF241)	-2.225	5.53E-07
At4g35770	ATSEN1 (ARABIDOPSIS THALIANA SENESCENCE 1); Senescence-associated gene that is strongly induce	-1.638	0.0018
At4g35800	NRPB1 (RNA polymerase II large subunit); Encodes the unique largest subunit of nuclear DNA-dependent RN	1.354	6.56E-05
At4g35860	ATGB2 (GTP-binding 2); GTP-binding protein ATGB2	1.261	6.05E-09
At4g35940	unknown protein	2.466	3.92E-09
At4g35985	Senescence/dehydration-associated protein-related	1.397	3.16E-08
At4g36040	Chaperone DnaJ-domain superfamily protein	-1.392	3.25E-07
At4g36350	ATPAP25 (ARABIDOPSIS THALIANA PURPLE ACID PHOSPHATASE 25)	-1.004	0.00033
At4g36410	UBC17 (ubiquitin-conjugating enzyme 17); ubiquitin-conjugating enzyme	-1.658	2.39E-06
At4g36470	S-adenosyl-L-methionine-dependent methyltransferases superfamily protein	-1.165	1.88E-07
At4g36500	unknown protein	1.25	4.82E-06
At4g36540	BEE2 (BR enhanced expression 2)	-2.216	1.31E-09
At4g36570	ATRL3 (RAD-like 3)	-1.188	4.04E-06
At4g36600	Late embryogenesis abundant (LEA) protein	1.812	1.02E-06
At4g36640	Sec14p-like phosphatidylinositol transfer family protein	1.881	2.42E-08
At4g36670	Major facilitator superfamily protein	-2.244	2.01E-09
At4g36740	ATHB40 (homeobox protein 40); Encodes a homeodomain leucine zipper class I (HD-Zip I) protein.	-1.248	0.0009
At4g36770	UDP-Glycosyltransferase superfamily protein	-1.88	5.72E-07
At4g36850	PQ-loop repeat family protein / transmembrane family protein	-1.126	0.00092
At4g36870	BLH2 (BEL1-like homeodomain 2); Encodes a member of the BEL family of homeodomain proteins. Plants do	-1.13	3.27E-05
At4g36950	MAPKKK21 (mitogen-activated protein kinase kinase kinase 21); member of MEKK subfamily	1.237	0.00759
At4g36988	CPuORF49 (conserved peptide upstream open reading frame 49); Upstream open reading frames (uORFs) are	3.688	7.55E-10
At4g36990	AT-HSFB1 (ARABIDOPSIS THALIANA CLASS B HEAT SHOCK FACTOR B1); encodes a protein whose s	3.987	5.35E-10
At4g37240	unknown protein	-1.139	6.86E-05
At4g37370	CYP81D8 (cytochrome P450, family 81, subfamily D, polypeptide 8); member of CYP81D	3.86	1.94E-09
At4g37400	CYP81F3 (cytochrome P450, family 81, subfamily F, polypeptide 3); member of CYP81F	-1.224	1.33E-05
At4g37450	AGP18 (arabinogalactan protein 18); AGP18 is a lysine-rich arabinogalactan-protein (AGP) and part of a mul	-1.174	4.49E-06
At4g37470	alpha/beta-Hydrolases superfamily protein	-1.742	1.47E-07
At4g37540	LBD39 (LOB domain-containing protein 39)	-1.605	1.75E-08
At4g37610	BT5 (BTB and TAZ domain protein 5); BTB and TAZ domain protein. Located in cytoplasm and expressed in	-1.149	0.0004
At4g37620	similar to RNase H domain-containing protein [Arabidopsis thaliana] (TAIR:AT4G09490.1); similar to 80C09	-1.083	2.45E-05
At4g37640	ACA2 (calcium ATPase 2); Encodes a calmodulin-regulated Ca(2+)-pump located in the endoplasmic reticulu	1.114	1.25E-07
At4g37685	unknown protein	-1.114	5.81E-07
At4g37760	SQE3 (squalene epoxidase 3)	1.24	2.67E-06
At4g37790	HAT22 (Homeobox-leucine zipper protein family); Encodes homeobox protein HAT22, member of the HD-Zip	-1.458	2.91E-07
At4g37890	EDA40 (embryo sac development arrest 40)	-1.095	1.83E-06
At4g37900	Protein of unknown function (duplicated DUF1399)	2.407	2.83E-09
At4g38030	Rhamnogalacturonate lyase family protein	2.66	6.51E-12
At4g38060	unknown protein	1.485	6.12E-07
At4g38540	FAD/NAD(P)-binding oxidoreductase family protein	1.016	0.0019
At4g38560	Arabidopsis phospholipase-like protein (PEARLI 4) family	2.072	3.20E-07
At4g38620	ATMYB4 (myb domain protein 4); Encodes a R2R3 MYB protein which is involved in the response to UV-B.	-1.013	5.89E-05
At4g38730	Protein of unknown function (DUF803)	1.004	2.81E-07
At4g38770	ATPRP4 (ARABIDOPSIS THALIANA PROLINE-RICH PROTEIN 4); Encodes one of four proline-rich prote	-1.177	0.0005
At4g38810	Calcium-binding EF-hand family protein	-1.197	4.16E-06
At4g38825	SAUR-like auxin-responsive protein family	-1.087	0.0003
At4g38840	SAUR-like auxin-responsive protein family	-1.214	2.10E-06
At4g38850	ATSAUR15 (ARABIDOPSIS THALIANA SMALL AUXIN UPREGULATED 15); mRNA is rapidly induced	-2.075	1.48E-07
At4g38860	SAUR-like auxin-responsive protein family	-1.47	5.93E-06
At4g38940	Galactose oxidase/kelch repeat superfamily protein	2.302	2.13E-09
At4g39030	EDS5 (ENHANCED DISEASE SUSCEPTIBILITY 5); Encodes an orphan multidrug and toxin extrusion trans	1.767	8.60E-05

At4g39070	B-box zinc finger family protein	-1.399	1.92E-07
At4g39090	RD19 (RESPONSIVE TO DEHYDRATION 19); Similar to cysteine proteinases, induced by desiccation but n	-1.598	0.0197
At4g39180	ATSEC14 (ARABIDOPSIS THALIANA SECRETION 14); encodes a protein that complements the function c	-1.106	7.19E-07
At4g39230	NmrA-like negative transcriptional regulator family protein; encodes a protein whose sequence is similar to ph	2.505	1.51E-10
At4g39235	unknown protein	2.157	9.00E-11
At4g39250	ATRL1 (RAD-like 1)	-1.23	0.00014
At4g39360	unknown protein	2.325	3.78E-07
At4g39370	UBP27 (ubiquitin-specific protease 27); Encodes a ubiquitin-specific protease.	1.567	6.50E-10
At4g39430	NA	1.286	8.61E-07
At4g39500	CYP96A11 (cytochrome P450, family 96, subfamily A, polypeptide 11); member of CYP96A	2.429	2.59E-07
At4g39520	GTP-binding protein-related; Encodes a member of the DRG (developmentally regulated G-protein) family. H	1.14	6.22E-08
At4g39580	Galactose oxidase/kelch repeat superfamily protein	1.255	9.01E-05
At4g39620	ATPPR5 (A. THALIANA PENTATRICOPEPTIDE REPEAT 5)	-1.072	2.00E-06
At4g39640	GGT1 (gamma-glutamyl transpeptidase 1); The gene encodes a gamma-glutamyltransferase (AKA gamma-glu	2.113	3.44E-09
At4g39670	Glycolipid transfer protein (GLTP) family protein	4.678	3.48E-07
At4g39770	Haloacid dehalogenase-like hydrolase (HAD) superfamily protein	-1.37	1.23E-06
At4g39780	Integrase-type DNA-binding superfamily protein; encodes a member of the DREB subfamily A-6 of ERF/AP2	-1.578	1.66E-07
At4g39890	AtRABH1c (RAB GTPase homolog H1C)	2.862	3.25E-10
At4g39940	AKN2 (APS-kinase 2); adenosine-5'-phosphosulfate-kinase (akn2) mRNA, complete	-2.606	9.93E-10
At4g39950	CYP79B2 (cytochrome P450, family 79, subfamily B, polypeptide 2); Belongs to cytochrome P450 and is invo	-2.929	4.38E-08
At4g40020	Myosin heavy chain-related protein	2.39	6.81E-07
At4g40065	other RNA	-1.657	1.71E-05
At4g40070	RING/U-box superfamily protein	-2.176	2.23E-08
At4g40080	ENTH/ANTH/VHS superfamily protein	1.523	3.44E-06
At5g01060	Protein kinase protein with tetratricopeptide repeat domain	3.681	8.89E-10
At5g01100	O-fucosyltransferase family protein	2.1	2.23E-06
At5g01180	ATPTR5 (ARABIDOPSIS THALIANA PEPTIDE TRANSPORTER 5); Encodes a dipeptide transporter expre	5.427	1.69E-12
At5g01210	HXXXD-type acyl-transferase family protein	-1.51	6.52E-08
At5g01215	other RNA; Potential natural antisense gene, locus overlaps with AT5G01210	-1.209	6.00E-06
At5g01220	SQD2 (sulfoquinovosyldiacylglycerol 2); involved in sulfolipid biosynthesis	-1.275	9.24E-05
At5g01250	alpha 1,4-glycosyltransferase family protein	1.203	1.30E-05
At5g01260	Carbohydrate-binding-like fold	1.325	2.59E-07
At5g01380	Homeodomain-like superfamily protein	1.143	0.00409
At5g01450	RING/U-box superfamily protein	2.212	9.81E-12
At5g01480	Cysteine/Histidine-rich C1 domain family protein	2.017	7.54E-10
At5g01540	LECRKA4.1 (lectin receptor kinase a4.1); Encodes LecRKA4.1, a member of the lectin receptor kinase subfan	1.502	2.33E-05
At5g01700	Protein phosphatase 2C family protein	2.175	8.29E-10
At5g01720	RNI-like superfamily protein	1.47	4.92E-06
At5g01760	ENTH/VHS/GAT family protein	1.011	2.16E-05
At5g01830	ARM repeat superfamily protein	1.266	2.13E-07
At5g01950	Leucine-rich repeat protein kinase family protein	1.359	0.0027
At5g02160	unknown protein	-1.947	2.51E-08
At5g02190	ATASP38 (ARABIDOPSIS THALIANA ASPARTIC PROTEASE 38); encodes an aspartic protease, has an in	-1.36	3.87E-07
At5g02200	FHL (far-red-elongated hypocotyl1-like); Encodes a small plant-specific protein with both nuclear localization	-1.037	8.51E-05
At5g02230	Haloacid dehalogenase-like hydrolase (HAD) superfamily protein	1.581	2.72E-06
At5g02260	ATEXPA9 (expansin A9); member of Alpha-Expansin Gene Family. Naming convention from the Expansin W	-1.843	4.94E-08
At5g02290	NAK (Protein kinase superfamily protein); Encodes a candidate protein kinase NAK that is similar to the onco	1.118	4.42E-05
At5g02410	DIE2/ALG10 family	-1.116	3.89E-07
At5g02430	Transducin/WD40 repeat-like superfamily protein	1.936	2.72E-10
At5g02480	HSP20-like chaperones superfamily protein	-1.569	6.55E-07
At5g02490	Heat shock protein 70 (Hsp 70) family protein	3.38	8.02E-08
At5g02640	unknown protein	-1.126	7.07E-07
At5g02750	RING/U-box superfamily protein	-1.003	6.68E-06
At5g02760	Protein phosphatase 2C family protein	-2.291	3.45E-09
At5g02780	GSTL1 (glutathione transferase lambda 1); Encodes a member of the lambda family of glutathione transferases	2.309	0.00125
At5g02800	Protein kinase superfamily protein	1.276	1.11E-08
At5g02820	BIN5 (BRASSINOSTEROID INSENSITIVE 5); Involved in the patterning and shape of leaf trichomes. Encod	1.308	2.93E-07
At5g02840	LCL1 (LHY/CCA1-like 1); CCA1 and LHY colocalize in the nucleus and form heterodimers in vivo. CCA1 ar	1.272	4.30E-07
At5g02890	HXXXD-type acyl-transferase family protein	-1.071	1.49E-05
At5g03010	Galactose oxidase/kelch repeat superfamily protein	2.577	4.20E-11
At5g03020	Galactose oxidase/kelch repeat superfamily protein	1.048	1.02E-05
At5g03030	Chaperone DnaJ-domain superfamily protein	1.304	5.45E-07
At5g03120	unknown protein	-1.407	2.82E-05
At5g03160	ATP58IPK (homolog of mamallian P58IPK); J domain protein localized in ER lumen. Can partially compensat	-1.046	1.58E-05
At5g03210	unknown protein	3.985	6.46E-08
At5g03230	Protein of unknown function, DUF584	-1.249	6.55E-07
At5g03285	other RNA	2.603	1.61E-10

At5g03330	Cysteine proteinases superfamily protein	1.172	5.17E-08
At5g03340	ATPase, AAA-type, CDC48 protein	2.603	5.20E-10
At5g03370	acylphosphatase family	1.465	1.29E-07
At5g03380	Heavy metal transport/detoxification superfamily protein	1.858	2.07E-07
At5g03406	Class II aaRS and biotin synthetases superfamily protein	1.111	2.61E-07
At5g03435	Ca2+dependent plant phosphoribosyltransferase family protein (Ca2+dependent plant phosphoribosyltransfera	3.588	1.50E-11
At5g03490	UDP-Glycosyltransferase superfamily protein	1.175	6.61E-06
At5g03495	RNA-binding (RRM/RBD/RNP motifs) family protein	2.066	3.14E-08
At5g03552	MIR822A (microRNA822A); Encodes a microRNA of unknown function. MicroRNAs are regulatory RNAs w	-1.856	2.10E-08
At5g03555	permease, cytosine/purines, uracil, thiamine, allantoin family protein	1.248	7.15E-07
At5g03560	Tetratricopeptide repeat (TPR)-like superfamily protein	1.352	1.48E-07
At5g03610	GDSL-like Lipase/Acylhydrolase superfamily protein	1.2	3.02E-07
At5g03630	ATMDAR2 (Pyridine nucleotide-disulphide oxidoreductase family protein)	1.046	0.0002
At5g03740	HD2C (histone deacetylase 2C); HD2-type histone deacetylase HDAC. Involved in the ABA and stress respon	1.308	5.42E-06
At5g03960	IQD12 (IQ-domain 12)	-1.147	1.92E-05
At5g03990	unknown protein	3.662	5.06E-11
At5g03995	unknown protein	-1.352	9.06E-08
At5g04190	PKS4 (phytochrome kinase substrate 4); Encodes phytochrome kinase substrate 4, a phytochrome signaling co	-1.339	1.33E-05
At5g04220	SYT3 (synaptotagmin 3)	-1.282	1.29E-06
At5g04230	PAL3 (phenyl alanine ammonia-lyase 3); Member of Phenylalanine ammonialyase (PAL) gene family. Differs	-1.944	2.83E-07
At5g04340	CZF2 (COLD INDUCED ZINC FINGER PROTEIN 2); putative c2h2 zinc finger transcription factor mRNA,	2.77	3.90E-09
At5g04360	ATLDA (limit dextrinase); Encodes an enzyme thought to be involved in the hydrolysis of the α-1,6 link	-1.12	1.06E-07
At5g04410	anac078 (Arabidopsis NAC domain containing protein 78); NAC family member, functions as a transcriptiona	1.945	8.79E-09
At5g04610	S-adenosyl-L-methionine-dependent methyltransferases superfamily protein	3.083	1.27E-10
At5g04720	ADR1-L2 (ADR1-like 2)	1.14	0.0003
At5g04750	F1F0-ATPase inhibitor protein, putative	1.383	0.0003
At5g04770	ATCAT6 (ARABIDOPSIS THALIANA CATIONIC AMINO ACID TRANSPORTER 6); Encodes a member (	-1.116	2.13E-06
At5g04830	Nuclear transport factor 2 (NTF2) family protein	1.096	5.53E-08
At5g04840	bZIP protein	2.853	1.58E-11
At5g04890	RTM2 (RESTRICTED TEV MOVEMENT 2); Specifically restricts the long-distance movement of tobacco etc	1.194	8.98E-06
At5g04930	ALA1 (aminophospholipid ATPase 1); Encodes a putative aminophospholipid translocase (p-type ATPase) inv	1.456	2.64E-06
At5g05090	Homeodomain-like superfamily protein	-1.247	1.19E-07
At5g05160	Leucine-rich repeat protein kinase family protein	-1.023	2.13E-05
At5g05220	unknown protein	7.694	1.74E-13
At5g05270	Chalcone-flavanone isomerase family protein	1.045	0.01395
At5g05300	unknown protein	2.753	2.90E-06
At5g05340	Peroxidase superfamily protein	-1.286	0.00863
At5g05410	DREB2 (DEHYDRATION-RESPONSIVE ELEMENT BINDING PROTEIN 2); Encodes a transcription factor	5.06	3.57E-11
At5g05420	FKBP-like peptidyl-prolyl cis-trans isomerase family protein	-1.842	2.87E-07
At5g05480	Peptide-N4-(N-acetyl-beta-glucosaminyl)asparagine amidase A protein	1.296	8.02E-08
At5g05490	DIF1 (DETERMINATE, INFERTILE 1); Encodes a RAD21-like gene essential for meiosis. Encodes a 627 a.a	2.939	1.52E-08
At5g05850	PIRL1 (plant intracellular ras group-related LRR 1); Encodes PIRL1, a member of the Plant Intracellular Ras-g	1.72	4.02E-08
At5g05890	UDP-Glycosyltransferase superfamily protein	-1.466	1.38E-05
At5g05960	Bifunctional inhibitor/lipid-transfer protein/seed storage 2S albumin superfamily protein	-1.032	6.27E-05
At5g06250	AP2/B3-like transcriptional factor family protein	1.236	1.73E-05
At5g06278	pseudogene of abscisic acid-responsive HVA22 family protein; pseudogene of abscisic acid-responsive HVA2	-1.023	7.81E-05
At5g06280	unknown protein	1.199	6.54E-07
At5g06320	NHL3 (NDR1/HIN1-like 3); encodes a protein whose sequence is similar to tobacco hairpin-induced gene (HI	1.602	6.96E-06
At5g06470	Glutaredoxin family protein	-1.574	8.72E-07
At5g06480	Immunoglobulin E-set superfamily protein	-1.15	3.42E-06
At5g06570	alpha/beta-Hydrolases superfamily protein	-1.422	1.02E-05
At5g06690	WCRKC1 (WCRKC thioredoxin 1); Encodes a thioredoxin (WCRKC1) localized in chloroplast stroma. Conta	-2.342	1.12E-07
At5g06720	ATPA2 (peroxidase 2)	-1.176	6.81E-05
At5g06750	Protein phosphatase 2C family protein	1.823	8.45E-10
At5g06800	myb-like HTH transcriptional regulator family protein	-1.285	2.80E-07
At5g06820	SRF2 (STRUBBELIG-receptor family 2)	5.051	2.50E-12
At5g06850	C2 calcium/lipid-binding plant phosphoribosyltransferase family protein	-1.222	1.76E-06
At5g06870	ATPGIP2 (ARABIDOPSIS POLYGALACTURONASE INHIBITING PROTEIN 2); Encodes a polygalacturon	-1.336	2.30E-05
At5g06960	OBF5 (OCS-element binding factor 5); Encodes a basic leucine zipper (B-ZIP) containing protein that interact	1.474	4.48E-08
At5g06980	unknown protein	1.489	4.52E-07
At5g06990	Protein of unknown function, DUF617	-1.059	0.00178
At5g07100	WRKY26 (WRKY DNA-binding protein 26); Encodes WRKY DNA-binding protein 26 (WRKY26).	-1.366	5.25E-06
At5g07110	PRA1.B6 (prenylated RAB acceptor 1.B6)	-1.462	3.90E-07
At5g07330	unknown protein	6.73	3.78E-14
At5g07560	GRP20 (glycine-rich protein 20); Lipid-binding oleosins, glycine-rich protein.	-1.127	1.41E-05
At5g07670	RNI-like superfamily protein	-1.047	5.76E-07
At5g07690	ATMYB29 (myb domain protein 29); Encodes a putative transcription factor (MYB29).	-3.65	1.70E-09

At5g07700	AtMYB76 (myb domain protein 76); Encodes a putative transcription factor (MYB76).	-2.531	3.49E-08
At5g07850	HXXXD-type acyl-transferase family protein	-1.007	0.00282
At5g08010	unknown protein	-1.149	1.68E-07
At5g08030	PLC-like phosphodiesterases superfamily protein	-2.179	0.00016
At5g08139	RING/U-box superfamily protein	1.508	1.18E-07
At5g08350	GRAM domain-containing protein / ABA-responsive protein-related	-1.492	2.01E-06
At5g08380	AGAL1 (alpha-galactosidase 1)	1.074	3.15E-08
At5g08440	unknown protein	1.096	7.64E-07
At5g08520	Duplicated homeodomain-like superfamily protein	-1.042	7.21E-08
At5g08695	RNA-binding (RRM/RBD/RNP motifs) family protein	1.009	4.56E-06
At5g08790	anac081 (Arabidopsis NAC domain containing protein 81); induced by wounding, belongs to a large family of	2.29	2.83E-08
At5g09280	Pectin lyase-like superfamily protein	1.445	6.55E-07
At5g09430	alpha/beta-Hydrolases superfamily protein	2.32	5.29E-08
At5g09590	HSC70-5 (HEAT SHOCK COGNATE); heat shock protein 70 (Hsc70-5); nuclear	2.209	2.39E-09
At5g09610	APUM21 (pumilio 21); Encodes a member of the Arabidopsis Pumilio (APUM) proteins containing PUF dom	1.773	2.66E-08
At5g09730	ATBXL3 (BETA-XYLOSIDASE 3); encodes a protein similar to a beta-xylosidase located in the extracellular	-1.997	3.69E-06
At5g09800	ARM repeat superfamily protein	1.708	3.44E-07
At5g09805	IDL3 (inflorescence deficient in abscission (IDA)-like 3); Similar to Inflorescence deficient in abscission (IDA	-1.152	3.57E-07
At5g09930	ATGCN2 (ARABIDOPSIS THALIANA GENERAL CONTROL NON-REPRESSIBLE 2); member of GCN su	6.683	4.02E-12
At5g09940	Protein of unknown function (DUF1635)	1.65	4.04E-06
At5g09990	PROPEP5 (elicitor peptide 5 precursor)	1.644	1.90E-08
At5g10000	ATFD4 (ferredoxin 4)	1.543	2.10E-07
At5g10180	AST68 (ARABIDOPSIS SULFATE TRANSPORTER 68); Encodes a low-affinity sulfate transporter expresse	-1.048	6.60E-06
At5g10190	Major facilitator superfamily protein	1.396	9.49E-09
At5g10336	unknown protein	2.692	1.85E-10
At5g10340	F-box family protein	1.633	8.08E-08
At5g10390	Histone superfamily protein	-1.259	2.05E-05
At5g10400	Histone superfamily protein	-1.217	1.54E-05
At5g10410	ENTH/ANTH/VHS superfamily protein	1.339	3.18E-07
At5g10430	AGP4 (arabinogalactan protein 4); Encodes arabinogalactan-protein (AGP4).	-1.283	7.81E-05
At5g10530	Concanavalin A-like lectin protein kinase family protein	-1.043	1.30E-05
At5g10570	basic helix-loop-helix (bHLH) DNA-binding superfamily protein	-2.19	8.24E-09
At5g10695	unknown protein	5.811	2.16E-11
At5g10760	Eukaryotic aspartyl protease family protein	-2.616	0.0007
At5g10800	RNA recognition motif (RRM)-containing protein	1.436	7.83E-09
At5g10830	S-adenosyl-L-methionine-dependent methyltransferases superfamily protein	1.371	1.74E-08
At5g10880	tRNA synthetase-related / tRNA ligase-related	2.898	6.46E-12
At5g10900	Calcineurin-like metallo-phosphoesterase superfamily protein	1.276	3.21E-07
At5g11020	Protein kinase superfamily protein	1.028	6.59E-06
At5g11050	AtMYB64 (myb domain protein 64); Member of R2R3-MYB transcription factor gene family.	1.564	7.11E-10
At5g11070	unknown protein	-1.464	1.20E-05
At5g11100	SYT4 (synaptotagmin 4)	3.521	1.38E-10
At5g11140	Arabidopsis phospholipase-like protein (PEARLI 4) family	4.452	1.89E-12
At5g11150	ATVAMP713 (vesicle-associated membrane protein 713); Member of Synaptobrevin-like AtVAMP7C, v-SNA	-1.426	1.98E-07
At5g11260	HY5 (ELONGATED HYPOCOTYL 5); Basic leucine zipper (bZIP) transcription factor. Nuclear localization.	1.283	1.26E-06
At5g11340	Acyl-CoA N-acyltransferases (NAT) superfamily protein	1.314	3.90E-07
At5g11400	Protein kinase superfamily protein	1.337	6.23E-08
At5g11410	Protein kinase superfamily protein	1.309	1.08E-06
At5g11420	Protein of unknown function, DUF642	-1.211	0.0011
At5g11610	Exostosin family protein	-1.856	1.43E-08
At5g11650	alpha/beta-Hydrolases superfamily protein	2.351	1.70E-09
At5g11680	FUNCTIONS IN: molecular_function unknown; INVOLVED IN: biological_process unknown; LOCATED IN	3.319	1.54E-11
At5g11930	Thioredoxin superfamily protein	2.044	6.86E-08
At5g11970	Protein of unknown function (DUF3511)	1.17	6.68E-07
At5g12000	Protein kinase protein with adenine nucleotide alpha hydrolases-like domain	2.861	1.79E-08
At5g12020	HSP17.6II (17.6 kDa class II heat shock protein)	10.167	8.33E-12
At5g12030	AT-HSP17.6A (heat shock protein 17.6A); Encodes a cytosolic small heat shock protein with chaperone activi	9.592	1.80E-11
At5g12040	Nitrilase/cyanide hydratase and apolipoprotein N-acyltransferase family protein	1.961	3.11E-10
At5g12050	unknown protein	-1.091	5.17E-05
At5g12110	Glutathione S-transferase, C-terminal-like	4.15	1.15E-11
At5g12170	CLT3 (CRT (chloroquine-resistance transporter)-like transporter 3); Encodes one of the CRT-Like transporters	-1.077	5.56E-05
At5g12250	TUB6 (beta-6 tubulin); Encodes a beta-tubulin. Expression of TUB6 has been shown to decrease in response to	-1.038	3.21E-05
At5g12270	2-oxoglutarate (2OG) and Fe(II)-dependent oxygenase superfamily protein	2.46	5.25E-09
At5g12280	SWAP (Suppressor-of-White-APricot)/surp RNA-binding domain-containing protein	3.057	4.83E-11
At5g12290	DGS1 (DGD1 SUPPRESSOR 1); Encodes a mitochondrial outer membrane protein, involved in galactoglycero	1.278	7.93E-06
At5g12910	Histone superfamily protein	-1.152	5.52E-05
At5g12930	unknown protein	2.088	1.63E-09

At5g12950	Putative glycosyl hydrolase of unknown function (DUF1680)	-1.43	9.55E-09
At5g13100	unknown protein	1.428	5.26E-09
At5g13130	Histidine kinase-, DNA gyrase B-, and HSP90-like ATPase family protein	1.333	3.86E-06
At5g13170	SAG29 (senescence-associated gene 29)	3.063	3.00E-07
At5g13190	CONTAINS InterPro DOMAIN/s: LPS-induced tumor necrosis factor alpha factor (InterPro:IPR006629); Has	3.288	5.49E-10
At5g13200	GRAM domain family protein	3.809	1.95E-09
At5g13210	Uncharacterised conserved protein UCP015417, vWA	1.899	4.86E-05
At5g13220	JAS1 (JASMONATE-ASSOCIATED 1); Plants overexpressing At5g13220.3, but not At5g13220.1 showed en	2.5	4.71E-06
At5g13330	Rap2.6L (related to AP2 6l); encodes a member of the ERF (ethylene response factor) subfamily B-4 of ERF/A	2.592	3.39E-07
At5g13370	Auxin-responsive GH3 family protein	2.027	1.10E-09
At5g13490	AAC2 (ADP/ATP carrier 2); Encodes mitochondrial ADP/ATP carrier	2.224	2.51E-07
At5g13550	SULTR4.1 (sulfate transporter 4.1); Encodes a sulfate transporter.	1.643	6.05E-09
At5g13700	ATPAO1 (polyamine oxidase 1); Encodes a protein with polyamine oxidase activity. The mRNA of this gene i	1.298	3.28E-06
At5g13750	ZIFL1 (zinc induced facilitator-like 1)	1.053	2.93E-06
At5g14020	Endosomal targeting BRO1-like domain-containing protein	3.77	2.72E-11
At5g14200	ATIMD1 (ARABIDOPSIS ISOPROPYLMALATE DEHYDROGENASE 1); The AtIMD1 is one out of 3 gene	-1.962	2.60E-07
At5g14250	COP13 (CONSTITUTIVE PHOTOMORPHOGENIC 13); Encodes subunit 3 of the COP9 signalosome.	1.039	4.85E-06
At5g14310	AtCXE16 (carboxyesterase 16)	1.145	3.08E-07
At5g14350	Pentatricopeptide repeat (PPR) superfamily protein	-1.032	3.00E-05
At5g14360	Ubiquitin-like superfamily protein	-1.492	8.64E-08
At5g14550	Core-2/I-branching beta-1,6-N-acetylglucosaminyltransferase family protein	-1.077	2.22E-07
At5g14602	FUNCTIONS IN: molecular_function unknown; INVOLVED IN: biological_process unknown; LOCATED IN	1.495	2.94E-07
At5g14640	ATSK13 (SHAGGY-LIKE KINASE 13)	1.341	3.19E-07
At5g14700	NAD(P)-binding Rossmann-fold superfamily protein	1.493	1.93E-05
At5g14740	BETA CA2 (BETA CARBONIC ANHYDRASE 2); Encodes a beta carbonic anhydrase likely to be localized i	1.676	2.37E-07
At5g14760	AO (L-aspartate oxidase); At5g14760 encodes for L-aspartate oxidase involved in the early steps of NAD bios	1.112	0.0009
At5g14920	Gibberellin-regulated family protein	-1.863	1.88E-06
At5g14940	Major facilitator superfamily protein	-1.958	1.86E-08
At5g15090	ATVDAC3 (ARABIDOPSIS THALIANA VOLTAGE DEPENDENT ANION CHANNEL 3); Encodes a volta	1.23	6.90E-07
At5g15230	GASA4 (GAST1 protein homolog 4); gibberellin-regulated (GASA4)	-1.465	7.39E-07
At5g15250	FTSH6 (FTSH protease 6); Encodes an FtsH protease that is localized to the chloroplast. AtFtsH6 is involved i	7.79	2.06E-15
At5g15254	unknown protein	1.173	2.89E-07
At5g15280	Pentatricopeptide repeat (PPR) superfamily protein	-1.005	2.40E-06
At5g15310	ATMYB16 (myb domain protein 16); Member of the R2R3 factor gene family.	-1.011	2.76E-05
At5g15410	ATCNGC2 (CYCLIC NUCLEOTIDE-GATED CHANNEL 2); 'defense, no death' gene (DND1) encodes a mu	-1.346	3.75E-07
At5g15450	APG6 (ALBINO AND PALE GREEN 6); Encodes a chloroplast-targeted Hsp101 homologue. Functions as a	2.865	6.62E-11
At5g15480	C2H2-type zinc finger family protein	1.751	1.50E-09
At5g15500	Ankyrin repeat family protein	1.121	0.00264
At5g15600	SP1L4 (SPIRAL1-like4); SPIRAL1-LIKE4 belongs to a six-member gene family in Arabidopsis; all members s	1.972	2.91E-07
At5g15725	unknown protein	1.535	2.17E-05
At5g15830	AtbZIP3 (basic leucine-zipper 3)	-1.575	4.80E-08
At5g15840	CO (CONSTANS); Encodes a protein showing similarities to zinc finger transcription factors, involved in regu	-1.868	8.27E-08
At5g15845	other RNA; Potential natural antisense gene, locus overlaps with AT5G15850	1.575	1.32E-07
At5g15850	COL1 (CONSTANS-like 1); Homologous to the flowering-time gene CONSTANS.	1.637	9.71E-07
At5g15860	ATPCME (prenylcysteine methylesterase); Encodes a protein with prenylcysteine methylesterase activity.	1.519	5.75E-08
At5g15870	glycosyl hydrolase family 81 protein	1.37	3.64E-07
At5g15940	NAD(P)-binding Rossmann-fold superfamily protein	1.956	2.36E-08
At5g15970	COR6.6 (COLD-RESPONSIVE 6.6); Encodes a gene that can be induced by cold and abscisic acid and may b	-1.783	1.08E-06
At5g16020	GEX3 (gamete-expressed 3); Encodes GEX3, a plasma membrane localized protein expressed in the male gam	2.275	3.29E-08
At5g16030	unknown protein	-1.07	5.00E-07
At5g16080	AtCXE17 (carboxyesterase 17)	1.393	4.80E-05
At5g16190	ATCSLA11 (ARABIDOPSIS THALIANA CELLULOSE SYNTHASE LIKE A11); encodes a gene similar to	-1.086	3.79E-05
At5g16200	50S ribosomal protein-related	2.583	9.76E-12
At5g16260	ELF9 (EARLY FLOWERING 9); Encodes a RNA binding protein ELF9 (EARLY FLOWERING9). Loss of E	1.2	1.73E-08
At5g16530	PIN5 (PIN-FORMED 5); Encodes PIN5, an atypical member of the PIN family. PIN5 is a functional auxin tra	-1.668	3.64E-07
At5g16570	GLN1.4 (glutamine synthetase 1.4); Encodes a cytosolic glutamine synthetase, the enzyme has high affinity wi	-1.071	0.0003
At5g16830	SYP21 (syntaxin of plants 21); member of SYP2 Gene Family. Over-expression of the gene in tobacco protop	1.386	1.03E-08
At5g16900	Leucine-rich repeat protein kinase family protein	3.498	5.04E-10
At5g16960	Zinc-binding dehydrogenase family protein	2.733	4.28E-10
At5g16970	AER (alkenal reductase); encodes a 2-alkenal reductase (EC 1.3.1.74), plays a key role in the detoxification of	2.008	5.13E-08
At5g16980	Zinc-binding dehydrogenase family protein	2.379	4.70E-08
At5g17000	Zinc-binding dehydrogenase family protein	2.334	3.24E-09
At5g17050	UGT78D2 (UDP-glucosyl transferase 78D2); The At5g17050 encodes a anthocyanidin 3-O-glucosyltransfera	1.208	0.00013
At5g17170	ENH1 (enhancer of sos3-1)	1.138	7.37E-05
At5g17270	Protein prenylyltransferase superfamily protein	1.274	2.68E-06
At5g17300	RVE1 (REVEILLE 1); Myb-like transcription factor that regulates hypocotyl growth by regulating free auxin l	3.606	3.99E-10
At5g17310	AtUGP2 (UDP-GLUCOSE PYROPHOSPHORYLASE 2)	2.115	4.32E-08

At5g17340	Putative membrane lipoprotein	-2.972	6.61E-05
At5g17350	unknown protein	1.56	6.09E-05
At5g17370	Transducin/WD40 repeat-like superfamily protein	1.503	2.21E-08
At5g17380	Thiamine pyrophosphate dependent pyruvate decarboxylase family protein	1.968	2.22E-07
At5g17390	Adenine nucleotide alpha hydrolases-like superfamily protein	2.472	8.83E-07
At5g17400	ER-ANT1 (endoplasmic reticulum-adenine nucleotide transporter 1); This gene is predicted to encode an ER-l	1.704	5.56E-08
At5g17410	Spc97 / Spc98 family of spindle pole body (SBP) component	1.453	1.35E-09
At5g17460	unknown protein	-1.053	0.0015
At5g17680	disease resistance protein (TIR-NBS-LRR class), putative	1.022	2.84E-06
At5g17780	alpha/beta-Hydrolases superfamily protein	1.107	3.07E-06
At5g18010	SAUR-like auxin-responsive protein family	-2.13	3.59E-08
At5g18020	SAUR-like auxin-responsive protein family	-1.961	2.54E-07
At5g18030	SAUR-like auxin-responsive protein family	-2.044	1.09E-07
At5g18040	unknown protein	1.172	2.25E-05
At5g18050	SAUR-like auxin-responsive protein family	-2.091	3.14E-08
At5g18060	SAUR-like auxin-responsive protein family	-2.076	2.20E-08
At5g18065	unknown protein	4.149	2.16E-11
At5g18080	SAUR-like auxin-responsive protein family	-2.078	5.20E-08
At5g18150	Methyltransferase-related protein	1.244	2.85E-07
At5g18180	H/ACA ribonucleoprotein complex, subunit Gar1/Naf1 protein	3.054	2.62E-11
At5g18290	SIP1B (SMALL AND BASIC INTRINSIC PROTEIN 1B); Belongs to a family of plant aquaporins.Similar to y	2.574	2.71E-09
At5g18330	ARM repeat superfamily protein	1.138	0.00309
At5g18340	ARM repeat superfamily protein	4.851	1.24E-12
At5g18350	Disease resistance protein (TIR-NBS-LRR class) family	1.373	1.63E-05
At5g18400	Cytokine-induced anti-apoptosis inhibitor 1, Fe-S biogenesis	2.361	4.31E-08
At5g18420	unknown protein	1.479	1.89E-09
At5g18430	GDSL-like Lipase/Acylhydrolase superfamily protein	-1.193	0.00031
At5g18460	Protein of Unknown Function (DUF239)	-1.123	3.50E-07
At5g18480	PGSIP6 (plant glycogenin-like starch initiation protein 6)	1.262	1.04E-08
At5g18490	Plant protein of unknown function (DUF946)	2.766	1.10E-11
At5g18600	Thioredoxin superfamily protein	-1.744	8.89E-07
At5g18930	BUD2 (BUSHY AND DWARF 2)	-1.23	7.08E-08
At5g19000	ATBPM1 (BTB-POZ AND MATH DOMAIN 1)	1.241	9.53E-09
At5g19080	RING/U-box superfamily protein	1.314	0.0013
At5g19190	unknown protein	-1.855	4.23E-08
At5g19240	Glycoprotein membrane precursor GPI-anchored	1.188	0.0061
At5g19470	atnudt24 (nudix hydrolase homolog 24)	1.608	0.00025
At5g19480	unknown protein	1.441	3.16E-08
At5g19600	SULTR3.5 (sulfate transporter 3.5); Encodes sulfate transporter Sultr3;5.	-1.627	5.36E-08
At5g19850	alpha/beta-Hydrolases superfamily protein	1.658	3.70E-09
At5g19855	Chaperonin-like RbcX protein	1.556	8.63E-09
At5g19875	unknown protein	4.231	4.45E-12
At5g19970	unknown protein	-1.11	3.46E-06
At5g20000	AAA-type ATPase family protein	1.712	4.52E-08
At5g20030	Plant Tudor-like RNA-binding protein	-1.159	1.18E-06
At5g20110	Dynein light chain type 1 family protein	-1.343	1.78E-06
At5g20150	ATSPX1 (ARABIDOPSIS THALIANA SPX DOMAIN GENE 1)	-1.095	0.0004
At5g20225	other RNA; Potential natural antisense gene, locus overlaps with AT5G20220	2.513	5.69E-09
At5g20370	serine-rich protein-related	2.219	7.17E-07
At5g20380	PHT4.5 (phosphate transporter 4.5); Encodes an inorganic phosphate transporter (PHT4;5).	1.401	5.36E-06
At5g20410	ATMGD2 (ARABIDOPSIS THALIANA MONOGALACTOSYLDIACYLGLYCEROL SYNTHASE 2); Enco	-1.115	0.00339
At5g20630	ATGER3 (ARABIDOPSIS THALIANA GERMIN 3); Encodes a germin-like protein. Its transcripts are more a	-1.203	0.0002
At5g20640	Protein of unknown function (DUF567)	-1.002	0.00012
At5g20700	Protein of unknown function (DUF581)	-1.203	0.0001
At5g20790	unknown protein	-2.408	3.37E-05
At5g20820	SAUR-like auxin-responsive protein family	-1.387	6.75E-06
At5g20910	AIP2 (ABI3-interacting protein 2)	1.957	3.53E-09
At5g20935	unknown protein	-1.093	5.13E-05
At5g21105	Plant L-ascorbate oxidase	-1.175	4.52E-07
At5g21430	Chaperone DnaJ-domain superfamily protein	1.061	0.0003
At5g21900	RNI-like superfamily protein	2.17	3.84E-09
At5g21940	unknown protein	-1.04	4.94E-05
At5g22000	RHF2A (RING-H2 group F2A); encodes a RING-type E3 ubiquitin ligase implicated in gametogenesis. Doubl	1.494	2.57E-07
At5g22060	ATJ2 (ARABIDOPSIS THALIANA DNAJ HOMOLOGUE 2); Co-chaperonin similar to E. coli DnaJ	2.789	3.49E-10
At5g22090	Protein of unknown function (DUF3049)	-1.28	7.47E-06
At5g22140	FAD/NAD(P)-binding oxidoreductase family protein	2.054	2.21E-09
At5g22220	E2F1 (E2F transcription factor 1); Member of the E2F transcription factors, (cell cycle genes), key components	1.125	5.34E-08

At5g22310	unknown protein	-1.67	3.26E-07
At5g22410	RHS18 (root hair specific 18)	1.759	4.75E-06
At5g22520	unknown protein	1.213	0.002
At5g22530	unknown protein	3.317	5.42E-07
At5g22540	Plant protein of unknown function (DUF247)	2.666	7.68E-06
At5g22570	ATWRKY38 (ARABIDOPSIS THALIANA WRKY DNA-BINDING PROTEIN 38); member of WRKY Trans	-1.808	0.0042
At5g22580	Stress responsive A/B Barrel Domain	-1.382	6.61E-07
At5g22660	FBD, F-box, Skp2-like and Leucine Rich Repeat domains containing protein	3.473	1.77E-10
At5g22670	F-box/RNI-like/FBD-like domains-containing protein	5.223	1.31E-14
At5g22680	FUNCTIONS IN: molecular_function unknown; INVOLVED IN: biological_process unknown; LOCATED IN	3.617	2.38E-06
At5g22690	Disease resistance protein (TIR-NBS-LRR class) family	3.396	5.91E-12
At5g22720	F-box/RNI-like superfamily protein	1.128	4.07E-07
At5g22860	Serine carboxypeptidase S28 family protein	-1.239	3.12E-06
At5g22880	H2B (HISTONE H2B); Encodes a histone 2B (H2B) protein. This protein can be ubiquitinated in planta, and t	-1.182	0.00013
At5g22920	CHY-type/CTCHY-type/RING-type Zinc finger protein	-1.999	3.81E-08
At5g22930	Protein of unknown function (DUF1635)	-1.351	3.28E-06
At5g23010	IMS3 (2-ISOPROPYLMALATE SYNTHASE 3); Encodes a methylthioalkylmalate synthase, catalyzes the cor	-2.61	1.03E-10
At5g23020	IMS2 (2-isopropylmalate synthase 2); methylthioalkymalate synthase-like. Also known as 2-isopropylmalate sy	-1.994	1.65E-07
At5g23210	SCPL34 (serine carboxypeptidase-like 34)	-2.111	2.00E-07
At5g23240	DNAJ heat shock N-terminal domain-containing protein	-3.603	1.77E-09
At5g23350	GRAM domain-containing protein / ABA-responsive protein-related	-1.009	7.72E-05
At5g23360	GRAM domain-containing protein / ABA-responsive protein-related	-1.202	7.77E-06
At5g23420	HMGB6 (high-mobility group box 6); Encodes HMGB6, a protein belonging to the subgroup of HMGB (high	-1.001	6.14E-05
At5g23425	NA	-1.233	0.00425
At5g23460	unknown protein	-1.49	8.03E-09
At5g23480	SWIB/MDM2 domain	2.387	4.75E-09
At5g23510	unknown protein	1.713	1.59E-07
At5g23600	RNA 2'-phosphotransferase, Tpt1 / KptA family	1.396	6.83E-09
At5g23660	MTN3 (homolog of Medicago truncatula MTN3); homolog of the Medicago nodulin MTN3	-2.159	1.62E-06
At5g23710	DNA binding	1.099	2.72E-06
At5g23870	Pectinacetylesterase family protein	-1.938	9.80E-09
At5g23980	FRO4 (ferric reduction oxidase 4); Encodes a ferric chelate reductase that is expressed at low levels in roots,sh	-2.744	5.54E-09
At5g24070	Peroxidase superfamily protein	1.131	8.68E-06
At5g24110	ATWRKY30 (ARABIDOPSIS THALIANA WRKY DNA-BINDING PROTEIN 30); member of WRKY Trans	5.431	4.19E-11
At5g24120	ATSIG5 (SIGMA FACTOR 5); Encodes a specialized sigma factor that functions in regulation of plastid genes	2.609	8.70E-09
At5g24200	alpha/beta-Hydrolases superfamily protein	-3.128	0.00024
At5g24390	Ypt/Rab-GAP domain of gyp1p superfamily protein	1.37	9.26E-07
At5g24470	APRR5 (pseudo-response regulator 5); Encodes a pseudo-response regulator whose mutation affects various ci	-1.186	0.00044
At5g24490	30S ribosomal protein, putative	-1.201	6.68E-07
At5g24530	DMR6 (DOWNY MILDEW RESISTANT 6); Encodes a putative 2OG-Fe(II) oxygenase that is defense-associa	-1.035	0.0184
At5g24570	unknown protein	-1.152	1.26E-06
At5g24580	Heavy metal transport/detoxification superfamily protein	-1.323	8.27E-07
At5g24590	ANAC091 (Arabidopsis NAC domain containing protein 91); Member of NAc protein family. Interacts with t	1.022	3.67E-05
At5g24640	unknown protein	2.345	6.51E-07
At5g24655	LSU4 (RESPONSE TO LOW SULFUR 4)	2.386	5.69E-07
At5g24660	LSU2 (RESPONSE TO LOW SULFUR 2)	2.739	3.71E-09
At5g24740	INVOLVED IN: protein localization (Protein of unknown function (DUF1162))	1.84	1.06E-07
At5g24810	ABC1 family protein	1.216	1.49E-07
At5g24830	Tetratricopeptide repeat (TPR)-like superfamily protein	-1.017	4.47E-07
At5g24910	CYP714A1 (cytochrome P450, family 714, subfamily A, polypeptide 1); member of CYP714A	-1.881	1.74E-05
At5g24920	AtGDU5 (glutamine dumper 5); Encodes a member of the GDU (glutamine dumper) family proteins involved	-1.788	5.29E-08
At5g25050	Major facilitator superfamily protein	1.04	5.02E-07
At5g25060	RNA recognition motif (RRM)-containing protein	1.225	1.87E-07
At5g25110	CIPK25 (CBL-interacting protein kinase 25); member of AtCIPKs	-1.451	5.14E-07
At5g25120	CYP71B11 (ytochrome p450, family 71, subfamily B, polypeptide 11); putative cytochrome P450	-1.098	0.0002
At5g25140	CYP71B13 (cytochrome P450, family 71, subfamily B, polypeptide 13); putative cytochrome P450	1.052	1.61E-05
At5g25190	Integrase-type DNA-binding superfamily protein; encodes a member of the ERF (ethylene response factor) sub	-1.081	7.30E-05
At5g25210	unknown protein	-1.758	2.55E-08
At5g25250	SPFH/Band 7/PHB domain-containing membrane-associated protein family	2.359	0.0007
At5g25440	Protein kinase superfamily protein	2.301	7.76E-07
At5g25450	Cytochrome bd ubiquinol oxidase, 14kDa subunit	6.299	4.58E-06
At5g25460	Protein of unknown function, DUF642	-1.889	1.43E-07
At5g25520	SPOC domain / Transcription elongation factor S-II protein	1.599	5.23E-08
At5g25530	DNAJ heat shock family protein	3.986	1.44E-10
At5g25580	BEST Arabidopsis thaliana protein match is: DDT domain superfamily (TAIR:AT1G18950.1)	1.112	3.62E-06
At5g25840	Protein of unknown function (DUF1677)	-1.391	1.75E-05
At5g25920	BEST Arabidopsis thaliana protein match is: Eukaryotic aspartyl protease family protein (TAIR:AT3G29750.1	2.129	4.24E-06

At5g25930	Protein kinase family protein with leucine-rich repeat domain	3.177	6.47E-10
At5g25970	Core-2/I-branching beta-1,6-N-acetylglucosaminyltransferase family protein	-1.181	2.54E-05
At5g26030	FC1 (ferrochelatase 1); encodes ferrochelatase I located in plastids. Involved in heme biosynthesis in non-phot	1.361	2.06E-06
At5g26220	ChaC-like family protein	5.05	1.75E-11
At5g26230	unknown protein	-1.253	2.84E-07
At5g26570	PWD (PHOSPHOGLUCAN WATER DIKINASE); chloroplastidic phosphoglucan, water dikinase (PWD) whi	-1.207	9.74E-07
At5g26920	CBP60G (Cam-binding protein 60-like G); Encodes a calmodulin-binding protein CBP60g (calmodulin bindin	1.312	0.00068
At5g27220	Frigida-like protein	-1.555	2.32E-07
At5g27250	transposase IS4 family protein, contains Pfam profile: PF01609 transposase DDE domain (transposable eleme	-1.147	6.64E-07
At5g27350	SFP1 (Major facilitator superfamily protein); Encodes a sugar-porter family protein that is induced during leaf	1.231	1.66E-06
At5g27420	CNI1 (carbon/nitrogen insensitive 1); Encodes CNI1 (Carbon/Nitrogen Insensitive1) (also named as ATL31), a	2.882	2.73E-08
At5g27460	Tetratricopeptide repeat (TPR)-like superfamily protein	1.062	1.86E-07
At5g27520	PNC2 (peroxisomal adenine nucleotide carrier 2); encodes a peroxisomal adenine nucleotide transporter, invol	1.044	3.92E-06
At5g27580	AGL89 (AGAMOUS-like 89)	1.675	2.83E-05
At5g27660	Trypsin family protein with PDZ domain	4.233	2.86E-12
At5g27760	Hypoxia-responsive family protein	2.579	8.80E-11
At5g27780	SAUR-like auxin-responsive protein family	-1.275	3.04E-07
At5g27950	P-loop containing nucleoside triphosphate hydrolases superfamily protein	-1.262	4.89E-08
At5g27980	Seed maturation protein	1.659	2.29E-08
At5g28176	pseudogene, hypothetical protein (transposable element gene)	1.818	6.48E-09
At5g28540	BIP1 (heat shock protein 70 (Hsp 70) family protein); Encodes the luminal binding protein BiP, an ER-localize	1.242	1.20E-05
At5g28630	glycine-rich protein	1.156	0.00068
At5g28913	SADHU4-1 (sadhu non-coding retrotransposon 4-1); Member of Sadhu non-coding retrotransposon family	-1.226	1.80E-07
At5g32072	pseudogene of Glucose-6-phosphate isomerase; pseudogene of Glucose-6-phosphate isomerase	2.201	3.18E-07
At5g33250	pseudogene, hypothetical protein, predicted proteins - Arabidopsis thaliana (transposable element gene)	1.376	2.50E-09
At5g33370	GDSL-like Lipase/Acylhydrolase superfamily protein	-1.105	0.0158
At5g35200	ENTH/ANTH/VHS superfamily protein	1.121	7.27E-07
At5g35320	unknown protein	3.996	6.56E-13
At5g35460	unknown protein	2.722	8.79E-11
At5g35480	unknown protein	-2.437	2.22E-06
At5g35490	ATMRU1 (ARABIDOPSIS MTO 1 RESPONDING UP 1); Encodes MRU1 (mto 1 responding up). Up-regula	-2.346	1.71E-06
At5g35688	unknown protein	2.018	3.44E-09
At5g35735	Auxin-responsive family protein	2.282	9.22E-07
At5g35960	Protein kinase family protein	2.486	1.95E-09
At5g36120	CCB3 (cofactor assembly, complex C (B6F))	-1.058	7.56E-06
At5g36290	Uncharacterized protein family (UPF0016)	1.19	5.33E-05
At5g36910	THI2.2 (thionin 2.2); Encodes a thionin that is expressed at a low basal level in seedlings and shows circadian	1.378	0.00025
At5g36920	unknown protein	1.543	5.92E-08
At5g36925	unknown protein	1.496	0.0018
At5g37175	similar to nucleic acid binding / ribonuclease H [Arabidopsis thaliana] (TAIR:AT2G15750.1); similar to 80C0	-1.134	0.00264
At5g37240	unknown protein	1.124	0.00011
At5g37260	CIR1 (CIRCADIAN 1); Encodes a MYB family transcription factor Circadian 1 (CIR1). Involved in circadian	2.448	1.03E-07
At5g37340	ZPR1 zinc-finger domain protein	4.53	1.52E-13
At5g37350	Serine/threonine-protein kinase Rio1	1.327	4.39E-09
At5g37380	Chaperone DnaJ-domain superfamily protein	1.278	2.06E-07
At5g37400	Family of unknown function (DUF577)	1.889	2.10E-08
At5g37440	Chaperone DnaJ-domain superfamily protein	1.909	1.97E-09
At5g37500	GORK (gated outwardly-rectifying K+ channel); Encodes a guard cell outward potassium channel. Belongs to	1.578	7.01E-08
At5g37550	unknown protein	2.594	1.34E-09
At5g37670	HSP20-like chaperones superfamily protein	6.677	2.60E-12
At5g37680	ARLA1A (ADP-ribosylation factor-like A1A); A member of ARF-like GTPase family. A thaliana has 21 meml	1.707	3.29E-10
At5g37710	alpha/beta-Hydrolases superfamily protein	1.695	2.93E-09
At5g37750	Chaperone DnaJ-domain superfamily protein	1.493	3.26E-07
At5g37940	Zinc-binding dehydrogenase family protein	1.112	0.00019
At5g37950	UDP-Glycosyltransferase superfamily protein	-1.347	0.00023
At5g37960	GroES-like family protein	1.685	6.63E-08
At5g38010	UDP-Glycosyltransferase superfamily protein	-1.036	3.48E-05
At5g38710	Methylenetetrahydrofolate reductase family protein	-1.25	9.71E-06
At5g38750	asparaginyl-tRNA synthetase family	1.046	1.60E-05
At5g38895	RING/U-box superfamily protein	2.796	3.19E-12
At5g38900	Thioredoxin superfamily protein	1.795	0.0016
At5g38930	RmlC-like cupins superfamily protein	-1.183	0.00142
At5g38940	RmlC-like cupins superfamily protein	-1.292	0.00027
At5g38970	BR6OX (BRASSINOSTEROID-6-OXIDASE); Encodes a polypeptide involved in the C-6 oxidation of brassir	-1.3	1.89E-06
At5g39020	Malectin/receptor-like protein kinase family protein	1.752	3.29E-08
At5g39080	HXXXD-type acyl-transferase family protein	-1.012	3.63E-06
At5g39090	HXXXD-type acyl-transferase family protein	1.238	1.91E-07

At5g39130	RmlC-like cupins superfamily protein	-2.142	1.24E-07
At5g39160	RmlC-like cupins superfamily protein	-1.792	6.57E-07
At5g39190	ATGER2 (A. THALIANA GERMIN LIKE PROTEIN 2); germin-like protein (GLP2a)	-2.063	2.22E-07
At5g39520	Protein of unknown function (DUF1997)	-2.045	1.29E-05
At5g39610	ANAC092 (Arabidopsis NAC domain containing protein 92); Encodes a NAC-domain transcription factor. Po	-2.294	1.63E-07
At5g39650	Protein of unknown function (DUF679)	3.028	1.77E-09
At5g39660	CDF2 (cycling DOF factor 2); Dof-type zinc finger domain-containing protein, identical to H-protein promoter	2.868	5.59E-11
At5g39760	AtHB23 (homeobox protein 23)	-1.361	6.99E-07
At5g39785	Protein of unknown function (DUF1666)	2.142	3.15E-08
At5g39860	BHLH136 (BASIC HELIX-LOOP-HELIX PROTEIN 136); Encodes PRE1 (PACLOBUTRAZOL RESISTAN(	-1.844	2.84E-07
At5g39865	Glutaredoxin family protein	-1.042	2.33E-06
At5g40010	AATP1 (AAA-ATPase 1)	-1.49	0.04435
At5g40150	Peroxidase superfamily protein	-1.102	4.64E-07
At5g40240	nodulin MtN21 /EamA-like transporter family protein	-1.555	1.09E-06
At5g40382	Cytochrome c oxidase subunit Vc family protein	1.323	6.50E-08
At5g40395	gi|2351067|dbj|AB006702.1| Arabidopsis thaliana U6acat At5g40400 gi|2351067|dbj|AB006702.1|	1.033	0.00743
At5g40450	unknown protein	-1.749	1.87E-06
At5g40630	Ubiquitin-like superfamily protein	-2.263	3.05E-09
At5g40645	RPM1-interacting protein 4 (RIN4) family protein	2.057	1.31E-06
At5g40670	PQ-loop repeat family protein / transmembrane family protein	1.403	2.05E-08
At5g40680	Galactose oxidase/kelch repeat superfamily protein	2.091	3.29E-10
At5g40720	Domain of unknown function (DUF23)	-1.098	6.07E-06
At5g40760	G6PD6 (glucose-6-phosphate dehydrogenase 6); Encodes a cytosolic glucose-6-phosphate dehydrogenase that	1.388	3.42E-08
At5g40770	ATPHB3 (prohibitin 3); prohibitin 3	1.293	3.64E-05
At5g40880	WD-40 repeat family protein / zfwd3 protein (ZFWD3)	2.237	1.95E-08
At5g40942	RNR2B (ribonucleotide reductase 2B); pseudogene of ribonucleoside-diphosphate reductase small chain/ ribo	-1.45	2.09E-06
At5g41050	Pollen Ole e 1 allergen and extensin family protein	-1.154	1.77E-05
At5g41070	DRB5 (dsRNA-binding protein 5); Encodes a double-stranded RNA binding protein.	1.276	1.44E-08
At5g41080	PLC-like phosphodiesterases superfamily protein	-1.022	0.0023
At5g41140	Myosin heavy chain-related protein	-1.284	2.64E-07
At5g41320	unknown protein	3.947	5.09E-11
At5g41360	ATXPB2 (ARABIDOPSIS HOMOLOG OF XERODERMA PIGMENTOSUM COMPLEMENTATION GROU	1.46	5.71E-07
At5g41390	PLAC8 family protein	1.672	4.87E-09
At5g41410	BEL1 (BELL 1); Homeodomain protein required for ovule identity.Loss of function mutations show homeotic	-1.305	9.81E-07
At5g41550	Disease resistance protein (TIR-NBS-LRR class) family	1.284	4.10E-06
At5g41560	FUNCTIONS IN: molecular_function unknown; INVOLVED IN: biological_process unknown; LOCATED IN	1.27	4.33E-07
At5g41610	ATCHX18 (ARABIDOPSIS THALIANA CATION/H+ EXCHANGER 18); member of Putative Na+/H+ antip	2.827	4.66E-08
At5g41620	FUNCTIONS IN: molecular_function unknown; INVOLVED IN: biological_process unknown; LOCATED IN	1.815	6.23E-11
At5g41740	Disease resistance protein (TIR-NBS-LRR class) family	1.052	0.0032
At5g41750	Disease resistance protein (TIR-NBS-LRR class) family	2.48	8.05E-05
At5g41761	unknown protein	1.365	1.49E-05
At5g41900	alpha/beta-Hydrolases superfamily protein	-1.546	1.11E-07
At5g41990	WNK8 (with no lysine (K) kinase 8); Encodes a member of the WNK family (9 members in all) of protein kina	2.099	3.91E-11
At5g42000	ORMDL family protein	1.081	3.91E-07
At5g42010	Transducin/WD40 repeat-like superfamily protein	2.152	4.63E-09
At5g42020	BIP (luminal binding protein); Luminal binding protein (BiP2) involved in polar nuclei fusion during prolifera	1.329	6.35E-07
At5g42050	DCD (Development and Cell Death) domain protein	1.365	6.14E-06
At5g42120	Concanavalin A-like lectin protein kinase family protein	1.271	3.97E-08
At5g42150	Glutathione S-transferase family protein	2.146	1.59E-08
At5g42170	SGNH hydrolase-type esterase superfamily protein	4.254	3.62E-11
At5g42180	Peroxidase superfamily protein	3.802	2.79E-08
At5g42220	Ubiquitin-like superfamily protein	1.601	2.39E-09
At5g42230	scpl41 (serine carboxypeptidase-like 41)	1.999	1.39E-08
At5g42380	CML37 (calmodulin like 37)	6.026	2.04E-07
At5g42720	Glycosyl hydrolase family 17 protein	1.153	1.70E-06
At5g42750	BKI1 (BRI1 kinase inhibitor 1); Encodes a plasma-membrane associated phosphoprotein that interacts directly	-1.234	2.00E-06
At5g42760	Leucine carboxyl methyltransferase	3.504	1.19E-09
At5g42785	unknown protein	1.129	0.01483
At5g42825	unknown protein	-1.272	1.49E-06
At5g42830	HXXXD-type acyl-transferase family protein	1.299	6.90E-05
At5g42900	COR27 (cold regulated gene 27)	-2.352	3.66E-10
At5g42965	Polynucleotidyl transferase, ribonuclease H-like superfamily protein	1.775	7.50E-05
At5g43040	Cysteine/Histidine-rich C1 domain family protein	-1.015	2.63E-06
At5g43170	AZF3 (zinc-finger protein 3); Encodes zinc finger protein. mRNA levels are elevated in response to high salin	1.74	1.19E-07
At5g43290	ATWRKY49 (ARABIDOPSIS THALIANA WRKY DNA-BINDING PROTEIN 49); member of WRKY Trans	-1.444	2.81E-05
At5g43300	PLC-like phosphodiesterases superfamily protein	1.208	8.57E-05
At5g43330	Lactate/malate dehydrogenase family protein	1.163	1.94E-06

At5g43350	ATPT1 (ARABIDOPSIS THALIANA PHOSPHATE TRANSPORTER 1); Encodes an inorganic phosphate tra	-1.944	1.18E-05
At5g43370	APT1 (ARABIDOPSIS PHOSPHATE TRANSPORTER 1); Encodes a phosphate transporter Pht1;2. Member	-1.496	2.19E-05
At5g43401	Defensin-like (DEFL) family protein; Encodes a defensin-like (DEFL) family protein.	4.081	1.02E-10
At5g43403	other RNA; Unknown gene	1.029	1.29E-07
At5g43420	RING/U-box superfamily protein	2.548	6.87E-08
At5g43440	2-oxoglutarate (2OG) and Fe(II)-dependent oxygenase superfamily protein; encodes a protein whose sequence	-1.689	6.62E-07
At5g43450	2-oxoglutarate (2OG) and Fe(II)-dependent oxygenase superfamily protein; encodes a protein whose sequence	1.614	0.0002
At5g43490	unknown protein	1.44	5.13E-08
At5g43620	Pre-mRNA cleavage complex II	1.903	1.54E-07
At5g43700	ATAUX2-11 (AUXIN INDUCIBLE 2-11); Auxin inducible protein similar to transcription factors.	-1.622	4.00E-06
At5g43950	Plant protein of unknown function (DUF946)	1.621	9.92E-10
At5g44005	unknown protein	-2.039	2.07E-06
At5g44020	HAD superfamily, subfamily IIIB acid phosphatase	-1.421	0.0002
At5g44060	unknown protein	2.025	2.99E-07
At5g44070	ATPCS1 (ARABIDOPSIS THALIANA PHYTOCHELATIN SYNTHASE 1); Phytochelatin synthase gene cor	2.901	1.18E-09
At5g44080	Basic-leucine zipper (bZIP) transcription factor family protein	1.29	5.48E-08
At5g44090	Calcium-binding EF-hand family protein	1.33	2.26E-07
At5g44130	FLA13 (FASCICLIN-like arabinogalactan protein 13 precursor)	-1.571	6.57E-07
At5g44210	ATERF-9 (ERF DOMAIN PROTEIN- 9); encodes a member of the ERF (ethylene response factor) subfamily l	-1.74	2.76E-07
At5g44260	Zinc finger C-x8-C-x5-C-x3-H type family protein	-2.085	5.47E-09
At5g44360	FAD-binding Berberine family protein	1.517	1.28E-07
At5g44380	FAD-binding Berberine family protein	-1.553	7.10E-05
At5g44390	FAD-binding Berberine family protein	-1.12	0.00077
At5g44400	FAD-binding Berberine family protein	-1.035	1.98E-05
At5g44410	FAD-binding Berberine family protein	-1.363	5.53E-08
At5g44440	FAD-binding Berberine family protein	-1.348	0.00192
At5g44530	Subtilase family protein	-1.389	1.03E-06
At5g44562	other RNA; Potential natural antisense gene, locus overlaps with AT5G44560	-1.791	0.0001
At5g44574	unknown protein	-1.029	8.40E-05
At5g44578	unknown protein	-1.225	8.69E-05
At5g44620	CYP706A3 (cytochrome P450, family 706, subfamily A, polypeptide 3); member of CYP706A	1.159	2.97E-05
At5g44680	DNA glycosylase superfamily protein	-1.739	1.31E-06
At5g44870	Disease resistance protein (TIR-NBS-LRR class) family	1.236	5.16E-07
At5g44990	Glutathione S-transferase family protein	3.143	4.67E-07
At5g45130	ATRAB-F2A (ARABIDOPSIS RAB HOMOLOG F2A); small GTP binding protein	1.621	1.44E-10
At5g45160	Root hair defective 3 GTP-binding protein (RHD3)	1.434	4.25E-06
At5g45180	Flavin-binding monooxygenase family protein	1.67	3.50E-09
At5g45410	unknown protein	1.148	2.68E-07
At5g45430	Protein kinase superfamily protein	1.06	1.52E-06
At5g45550	Mob1/phocein family protein	1.137	8.13E-07
At5g45630	Protein of unknown function, DUF584	2.632	6.50E-08
At5g45650	subtilase family protein	-1.294	6.43E-06
At5g45710	AT-HSFA4C (HEAT SHOCK TRANSCRIPTION FACTOR A4C); member of Heat Stress Transcription Fact	1.202	8.10E-08
At5g45820	CIPK20 (CBL-interacting protein kinase 20); Encodes a CBL-interacting serine/threonine protein kinase comp	-2.035	1.82E-06
At5g46050	ATPTR3 (ARABIDOPSIS THALIANA PEPTIDE TRANSPORTER 3); Encodes a di- and tri-peptide transpor	-1.614	3.49E-06
At5g46080	Protein kinase superfamily protein	2.159	2.14E-09
At5g46170	F-box family protein	1.754	1.62E-09
At5g46200	Protein of Unknown Function (DUF239)	5.4	4.44E-13
At5g46230	Protein of unknown function, DUF538	1.166	1.21E-05
At5g46240	KAT1 (potassium channel in Arabidopsis thaliana 1); Encodes a potassium channel protein (KAT1). ABA trig	-1.937	1.03E-08
At5g46270	Disease resistance protein (TIR-NBS-LRR class) family	-1.348	4.20E-06
At5g46295	unknown protein	3.099	6.55E-09
At5g46330	FLS2 (FLAGELLIN-SENSITIVE 2); Encodes a leucine-rich repeat serine/threonine protein kinase that is expr	-1.348	6.86E-09
At5g46410	SSP4 (SCP1-like small phosphatase 4); Encodes a SCP1-like small phosphatase (SSP). Three SSPs form a uni	1.865	8.74E-06
At5g46470	RPS6 (RESISTANT TO P. SYRINGAE 6); Encodes RPS6 (RESISTANT TO P. SYRINGAE 6), a member of	1.932	5.01E-05
At5g46490	Disease resistance protein (TIR-NBS-LRR class) family	1.233	6.05E-05
At5g46580	pentatricopeptide (PPR) repeat-containing protein	-1.092	7.69E-07
At5g46600	Aluminium activated malate transporter family protein	-1.579	1.38E-05
At5g46610	Aluminium activated malate transporter family protein	1.723	2.68E-08
At5g46710	PLATZ transcription factor family protein	1.278	5.74E-07
At5g46780	VQ motif-containing protein	1.911	9.97E-08
At5g46910	Transcription factor jumonji (jmj) family protein / zinc finger (C5HC2 type) family protein	2.831	6.62E-11
At5g47070	Protein kinase superfamily protein	1.514	2.86E-07
At5g47075	LCR20 (low-molecular-weight cysteine-rich 20); Encodes a member of a family of small,secreted, cysteine ricl	1.43	0.00045
At5g47077	LCR6 (low-molecular-weight cysteine-rich 6); Encodes a member of a family of small,secreted, cysteine rich p	2.906	2.91E-10
At5g47120	ATBI-1 (ARABIDOPSIS BAX INHIBITOR 1); Encodes BI-1, a homolog of mammalian Bax inhibitor 1. Fun	3.127	1.98E-10
At5g47160	YDG/SRA domain-containing protein	1.927	3.01E-08

At5g47175	LCR3 (low-molecular-weight cysteine-rich 3); Encodes a member of a family of small,secreted, cysteine rich p	2.707	3.15E-10
At5g47230	ATERF-5 (ETHYLENE RESPONSIVE ELEMENT BINDING FACTOR- 5); encodes a member of the ERF (e	1.026	0.00387
At5g47240	atnudt8 (nudix hydrolase homolog 8)	-2.242	1.63E-07
At5g47250	LRR and NB-ARC domains-containing disease resistance protein	1.711	1.42E-09
At5g47330	alpha/beta-Hydrolases superfamily protein	-1.818	3.10E-06
At5g47370	HAT2 (Homeobox-leucine zipper protein 4 (HB-4) / HD-ZIP protein); homeobox-leucine zipper genes induce	-1.804	3.21E-08
At5g47550	Cystatin/monellin superfamily protein	-1.153	4.87E-08
At5g47560	ATTDT (TONOPLAST DICARBOXYLATE TRANSPORTER); Encodes a tonoplast malate/fumarate transpo	-1.006	0.0001
At5g47610	RING/U-box superfamily protein	3.406	4.41E-12
At5g47780	GAUT4 (galacturonosyltransferase 4); Encodes a protein with putative galacturonosyltransferase activity.	-1.11	1.66E-07
At5g47800	Phototropic-responsive NPH3 family protein	-1.056	4.26E-06
At5g47830	unknown protein	3.777	2.50E-12
At5g47910	RBOHD (respiratory burst oxidase homologue D); NADPH/respiratory burst oxidase protein D (RbohD).Intera	1.377	1.64E-05
At5g47960	ATRABA4C (RAB GTPase homolog A4C); Encodes a small molecular weight g-protein.	1.849	2.52E-06
At5g48030	GFA2 (gametophytic factor 2); encodes a mitochondrially targeted DNAJ protein involved in female gametop	1.21	8.58E-08
At5g48180	NSP5 (nitrile specifier protein 5); Encodes a nitrile-specifier protein NSP5. NSP5 is one out of five (At3g1640	2.625	2.29E-09
At5g48380	BIR1 (BAK1-interacting receptor-like kinase 1); Encodes a BAK1-interacting receptor-like kinase named BIR	1.028	3.62E-06
At5g48430	Eukaryotic aspartyl protease family protein	-1.355	0.00194
At5g48460	Actin binding Calponin homology (CH) domain-containing protein	-1.078	1.19E-06
At5g48530	unknown protein	2.938	5.43E-11
At5g48540	receptor-like protein kinase-related family protein	4.45	5.42E-10
At5g48570	FKBP-type peptidyl-prolyl cis-trans isomerase family protein	5.906	2.30E-12
At5g48610	unknown protein	1.754	2.26E-08
At5g48655	RING/U-box superfamily protein	1.37	8.27E-08
At5g48657	defense protein-related	2.874	3.05E-07
At5g48730	Pentatricopeptide repeat (PPR) superfamily protein	-1.167	7.69E-08
At5g48850	ATSDI1 (SULPHUR DEFICIENCY-INDUCED 1); homologous to the wheat sulphate deficiency-induced gen	3.979	1.26E-07
At5g48900	Pectin lyase-like superfamily protein	-1.817	1.11E-09
At5g49015	Expressed protein	-1.258	2.05E-06
At5g49170	unknown protein	-1.632	1.29E-07
At5g49200	WD-40 repeat family protein / zfwd4 protein (ZFWD4)	3.42	1.04E-08
At5g49360	ATBXL1 (BETA-XYLOSIDASE 1); Encodes a bifunctional {beta}-D-xylosidase/{alpha}-L-arabinofuranosida	-1.523	4.21E-05
At5g49390	LOCATED IN: endomembrane system	1.269	0.0072
At5g49480	ATCP1 (Ca2+-binding protein 1); AtCP1 encodes a novel Ca2+-binding protein, which shares sequence simila	2.626	9.49E-09
At5g49525	unknown protein	-1.172	2.45E-07
At5g49600	Protein of unknown function, DUF538	-1.862	0.00012
At5g49660	Leucine-rich repeat transmembrane protein kinase family protein	-1.942	5.53E-08
At5g49730	ATFRO6 (ferric reduction oxidase 6); Encodes a plasma membrane-located ferric chelate reductase. Its mRNA	-1.537	0.0001
At5g49930	emb1441 (embryo defective 1441)	1.297	7.53E-07
At5g49960	unknown protein	-1.25	2.15E-07
At5g50030	Plant invertase/pectin methylesterase inhibitor superfamily protein	3.938	4.44E-13
At5g50100	Putative thiol-disulphide oxidoreductase DCC	1.563	1.49E-07
At5g50120	Transducin/WD40 repeat-like superfamily protein	-1.317	0.00046
At5g50240	PIMT2 (protein-l-isoaspartate methyltransferase 2); L-isoaspartyl methyltransferase 2 (PIMT2)gene, alternativ	2.717	4.09E-10
At5g50335	unknown protein	-1.613	4.84E-07
At5g50450	HCP-like superfamily protein with MYND-type zinc finger	-1.155	1.54E-06
At5g50610	unknown protein	-1.321	5.74E-05
At5g50740	Heavy metal transport/detoxification superfamily protein	-1.033	6.19E-06
At5g50760	SAUR-like auxin-responsive protein family	-1.104	8.87E-05
At5g50780	Histidine kinase-, DNA gyrase B-, and HSP90-like ATPase family protein	1.376	5.74E-07
At5g50790	Nodulin MtN3 family protein	-1.722	0.00143
At5g50800	Nodulin MtN3 family protein	-2.582	4.76E-05
At5g50820	anac097 (NAC domain containing protein 97)	-1.238	8.34E-07
At5g50915	basic helix-loop-helix (bHLH) DNA-binding superfamily protein	1.742	9.78E-06
At5g50960	ATNBP35 (NUCLEOTIDE BINDING PROTEIN 35); Highly similar to Saccharomyces cerevisiae NBP35, loc	1.031	1.52E-06
At5g51050	Mitochondrial substrate carrier family protein	1.193	2.92E-08
At5g51060	ATRBOHC (A. THALIANA RESPIRATORY BURST OXIDASE HOMOLOG C); RHD2 (along with RHD3	1.969	4.56E-06
At5g51130	S-adenosyl-L-methionine-dependent methyltransferases superfamily protein	1.336	9.74E-07
At5g51370	RNI-like superfamily protein	1.66	5.79E-10
At5g51420	long-chain-alcohol O-fatty-acyltransferase family protein / wax synthase family protein	1.825	0.00012
At5g51430	EYE (EMBRYO YELLOW); Encodes a protein that is homologous to Cog7, a subunit of the conserved oligon	2.07	7.02E-09
At5g51440	HSP20-like chaperones superfamily protein	7.403	4.78E-12
At5g51451	RGF5 (root meristem growth factor 5); Encodes a root meristem growth factor (RGF). Belongs to a family of f	2.144	1.60E-08
At5g51550	EXL3 (EXORDIUM like 3)	-1.098	1.94E-05
At5g51630	Disease resistance protein (TIR-NBS-LRR class) family	2.572	4.57E-10
At5g51670	Protein of unknown function (DUF668)	-1.348	9.26E-07
At5g51720	2 iron, 2 sulfur cluster binding	1.063	3.82E-05

At5g51740	Peptidase family M48 family protein	3.118	6.57E-12
At5g51790	basic helix-loop-helix (bHLH) DNA-binding superfamily protein	-1.651	1.31E-05
At5g51810	GA20OX2 (gibberellin 20 oxidase 2); Encodes gibberellin 20-oxidase. Involved in gibberellin biosynthesis. U	-1.429	1.17E-07
At5g51830	pfkB-like carbohydrate kinase family protein	1.609	6.73E-07
At5g52000	IMPA-8 (importin alpha isoform 8); Putative importin alpha isoform. When overexpressed can rescue the impa	2.99	2.78E-08
At5g52020	Integrase-type DNA-binding superfamily protein; encodes a member of the DREB subfamily A-4 of ERF/AP2	1.203	2.07E-05
At5g52120	AtPP2-A14 (phloem protein 2-A14)	-1.317	6.20E-07
At5g52250	Transducin/WD40 repeat-like superfamily protein; Encodes a transducin protein whose gene expression is ind	-1.83	6.88E-10
At5g52280	Myosin heavy chain-related protein	-1.121	4.70E-07
At5g52310	COR78 (COLD REGULATED 78); cold regulated gene, the 5' region of cor78 has cis-acting regulatory elemen	-1.331	6.70E-06
At5g52570	BCH2 (BETA CAROTENOID HYDROXYLASE 2); Converts β-carotene to zeaxanthin via cryptoxanth	5.062	4.93E-11
At5g52580	RabGAP/TBC domain-containing protein	1.046	6.03E-05
At5g52610	F-box and associated interaction domains-containing protein	1.133	0.00104
At5g52640	AtHsp90-1 (HEAT SHOCK PROTEIN 90-1); Encodes a cytosolic heat shock protein AtHSP90.1. AtHSP90.1	6.235	3.98E-11
At5g52740	Copper transport protein family	1.765	5.01E-06
At5g52750	Heavy metal transport/detoxification superfamily protein	2.155	4.05E-07
At5g52760	Copper transport protein family	2.835	0.0003
At5g52790	CBS domain-containing protein with a domain of unknown function (DUF21)	2.816	1.44E-10
At5g52830	ATWRKY27 (ARABIDOPSIS THALIANA WRKY DNA-BINDING PROTEIN 27); Encodes a WRKY transc	-1.029	3.06E-06
At5g52882	P-loop containing nucleoside triphosphate hydrolases superfamily protein	-1.042	9.29E-06
At5g52900	unknown protein	-1.57	1.21E-06
At5g53000	TAP46 (2A phosphatase associated protein of 46 kD); PP2A-associated protein with a possible function in the	1.54	2.75E-09
At5g53020	Ribonuclease P protein subunit P38-related	-1.262	2.71E-07
At5g53048	other RNA; Potential natural antisense gene, locus overlaps with AT5G53050	-1.783	0.0002
At5g53050	alpha/beta-Hydrolases superfamily protein	1.78	9.91E-09
At5g53120	ATSPDS3 (SPERMIDINE SYNTHASE 3); encodes a novel spermine synthase and is a paralog of previously c	1.505	7.76E-07
At5g53200	TRY (TRIPTYCHON)	1.013	0.00018
At5g53400	BOB1 (BOBBER1); Encodes BOBBER1 (BOB1), a non-canonical small heat shock protein required for both	3.093	1.62E-11
At5g53460	GLT1 (NADH-dependent glutamate synthase 1); NADH-dependent glutamate synthase	-1.374	1.05E-05
At5g53550	ATYSL3 (YELLOW STRIPE LIKE 3)	-1.549	3.48E-07
At5g53680	RNA-binding (RRM/RBD/RNP motifs) family protein	6.153	5.22E-15
At5g53730	Late embryogenesis abundant (LEA) hydroxyproline-rich glycoprotein family	-1.861	4.53E-07
At5g53870	ENODL1 (early nodulin-like protein 1)	1.019	0.00022
At5g53880	unknown protein	-1.191	1.57E-07
At5g53890	PSKR2 (phytosylfokine-alpha receptor 2); Encodes a leucine-rich repeat receptor kinase (LRR-RK) involved i	-1.286	1.52E-08
At5g53970	Tyrosine transaminase family protein; encodes tyrosine aminotransferase which is strongly induced upon aging	2.081	2.52E-08
At5g53980	ATHB52 (homeobox protein 52); Encodes a homeodomain leucine zipper class I (HD-Zip I) protein.	-1.573	1.02E-05
At5g54145	unknown protein	-2.087	1.92E-09
At5g54165	unknown protein	8.589	1.46E-12
At5g54190	PORA (protochlorophyllide oxidoreductase A); light-dependent NADPH:protochlorophyllide oxidoreductase A	-1.986	0.0026
At5g54300	Protein of unknown function (DUF761)	1.692	7.17E-08
At5g54470	B-box type zinc finger family protein	1.246	0.00016
At5g54490	PBP1 (pinoid-binding protein 1); Encodes a PINOID (PID)-binding protein containing putative EF-hand calciu	1.883	8.08E-07
At5g54510	DFL1 (DWARF IN LIGHT 1); Encodes an IAA-amido synthase that conjugates Ala, Asp, Phe, and Trp to auxi	-1.741	1.74E-08
At5g54585	unknown protein	-1.539	1.18E-05
At5g54660	HSP20-like chaperones superfamily protein	-1.089	1.77E-05
At5g54690	GAUT12 (galacturonosyltransferase 12); Encodes a protein with putative galacturonosyltransferase activity. M	1.493	4.21E-08
At5g54710	Ankyrin repeat family protein	1.888	1.54E-07
At5g54860	Major facilitator superfamily protein	2.992	4.48E-11
At5g54870	unknown protein	1.117	1.73E-05
At5g55050	GDSL-like Lipase/Acylhydrolase superfamily protein	1.685	3.87E-05
At5g55240	ATPXG2 (ARABIDOPSIS THALIANA PEROXYGENASE 2); Catalyze hydroperoxide-dependent mono-oxy	1.471	3.71E-06
At5g55250	IAMT1 (IAA carboxylmethyltransferase 1); Encodes an enzyme which specifically converts IAA to its methyl	-1.01	0.0013
At5g55410	Bifunctional inhibitor/lipid-transfer protein/seed storage 2S albumin superfamily protein	-2.677	0.00194
At5g55420	Encodes a Protease inhibitor/seed storage/LTP family protein [pseudogene]; Encodes a Protease inhibitor/seed	-1.239	0.00021
At5g55460	Bifunctional inhibitor/lipid-transfer protein/seed storage 2S albumin superfamily protein	-1.131	0.00214
At5g55580	Mitochondrial transcription termination factor family protein	-1.098	2.74E-07
At5g55620	unknown protein	-1.047	9.54E-06
At5g55690	MADS-box transcription factor family protein	-2.599	4.66E-05
At5g55840	Pentatricopeptide repeat (PPR) superfamily protein	1.391	3.17E-09
At5g55970	RING/U-box superfamily protein	1.682	4.52E-06
At5g56010	AtHsp90-3 (HEAT SHOCK PROTEIN 90-3); A member of heat shock protein 90 (HSP90) gene family. Expre	1.114	6.46E-06
At5g56030	AtHsp90.2 (HEAT SHOCK PROTEIN 90.2); A member of heat shock protein 90 (HSP90) gene family. Expre	1.26	4.25E-07
At5g56040	Leucine-rich receptor-like protein kinase family protein	-1.191	9.48E-08
At5g56150	UBC30 (ubiquitin-conjugating enzyme 30)	1.484	1.59E-06
At5g56310	Pentatricopeptide repeat (PPR) superfamily protein	-1.201	4.91E-06
At5g56320	ATEXPA14 (expansin A14); member of Alpha-Expansin Gene Family. Naming convention from the Expansin	-1.673	3.98E-08

At5g56350	Pyruvate kinase family protein	1.025	2.26E-05
At5g56430	F-box/FBD-like domains containing protein	1.226	8.50E-06
At5g56600	PFN3 (PROFILIN 3); Encodes profilin3, a low-molecular weight, actin monomer-binding protein that regulate	1.342	6.90E-10
At5g56690	FBD, F-box and Leucine Rich Repeat domains containing protein	1.137	3.08E-07
At5g56750	NDL1 (N-MYC downregulated-like 1)	2.909	6.76E-11
At5g56840	myb-like transcription factor family protein	1.201	0.00011
At5g56870	BGAL4 (beta-galactosidase 4)	-1.683	1.20E-05
At5g56880	unknown protein	1.728	2.35E-06
At5g56960	basic helix-loop-helix (bHLH) DNA-binding family protein	5.512	2.95E-11
At5g57010	calmodulin-binding family protein	1.804	0.00024
At5g57035	U-box domain-containing protein kinase family protein	1.64	1.87E-08
At5g57050	ABI2 (ABA INSENSITIVE 2); Encodes a protein phosphatase 2C and is involved in ABA signal transduction.	3.337	2.03E-10
At5g57060	FUNCTIONS IN: molecular_function unknown; INVOLVED IN: defense response to Gram-negative bacteriu	1.003	8.75E-07
At5g57110	ACA8 (autoinhibited Ca2+ -ATPase, isoform 8); Arabidopsis-autoinhibited Ca2+ -ATPase, isoform 8, contain	-1.28	0.0001
At5g57123	unknown protein	2.049	1.04E-05
At5g57180	CIA2 (chloroplast import apparatus 2); Transcription regulator responsible for specific upregulation of the tra	-1.052	8.23E-07
At5g57220	CYP81F2 (cytochrome P450, family 81, subfamily F, polypeptide 2); member of CYP81F, involved in glucosi	1.463	4.36E-05
At5g57260	CYP71B10 (cytochrome P450, family 71, subfamily B, polypeptide 10); putative cytochrome P450	1.884	7.45E-07
At5g57350	AHA3 (H(+)-ATPase 3); member of Plasma membrane H+-ATPase family	-1.433	1.11E-07
At5g57567	unknown protein	-1.29	4.55E-07
At5g57570	GCK domain-containing protein	-1.247	1.86E-06
At5g57630	CIPK21 (CBL-interacting protein kinase 21); CBL-interacting protein kinase	-2.076	3.28E-10
At5g57640	GCK domain-containing protein	-1.507	1.77E-05
At5g57685	ATGDU3 (ARABIDOPSIS THALIANA GLUTAMINE DUMPER 3); Encodes a member of the GDU (glutam	-1.113	3.09E-06
At5g57710	Double Clp-N motif-containing P-loop nucleoside triphosphate hydrolases superfamily protein	-1.087	5.21E-07
At5g57760	unknown protein	-3.738	1.23E-09
At5g57770	Plant protein of unknown function (DUF828) with plant pleckstrin homology-like region	-1.257	1.88E-05
At5g57780	EXPRESSED IN: 18 plant structures	-1.558	1.44E-06
At5g57910	unknown protein	2.82	1.50E-10
At5g57920	ENODL10 (early nodulin-like protein 10)	2.329	1.53E-08
At5g58040	ATFIP1[V] (homolog of yeast FIP1 [V]); Encodes a subunit of the polyadenylation apparatus that interacts wit	1.599	7.50E-05
At5g58070	ATTIL (TEMPERATURE-INDUCED LIPOCALIN); Encodes a temperature-induced lipocalin TIL1. Involvec	2.466	2.16E-09
At5g58110	chaperone binding	2.171	3.95E-10
At5g58120	Disease resistance protein (TIR-NBS-LRR class) family	3.075	1.08E-08
At5g58290	RPT3 (regulatory particle triple-A ATPase 3); 26S proteasome AAA-ATPase subunit RPT3 (RPT3) mRNA,	1.076	2.71E-05
At5g58310	ATMES18 (ARABIDOPSIS THALIANA METHYL ESTERASE 18); Encodes a protein shown to have methy	1.743	7.53E-05
At5g58320	Kinase interacting (KIP1-like) family protein	1.256	2.85E-06
At5g58360	ATOFP3 (ARABIDOPSIS THALIANA OVATE FAMILY PROTEIN 3)	-1.276	2.32E-07
At5g58430	ATEXO70B1 (exocyst subunit exo70 family protein B1); A member of EXO70 gene family, putative exocyst s	1.937	2.31E-10
At5g58570	unknown protein	-2.148	2.17E-07
At5g58580	ATL63 (TOXICOS EN LEVADURA 63)	-1.3	1.04E-05
At5g58590	RANBP1 (RAN binding protein 1); Encodes a Ran-binding protein 1 homolog (RanBP1).	1.304	9.42E-08
At5g58660	2-oxoglutarate (2OG) and Fe(II)-dependent oxygenase superfamily protein	-1.765	9.09E-06
At5g58670	ATPLC (ARABIDOPSIS THALIANA PHOSPHOLIPASE C); phosphatidylinositol-specific phospholipase C i	-1.698	7.74E-07
At5g58680	ARM repeat superfamily protein	2.479	1.37E-10
At5g58760	DDB2 (damaged DNA binding 2); Encodes a DDB1a interacting protein DDB2 required for UV-B tolerance a	1.42	0.00013
At5g58770	Undecaprenyl pyrophosphate synthetase family protein	7.225	3.38E-12
At5g58787	RING/U-box superfamily protein	2.258	7.05E-11
At5g58810	subtilisin-like serine protease, pseudogene, contains similarity to prepro-cucumisin GI:807698 from (Cucumis	2.965	3.90E-09
At5g58830	Subtilisin-like serine endopeptidase family protein	1.491	1.94E-06
At5g58850	ATMYB119 (MYB DOMAIN PROTEIN 119); Encodes a putative transcription factor, member of the R2R3 f	1.032	2.13E-07
At5g58900	Homeodomain-like transcriptional regulator	-1.484	1.45E-07
At5g58940	CRCK1 (calmodulin-binding receptor-like cytoplasmic kinase 1); Arabidopsis thaliana calmodulin-binding rec	1.558	1.03E-07
At5g59020	Protein of unknown function (DUF3527)	-1.033	1.61E-07
At5g59080	unknown protein	-1.647	2.00E-06
At5g59220	HAI1 (highly ABA-induced PP2C gene 1)	1.266	3.42E-06
At5g59230	transcription factor-related	2.087	3.32E-07
At5g59340	WOX2 (WUSCHEL related homeobox 2); Encodes a WUSCHEL-related homeobox gene family member with	1.613	5.23E-06
At5g59360	unknown protein	-1.247	5.69E-07
At5g59390	XH/XS domain-containing protein	1.457	1.36E-05
At5g59450	GRAS family transcription factor	1.032	7.81E-05
At5g59510	DVL18 (DEVIL 18)	1.091	0.00016
At5g59520	ZIP2 (ZRT/IRT-like protein 2); encodes a metal ion transporter whose expression is regulated by copper.	-1.014	0.0008
At5g59530	2-oxoglutarate (2OG) and Fe(II)-dependent oxygenase superfamily protein	1.101	4.69E-05
At5g59540	2-oxoglutarate (2OG) and Fe(II)-dependent oxygenase superfamily protein	2.122	7.26E-09
At5g59550	zinc finger (C3HC4-type RING finger) family protein	2.423	1.25E-07
At5g59570	Homeodomain-like superfamily protein	-1.747	4.84E-07

At5g59630	pseudogene, glycine-rich protein, blastp match of 53% identity and 5.4e-10 P-value to GP|9837280|gb|AAG0(	1.734	1.62E-09
At5g59720	HSP18.2 (heat shock protein 18.2); encodes a low molecular weight heat shock protein that contains the heat s	6.918	7.25E-13
At5g59730	ATEXO70H7 (exocyst subunit exo70 family protein H7); A member of EXO70 gene family, putative exocyst :	2.286	2.29E-07
At5g59750	DHBP synthase RibB-like alpha/beta domain	1.035	7.49E-07
At5g59820	RHL41 (RESPONSIVE TO HIGH LIGHT 41); Encodes a zinc finger protein involved in high light and cold ac	4.788	2.25E-08
At5g59870	HTA6 (histone H2A 6); Encodes HTA6, a histone H2A protein.	-1.209	5.71E-05
At5g59920	ULI3 (UV-B LIGHT INSENSITIVE 3); Isolated in a screen for UV-B insensitive mutants using a hypocotyl gr	1.295	3.07E-05
At5g59970	Histone superfamily protein	-1.111	6.07E-05
At5g60100	APRR3 (pseudo-response regulator 3); Encodes pseudo-response regulator 3 (APRR3/PRR3). PRR3 transcript	-2.226	1.46E-07
At5g60130	AP2/B3-like transcriptional factor family protein	2.386	3.00E-10
At5g60170	RNA binding (RRM/RBD/RNP motifs) family protein	1.717	6.44E-05
At5g60250	zinc finger (C3HC4-type RING finger) family protein	1.663	2.98E-05
At5g60280	Concanavalin A-like lectin protein kinase family protein	1.329	1.06E-05
At5g60400	unknown protein	-1.095	1.44E-05
At5g60610	F-box/RNI-like superfamily protein	3.731	7.97E-12
At5g60680	Protein of unknown function, DUF584	-1.318	3.12E-06
At5g60710	Zinc finger (C3HC4-type RING finger) family protein	-1.143	3.81E-07
At5g60780	ATNRT2.3 (ARABIDOPSIS THALIANA NITRATE TRANSPORTER 2.3); member of High affinity nitrate tı	-1.447	3.07E-05
At5g60800	Heavy metal transport/detoxification superfamily protein	1.973	9.46E-07
At5g60890	ATR1 (ALTERED TRYPTOPHAN REGULATION 1); Myb-like transcription factor that modulates expressio	-1.64	1.53E-07
At5g60900	RLK1 (receptor-like protein kinase 1); Encodes a receptor-like protein kinase.	-1.893	0.00048
At5g61000	RPA70D (Replication factor-A protein 1-related)	-1.021	0.00041
At5g61180	Putative endonuclease or glycosyl hydrolase	1.657	0.00026
At5g61190	putative endonuclease or glycosyl hydrolase with C2H2-type zinc finger domain	2.233	1.54E-09
At5g61210	SNAP33 (soluble N-ethylmaleimide-sensitive factor adaptor protein 33); membrane localized t-SNARE SNAP	1.489	2.82E-07
At5g61290	Flavin-binding monooxygenase family protein	-1.088	2.89E-06
At5g61370	Pentatricopeptide repeat (PPR) superfamily protein	-1.164	8.71E-06
At5g61380	AtTOC1 (TIMING OF CAB EXPRESSION 1); Pseudo response regulator involved in the generation of circad	-1.257	1.53E-07
At5g61420	HAG1 (HIGH ALIPHATIC GLUCOSINOLATE 1); Encodes a nuclear localized member of the MYB transcri	-2.02	7.09E-09
At5g61430	ANAC100 (NAC domain containing protein 100)	-1.391	4.01E-06
At5g61510	GroES-like zinc-binding alcohol dehydrogenase family protein	1.91	6.55E-09
At5g61520	Major facilitator superfamily protein	-1.072	8.98E-05
At5g61560	U-box domain-containing protein kinase family protein	2.538	3.64E-10
At5g61590	Integrase-type DNA-binding superfamily protein; encodes a member of the ERF (ethylene response factor) sub	-1.639	2.03E-07
At5g61610	Oleosin family protein	-1.357	1.71E-05
At5g61650	CYCP4.2 (CYCLIN P4.2); The P-type cyclins (CYCPs) share a conserved central region of 100 amino acids ('	-2.381	7.66E-06
At5g61660	glycine-rich protein	-2.408	7.80E-09
At5g61730	ATATH11 (ARABIDOPSIS THALIANA ABC2 HOMOLOG 11); member of ATH subfamily	2.64	1.51E-10
At5g61900	BON1 (BONZAI 1); Encodes a plasma-membrane localized, copine-like protein, which is a member of a newl	1.186	1.46E-05
At5g61980	AGD1 (ARF-GAP domain 1); A member of ARF GAP domain (AGD), A thaliana has 15 members, grouped i	2.104	8.35E-09
At5g62020	AT-HSFB2A (ARABIDOPSIS THALIANA HEAT SHOCK TRANSCRIPTION FACTOR B2A); member of	4.568	4.42E-12
At5g62080	Bifunctional inhibitor/lipid-transfer protein/seed storage 2S albumin superfamily protein	1.454	1.24E-05
At5g62090	SLK2 (SEUSS-like 2)	1.367	2.83E-08
At5g62180	AtCXE20 (carboxyesterase 20)	1.672	7.94E-08
At5g62200	Embryo-specific protein 3, (ATS3)	1.159	1.74E-08
At5g62280	Protein of unknown function (DUF1442)	-3.725	3.97E-09
At5g62350	Plant invertase/pectin methylesterase inhibitor superfamily protein	-1.436	2.21E-07
At5g62360	Plant invertase/pectin methylesterase inhibitor superfamily protein	-2.885	8.20E-08
At5g62430	CDF1 (cycling DOF factor 1); Dof-type zinc finger domain-containing protein, similar to H-protein promoter b	2.398	1.09E-08
At5g62480	ATGSTU9 (glutathione S-transferase tau 9); Encodes glutathione transferase belonging to the tau class of GST	1.06	0.01972
At5g62520	similar to RCD one 5 (SRO5); FUNCTIONS IN: NAD+ ADP-ribosyltransferase activity; INVOLVED IN: oxy	1.894	0.0015
At5g62570	Calmodulin binding protein-like	1.074	2.15E-05
At5g62620	Galactosyltransferase family protein	2.248	1.26E-10
At5g62623	Putative membrane lipoprotein; Encodes a defensin-like (DEFL) family protein.	1.755	2.38E-09
At5g62627	Putative membrane lipoprotein; Encodes a defensin-like (DEFL) family protein.	1.199	0.00012
At5g62630	HIPL2 (hipl2 protein precursor)	-1.404	1.24E-07
At5g62680	Major facilitator superfamily protein	-1.238	1.67E-07
At5g62720	Integral membrane HPP family protein	-2.265	5.26E-09
At5g62730	Major facilitator superfamily protein	-2.222	3.97E-05
At5g62770	Protein of unknown function (DUF1645)	1.204	1.68E-05
At5g62850	AtVEX1 (VEGETATIVE CELL EXPRESSED1); Encodes a protein that is expressed in vegetative cells of pol	-1.249	4.68E-06
At5g62900	unknown protein	-1.111	5.53E-07
At5g62920	ARR6 (response regulator 6); Encodes a Type-A response regulator that is responsive to cytokinin treatment.	-1.286	3.44E-06
At5g62990	emb1692 (embryo defective 1692)	-1.235	2.93E-06
At5g63070	Ribosomal protein S19 family protein	2.358	2.09E-09
At5g63130	Octicosapeptide/Phox/Bem1p family protein	1.439	4.57E-06
At5g63160	BT1 (BTB and TAZ domain protein 1); BTB and TAZ domain protein. Short-lived nuclear-cytoplasmic protei	-2.367	1.70E-05

At5g63180	Pectin lyase-like superfamily protein	-1.319	1.33E-07
At5g63195	other RNA; Potential natural antisense gene, locus overlaps with AT5G63190	1.565	7.82E-08
At5g63300	Ribosomal protein S21 family protein	3.153	4.89E-10
At5g63350	unknown protein	1.259	2.78E-06
At5g63390	O-fucosyltransferase family protein	1.761	7.34E-08
At5g63420	emb2746 (embryo defective 2746)	-1.127	2.22E-05
At5g63500	Protein of unknown function (DUF 3339)	2.528	3.38E-10
At5g63710	Leucine-rich repeat protein kinase family protein	-1.139	3.38E-06
At5g63770	ATDGK2 (diacylglycerol kinase 2); a member of the diacylglycerol kinase gene family. Encodes a functional d	1.905	2.19E-07
At5g63790	ANAC102 (NAC domain containing protein 102); Encodes a member of the NAC family of transcription facto	1.702	3.18E-09
At5g63800	BGAL6 (beta-galactosidase 6); Involved in mucilage formation. Mutants form columella and outer cell wall ar	-1.314	1.22E-05
At5g63810	BGAL10 (beta-galactosidase 10); member of Glycoside Hydrolase Family 35	-1.715	1.07E-06
At5g63820	Protein of unknown function (DUF626)	-1.41	9.90E-07
At5g63830	HIT-type Zinc finger family protein	1.181	9.09E-07
At5g63850	AAP4 (amino acid permease 4); Amino acid transporter whose expression is downregulated by dehydration.	-1.929	1.15E-08
At5g64000	SAL2 (Inositol monophosphatase family protein); 3'(2'),5'-bisphosphate nucleotidase	-1.712	0.00322
At5g64110	Peroxidase superfamily protein	-1.199	0.00271
At5g64170	dentin sialophosphoprotein-related	1.787	1.25E-06
At5g64190	unknown protein	-1.67	4.42E-05
At5g64200	ATSC35 (ARABIDOPSIS THALIANA ORTHOLOG OF HUMAN SPLICING FACTOR SC35); encodes an S	1.298	5.29E-08
At5g64230	unknown protein	3.374	3.56E-10
At5g64250	Aldolase-type TIM barrel family protein	1.486	2.43E-06
At5g64310	AGP1 (arabinogalactan protein 1); Encodes arabinogalactan-protein (AGP1).	1.386	9.81E-07
At5g64400	CONTAINS InterPro DOMAIN/s: CHCH (InterPro:IPR010625); BEST Arabidopsis thaliana protein match is:	1.241	3.74E-08
At5g64410	ATOPT4 (ARABIDOPSIS THALIANA OLIGOPEPTIDE TRANSPORTER 4); oligopeptide transporter	-1.366	6.31E-08
At5g64490	ARM repeat superfamily protein	-1.307	1.38E-06
At5g64510	unknown protein	6.581	6.17E-13
At5g64572	other RNA; Potential natural antisense gene, locus overlaps with AT5G64570	-1.083	1.05E-05
At5g64640	Plant invertase/pectin methylesterase inhibitor superfamily	-1.087	3.48E-06
At5g64700	nodulin MtN21 /EamA-like transporter family protein	-1.204	3.70E-05
At5g64750	ABR1 (ABA REPRESSOR1); Encodes a putative transcription factor containing an AP2 domain. Is a member	2.422	3.59E-08
At5g64770	RGF9 (root meristem growth factor 9); Encodes a root meristem growth factor (RGF). Belongs to a family of f	-1.858	5.23E-08
At5g64840	ATGCN5 (general control non-repressible 5); member of GCN subfamily	1.097	1.69E-06
At5g64905	PROPEP3 (elicitor peptide 3 precursor)	1.258	6.13E-05
At5g64910	unknown protein	1.524	8.87E-07
At5g64930	CPR5 (CONSTITUTIVE EXPRESSION OF PR GENES 5); Regulator of expression of pathogenesis-related (I	1.382	2.22E-07
At5g64950	Mitochondrial transcription termination factor family protein	2.866	9.81E-11
At5g65010	ASN2 (asparagine synthetase 2); Encodes asparagine synthetase (ASN2).	-1.246	6.75E-06
At5g65200	ATPUB38 (ARABIDOPSIS THALIANA PLANT U-BOX 38); Encodes a protein containing a U-box and an A	1.003	8.89E-07
At5g65310	ATHB5 (homeobox protein 5); Encodes a class I HDZip (homeodomain-leucine zipper) protein that is a positi	-1.663	1.39E-08
At5g65320	basic helix-loop-helix (bHLH) DNA-binding superfamily protein	-1.25	1.55E-06
At5g65360	Histone superfamily protein	-1.129	2.68E-05
At5g65380	MATE efflux family protein	1.133	3.86E-08
At5g65390	AGP7 (arabinogalactan protein 7)	-1.215	1.64E-06
At5g65590	Dof-type zinc finger DNA-binding family protein	-1.01	3.89E-05
At5g65600	Concanavalin A-like lectin protein kinase family protein	2.708	1.88E-07
At5g65630	GTE7 (global transcription factor group E7); This gene is predicted to encode a bromodomain-containing prote	-1.038	9.22E-07
At5g65650	Protein of unknown function (DUF1195)	-1.051	9.49E-06
At5g65730	XTH6 (xyloglucan endotransglucosylase/hydrolase 6)	-1.134	0.00176
At5g65800	ACS5 (ACC synthase 5); 1-aminocyclopropane-1-carboxylate synthase (ACS) is encoded by a multigene famil	-1.45	1.01E-05
At5g65860	ankyrin repeat family protein	-1.008	1.34E-05
At5g65920	ARM repeat superfamily protein	1.475	9.13E-07
At5g65970	ATMLO10 (MILDEW RESISTANCE LOCUS O 10); A member of a large family of seven-transmembrane do	-1.244	3.24E-07
At5g66020	ATSAC1B (SUPPRESSOR OF ACTIN 1B); Mutants in this gene are unable to express female sterility in resp	1.427	1.88E-05
At5g66050	Wound-responsive family protein	1.02	7.15E-08
At5g66070	RING/U-box superfamily protein	1.78	4.73E-06
At5g66090	unknown protein	1.536	3.47E-08
At5g66100	winged-helix DNA-binding transcription factor family protein	1.193	1.61E-07
At5g66170	STR18 (sulfurtransferase 18); Encodes a thiosulfate sulfurtransferase/rhodanese.	1.005	3.39E-05
At5g66210	CPK28 (calcium-dependent protein kinase 28); member of Calcium Dependent Protein Kinase	1.443	0.0002
At5g66330	Leucine-rich repeat (LRR) family protein	-1.224	4.91E-08
At5g66440	unknown protein	-1.147	7.94E-08
At5g66480	unknown protein	1.349	1.67E-07
At5g66580	unknown protein	-1.202	4.76E-05
At5g66590	CAP (Cysteine-rich secretory proteins, Antigen 5, and Pathogenesis-related 1 protein) superfamily protein	-1.827	5.35E-10
At5g66620	DAR6 (DA1-related protein 6)	2.019	6.03E-07
At5g66631	Tetratricopeptide repeat (TPR)-like superfamily protein	1.264	3.49E-07

At5g66850	MAPKKK5 (mitogen-activated protein kinase kinase kinase 5); member of MEKK subfamily	1.811	2.54E-07
At5g66880	SNRK2-3 (SUCROSE NONFERMENTING 1 (SNF1)-RELATED PROTEIN KINASE 2-3); encodes a membe	1.569	5.38E-09
At5g66890	Leucine-rich repeat (LRR) family protein	1.071	0.00625
At5g66900	Disease resistance protein (CC-NBS-LRR class) family	1.242	5.10E-07
At5g67060	HEC1 (HECATE 1)	-1.011	0.00118
At5g67090	Subtilisin-like serine endopeptidase family protein	-1.174	6.35E-06
At5g67140	F-box/RNI-like superfamily protein	1.236	2.79E-06
At5g67250	SKIP2 (SKP1/ASK1-interacting protein 2); Encodes an SKP1 interacting partner (SKIP2).Encodes an F-box p	1.225	1.21E-07
At5g67310	CYP81G1 (cytochrome P450, family 81, subfamily G, polypeptide 1); member of CYP81G	2.632	4.73E-05
At5g67340	ARM repeat superfamily protein	1.956	1.31E-07
At5g67350	unknown protein	1.934	7.82E-08
At5g67370	Protein of unknown function (DUF1230)	1.494	3.29E-05
At5g67390	unknown protein	-1.219	6.54E-05
At5g67450	AZF1 (zinc-finger protein 1); Encodes zinc-finger protein. mRNA levels are elevated in response to low tempe	1.79	3.60E-06
At5g67540	Arabinanase/levansucrase/invertase	1.483	1.12E-08
At5g67620	unknown protein	-1.693	2.18E-06
AtCg00100	tRNA-Gly; tRNA-Gly	-1.328	0.00033
AtCg00130	ATPF (ATPase, F0 complex, subunit B/B', bacterial/chloroplast); ATPase F subunit.	-1.239	0.00029
AtCg00640	RPL33 (ribosomal protein L33); encodes a chloroplast ribosomal protein L33, a constituent of the large subuni	-1.262	0.00012
AtCg00650	RPS18 (ribosomal protein S18); chloroplast-encoded ribosomal protein S18	-1.154	1.73E-06
AtCg00750	RPS11 (ribosomal protein S11); 30S chloroplast ribosomal protein S11	-1.136	3.21E-07
AtCg00770	RPS8 (ribosomal protein S8); chloroplast 30S ribosomal protein S8	-1.316	1.81E-06
AtCg00780	RPL14 (ribosomal protein L14); encodes a chloroplast ribosomal protein L14, a constituent of the large subuni	-1.466	1.20E-07
AtCg00790	RPL16 (ribosomal protein L16); chloroplast gene encoding a ribosomal protein L16, which is a constituent of !	-1.378	9.31E-08
AtCg00800	RPS3 (RESISTANCE TO PSEUDOMONAS SYRINGAE 3); encodes a chloroplast ribosomal protein S3, a co	-1.097	5.52E-07
AtCg00810	RPL22 (ribosomal protein L22); encodes a chloroplast ribosomal protein L22, a constituent of the large subuni	-1.028	3.11E-06
AtCg00840	RPL23 (RIBOSOMAL PROTEIN L23); One of two chloroplast genes that encode chloroplast ribosomal protei	-1.036	8.19E-06
AtCg00930	tRNA-Ile; tRNA-Ile	-1.595	1.50E-06
AtCg01040	YCF5 (Cytochrome C assembly protein); hypothetical protein	-1.17	3.12E-05
AtMg00160	COX2 (cytochrome oxidase 2); cytochrome c oxidase subunit 2	1.065	0.0002
AtMg01370	unknown protein.; hypothetical protein	-1.152	1.24E-07
